# Anti-colorectal cancer effects of IRX4 and sensitivity studies to oxaliplatin

**DOI:** 10.3389/fimmu.2025.1581244

**Published:** 2026-01-21

**Authors:** Mei Jia, Dexian Kong, Jiaxu Fan, Bingqian Heng, Jian Zhao, Binghui Li, Shuang Liu, Peng Kong, Yabin Liu

**Affiliations:** 1 Department of General Surgery, the Fourth Hospital of Hebei Medical University, Shijiazhuang, China; 2 Department of Endocrinology, Fourth Affiliated Hospital, Hebei Medical University, Shijiazhuang, China; 3 Obstetrics and Gynecology Department, Shijiazhuang People's Hospital, Shijiazhuang, Hebei, China; 4 General Surgery Department, Bethune International Peace Hospital, Shijiazhuang, Hebei, China; 5 Department of Biochemistry and Molecular Biology, College of Basic Medicine, Key Laboratory of Neural and Vascular Biology of Ministry of Education, Key Laboratory of Vascular Biology of Hebei Province, Hebei Medical University, Shijiazhuang, China

**Keywords:** chemosensitivity, colorectal cancer, DNA methylation, IRX4, oxaliplatin

## Abstract

**Introduction:**

Colorectal cancer (CRC) remains one of the most lethal malignancies globally. Chemoresistance or reduced chemosensitivity is a key factor contributing to treatment failure in CRC, particularly in patients with advanced-stage disease. Iroquois homeobox protein 4 (IRX4), a transcription factor expressed in multiple tissues, has been demonstrated to be implicated in the progression of many cancers. Based on this background, IRX4 identified through Illumina Infinium 935K methylation chip analysis was chosen to explore its potential role in CRC.

**Methods:**

Following initial screening via the Illumina Infinium 935K methylation chip, we further confirmed the hypermethylation of IRX4 in CRC tissues using pyrosequencing technology. The expression levels of IRX4 were determined by immunofluorescence (IF) staining, reverse transcription-quantitative polymerase chain reaction (RT-qPCR), and western blot (WB) assays. *In vitro* cell experiments evaluated the effects of IRX4 overexpression on the proliferation, invasion, migration, and apoptosis of CRC cells, as well as its impact on the chemosensitivity to oxaliplatin (OXA). Mechanistically, after IRX4 overexpression, the phosphorylation level of epidermal growth factor receptor (EGFR) in CRC cells was detected. Luciferase reporter gene assays and immunofluorescence staining were used to investigate the effects of IRX4 overexpression on the activation of the nuclear factor-κB (NF-κB) pathway and the nuclear translocation of the p65 subunit.

**Results:**

IRX4 was downregulated in CRC tissues and cell lines, and its expression was negatively correlated with tumor invasion depth and poor prognosis. In vitro experiments demonstrated that the overexpression of IRX4 inhibited CRC cell proliferation, migration, and invasion and promoted apoptosis. Moreover, IRX4 overexpression enhanced the chemosensitivity of CRC cells to OXA. Mechanistically, IRX4 overexpression significantly inhibited the TNF-α-induced NF-κB transcriptional activity and suppressed the nuclear translocation of NF-κB p65 in CRC cells. Furthermore, combined treatment with OXA and IRX4 overexpression significantly reduced the levels of EGFR and the phosphorylationlevels of EGFR downstream signaling molecules.

**Conclusion:**

These findings suggest that IRX4 may be a prognostic biomarker and therapeutic target in CRC. Moreover, IRX4 might regulate CRC progression and chemosensitivity by inhibiting the NF-κB /EGFR pathway, suggesting its potential as a therapeutic target to improve chemotherapeutic efficacy in CRC.

## Introduction

Colorecal cancer (CRC) is the third most common malignancy worldwide, with nearly 2 million people newly diagnosed with CRC and nearly 900,000 deaths from CRC each year (1). In China, the National Cancer Center reported approximately 517,100 new CRC cases and 240,000 deaths in 2022, making CRC the second most common cancer and the fourth leading cause of cancer mortality in the country (2). Treatment modalities for CRC include surgery, chemotherapy, radiotherapy, and targeted therapy, with chemotherapy playing a pivotal role. Preoperative neoadjuvant chemotherapy increases resection rates and preserves sphincter function, while postoperative adjuvant chemotherapy improves survival in patients at high risk of recurrence and metastasis (3). Advanced CRC often presents with metastasis, particularly to the liver and lungs, where chemotherapy serves as a key strategy to extend survival, slow disease progression, improve quality of life, and enable subsequent therapies (4). However, the development of chemotherapy resistance remains a significant barrier, reducing the effectiveness of chemotherapeutic agents and adversely impacting long-term survival. Identifying the mechanisms underlying chemotherapy resistance is, therefore, of paramount clinical importance.

Methylation dysregulation has been closely linked to tumorigenesis and contributes to chemotherapy resistance via various mechanisms. Methylation-induced silencing of tumor suppressor genes, for instance, can result in the loss of chemotherapy drug sensitivity in tumor cells (5). Additionally, studies suggest that methylation aberrations can suppress the transcription of specific genes, leading to the silencing of tumor suppressor genes or activation of oncogenes, thus promoting tumorigenesis (6, 7). Given its potential, methylation may serve as a novel target for cancer research and therapy. Illumina methylation array analysis has revealed significant hypermethylation of IRX4 in CRC, suggesting its involvement in the initiation and progression of CRC. IRX4 is a transcription factor expressed in various tissues, and its methylation dysregulation has been implicated in tumorigenesis. For example, hypermethylation of IRX4 has been shown to promote the growth of human pancreatic cancer cells (8). Moreover, dysregulated IRX4 expression may contribute to chemotherapy resistance (9, 10). Consequently, IRX4 and its methylation status could serve as novel biomarkers for predicting chemotherapy resistance and may represent promising therapeutic targets for cancer treatment.

Previous studies indicate that IRX4 may influence the sensitivity of chemotherapeutic agents by modulating cancer cell behavior, with relevant reports emerging from research on non-small cell lung cancer (NSCLC) and prostate cancer (PC) (9, 10). This prompts the investigation of a potential association between IRX4 and OXA, a first-line chemotherapy drug in CRC. OXA, a third-generation platinum-based agent introduced in France in 1996, has become a cornerstone in clinical oncology, particularly for gastrointestinal cancers. Like its predecessors, cisplatin and carboplatin, OXA exerts its anticancer effects by inhibiting DNA replication and transcription, leading to cell apoptosis.

Recent studies have also shown that OXA can induce immunogenic cell death, thereby activating anti-cancer immune responses, which offers a distinct advantage in cancer therapy (11–13). OXA demonstrates significant therapeutic efficacy in CRC, with relatively low toxicity and reduced tumor resistance, making it a first-line treatment for CRC and increasingly recognized in the management of other gastrointestinal malignancies such as gastric and pancreatic cancer. The FOLFOX and XELOX regimens, which combine OXA with 5-fluorouracil (5-FU) and capecitabine, respectively, are widely used for metastatic CRC treatment (14–16). Furthermore, ongoing research continues to explore OXA’s efficacy in various solid tumors, including liver and ovarian cancer, with the goal of expanding its therapeutic potential. However, reduced sensitivity to OXA remains a major challenge, contributing to chemotherapy failure in some patients. As a result, strategies to enhance OXA sensitivity are critical for improving survival rates and prognosis in patients with CRC.

IRX4, a member of the Iroquois family of transcription factors, is located at the 5p15.33 chromosomal locus and plays a vital role in embryonic development, particularly in the formation of organs such as the heart and prostate. As the first identified cardiac transcription factor, IRX4 is predominantly expressed in ventricular myocytes, where its expression is tightly regulated through specific gene regulation mechanisms (17). Increasing evidence suggests that IRX4’s role extends beyond cardiac development and into oncology. Notably, its involvement in PC has garnered significant attention. Studies have demonstrated that IRX4 expression is associated with PC susceptibility, where its levels directly influence the proliferative capacity of PC cells. The vitamin D receptor regulates this expression, with IRX4 silencing promoting PC cell growth, while overexpression of IRX4 yields the opposite effect (18, 19). Additionally, preliminary research suggests that IRX4 could serve as a plasma biomarker for breast cancer, where its expression is upregulated (20). Elevated IRX4 expression has also been observed in lung cancer, correlating with poor prognosis in patients, although the underlying mechanisms remain unclear (21). In pancreatic cancer, IRX4 expression is epigenetically regulated, with decreased levels potentially contributing to enhanced tumor cell proliferation, migration, and invasiveness (8). While research on IRX4 in CRC is limited, studies exploring its functions in other cancer types offer promising insights into its potential tumor-suppressive roles and therapeutic value in CRC.

This study first examined IRX4 expression in CRC and its correlation with methylation levels through bioinformatics analysis. The relationship between IRX4 expression and CRC prognosis was also assessed. Using clinical specimens, IRX4 expression was further validated at both the molecular and protein levels. Statistical analyses were performed to explore the correlation between IRX4 expression and clinical parameters, highlighting its clinical relevance in CRC. Finally, *in vitro* experiments were conducted to validate the potential role of IRX4 in CRC and its impact on chemotherapy sensitivity.

## Materials and methods

### Integrated analysis of DNA methylation and gene expression with functional validation

To investigate the potential role of methylation in CRC, we compared gene methylation levels between normal and CRC tissues. DNA methylation data for CRC were obtained from The Cancer Genome Atlas (TCGA) database via the Genomic Data Commons (GDC) data portal version 2.0 (https://portal.gdc.cancer.gov/projects/). The methylation differences between normal tissues and CRC tissues were analyzed with the R language ChAMP package, applying a threshold of *P* < 1–^10^ and |logFC|>0.02 to screen for differentially methylated genes. At the same time, the RNA-seq TPM data of normal tissues and CRC tissues were sourced from TCGA and Genotype Tissue Expression (GTEx Version 10) database (https://www.gtexportal.org/home/), and the expression differences were analyzed by the R language limma package after log2(TPM + 1) transformation, and *P* < 0.05 and |logFC|>1 were regarded as genes were differentially expressed between the two groups. The intersection of the two was used to obtain the CRC methylation differential gene set, with core genes screened by logFC value sorting. Expression differences were illustrated using violin plots, while correlation analysis identified closely related genes, visualized through chordal plots. The subcellular localization of these key regulatory genes was clarified using the Genecard database (Version 5.22; https://www.genecards.org/). Difference results and intersection results were shown by volcano, heatmap and Venn diagrams, respectively, and visualized by R language pheatmap package and ggplot2 package. The acquisition and aggregation of all bioinformatics data were finalized on December 20, 2024.

Based on the findings from the preliminary Infinium 935K methylation microarray and bioinformatics differential analysis, pyrophosphate sequencing was performed to validate the methylation status of the IRX4 promoter region. Genomic DNA was extracted from tissue samples using the Qiagen DNeasy Blood & Tissue Kit (50) (Catalog No. 69504, QIAGEN, Germany).DNA was quantified with Qubit^®^ 3.0 Fluorometer (Thermo Fisher, USA), and purity verified by NanoDrop (A260/A280 >1.8).Bisulfite conversion was performed using the EZ DNA Methylation Kit (Zymo Research, USA), followed by post-conversion quantification. Two fragments within the IRX4 promoter region were selected for methylation analysis. Target sequences were amplified via Polymerase Chain Reaction (PCR) using gene-specific primers (Primers are listed in **Supplementary Table 1**). Pyrophosphate sequencing was then performed by a Pyrosequencing detector (PyroMark Q96 ID, QIAGEN, Germany) to record the signal intensity in real time and accurately detect the methylation level of the target fragments. Delta beta values were used for difference analysis of methylation sites and statistical methods were used to assess the statistical significance of differences between groups.

### Prognostic analysis

To investigate the impact of the key gene IRX4 on CRC prognosis, two CRC prognostic datasets, GSE37892 and GSE143985, were retrieved from the Gene Expression Omnibus (GEO) database (https://www.ncbi.nlm.nih.gov/geo/). Based on IRX4 expression levels in CRC, the optimal cutoff value was determined, and Cox regression analysis was conducted to assess prognosis. Kaplan-Meier survival curves were gen-erated to visualize the results, with *P* < 0.05 considered statistically significant.

### Enrichment analysis

To elucidate the mechanisms through which IRX4 may influence CRC progression, an enrichment analysis was performed. Samples were stratified into high- and low-expression groups based on the median expression level of IRX4, with the low-expression group serving as the control. Differential expression analysis was conducted using the limma package in R, identifying genes with a P-value < 0.05 and |logFC| > 1 as differentially expressed between the two groups, leading to the identification of IRX4-associated genes. Gene Set Enrichment Analysis (GSEA) was performed with the “clusterProfiler” package to further investigate the potential roles of IRX4 in both pan-cancer and CRC. GSEA results were presented using correlation circle plots, ridge plots, and other appropriate visualizations.

### Correlation analysis of IRX4 with RNA and DNA methylation

To investigate the relationship between IRX4 and RNA methylation, relevant gene sets associated with m1A, m5C, and m6A RNA modifications were reviewed from the literature. Spearman correlation analysis was performed to examine the relationship between IRX4 expression and RNA methylation across various cancers, with the results visualized as a heatmap. Based on the median IRX4 expression level, samples were grouped into high- and low-expression categories to assess the expression of m1A/m5C/m6A-related genes in CRC. Differences in DNA methylation levels of IRX4-associated methylation sites between normal and CRC tissues were analyzed, with the top three sites showing the most significant differences presented in box plots.

### Correlation between OXA and IRX4 and clinical correlation analysis

To further explore the potential link between OXA and IRX4, predictive analysis was performed using the Search Tool for Interactions of Chemicals (STITCH) database (http://stitch.embl.de/), and the results were visualized in a network diagram. Additionally, genes associated with OXA were retrieved from the GeneCards database and intersected with IRX4-related DEGs. The overlapping genes were identified and visualized in a Venn diagram. To determine the clinical relevance of IRX4 as a biomarker, we downloaded two CRC datasets, GSE124808 and GSE77932, associated with OXA treatment efficacy from the GEO database, pooled the two datasets, and divided them into oxaliplatin-responsive and non-responsive groups using the R package “limma” to remove batch effects. OXA difference analysis was performed, and the results were displayed in the form of box plots. *P* < 0.05 was considered statistically different between the two groups.

### Clinical specimens and data collection

Clinical specimens used in this study were collected from cancerous tissues and corresponding adjacent normal tissues (more than 10 cm from the tumor margin) of patients with CRC, confirmed by pathology at the Department of General Surgery, Fourth Hospital of Hebei Medical University, between December 2022 and March 2023. Tissues were immediately frozen in liquid nitrogen following surgical resection and subsequently stored at -80°C. Informed consent was obtained from all patients prior to participation. The ages of the patients ranged from 34 to 83 years, with a mean age of 64.05 ± 9.86 years. The clinical exclusion criteria were as follows: (1) patients with a history of preoperative chemotherapy or radiotherapy; (2) metastatic CRC; (3) patients without pathological confirmation before surgery. A total of 80 CRC tissue specimens (experimental group) and 80 corresponding adjacent normal tissue samples (control group) were included. Of these, 42 patients were male and 38 were female.

### Cell line culture

The normal human intestinal epithelial cell line FHC and CRC cell lines HCT116, SW480, HCT8, and Caco2 were purchased from the American Type Culture Collection (ATCC; Manassas, VA, USA). The cell culture conditions were as follows: FHC and SW480 cell lines were cultured in GibcTM Dulbecco’s Modified Eagle Medium (DMEM) high-glucose medium (Cat No. 11965092), HCT116 and HCT-8 cell lines in Gibco™ RPMI 1640 medium (Cat No. 11875093), and Caco2 cells in Gibco™ Minimum Essential Medium (MEM) (with NEAA) (Cat No. 10370021). MEM medium was supplemented with 20% fetal bovine serum (ExCell, Cat No. FCS500), while the other media were supplemented with 10% fetal bovine serum. All media contained 100 U/mL penicillin and 0.1 mg/mL streptomycin solution (SEVEN, Cat No. 15140122). Cells were cultured in a humidified incubator at 37°C with 5% CO2. When cell density reached 80%-90%, cells were passaged at a 1:2 ratio every 2–3 days. Cells in the logarithmic growth phase were used for subsequent experiments.

### RT-qPCR

Total mRNA was extracted from CRC tissues and corresponding adjacent normal tissues using the Trizol method, as well as from four CRC cell lines and normal intestinal epithelial cell lines. The concentration and optical density (OD) values of the extracted total mRNA were measured using a spectrophotometer, and the mRNA was then reverse-transcribed into complementary DNA (cDNA). Next, RT-qPCR was performed on RT-qPCR instrument. The primer sequences for PCR amplification are listed in in **Supplementary Table 1**, with glyceraldehyde-3-phosphate dehydrogenase (GAPDH) used as the internal control for IRX4. All experiments were performed in triplicate. After the reaction, the relative expression levels were calculated using the 2-ΔΔCt method based on the cycle threshold (Ct) values obtained for each well.

### Western blot analysis

CRC tissues and adjacent normal tissues were homogenized, and total proteins were extracted using RIPA lysis buffer. Cells were lysed with ice-cold RIPA lysis buffer to extract total proteins. The protein concentration of the samples was determined using the BCA protein assay, with absorbance measured at 570 nm via a microplate reader. Following protein quantification, the samples were separated by sodium dodecyl sulfate-polyacrylamide gel electrophoresis (SDS-PAGE) and transferred onto a polyvinylidene fluoride (PVDF) membrane. The membrane was blocked with 5% non-fat milk at room temperature for 1 hour to minimize non-specific binding. Primary antibodies targeting IRX4 and GAPDH were incubated overnight at 4°C. After three washes with Tris-buffered saline with Tween (TBST), the membrane was incubated with rabbit and mouse secondary antibodies for 1 hour at room temperature. Chemi-luminescent detection was performed using a chemiluminescence imaging system, and images were captured and stored. Grayscale analysis was conducted using ImageJ and GraphPad Prism 10 software.

### IF staining and laser confocal scanning analysis

Logarithmic-phase cells were seeded into 12-well plates containing 14 mm coverslips at the bottom, at a density of approximately 30,000 cells per well. After 24 hours of culture, cells were collected and fixed with 4% paraformaldehyde (PFA) at room temperature for 15 minutes. Permeabilization was performed using 0.3% Triton X-100 at room temperature for 10 minutes. The cells were then blocked with a 3% bovine serum albumin (BSA) solution containing goat serum at 4°C for 2 hours. After washing with phosphate-buffered saline (PBS), the cells were incubated overnight at 4°C with a primary antibody (1:200). Following removal of unbound primary antibody, the cells were incubated for 1 hour at room temperature in the dark with a fluorescently labeled rabbit secondary antibody (1:200), followed by nuclear staining for 10 minutes. Finally, cells were mounted with a mounting medium and observed under a laser scanning confocal microscope (Nikon, A1RHD25), with images captured and stored.

### Plasmid construction and cell transfection

A biological company was commissioned to design and construct the IRX4 pcDNA3.1(+) plasmid. The overexpression plasmid was used as the experimental group, and the empty vector plasmid was used as the control group. The primer sequences for sequencing are listed in **Supplementary Table 1**. Cells were seeded into 6-well plates with two replicate wells for each cell type. Transfection was performed when cells reached approximately 70% confluence. After transfection, cells were cultured for 72 hours before harvesting for RT-qPCR, WB, and IF experiments to assess mRNA and protein expression levels of the target gene, evaluating transfection efficiency.

### Cell counting kit-8 cytotoxicity assay

**Figure 1:**
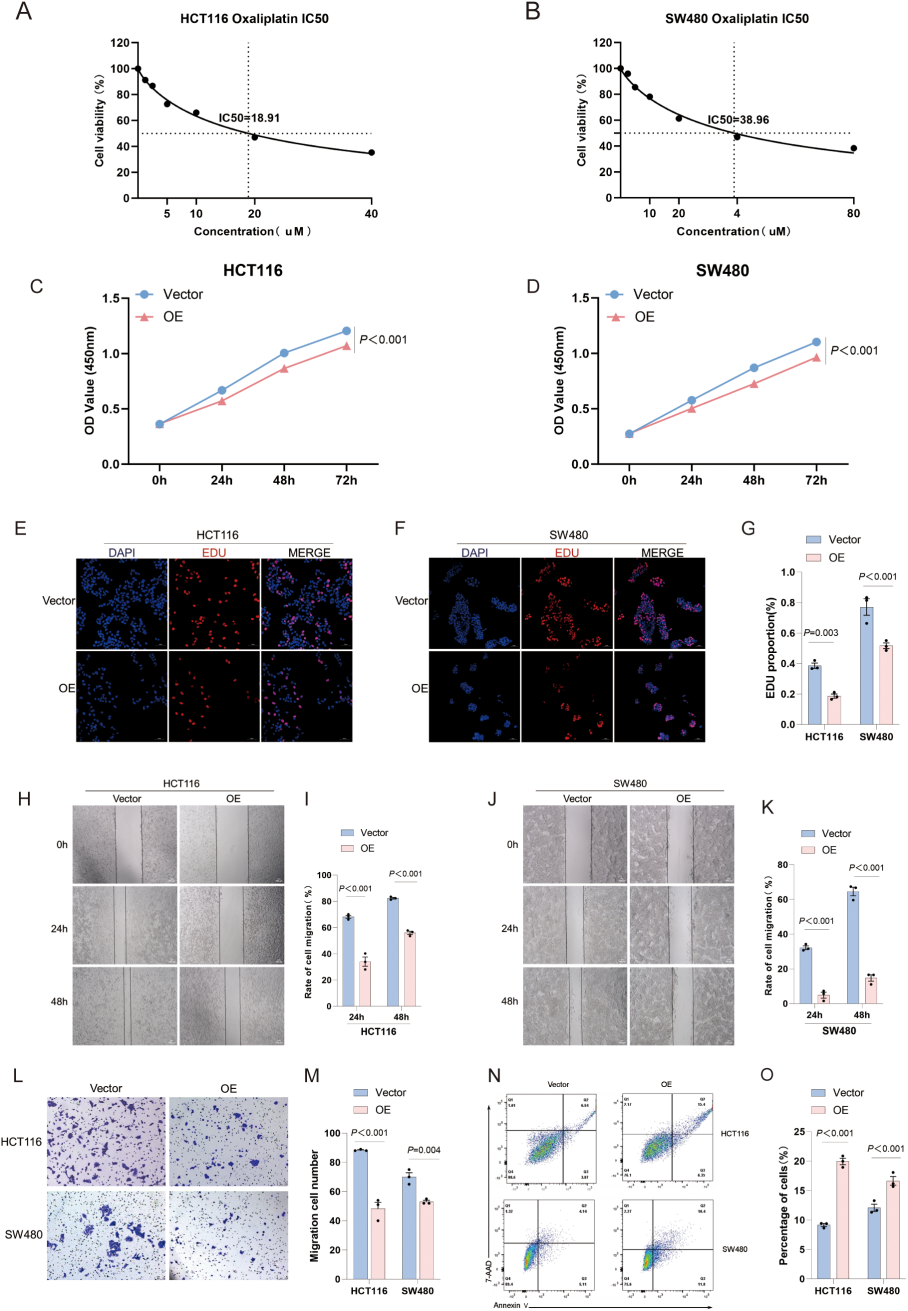
IRX4 Overexpression Alters the Biological Behaviors of CRC Cells. **(A, B)** IC50 values for HCT116 and SW480 cells. **(C, D)** CCK-8 proliferation assay results for HCT116 and SW480 cells. **(E–G)** EDU proliferation assay results for HCT116 and SW480 cells. **(H–K)** Scratch assay results for HCT116 and SW480 cells. **(L, M)** Transwell invasion assay results for HCT116 and SW480 cells. **(N, O)** Apoptosis analysis results for HCT116 and SW480 cells. Vector: Control group (empty vector); OE: Overexpression plasmid group. Data are presented as the mean ± SEM (n = 3). *P* < 0.05 is considered the difference to be statistically significant.

To assess the cytotoxicity of OXA on CRC cells, log-phase cells were harvested and suspended to prepare a cell suspension, which was seeded evenly into a 96-well plate, with approximately 3,000 cells per well. Based on preliminary results, OXA treatment concentrations for HCT116 cells were 5 μM, 10 μM, 20 μM, and 40 μM, and for SW480 cells, 10 μM, 20 μM, 40 μM, and 80 μM. Each concentration included three replicates. At each corresponding time point, 10 μL of CCK-8 reagent was added to each well, and cells were incubated at 37°C for an additional 2 hours. Absorbance at 450 nm was measured using a microplate reader, and half maximal inhibitory concentration (IC50) values were calculated (**Figures 1A, B**). Based on the IC50 values and relevant literature, 10 μM OXA for HCT116 cells and 20 μM OXA for SW480 cells were selected for 24-hour stimulation in subsequent experiments.

### CCK-8 cell proliferation assay

Logarithmic-phase cells were harvested, suspended to prepare a cell suspension, and then evenly seeded into a 96-well plate at approximately 1,000 cells per well, with six replicate wells for each group. The 96-well plate was incubated at 37°C for various time points (24 h, 48 h, 72 h, and 96 h). At each predetermined time point, 10 μL of CCK-8 reagent was added to each well, and the plate was returned to the incubator for an additional 2 hours. After incubation, absorbance at 450 nm was measured using a microplate reader. Cell viability was assessed based on the OD values of the experimental and control groups.

### 5-ethynyl-2’-deoxyuridine cell proliferation assay

For assessing cell proliferation, HCT116 and SW480 cells from different transfection and drug treatment groups were cultured to the logarithmic phase. A 10 μM concentration of EdU was added to the culture medium, and cells were incubated at 37°C for 2 hours. After EdU treatment, cells were fixed with 4% PFA solution for 30 minutes. The cells were then permeabilized with 0.3% Triton X-100 at room temperature for 20 minutes. Fluorescently labeled Click reagents were added to the cells, and cells were incubated at room temperature in the dark for 60 minutes. After the treatment, the cells were stained with DAPI for 10 minutes to visualize the DNA. Cell images were captured using a laser-scanning confocal microscope. The proportion of EdU-positive cells was calculated, and data analysis and comparisons were performed.

### Cell scratch assay

Three horizontal lines, spaced 1 cm apart, were drawn on the bottom of a 6-well plate using a black marker. HCT116 and SW480 cells were transfected and cultured to approximately 70% confluence. A 200 μL pipette tip was used to create three vertical scratches on the cell monolayer, perpendicular to the horizontal lines in the 6-well plate. After washing with PBS, cell images were captured at 0 hours. The cells were then incubated for 24, 48, and 72 hours, with images captured at each time point to assess cell migration and calculate the migration area.

### Transwell cell invasion assay

The basement membrane gel was thawed overnight at 4°C, while Transwell inserts and 24-well plates were pre-cooled at -20°C. The gel was diluted to 1 mg/mL, and 50 μL was added to the upper chamber of each well, followed by incubation at 37°C for 3 hours. Approximately 1 × 10^5^ cells were seeded in the upper chamber, and 500 μL of DMEM containing 10% FBS was added to the lower chamber. After 24 hours of incubation, cells were fixed with 4% PFA for 30 minutes and then stained with 1% crystal violet for 20 minutes. Non-migrated cells in the upper chamber were gently removed using a cotton swab. The cells were photographed and counted under an inverted microscope.

### Flow cytometry assay for cell apoptosis

Following transfection and drug treatment, cells were digested with 0.25% trypsin without EDTA, washed twice with pre-cooled 1 × PBS, and adjusted to a concentration of 1 × 10^6^ cells/mL. The cells were resuspended in Annexin V Binding Buffer (Bio-Legend, cat. no. 422201). A 100 μL aliquot of the suspension was transferred to a 5 mL flow cytometry tube, and 5 μL of fluorescently labeled Annexin V (BioLegend, cat. no. 640906) and 7-AAD viability dye (BioLegend, cat. no. 420403) were added for staining. The cells were gently vortexed and incubated in the dark at room temperature for 15 minutes. Following incubation, 300 μL of Annexin V Binding Buffer was added to each tube, and immediate analysis was performed using a flow cytometer. Data were analyzed using FlowJo version 10.8.1 software.

### Detection of luciferase NF-κB reporter gene

Cells (1 × 10^6^ cells/well) were cultured in 6-well plates and then transfected with the pNFκB-TA-luc-CP reporter plasmid and pcDNA3.1-IRX4 plasmids using Lipo8000™ Transfection Reagent (Beyotime, cat. no. C0533, China). Twenty-four hours after transfection, cells were treated with TNF-α (20 ng/ml) for 12 h. The supernatant was removed and washed twice with PBS buffer. Cells were lysed with Reporter Lysis Buffer (Beyotime, RG009S, China). After thorough lysis, the supernatant was collected following centrifugation at 12,000 × g for 30 min at 4°C. 4 duplicate wells were set for each sample, 100ul of sample was taken, and the same volume of reporter cell lysate was taken as blank control, 100μL of firefly luciferase detection reagent was taken from each well, and relative light unit was determined by multifunctional microplate reader after mixing well.

### Evaluation of extracellular vesicles mRNA in the culture medium supernatant

HT29 cells were seeded into 6-well plates at a density of 1 × 10^6^ cells/well and allowed to reach approximately 70% confluence on the day of transfection. Twenty-four hours after transfection, the conditioned medium was collected for subsequent analysis. Cell debris was removed by centrifugation at 700 × g for 10 min at 4°C, and the supernatant was transferred to a new 1.5 mL tube. This supernatant was then centrifuged at 15,000 × g for 30 min at 4°C to precipitate large EVs. The supernatant was discarded, and the pellet was retained. Total RNA was extracted using TRIzol reagent. mRNA was reverse-transcribed into cDNA and analyzed by real-time RT-PCR. All experiments were independently repeated in triplicate. The cycle threshold value of each gene was normalized to that of GAPDH, and relative gene expression was calculated using the 2^−^ΔΔCt method.

### Statistical analysis

All data are representative of at least three separate experiments. Statistical analysis was performed using SPSS 29.0 and GraphPad Prism 10, with data expressed as mean ± standard deviation. The t-test was used for comparisons between two groups, and one-way analysis of variance was applied to assess IRX4 expression in CRC. The association between IRX4 expression (grouped as high/low expression) and clinicopathological characteristics of CRC was assessed using chi-square tests. Multiple testing correction was performed via the Benjamini-Hochberg procedure to control the false discovery rate. Binary logistic regression analysis was further employed to determine whether IRX4 expression serves as an independent factor. Statistical significance was defined as *P < 0.05, **P < 0.01, ***P < 0.001, and ns denoted non-significant. *P* < 0.05 is considered statistically significant.

## Results

### IRX4 as a methylation difference hub gene

**Figure 2:**
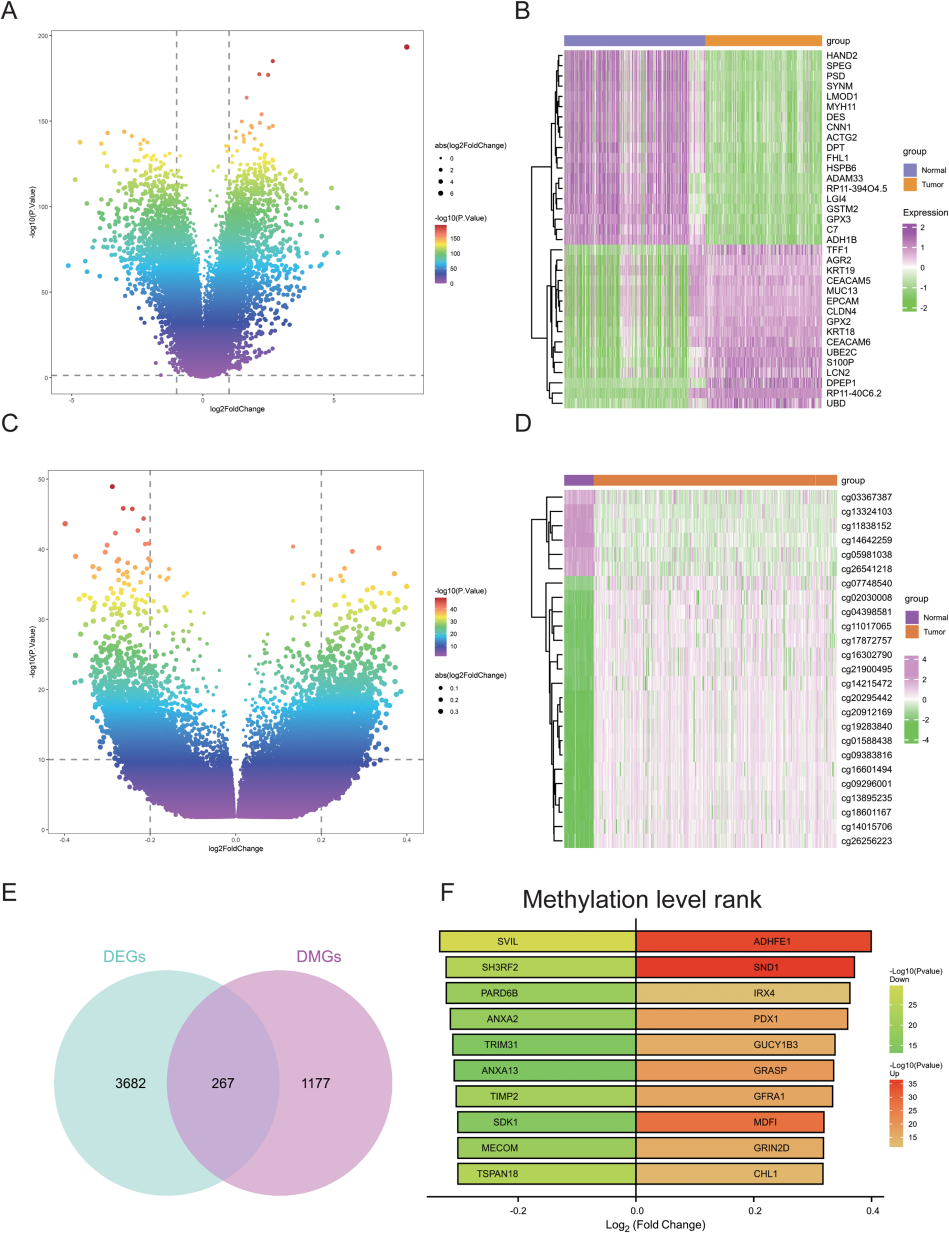
Screening of Core Differential Methylation Genes. **(A)** Volcano plot of differential gene expression analysis between normal and CRC tissues. **(B)** Heatmap of differential gene expression between normal and CRC tissues. **(C)** Volcano plot of differential methylation level analysis be-tween normal and CRC tissues. **(D)** Heatmap of differential methylation levels between normal and CRC tissues. **(E)** Venn diagram showing the overlap of differentially expressed and differentially methylated genes between normal and CRC tissues. **(F)** Ranking of methylation levels for the intersecting genes.

Differential expression analysis between CRC and normal tissues identified a set of DEGs, which were visualized using volcano plots (**Figure 2A**). Compared to normal tissues, genes such as TFF1, AGR2, and KRT19 were upregulated in CRC, while genes such as HAND2, SPEG, and PSD were downregulated (**Figure 2B**). Additionally, methylation differences were analyzed, and the distribution of differential methylation sites was also displayed in the form of volcano plots (**Figure 2C**). The methylation analysis revealed that several methylation sites, including cg03367387, cg13324103, and cg11838152, exhibited decreased methylation levels in CRC compared to normal tissues, while sites such as cg07748540, cg02030008, and cg04398581 showed increased methylation levels in CRC (**Figure 2D**). Intersection of genes corresponding to differential methylation sites with DEGs yielded a total of 267 overlapping genes (**Figure 2E**). These genes were ranked according to the logFC of their methylation levels, and the top 10 upregulated and top 10 downregulated genes were displayed. Notably, the methylation levels of ADHFE1, SND1, and IRX4 were significantly upregulated, while the methylation levels of SVIL, SH3RF2, and PARD6B were significantly downregulated (**Figure 2F**). A literature review highlighted the limited research on IRX4 in CRC and the unclear mechanisms governing its role in CRC progression. As a result, IRX4 was selected as a hub gene for further investigation.

### Analysis of differential methylation levels of CpG sites in CRC tissues

**Figure 3:**
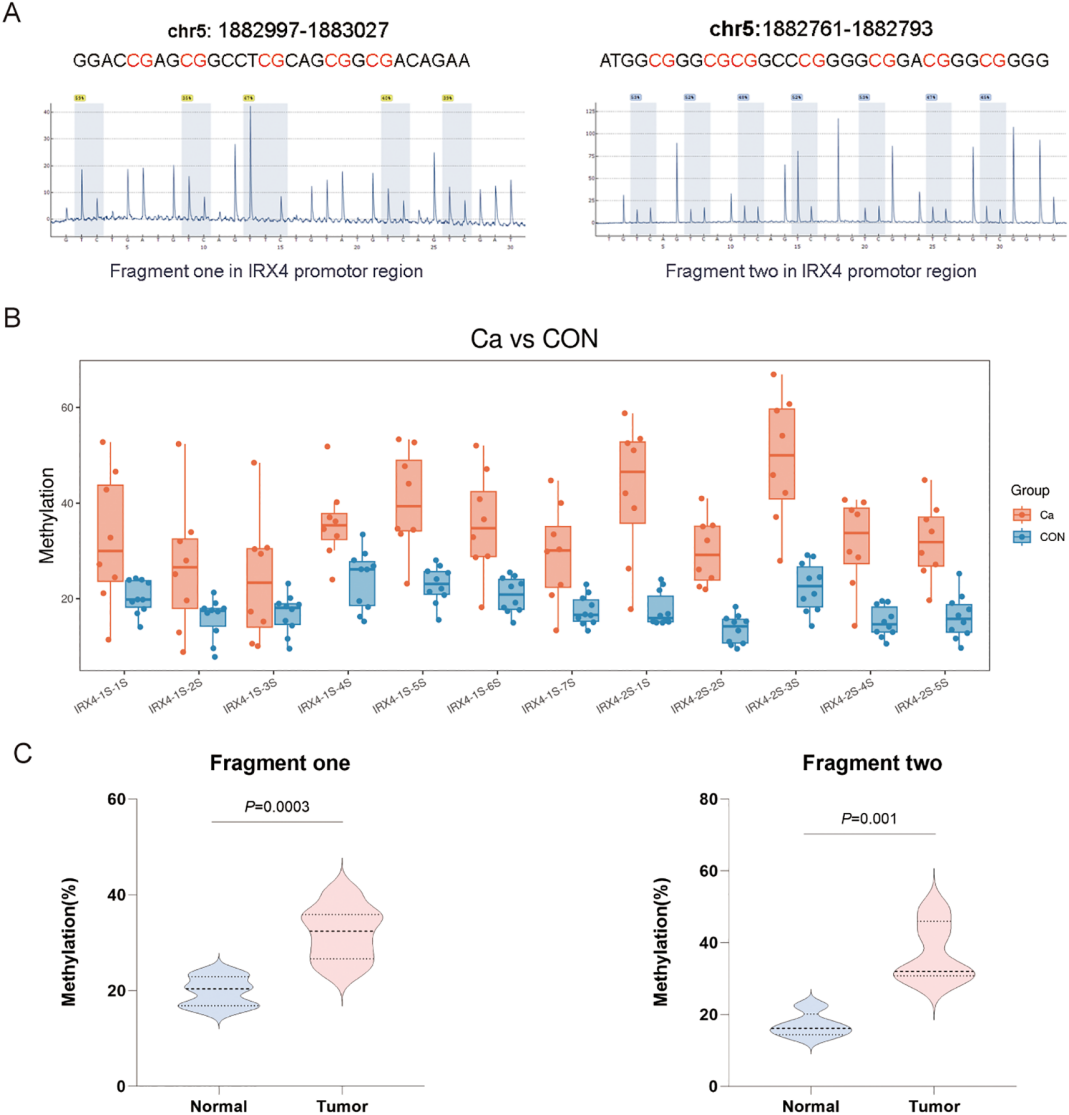
Differential Methylation Analysis of CpG Sites in CRC Tissues. **(A)** Schematic diagram of two fragment sequences within the IRX4 promoter region, with CpG sites marked in red. Fragment 1 contains five methylation sites, and Fragment 2 contains seven. A pyrosequencing heatmap below shows the methylation percentages of each CpG site. **(B)** Intergroup difference analysis of methylation sites within the two IRX4 promoter fragments, presented as a bee swarm plot. **(C)** Average methylation levels of Fragment 1 and Fragment 2 in CRC and adjacent normal tissues. *P* < 0.05 is considered the difference to be statistically significant.

Methylation chip and bioinformatics analysis revealed a significant association between IRX4 methylation and CRC. Consequently, two fragments from the IRX4 promoter region were selected for pyrosequencing-based methylation analysis (**Figure 3A**). The methylation levels of each CpG site across the samples were displayed using a bee swarm plot, and intergroup differences were assessed (**Figure 3B**). The methylation percentage for each CpG site was also analyzed, showing that the average methylation levels of fragments 1 and 2 in CRC tissues were significantly higher than those in adjacent normal tissues (*P* = 0.0003 and *P* = 0.001, respectively) (**Figure 3C**).

### Correlation of IRX4 with methylated genes in CRC and differences in methylation levels

**Figure 4:**
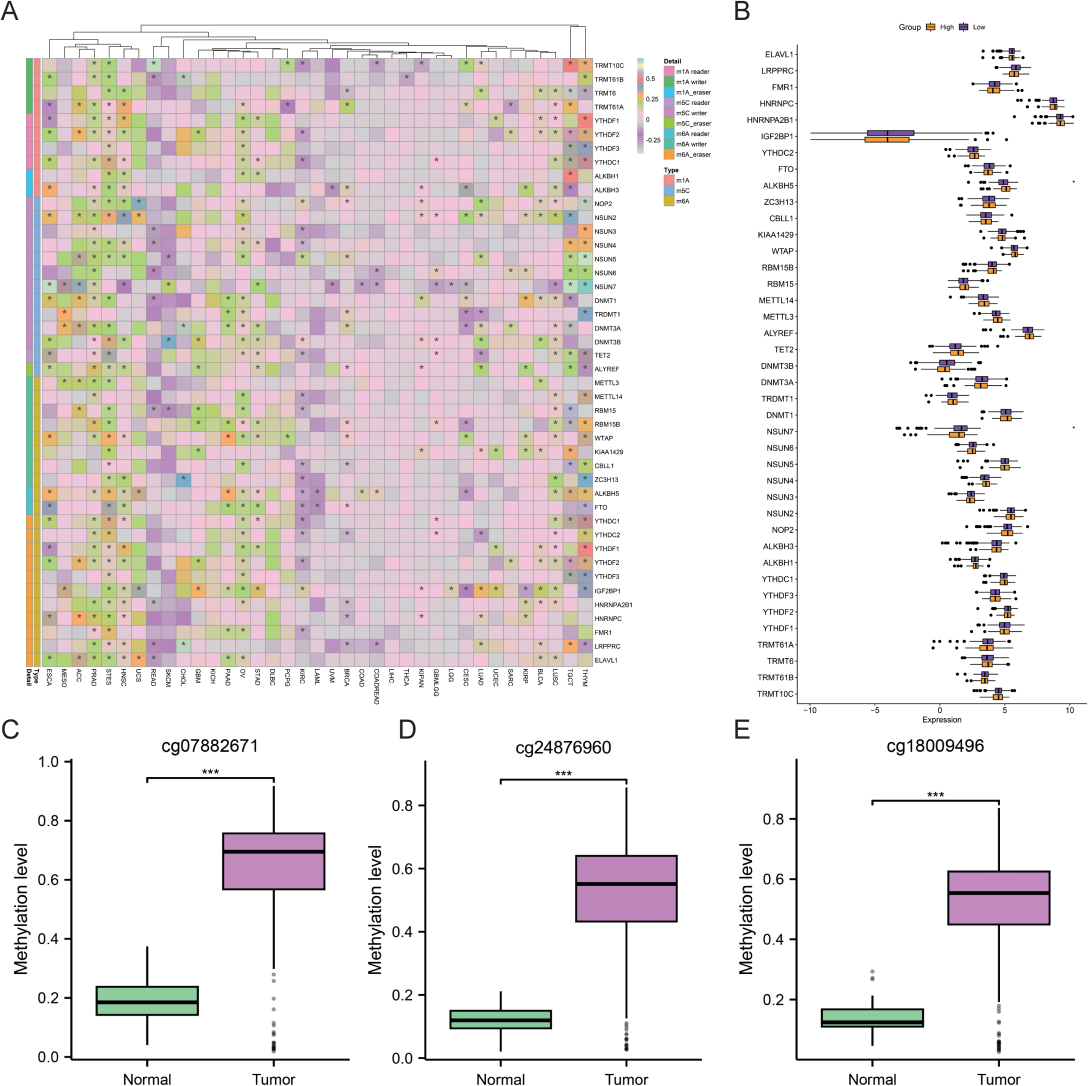
Correlation of IRX4 with m1A/m5C/m6A Methylation-Related Genes and Methylation Site Level Differences in CRC. **(A)** Correlation heatmap illustrating the relationship between IRX4 expression and the expression of m1A/m5C/m6A-related genes in pan-cancer. **(B)** Boxplot showing expression differences of m1A/m5C/m6A-related genes across groups based on median IRX4 expression. **(C)** Boxplot depicting methylation level differences of cg07882671, **(D)** cg24876960, and **(E)** cg18009496 between normal and CRC groups. ***P < 0.001. The symbol * indicates statistical significance (P<0.05).

To explore the potential role of IRX4 methylation in CRC, a correlation analysis was conducted. Results revealed that, in pan-cancer, IRX4 was closely associated with several m1A/m5C/m6A methylation-related genes, such as NOP2, DNMT3B, and IGF2BP1 (**Figure 4A**). Further analysis of the relationship between IRX4 expression and m1A/m5C/m6A methylation-related genes in CRC revealed potential associations with genes like ALKBH5 and NSUN7 (**Figure 4B**). Additionally, the methylation levels of IRX4 were significantly higher in CRC tissues compared to normal tissues, with the most pronounced differences observed at three sites: cg07882671 (**Figure 4C**), cg24876960 (**Figure 4D**), and cg18009496 (**Figure 4E**) (*P* < 0.001). These results are consistent with the previous pyrosequencing-based methylation analysis.

### IRX4-related signaling pathways

**Figure 5:**
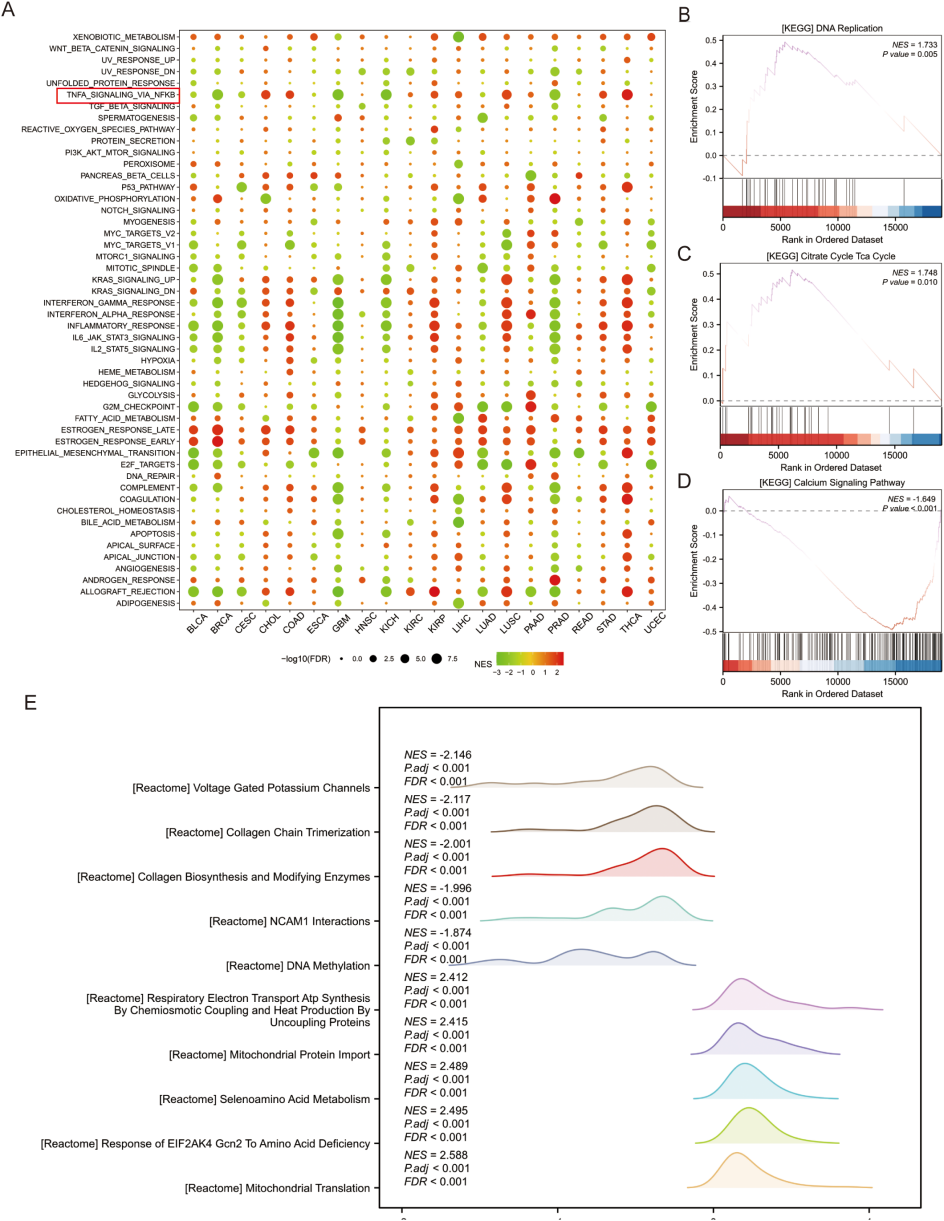
IRX4 is Associated with Multiple Cancer and Metabolic Signaling Pathways. **(A)** Pan-cancer GSEA based on IRX4 expression differential results grouped by median values and the HALL-MARK dataset, showing potential associations with key signaling pathways. **(B)** GSEA based on the KEGG dataset displaying the DNA replication pathway. **(C)** GSEA based on the KEGG dataset displaying the citrate cycle (TCA cycle). **(D)** GSEA based on the KEGG dataset displaying the calcium signaling pathway. **(E)** Ridge plot displaying the GSEA results from the REACTOME gene set. Each row corresponds to a distinct Reactome pathway. NES (Normalized Enrichment Score): Quantifies pathway enrichment magnitude. Negative values indicate the enrichment of downregulated genes, while positive values indicate the enrichment of upregulated genes. (FDR < 0.001, adjusted *P* < 0.001).

GSEA enrichment analyze was conducted to investigate signaling pathway with significant regulatory effects closely associated with IRX4. Pan-cancer GSEA revealed that IRX4 downregulation is strongly associated with the activation of key signaling pathways, including NF-κB and JAK-STAT3 (**Figure 5A**). GSEA of IRX4-associated DEGs in CRC identified significant links with pathways like DNA replication (**Figure 5B**), the citrate cycle (TCA cycle) (**Figure 5C**), and calcium signaling (**Figure 5D**). Additionally, REACTOME analysis highlighted the prominent association of IRX4 with pathways such as voltage-gated potassium channels, DNA methylation, and mitochondrial translation (**Figure 5E**). These results provide valuable insights into the potential roles and mechanistic pathways through which IRX4 may influence cancer progression.

### IRX4 is predominantly expressed in the nucleus and its expression level is significantly downregulated in CRC

**Figure 6:**
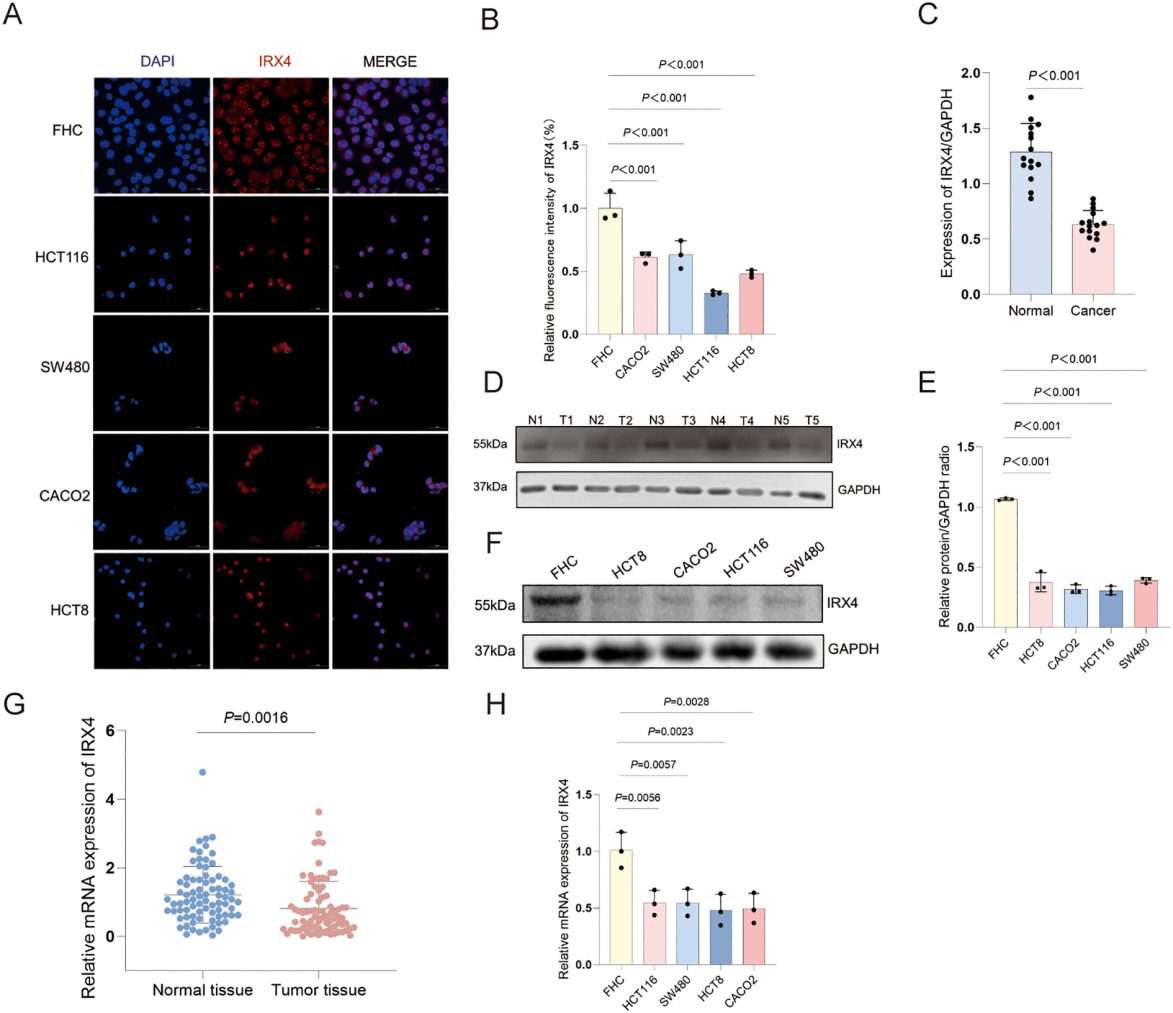
IRX4 Expression is Significantly Downregulated in CRC. **(A, B)** Immunofluorescence staining of IRX4 (red) and the cell nucleus (blue), showing that IRX4 is primarily localized in the nucleus, with significantly reduced average fluorescence intensity in CRC cells compared to normal intestinal epithelial cells. Scale bar: 50 μm. **(C, D)** Protein expression of IRX4 in CRC tissues and adjacent normal tissues with statistical analysis. **(E, F)** Proin expression of IRX4 in CRC cells and normal intestinal epithelial cells. **(G)** RT-qPCR analysis of IRX4 expression in CRC tissues and corresponding adjacent normal tissues. **(H)** mRNA expression of IRX4 in four CRC cell lines compared to FHC cells. Data are presented as the mean ± SEM (n = 3). *P* < 0.05 is considered the difference to be statistically significant.

In this study, 80 human CRC tissue samples and corresponding adjacent normal tissues were collected, along with a normal intestinal epithelial cell line (FHC) and four CRC cell lines (HCT116, SW480, HCT8, and Caco2). RT-qPCR and WB analyses were performed to assess the mRNA and protein expression levels of IRX4 in both CRC tissues and cell lines. The results demonstrated a significant downregulation of IRX4 mRNA and protein expression in CRC tissues compared to adjacent normal tissues (*P* = 0.0016 and *P* < 0.001, respectively) (**Figures 6C, D, G**). Similarly, IRX4 expression was significantly lower in the four CRC cell lines compared to FHC cells at both mRNA and protein levels (*P* < 0.01 and *P* < 0.001, respectively) (**Figures 6E, F, H**). IF staining revealed that IRX4 was predominantly localized in the nucleus in both FHC and CRC cell lines. Semi-quantitative IF analysis confirmed that IRX4 protein expression was markedly reduced in CRC cells (*P* < 0.001) (**Figures 6A, B**). The expression pattern of IRX4 in CRC tissues and cells was consistent with the methylation chip sequencing findings.

### Low IRX4 Expression predicts poor prognosis and is negatively correlated with tumor invasion depth (T Stage) in CRC

**Figure 7:**
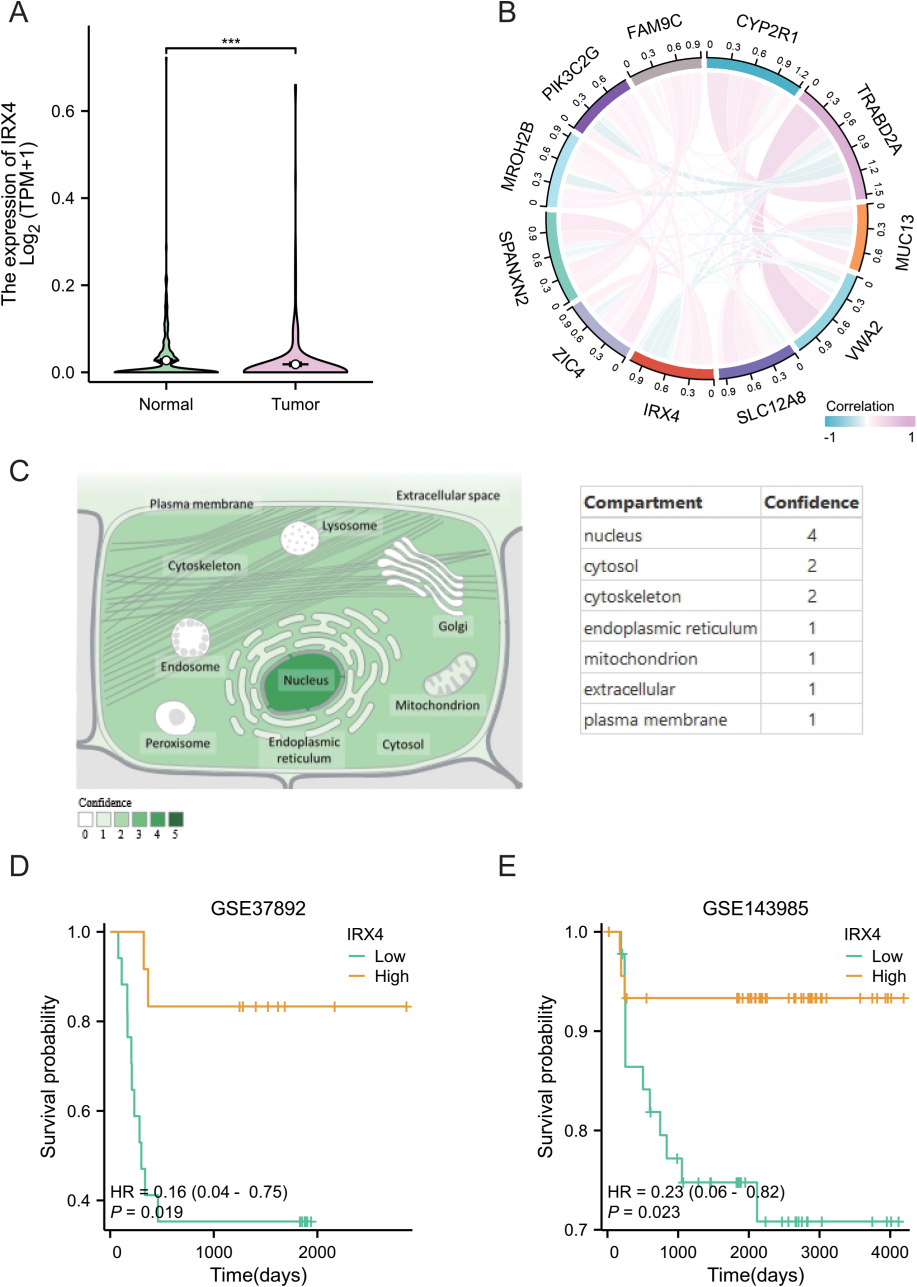
Low Expression of IRX4 in CRC and Its Association with Poor Prognosis. **(A)** Expression levels of IRX4 in normal and CRC tissues. **(B)** Correlation diagram highlighting genes closely associated with IRX4 expression in CRC. **(C)** Subcellular localization of IRX4 as shown by Genecard. **(D)** Impact of IRX4 expression on recurrence-free survival in patients with CRC from the GSE37892 dataset. **(E)** Impact of IRX4 expression on recurrence-free survival in patients with CRC from the GSE143985 dataset. Data are presented as the mean ± SEM (n = 3). ***P < 0.001.

To further explore the role of IRX4 in CRC, IRX4 expression was compared between normal and CRC tissues. The results indicated a significant downregulation of IRX4 in CRC tissues (**Figure 7A**) (*P* < 0.001). Correlation analysis of genes associated with IRX4 expression revealed a strong association with genes such as SLC12A8, VWA2, and MUC13 (**Figure 7B**). Subcellular localization studies confirmed that IRX4 is predominantly confined to the nucleus (**Figure 7C**). Furthermore, survival analysis demonstrated that low IRX4 expression correlates with shorter recurrence-free survival and poorer prognosis in patients with CRC (**Figures 7D, E**).

Based on the IRX4 expression levels in CRC, the 80 clinical samples were categorized into low and high expression groups. Chi-square tests and univariate analysis were conducted using SPSS 29.0 software. The results indicated that in CRC tissues, IRX4 expression did not exhibit significant associations with tumor location, gender, age, vascular invasion, neural invasion, lymph node metastasis, HER2 status, KRAS/NRAS mutations, or tumor markers (CEA, CA199, and CA724) (*P* > 0.05). For HER2(2+) patients, FISH testing was performed to confirm the gene status. However, IRX4 expression was significantly correlated with tumor invasion depth (*P* = 0.025) (**Table 1**). Binary logistic regression analysis, using IRX4 expression as the independent variable and tumor invasion depth (shallow invasion: T1-T2; deep invasion: T3-T4) as the dependent variable, revealed that IRX4 expression negatively influenced tumor invasion depth (*P* = 0.028) (**Table 2**).

**Table 1:** Relationship between IRX4 expression and clinicopathological parameters.

Groups	IRX4(%)		χ2	*P*
	Low expression group	High expression group		
Gender				
Female	23(41.07)	13(54.17)	1.164	0.281
Male	33(58.93)	11(45.83)		
Age				
≤60 years	19(33.93)	7(29.17)	0.174	0.677
>60 years	37(66.07)	17(70.83)		
Tumor Location				
Rectum	26(46.43)	14(58.33)	0.952	0.329
Colon	30(53.57)	10(41.67)		
Depth of Invasion				
T1-T2	22(39.29)	16(66.67)	5.051	0.025*
T3-T4	34(60.71)	8(33.33)		
Vascular Emboli				
Yes	9(16.07)	6(25.00)	0.879	0.348
No	47(83.93)	18(75.00)		
Nerve Invasion				
Yes	16(28.57)	4(16.67)	1.27	0.26
No	40(71.43)	20(83.33)		
Lymph Node Metastasis				
Yes	29(51.79)	7(29.17)	3.473	0.062
No	27(48.21)	17(70.83)		
CEA				
≤5.0	38(67.86)	19(79.17)	1.049	0.306
>5.0	18(32.14)	5(20.83)		
CA199				
≤30.0	45(80.36)	23(95.83)	3.156	0.076
>30.0	11(19.64)	1(4.17)		
CA724				
≤6.9	43(76.79)	22(91.67)	2.442	0.118
>6.9	13(23.21)	2(8.33)		
KRAS, NRAS				
Wild-type	30(53.57)	9(37.50)	1.737	0.188
Mutant	26(46.43)	15(62.50)		
HER-2				
0	26(46.43)	9(37.50)	1.479	0.687
1+	22(39.29)	9(37.50)		
2+,FISH(-)	7(12.50)	5(20.83)		
3+	1(1.79)	1(4.17)		

* :*P* < 0.05.

**Table 2:** Binary logistic regression analysis results.

	Regression coefficient	Standard error	z-value	Wald *χ2*	P-value	ORValue	95% CI for OR value
IRX4	-1.128	0.512	-2.203	4.854	0.028	0.324	0.119 ~ 0.883
Intercept	0.435	0.274	1.591	2.531	0.112	1.545	0.904 ~ 2.642

[Note: Dependent variable = Depth of Invasion(shallow invasion: T1-T2; deep invasion: T3-T4)].

### IRX4 is effectively overexpressed in plasmid-transfected CRC cells

**Figure 8:**
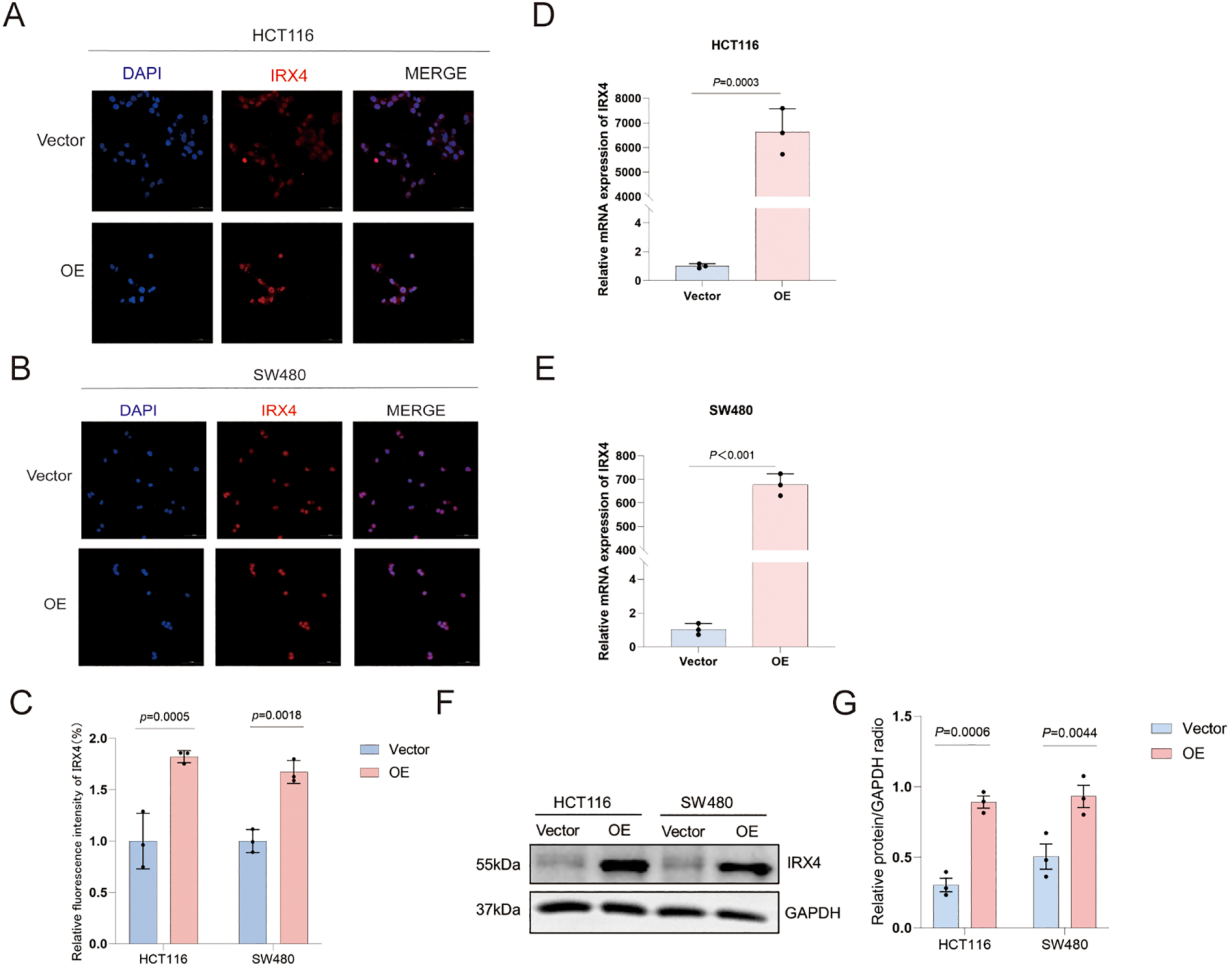
IRX4 is Effectively Overexpressed in Plasmid-Transfected CRC Cells. **(A, B)** mRNA expression of IRX4 in HCT116 and SW480 cells following plasmid transfection. **(C–E)** IF results and semi-quantitative analysis of IRX4 expression in HCT116 and SW480 cells post-transfection. **(F, G)** IRX4 protein expression levels in HCT116 and SW480 cells after plasmid transfection with statistical analysis. Vector: empty vector transfection group; OE: over-expression plasmid transfection group. Data are presented as the mean ± SEM (n=3). *P* < 0.05 is considered the difference to be statistically significant.

To further investigate IRX4’s functional role, HCT116 and SW480 cells were transfected with either IRX4 empty vector (control group - Vector) or overexpression plasmids (experimental group - OE). RT-qPCR, WB, and IF experiments were performed to confirm transfection efficiency. The results showed that, compared to the control group, IRX4 mRNA expression was significantly upregulated in CRC cells transfected with the overexpression plasmid (*P =* 0.0003 and *P* < 0.001, respectively) (**Figures 8A, B**). Moreover, IRX4 fluorescence intensity was significantly increased (*P =* 0.0005 and *P =* 0.0018, respectively) (**Figures 8C–E**), and total protein levels were substantially elevated (*P =* 0.0006 and *P =* 0.0044, respectively) (**Figures 8F, G**). Overall, effective overexpression of IRX4 was achieved in both HCT116 and SW480 cell lines, with a more pronounced effect observed in the HCT116 cells.

### IRX4 overexpression inhibits CRC cell biological behaviors

To further investigate the impact of IRX4 on the biological functions of CRC cells, IRX4 overexpression was induced in HCT116 and SW480 cells via efficient transfection of the PcDNA3.1 plasmid carrying the IRX4 gene. Transfection efficiency was validated by RT-qPCR, WB, and IF (**Figure 8**). The OD was measured at 450 nm, and the 50% maximum inhibitory concentration (IC50) of the OXA was calculated from the OD value (**Figures 1A, B**).The CCK-8 assay revealed a reduction in the cell viability of CRC cells upon IRX4 overexpression (*P* < 0.001) (**Figures 1C, D**). However, the effect of IRX4 overexpression on cell viability was is still insufficient (only 12% difference at 72 hours), despite being significant. EdU assay showed that overexpression of IRX4 significantly inhibited CRC cell proliferation (*P =* 0.003 and *P* < 0.001, respectively) (**Figures 1E–G**). In the scratch assay, migration rates were evaluated at 24 and 48 hours, with IRX4 overexpression significantly decreasing cell migration in both HCT116 and SW480 cells (*P* < 0.001) (**Figures 1H–K**). Similarly, the Transwell invasion assay demonstrated that IRX4 overexpression markedly suppressed the invasive potential of CRC cells (*P* < 0.001 and *P* = 0.004, respectively) (**Figures 1L, M**). Furthermore, Annexin V/7-AAD staining and flow cytometry analysis revealed a significant increase in apoptosis in IRX4-overexpressing CRC cells (*P* < 0.001) (**Figures 1N, O**). The results from three independent experiments consistently demonstrated significant differences in cell proliferation, migration, invasion, and apoptosis, emphasizing the pivotal role of IRX4 in regulating CRC cell biological functions.

### Overexpression of IRX4 enhances the cytotoxicity of OXA on CRC cells

**Figure 9:**
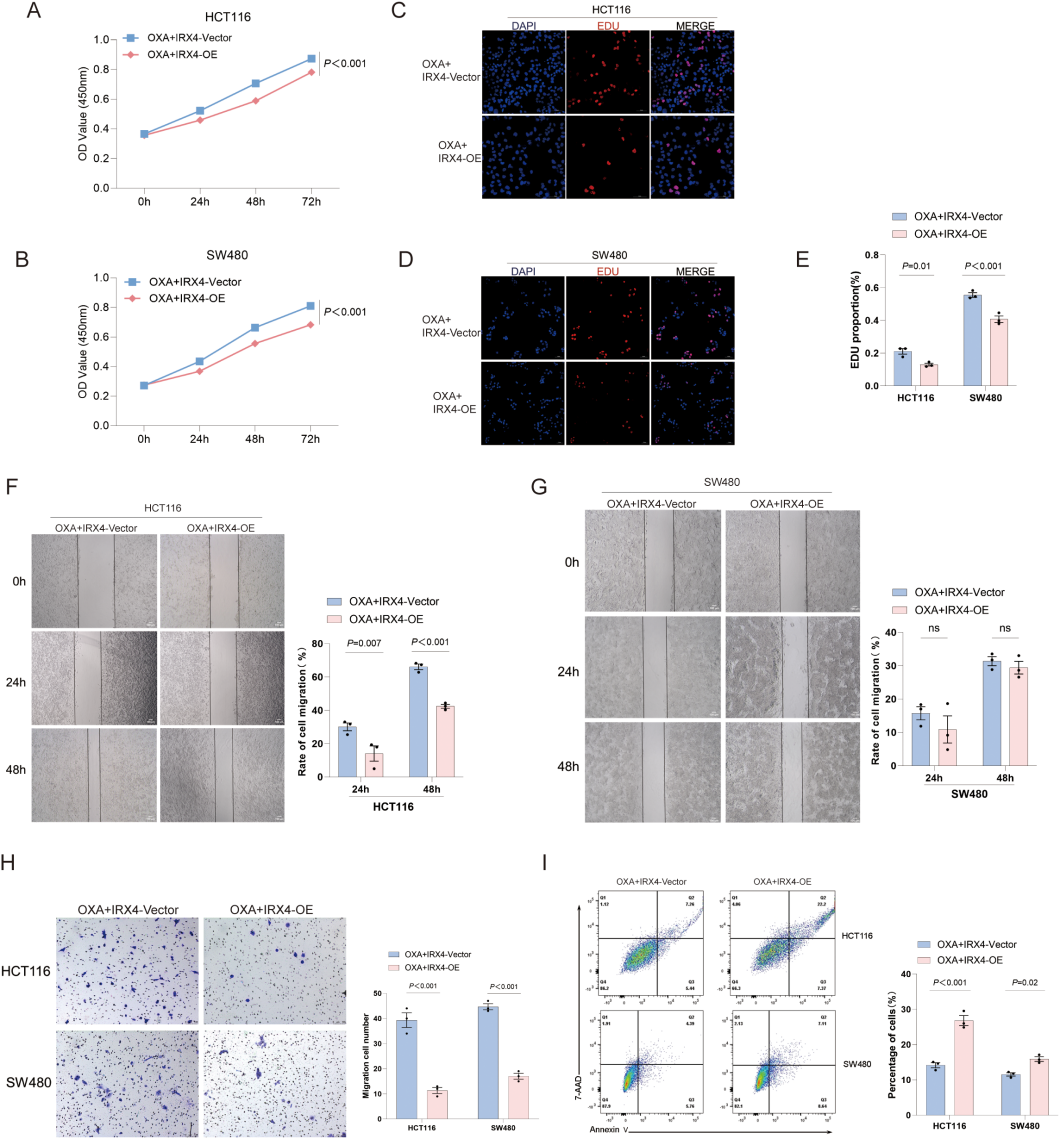
IRX4 Overexpression Enhances CRC Cell Sensitivity to OXA Chemotherapy. **(A, B)** CCK-8 proliferation assay results for HCT116 and SW480 cells. **(C-E)** EDU proliferation assay results. **(F, G)** Scratch assay results. **(H)** Transwell invasion assay results. **(I)** Flow cytometry analysis of cell apoptosis using Annexin V and 7-AAD dual staining. Vector: Empty plasmid transfection group; OE: Overexpression plasmid transfection group. Data are presented as the mean ± SEM (n = 3). *P* < 0.05 is considered the difference to be statistically significant, ns: No statistical significance.

To explore the impact of IRX4 on OXA chemotherapy sensitivity, IRX4 overexpression was induced in HCT116 and SW480 cells via plasmid transfection. CCK-8 assays revealed that IRX4 overexpression, in combination with OXA, more effectively suppressed cell viability in both HCT116 and SW480 cells compared to OXA treatment alone (*P* < 0.001) (**Figures 9A, B**). EDU assays corroborated these observations (*P* = 0.01 and *P* < 0.001, respectively) (**Figures 9C–E**), suggesting that IRX4 overexpression enhances the inhibitory effect of OXA on cell proliferation. In the scratch assay, a marked reduction in the migration rate of HCT116 cells was observed with IRX4 overexpression combined with OXA treatment (*P* = 0.007 and *P* < 0.001, respectively) (**Figure 9F**). Although a trend toward decreased migration was noted in SW480 cells, this result did not reach statistical significance (**Figure 9G**) (ns), likely due to the small sample size. Further investigation with a larger sample size and more robust experimental design is needed to confirm this trend. Similarly, the Transwell invasion assay revealed a significant decrease in invasion rate in CRC cells treated with OXA and overexpressing IRX4 (*P* < 0.001) (**Figure 9H**). Additionally, Annexin V/7-AAD dual staining showed that IRX4 overexpression, in conjunction with OXA, promoted apoptosis in CRC cells (*P* < 0.001 and *P* = 0.02, respectively) (**Figure 9I**). The above results suggested that IRX4 overexpression might enhance the antitumor effect of OXA on CRC cells, thus improving the therapeutic effect.

### OXA resistance is associated with EGFR overactivation

**Figure 10:**
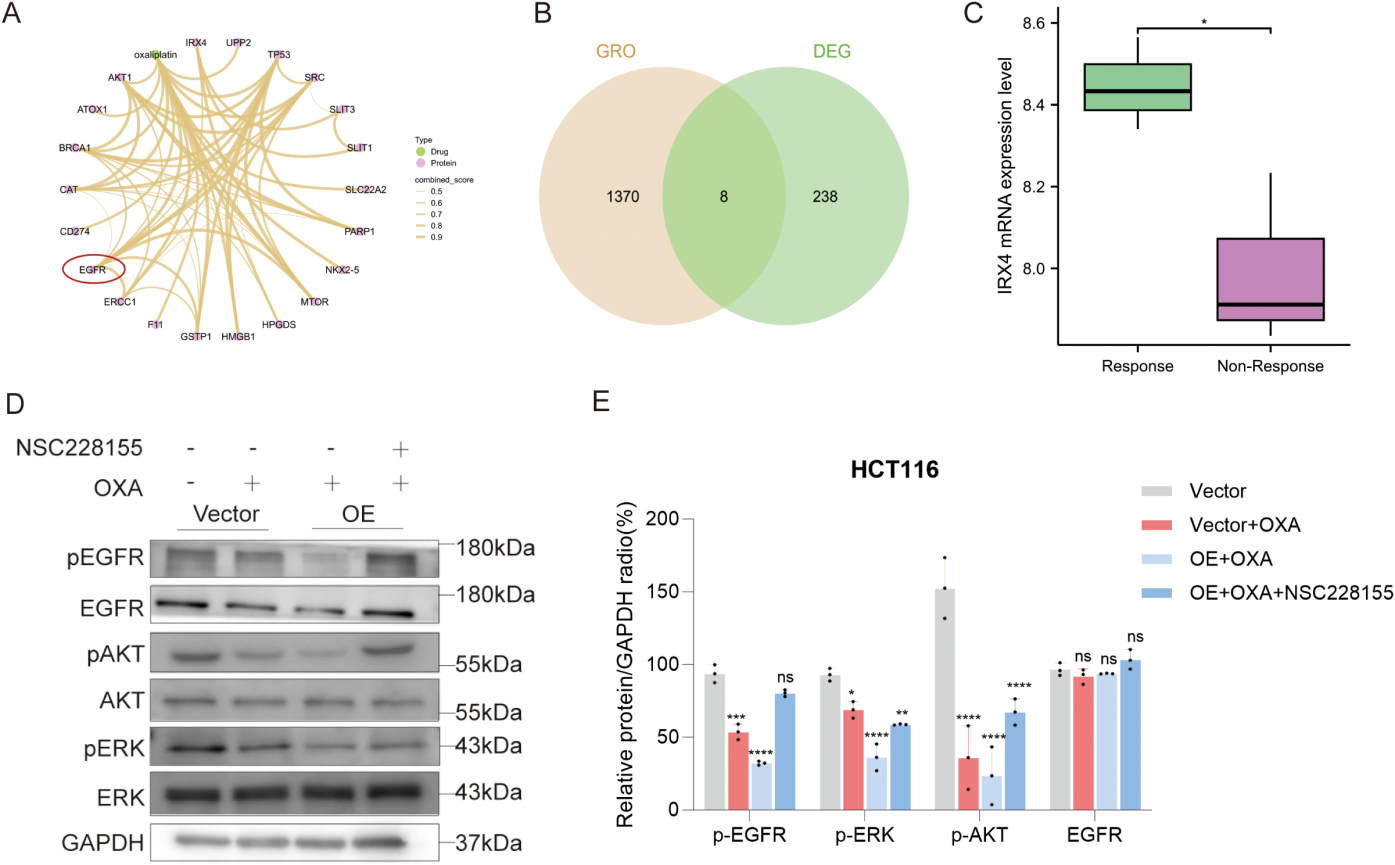
Association between OXA sensitivity and EGFR overactivation in CRC. **(A)** Interaction network of OXA, IRX4, and EGFR signaling. **(B)** Venn diagram showing the overlap between genes related to OXA and DEGs classified by IRX4 median expression. **(C)** IRX4 expression levels in responders and non-responders in OXA-treated CRC patients. **(D, E)** Protein levels of EGFR, p-EGFR (Tyr1197), AKT, p-AKT (Ser473), ERK and p-ERK (Thr202/Tyr204) were detected in HCT116 cells by Western blot. GAPDH is used as an internal reference, and GAPDH was used as internal reference. Data are presented as the mean ± SEM (n = 3). *P < 0.05, **P < 0.01, ***P < 0.001, and ns denoted non-significant. *P* < 0.05 is considered the difference to be statistically significant.

To explore the clinical relevance of IRX4 as a biomarker, we utilized the GEO dataset to analyze differences in IRX4 expression between oxaliplatin-responsive (R) and non-responsive (NR) groups. Our results revealed that the NR group had significantly lower IRX4 expression levels compared to the R group(*P* < 0.05) (**Figure 10C**). These findings suggest that IRX4 expression is closely associated with OXA treatment efficacy. We further explored potential associations among OXA, EGFR, and IRX4 in CRC using the STITCH database. These analyses indicated that IRX4 may regulate OXA efficacy via the EGFR signaling pathway (**Figure 10A**). To identify functional links between IRX4 and OXA, we screened OXA-related genes from the GeneCards database and performed intersection analysis with IRX4-associated differentially expressed genes. This yielded eight co-regulated genes (CSDM1, MYH1, SCN2A, NTF4, PIWIL3, DNTT, PRDM14, and MIF) that functionally connect IRX4 to OXA (**Figure 10B**) and may modulate OXA responses through interactions with IRX4. It is well established that activation of the EGFR signaling pathway contributes to resistance of tumors to OXA (22, 23). To further elucidate the roles of IRX4 and EGFR in chemosensitivity in CRC, we conducted *in vitro* experiments using the HCT116 cell line. Western blot analysis demonstrated that the combination of OXA and IRX4 overexpression significantly reduced the phosphorylation levels of phosphorylated EGFR (p-EGFR) and its key downstream effectors, phosphorylated AKT (p-AKT) and phosphorylated ERK (p-ERK), compared to OXA treatment alone. Treatment of HCT116 cells with the EGFR agonist NSC228155 following IRX4 overexpression and OXA administration partially restored the phosphorylation of p-EGFR, p-AKT, and p-ERK in HCT116 cells (**Figures 10D, E**). These findings suggest that IRX4 may mitigate OXA resistance in CRC by inhibiting the activation of EGFR signaling pathways and suppressing the activation of two major downstream signaling pathways, PI3K/Akt and MAPK/ERK.

### IRX4 inhibits NF-κB pathway

Enrichment analysis revealed that high IRX4 expression in tumor tissues was closely associated with the NF-κB signaling pathway. To evaluate the impact of IRX4 on NF-κB signaling, we assessed NF-κB transcriptional activity using a luciferase reporter assay. Our results demonstrated that IRX4 overexpression significantly attenuated TNF-α-induced NF-κB luciferase activity (*P* = 0.0001) (**Supplementary Figure 1D**). Additionally, IF staining showed that IRX4 overexpression inhibited the nuclear translocation of the NF-κB/p65 protein in HT29 cells (**Supplementary Figure 1A**). Subsequently, we performed quantitative analysis of p65 in the cytoplasm and nucleus and IκBα expression by Western blot. The results demonstrated that after IRX4 overexpression, the nuclear protein level of p65 was significantly decreased, whereas no significant change was observed in IκBα (**Supplementary Figures 1B, C, E**). This indicates that IRX4-mediated activation of p65 is independent of IκBα. Collectively, these findings suggest a close association between IRX4 and the NF-κB signaling pathway.

## Discussion

This study, integrating methylation chip data with bioinformatics analysis and pyrosequencing technology, identifies IRX4 as a core gene and systematically investigates its clinical relevance in CRC. Initial assessment of IRX4 expression in CRC tissues and cells, alongside an analysis of its correlation with clinical pathological features and patient prognosis, revealed that low IRX4 expression correlates with shorter disease-free survival and poorer prognosis. *In vitro* experiments showed that the overexpression of IRX4 inhibited CRC cells proliferation, migration, and invasion and promoted apoptosis. It’s worth noting that the regulatory effect of IRX4 on the migration and invasion abilities of CRC cells is more prominent. Clinically, IRX4 is downregulated and negatively correlated with tumor invasiveness in CRC tissues. Similarly, following combined treatment with OXA and IRX4 overexpression, the inhibitory effect on CRC cell migration and invasion was more significant compared with its impact on cell viability. These findings that IRX4 may affect tumor progression by inhibiting the migration and invasion of tumor cells, and IRX4 has certain clinical potential value in enhancing the chemotherapy sensitivity of OXA.

IRX4 has been implicated in the development and progression of various cancers. Studies suggest that IRX4 may function as a tumor suppressor in PC, pancreatic cancer, and oral squamous cell carcinoma (OSCC). In PC, IRX4 is proposed as a potential risk gene and is thought to regulate gene expression through interaction with the vitamin D receptor, thereby inhibiting cancer cell proliferation and differentiation (18, 19). In pancreatic cancer, IRX4 gene methylation suppresses its expression, promoting cell proliferation, while its reactivation inhibits cancer cell growth (8). In OSCC, IRX4 expression is significantly lower in SCC-9 cells compared to OKF6-TERT1R cells, with this disparity potentially regulated by epigenetic mechanisms (24). Whole-genome DNA methylation analysis revealed increased methylation of IRX4 in SCC-9 cells compared to OKF6-TERT1R cells (25). Such epigenetic alterations in IRX4 may serve as a risk factor for OSCC (26), and its methylation status has been proposed as a marker for predicting radiotherapy efficacy in oropharyngeal cancer (27). In HPV-positive OSCC, hypermethylation of the IRX4 promoter region negatively correlates with its transcription levels and is significantly associated with improved survival outcomes in patients with HPV-positive OPSCC (28). These findings collectively suggest that IRX4 functions as a tumor suppressor gene in the initiation and progression of several cancers, with a significant association with methylation. In the present study, bioinformatics differential analysis and pyrosequencing confirmed that IRX4 methylation is closely linked to CRC development. Furthermore, *in vitro* experiments revealed that IRX4 overexpression significantly inhibited tumor-related biological behaviors of CRC cells. We observed that the effect of IRX4 on the proliferative capacity of CRC cells was significantly weaker than its effect on that of prostate cancer cells. Combining with the results of this study, we speculate that the core regulatory role of IRX4 in CRC does not rely on changes in cell viability, but rather modulates disease progression by inhibiting tumor cell migration and invasion.

In this study, GO, KEGG, and GSEA enrichment analyses were performed to explore the potential signaling pathways through which IRX4 may exert its tumor-suppressive effects in CRC. The findings suggest that IRX4’s mechanism in CRC is multifaceted, encompassing regulation of cell proliferation, metabolic pathways, epigenetic modifications, and signal transduction. Specifically, IRX4 appears to influence several key pathways, including NF-κB, JAK-STAT3, DNA replication, calcium signaling, and metabolic regulation, all of which may contribute to the inhibition of CRC initiation and progression. The NF-κB pathway is notably involved in inflammation, cell survival, proliferation, and metastasis, while the JAK-STAT3 pathway is essential for regulating cell proliferation, immune evasion, and drug resistance (29, 30). To assess the regulation of NF-κB signaling by IRX4, we examined the nuclear translocation of the NF-κB p65 subunit and NF-κB luciferase reporter activity. Our results showed that overexpression of IRX4 inhibited the nuclear translocation of p65 and NF-κB luciferase reporter activity. These results suggest that IRX4 may be an inhibitor of NF-κB in CRC cells.

IRX4 expression is closely linked to chemotherapy resistance. Previous studies have demonstrated that IRX4 can enhance chemotherapy resistance in tumor cells by modulating cancer stem cell-like properties. In NSCLC, silencing IRX4 expression increases the cytotoxic effects of gefitinib in PC-9/GR cells (9). Furthermore, a recent study showed that IRX4-PEP1 promotes docetaxel resistance in PC cells by regulating the Wnt signaling pathway and cancer stem cell traits (10). These findings suggest that targeting IRX4 may help overcome chemotherapy resistance. Unfortunately, the role of IRX4 in chemotherapy resistance in CRC has not been fully explored. Our study is the first to demonstrate that IRX4 overexpression enhances CRC cell sensitivity to OXA. IRX4 may enhance sensitivity to OXA by modulating the EGFR signaling pathway, as predicted using the STITCH database. This experiment confirmed that IRX4 could regulate EGFR signaling in CRC. Considering the significant inhibitory effect of IRX4 on the migration and invasion abilities of CRC cells, as well as the core regulatory position of the EGFR signaling pathway in tumor invasion, we speculate that IRX4 may play a key role in the migration and invasion of CRC cells by targeting the EGFR signaling pathway. This provides a key basis for the tumor suppressor mechanism of IRX4 in CRC.

This study provides novel insights into the role of IRX4 in CRC. However, certain limitations remain. First, this study was conducted only in HCT116 and SW480 cells, not in other CRC cell lines. Additionally, the number of clinical samples collected was limited. Furthermore, only overexpression experiments were performed, while loss-of-function experiments for IRX4 are lacking. Future studies should therefore be conducted in additional CRC cell lines or using animal models. Second, since this study focused on exploring the downstream activation mechanism of IRX4 in CRC and did not quantify IRX4 methylation status in *in vitro* cell models, future studies will further quantify IRX4 methylation status in *in vitro* models and correlate it with IRX4 expression levels and OXA sensitivity. Lastly, this study focused solely on phenotypic-level analyses. While the functional role of IRX4 in CRC cells was preliminarily validated through assays for cell proliferation, migration, invasion, and apoptosis, the underlying molecular mechanisms and specific signaling pathways regulated by IRX4 remain unexplored. Although enrichment analyses suggest potential associations between IRX4 and several critical pathways, these findings are still hypothetical and require further experimental validation. Future studies should address these limitations and provide deeper insights into the molecular role of IRX4 in CRC, along with its potential clinical applications.

Future research should examine the role of IRX4 in CRC and other malignancies from various perspectives, including clinical validation, signaling pathway interactions, combination therapies, and epigenetic regulation. Tumor cells actively secrete exosomes into the surrounding microenvironment, which not only facilitate intercellular communication among cancer cells but also influence cell-cell interactions within the tumor microenvironment (31). A comprehensive investigation of this mechanism will deepen our understanding of the multifaceted roles of IRX4 in tumor initiation, progression, and therapeutic responses, thereby offering a more solid theoretical foundation and practical support for clinical applications.

## Data Availability

The datasets presented in this study can be found in online repositories. The names of the repository/repositories and accession number(s) can be found in the article/**Supplementary Material**.
