# Supplementary material for: Anti-colorectal cancer effects of IRX4 and sensitivity studies to oxaliplatin
Source: Front Immunol. 2026 Jan 21;16:1581244. doi: 10.3389/fimmu.2025.1581244 (PMC12867854; doi:10.3389/fimmu.2025.1581244)
Supplement: Supplementary file 1 [file DataSheet1.pdf]

Bullet Edits

EDITORIAL CERTIFICATE

This document certifies that the paper listed below was edited and proofread for proper English language, grammar, punctuation, spelling, and overall style by one or more than one highly qualified native speakers at Bullet Edits. All of the suggested amendments were tracked with the Microsoft Word "Track Changes" feature. Therefore, the author had the option to reject or accept each change individually.

Manuscript Title:

Anti-colorectal cancer effects and sensitivity studies to oxaliplatin

Anti-counterfeiting Code:

e1927f754f632bb40f1f581472a21a12

Date Issued:

2025-01-09 09:59:02

Bullet Edits is a registered company headquartered in the UK with a global presence. We offer a range of editing, proofreading services to authors. Our Ph.D. editors are all native English speakers from the USA and UK. Authors who work with Bullet Edits are guaranteed excellent language quality and timely delivery.

Address: BULLET EDITS LIMITED, 85 Great Portland Street, London,UK
Tel: UK. (+44)20457 70286 / US.(+1)312-313-9179
Web: www.bulletedits.cn/Email: info@bulletedits.cn
VAT: GB 378 9316 43

BULLET EDITS LIMITED ★ COMPANY NO. 13345261 SEAL ★ ENGLAND AND WALES
