# Supplementary material for: Anti-colorectal cancer effects of IRX4 and sensitivity studies to oxaliplatin
Source: Front Immunol. 2026 Jan 21;16:1581244. doi: 10.3389/fimmu.2025.1581244 (PMC12867854; doi:10.3389/fimmu.2025.1581244)

CpG Pyrogram Report Run: 20240801 A

Well: C9
Assay: -1S
Sample ID: 17
Sequence Before Bisulfite Treatment: -
Sequence to analyze: GYGGGYGYGGTTYGGGGYGGAYGGGYGGGGTTTGTAGGGTTTTG

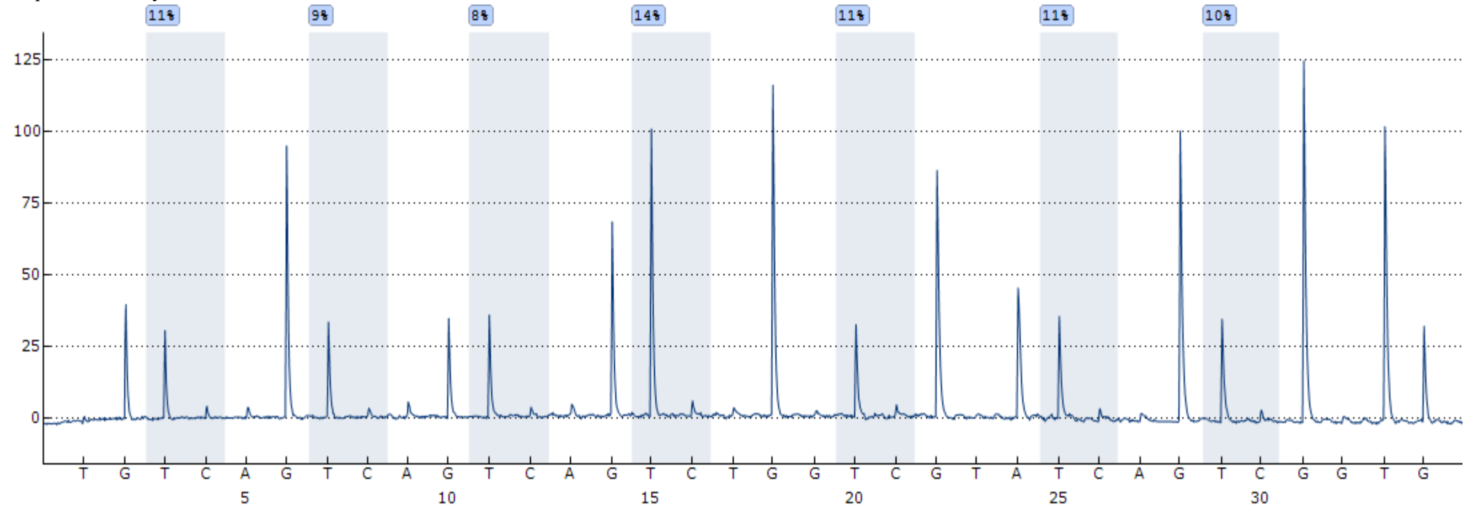

Well: C10
Assay: -1S
Sample ID: 18
Sequence Before Bisulfite Treatment: -
Sequence to analyze: GYGGGYGYGGTTYGGGGYGGAYGGGYGGGGTTTGTAGGGTTTTG

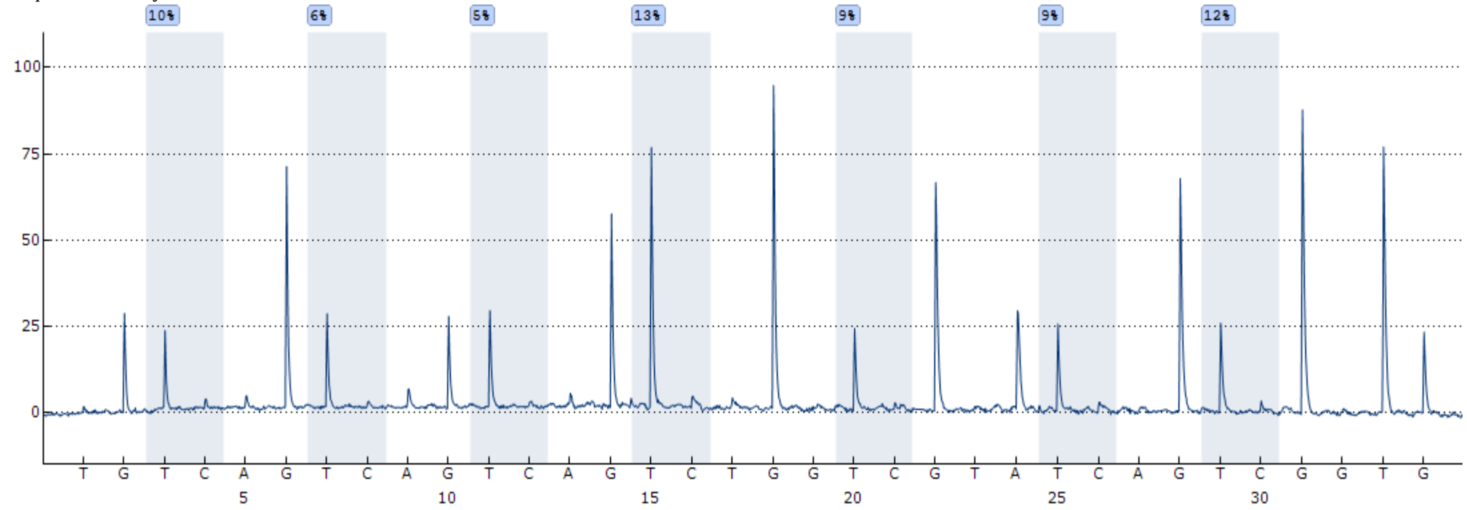

Well: C11
Assay: -1S
Sample ID: 19
Sequence Before Bisulfite Treatment: -
Sequence to analyze: GYGGGYGYGGTTYGGGGYGGAYGGGYGGGGTTTGTAGGGTTTTG

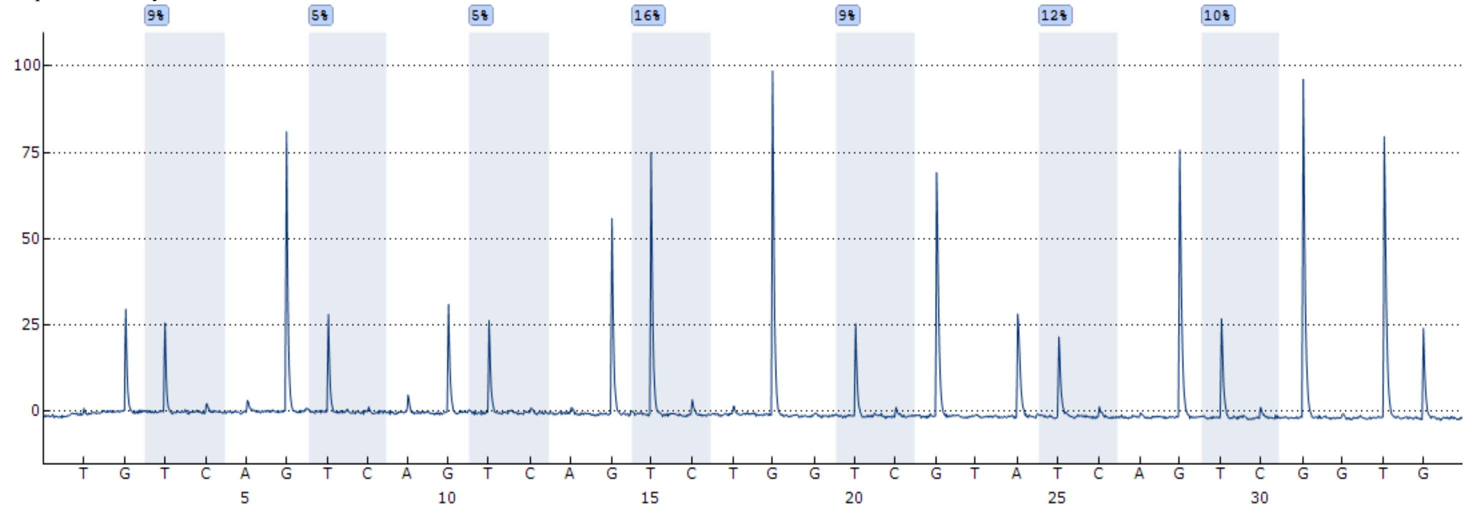

CpG Pyrogram Report Run: 20240801 A

Well: C12
Assay: -1S
Sample ID: 20
Sequence Before Bisulfite Treatment: -
Sequence to analyze: GYGGGYGYGGTTYGGGGYGGAYGGGYGGGGTTTGTAGGGTTTTG

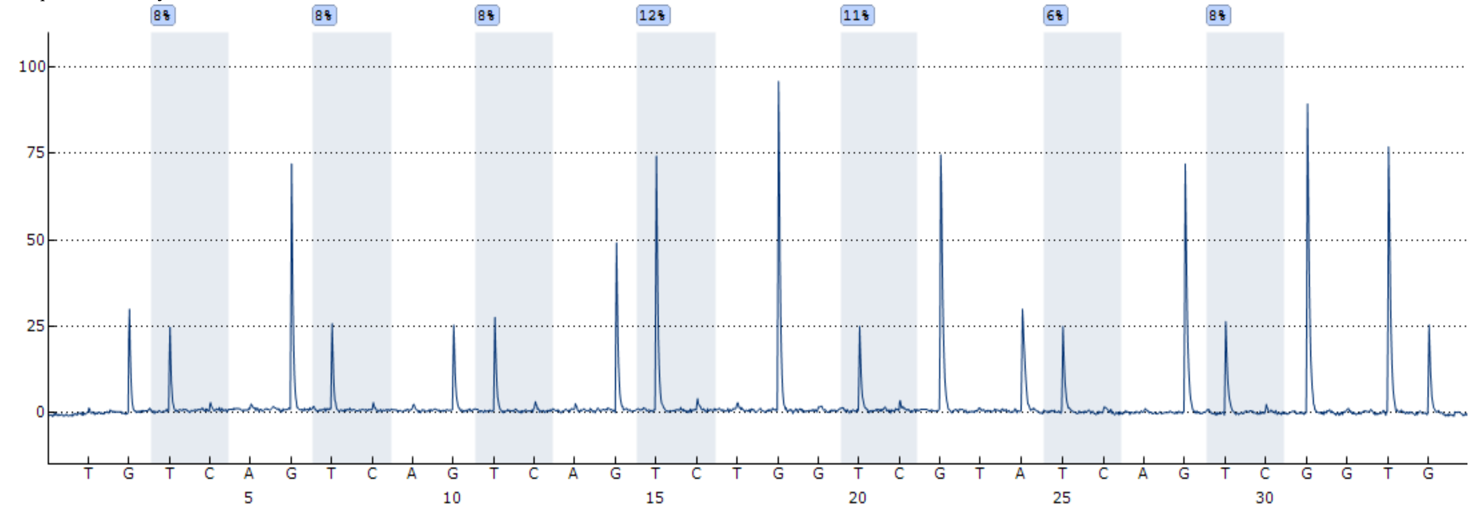

Well: D1
Assay: -1S
Sample ID: 21
Sequence Before Bisulfite Treatment: -
Sequence to analyze: GYGGGYGYGGTTYGGGGYGGAYGGGYGGGGTTTGTAGGGTTTTG

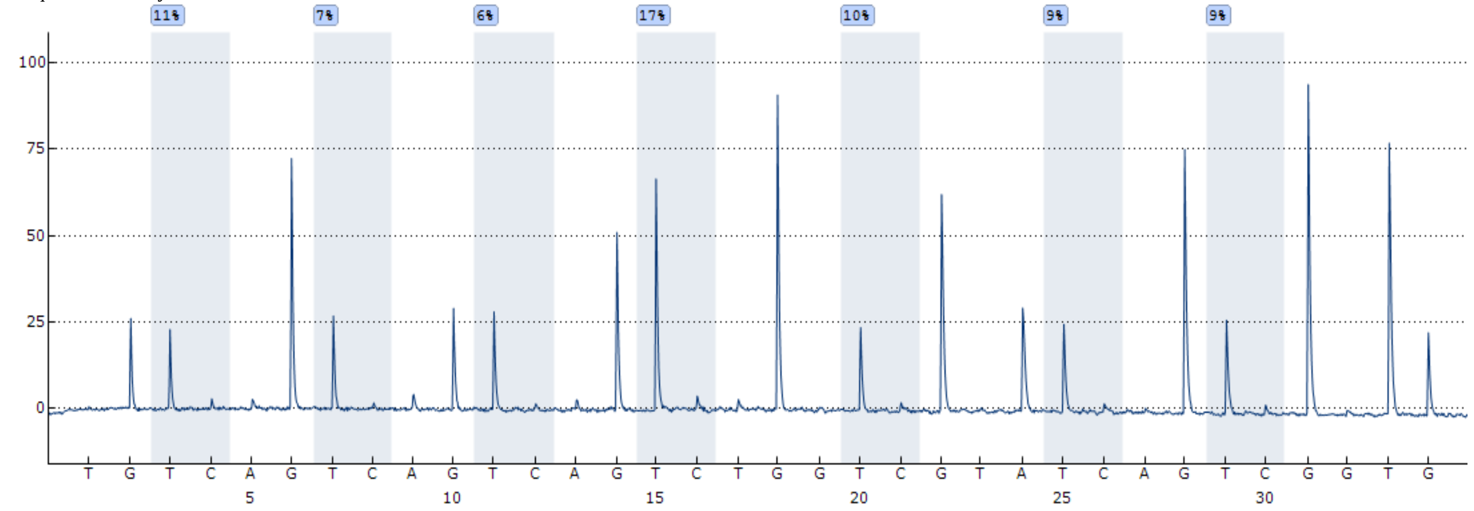

Well: D2
Assay: -1S
Sample ID: 22
Sequence Before Bisulfite Treatment: -
Sequence to analyze: GYGGGYGYGGTTYGGGGYGGAYGGGYGGGGTTTGTAGGGTTTTG

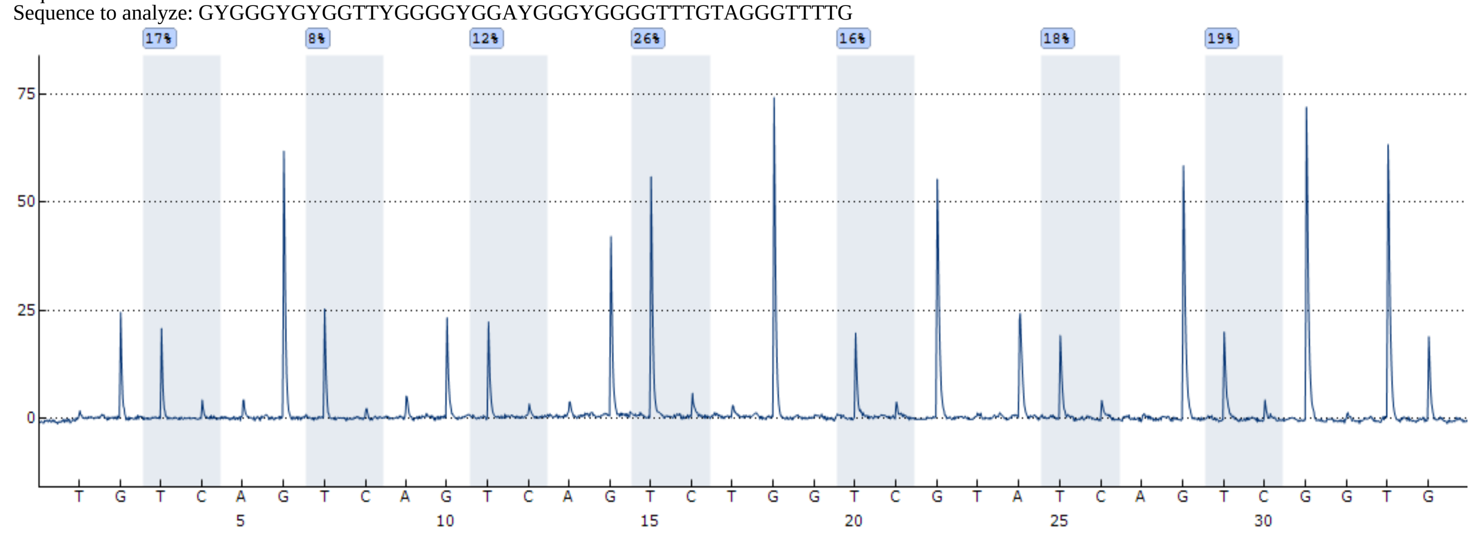

CpG Pyrogram Report Run: 20240801 A

Well: D3
Assay: -1S
Sample ID: 23
Sequence Before Bisulfite Treatment: -
Sequence to analyze: GYGGGYGYGGTTYGGGGYGGAYGGGYGGGGTTTGTAGGGTTTTG

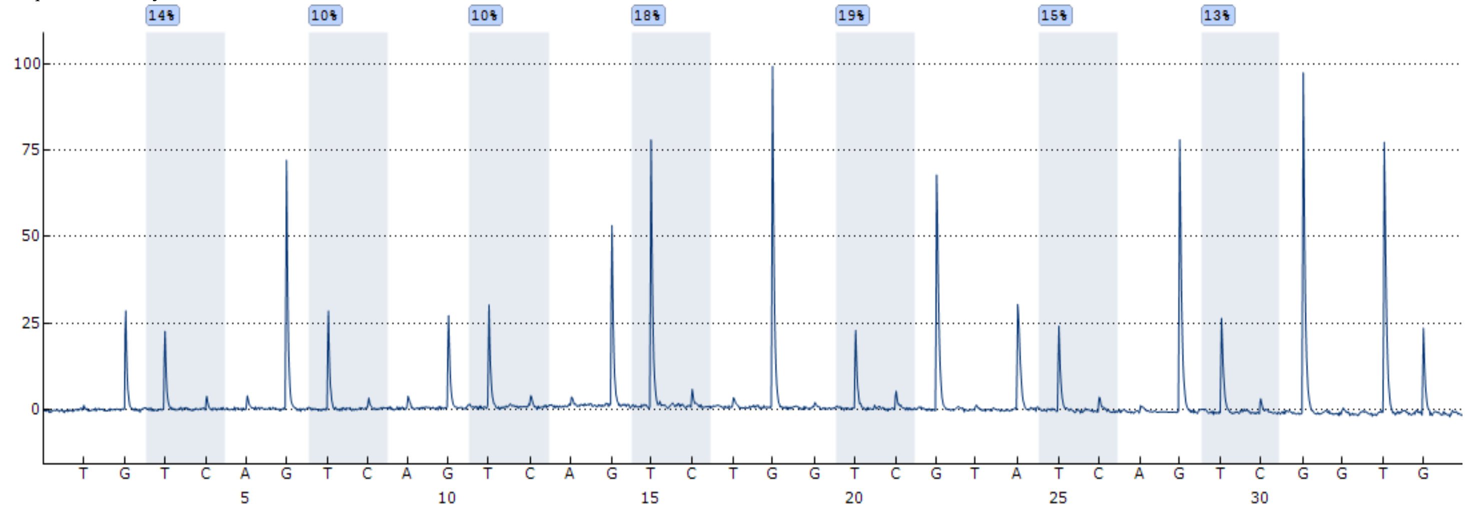

Well: D4
Assay: -1S
Sample ID: 24
Sequence Before Bisulfite Treatment: -
Sequence to analyze: GYGGGYGYGGTTYGGGGYGGAYGGGYGGGGTTTGTAGGGTTTTG

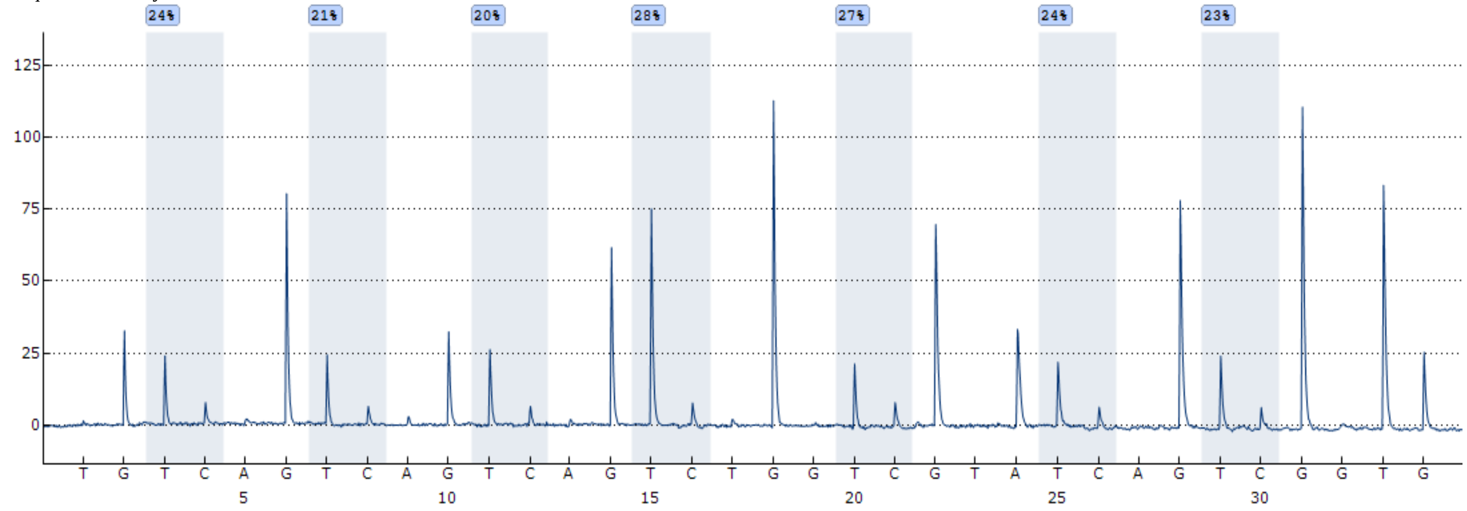

Well: D5
Assay: -1S
Sample ID: 25
Sequence Before Bisulfite Treatment: -
Sequence to analyze: GYGGGYGYGGTTYGGGGYGGAYGGGYGGGGTTTGTAGGGTTTTG

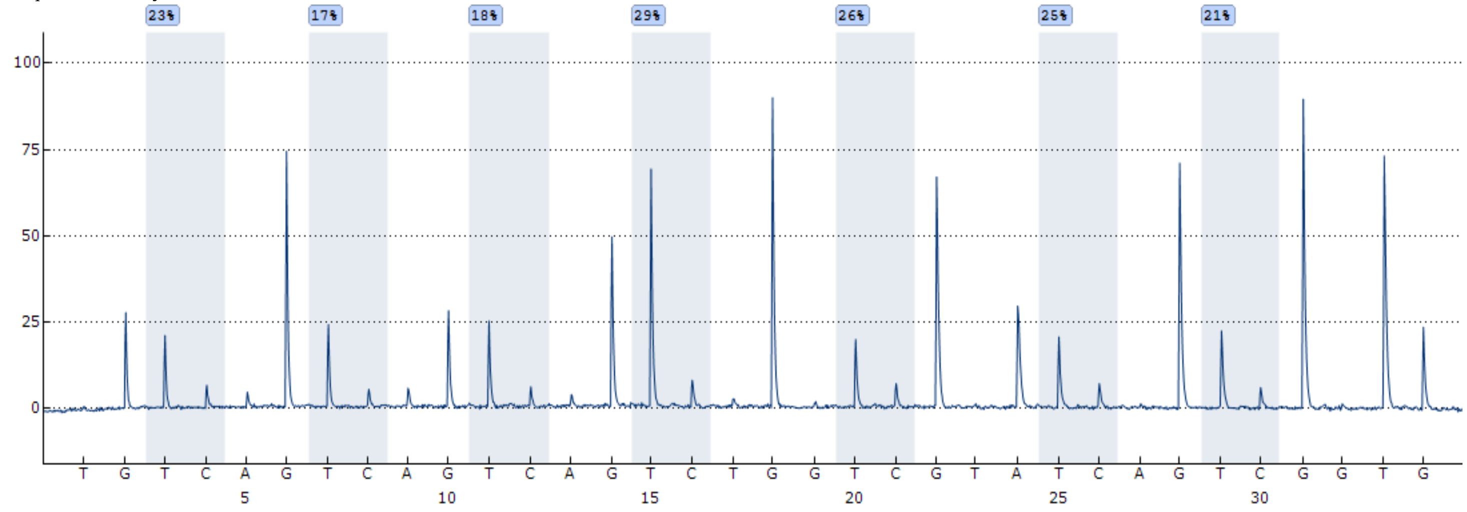

CpG Pyrogram Report Run: 20240801 A

Well: D6
Assay: -1S
Sample ID: 26
Sequence Before Bisulfite Treatment: -
Sequence to analyze: GYGGGYGYGGTTYGGGGYGGAYGGGYGGGGTTTGTAGGGTTTTG

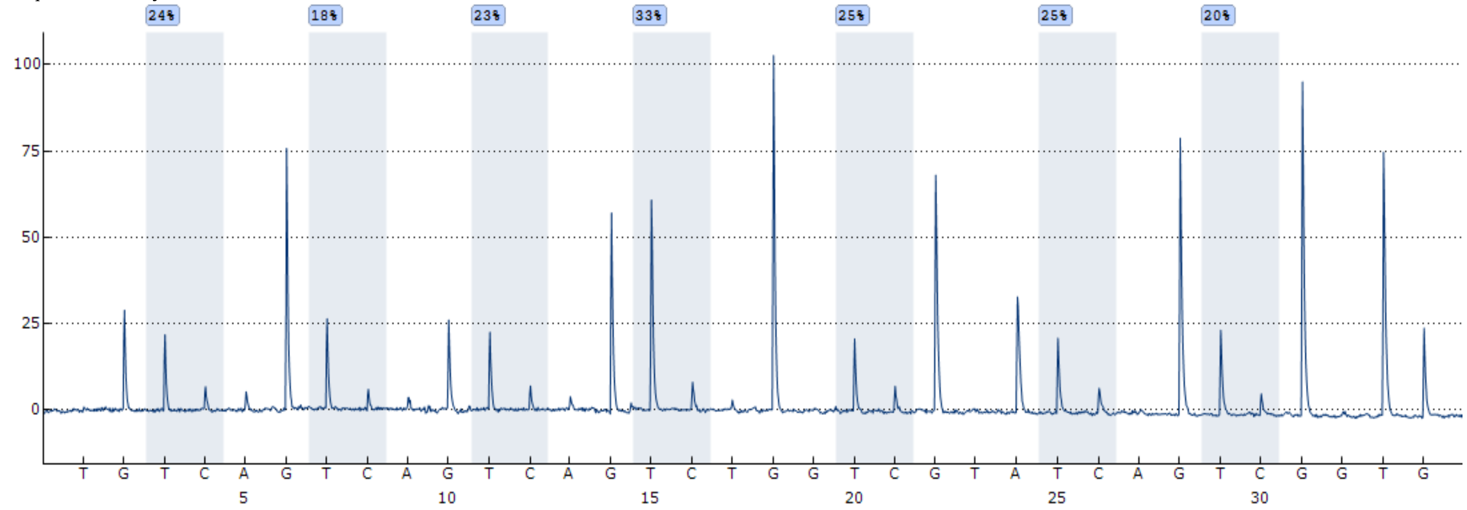

Well: D7
Assay: -1S
Sample ID: 27
Sequence Before Bisulfite Treatment: -
Sequence to analyze: GYGGGYGYGGTTYGGGGYGGAYGGGYGGGGTTTGTAGGGTTTTG

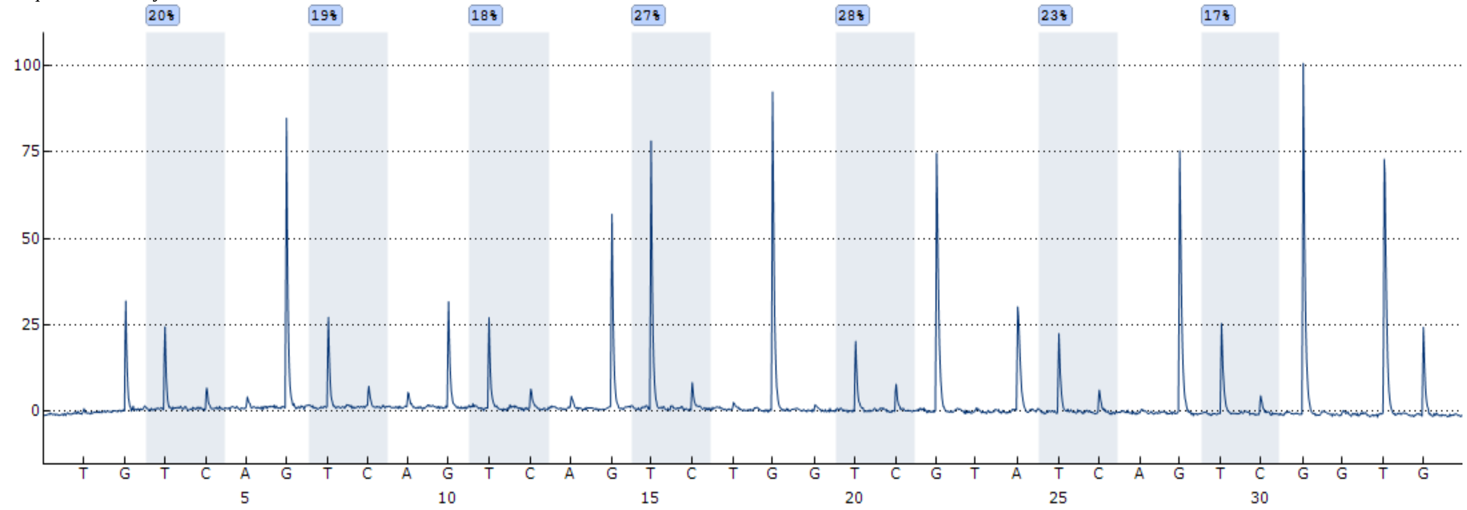

Well: D8
Assay: -1S
Sample ID: 28
Sequence Before Bisulfite Treatment: -
Sequence to analyze: GYGGGYGYGGTTYGGGGYGGAYGGGYGGGGTTTGTAGGGTTTTG

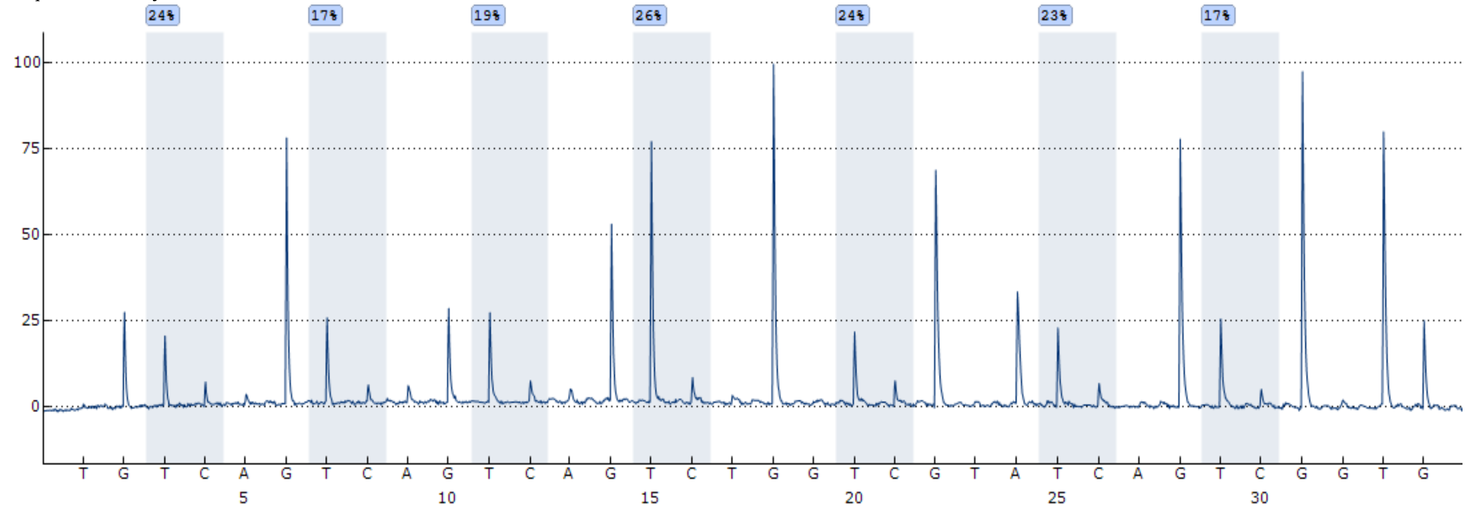

Supplement: Supplementary file 2 [file DataSheet2.zip › Analysis of Methylated Phosphorylation Data(Ca.VS.CON)/大肠癌1S 17-28.pdf]
