# Supplementary material for: Anti-colorectal cancer effects of IRX4 and sensitivity studies to oxaliplatin
Source: Front Immunol. 2026 Jan 21;16:1581244. doi: 10.3389/fimmu.2025.1581244 (PMC12867854; doi:10.3389/fimmu.2025.1581244)

CpG Pyrogram Report  Run: 20240801 B

Well: B8
Assay: -1S
Sample ID: 29
Sequence Before Bisulfite Treatment: -
Sequence to analyze: GYGGGYGYGGTTYGGGGYGGAYGGGYGGGGTTTGTAGGGTTTTG

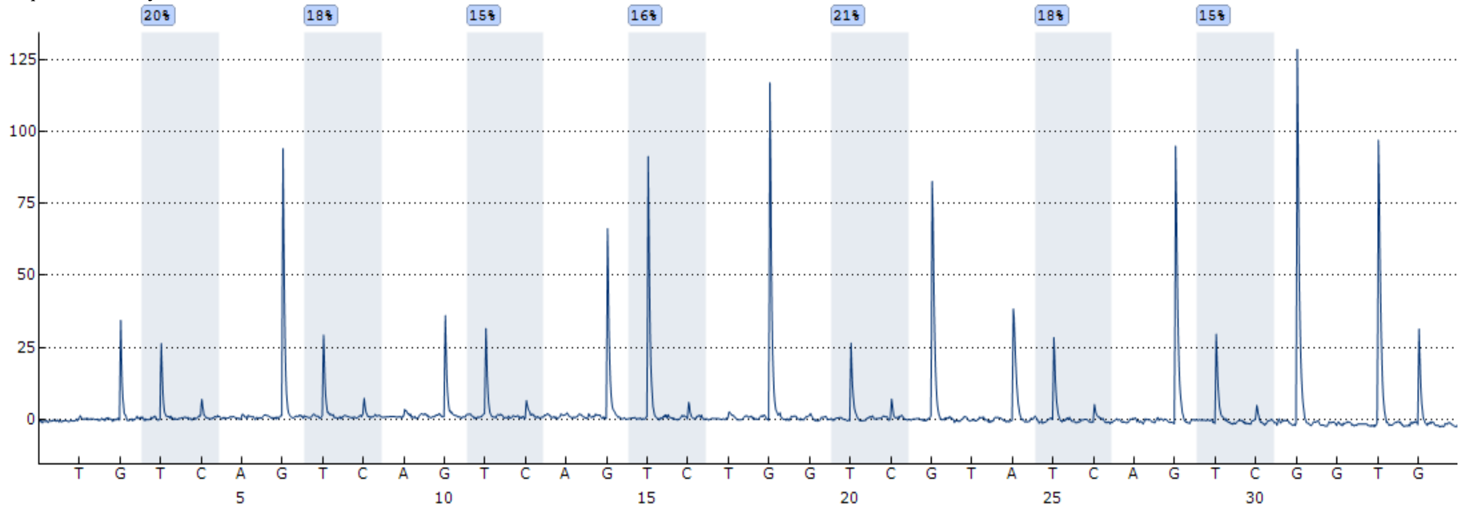


Well: B9
Assay: -1S
Sample ID: 30
Sequence Before Bisulfite Treatment: -
Sequence to analyze: GYGGGYGYGGTTYGGGGYGGAYGGGYGGGGTTTGTAGGGTTTTG

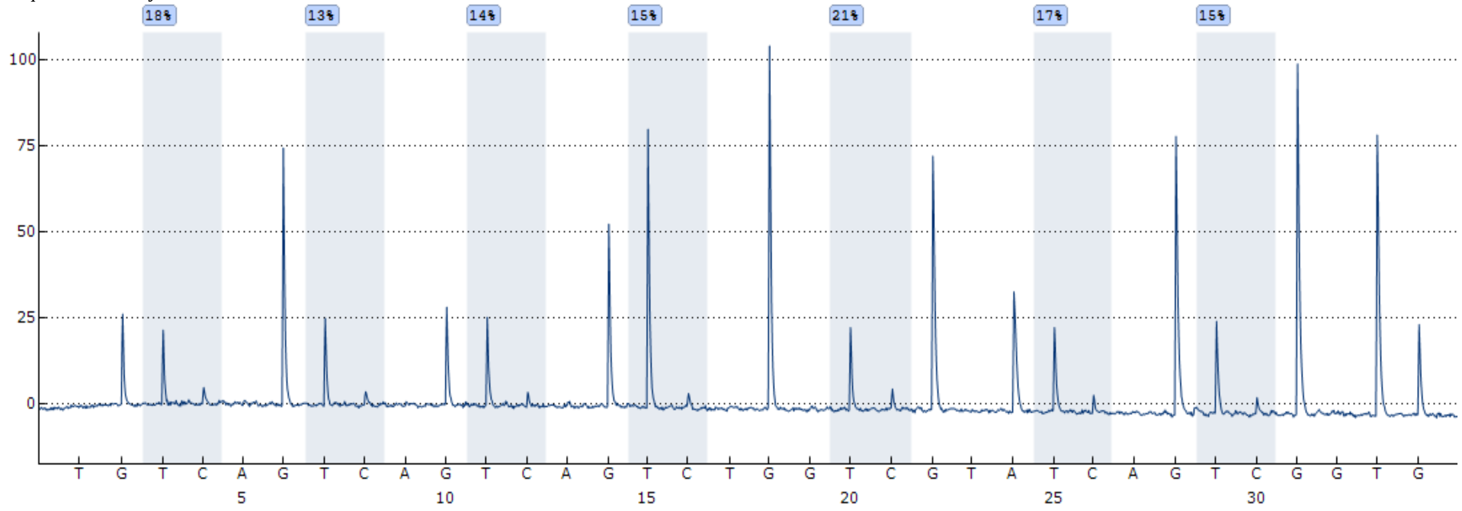


Well: B10
Assay: -1S
Sample ID: 31
Sequence Before Bisulfite Treatment: -
Sequence to analyze: GYGGGYGYGGTTYGGGGYGGAYGGGYGGGGTTTGTAGGGTTTTG

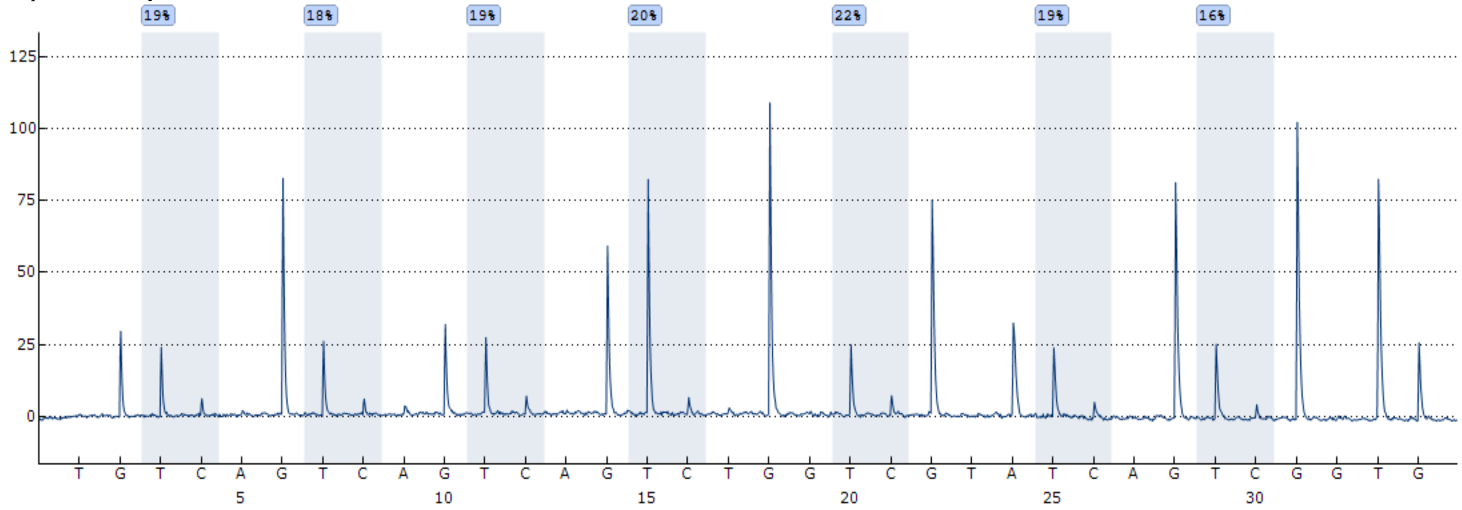

CpG Pyrogram Report  Run: 20240801 B

Well: B11
Assay: -1S
Sample ID: 32
Sequence Before Bisulfite Treatment: -
Sequence to analyze: GYGGGYGYGGTTYGGGGYGGAYGGGYGGGGTTTGTAGGGTTTTG

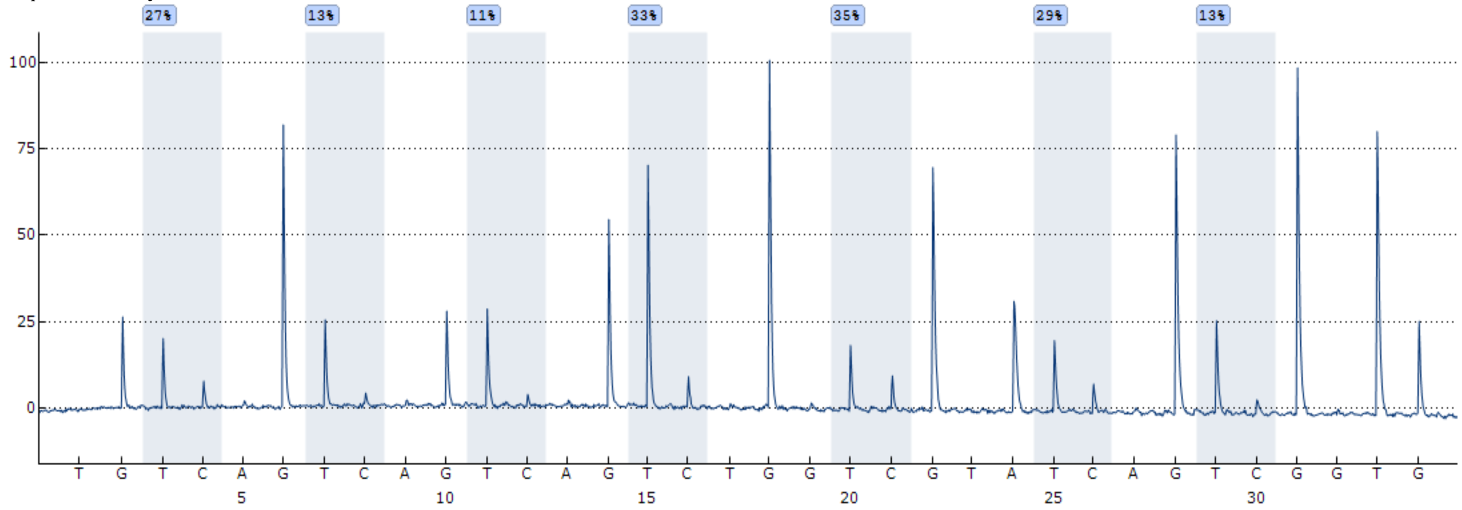


Well: B12
Assay: -1S
Sample ID: 33
Sequence Before Bisulfite Treatment: -
Sequence to analyze: GYGGGYGYGGTTYGGGGYGGAYGGGYGGGGTTTGTAGGGTTTTG

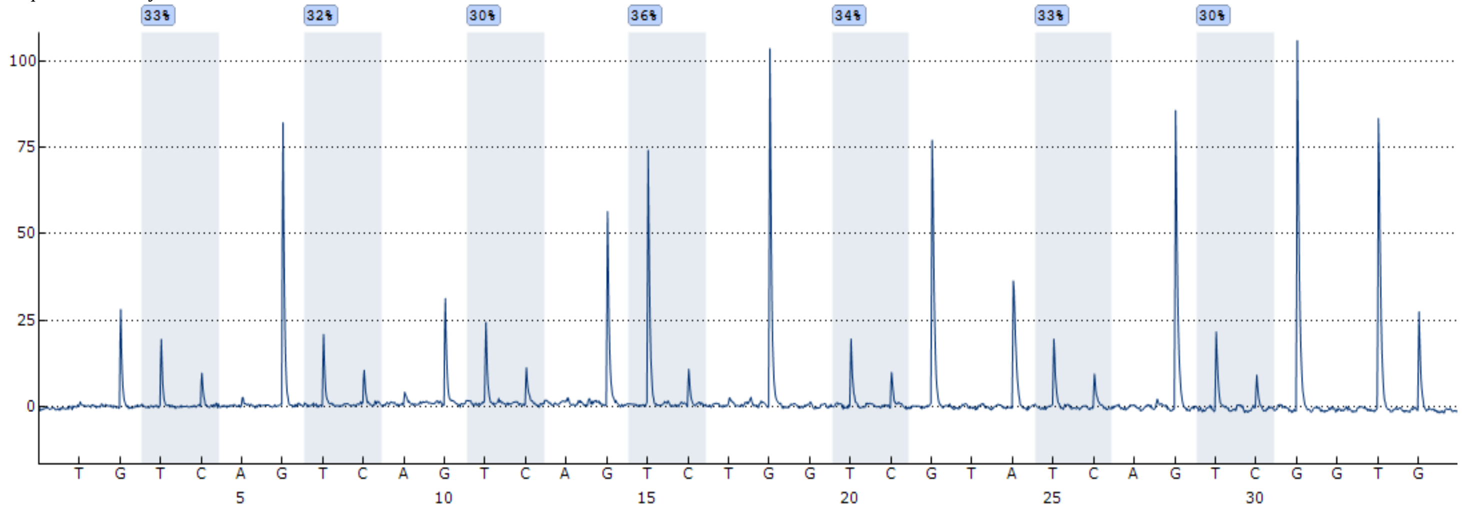


Well: C1
Assay: -1S
Sample ID: 34
Sequence Before Bisulfite Treatment: -
Sequence to analyze: GYGGGYGYGGTTYGGGGYGGAYGGGYGGGGTTTGTAGGGTTTTG

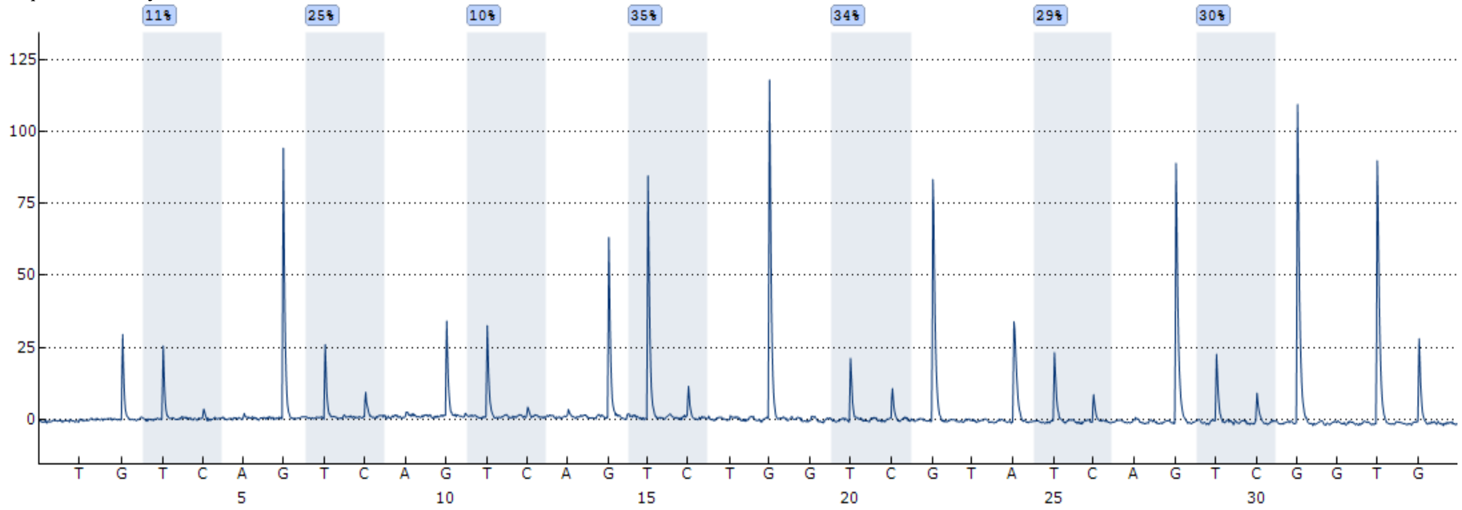

CpG Pyrogram Report  Run: 20240801 B

Well: C2
Assay: -1S
Sample ID: 35
Sequence Before Bisulfite Treatment: -
Sequence to analyze: GYGGGYGYGGTTYGGGGYGGAYGGGYGGGGTTTGTAGGGTTTTG

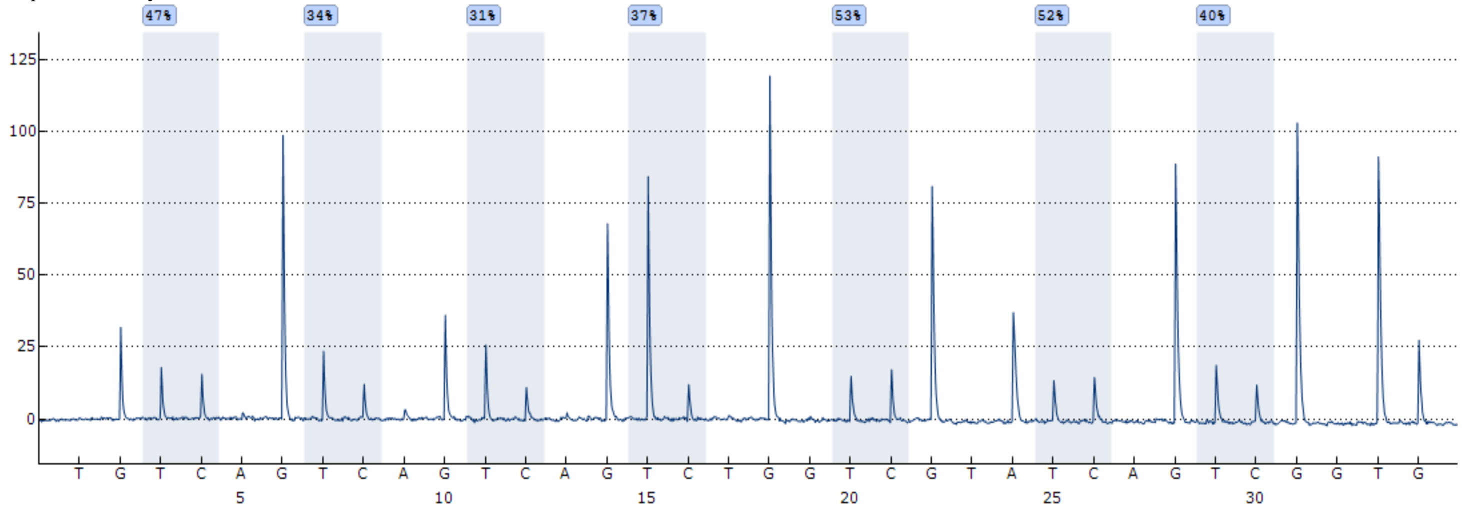


Well: C3
Assay: -1S
Sample ID: 36
Sequence Before Bisulfite Treatment: -
Sequence to analyze: GYGGGYGYGGTTYGGGGYGGAYGGGYGGGGTTTGTAGGGTTTTG

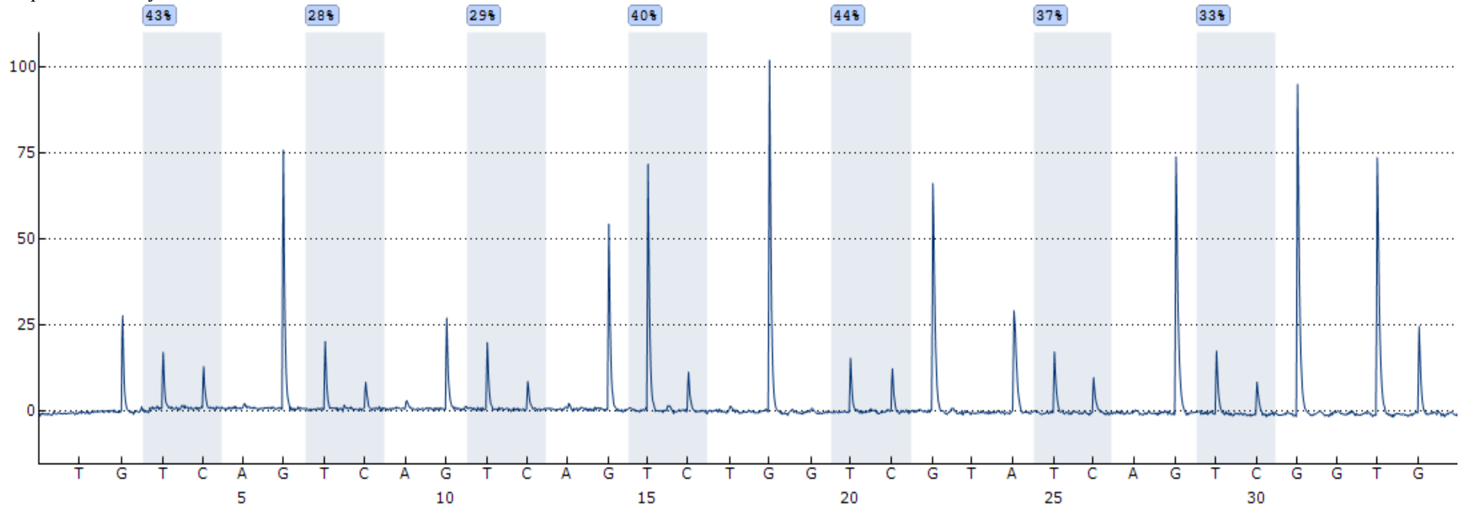


Well: C4
Assay: -1S
Sample ID: 37
Sequence Before Bisulfite Treatment: -
Sequence to analyze: GYGGGYGYGGTTYGGGGYGGAYGGGYGGGGTTTGTAGGGTTTTG

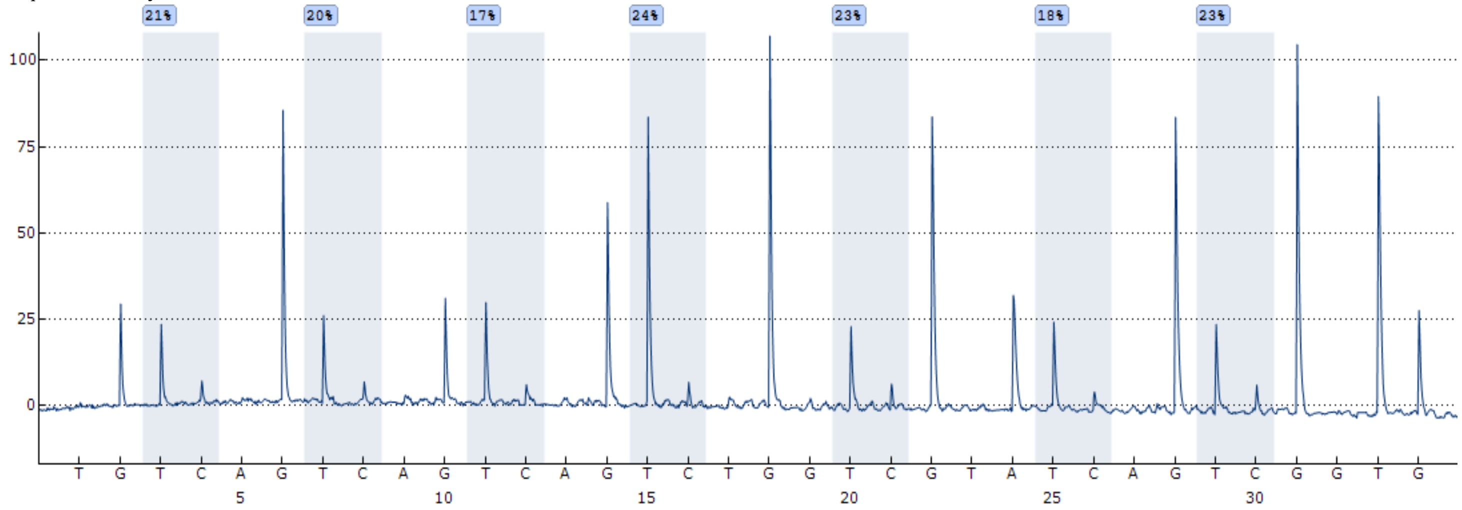

CpG Pyrogram Report  Run: 20240801 B

Well: C5
Assay: -1S
Sample ID: 38
Sequence Before Bisulfite Treatment: -
Sequence to analyze: GYGGGYGYGGTTYGGGGYGGAYGGGYGGGGTTTGTAGGGTTTTG

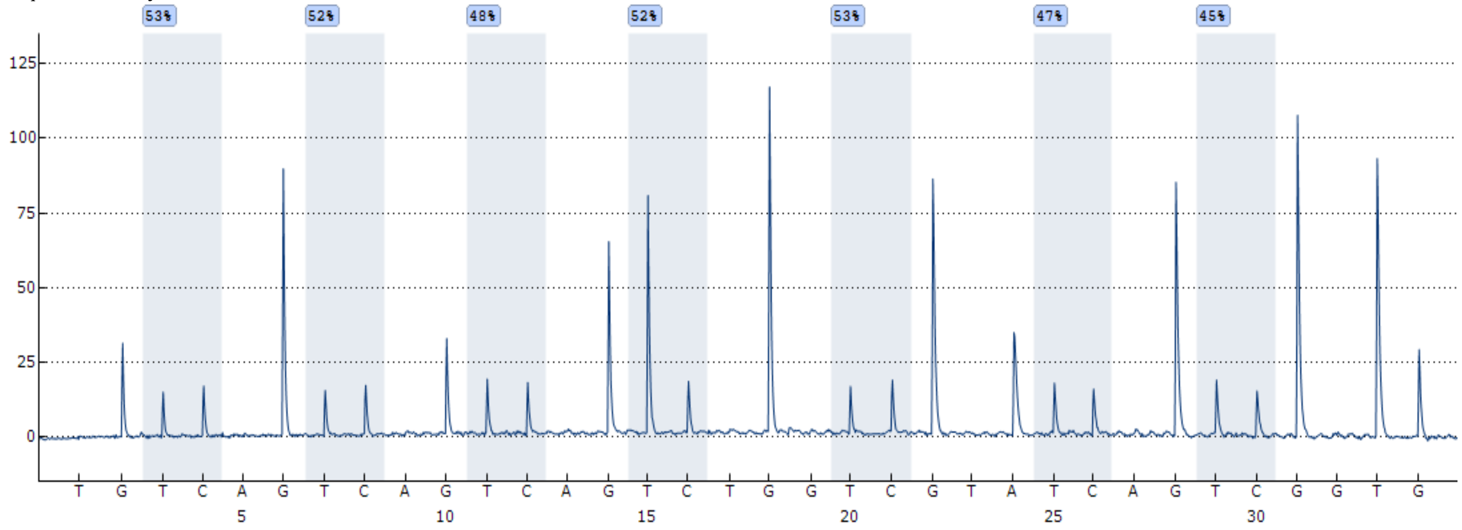


Well: C6
Assay: -1S
Sample ID: 39
Sequence Before Bisulfite Treatment: -
Sequence to analyze: GYGGGYGYGGTTYGGGGYGGAYGGGYGGGGTTTGTAGGGTTTTG

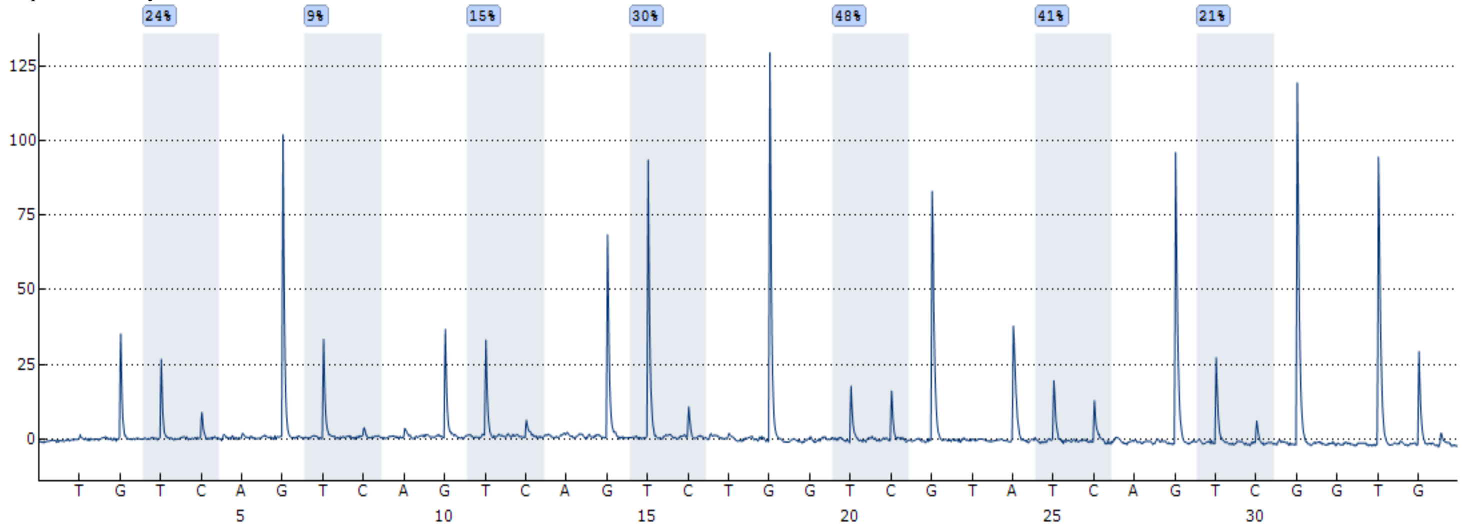

Supplement: Supplementary file 2 [file DataSheet2.zip › Analysis of Methylated Phosphorylation Data(Ca.VS.CON)/大肠癌1S 29-39.pdf]
