# Supplementary material for: Anti-colorectal cancer effects of IRX4 and sensitivity studies to oxaliplatin
Source: Front Immunol. 2026 Jan 21;16:1581244. doi: 10.3389/fimmu.2025.1581244 (PMC12867854; doi:10.3389/fimmu.2025.1581244)

CpG Pyrogram Report Run: 20240801 A

Well: D9
Assay: -2S
Sample ID: 17
Sequence Before Bisulfite Treatment: -
Sequence to analyze: TYGAGYGGTTTYGTAGYGGYGATAGAAATATATATTTTA

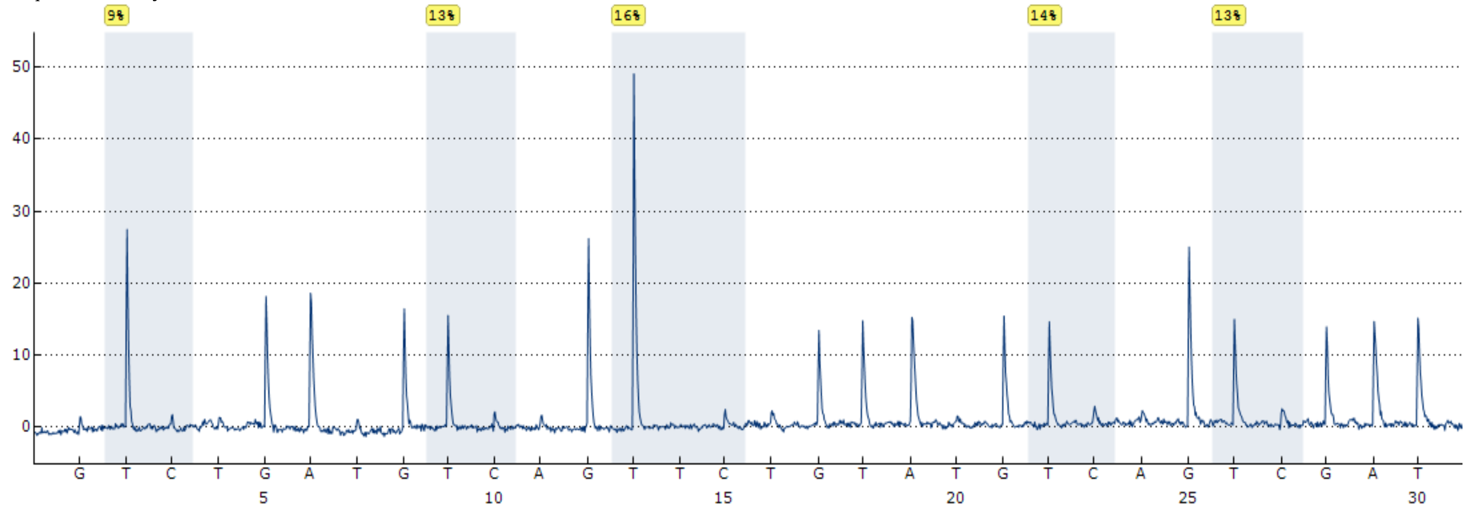

Well: D10
Assay: -2S
Sample ID: 18
Sequence Before Bisulfite Treatment: -
Sequence to analyze: TYGAGYGGTTTYGTAGYGGYGATAGAAATATATATTTTA

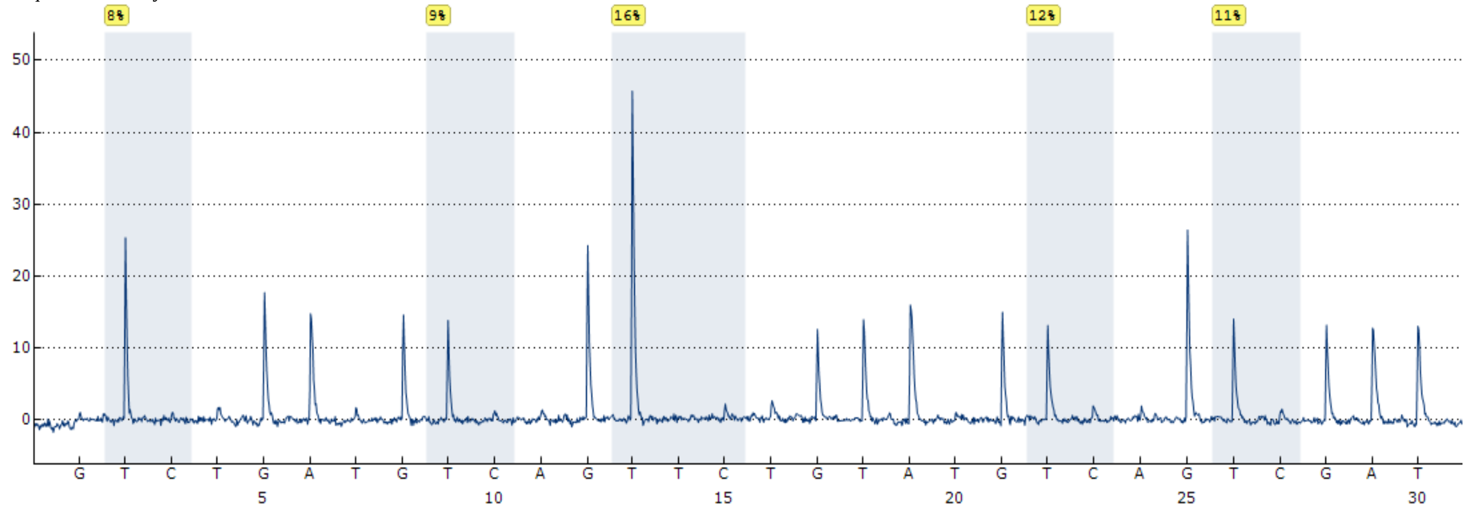

Well: D11
Assay: -2S
Sample ID: 19
Sequence Before Bisulfite Treatment: -
Sequence to analyze: TYGAGYGGTTTYGTAGYGGYGATAGAAATATATATTTTA

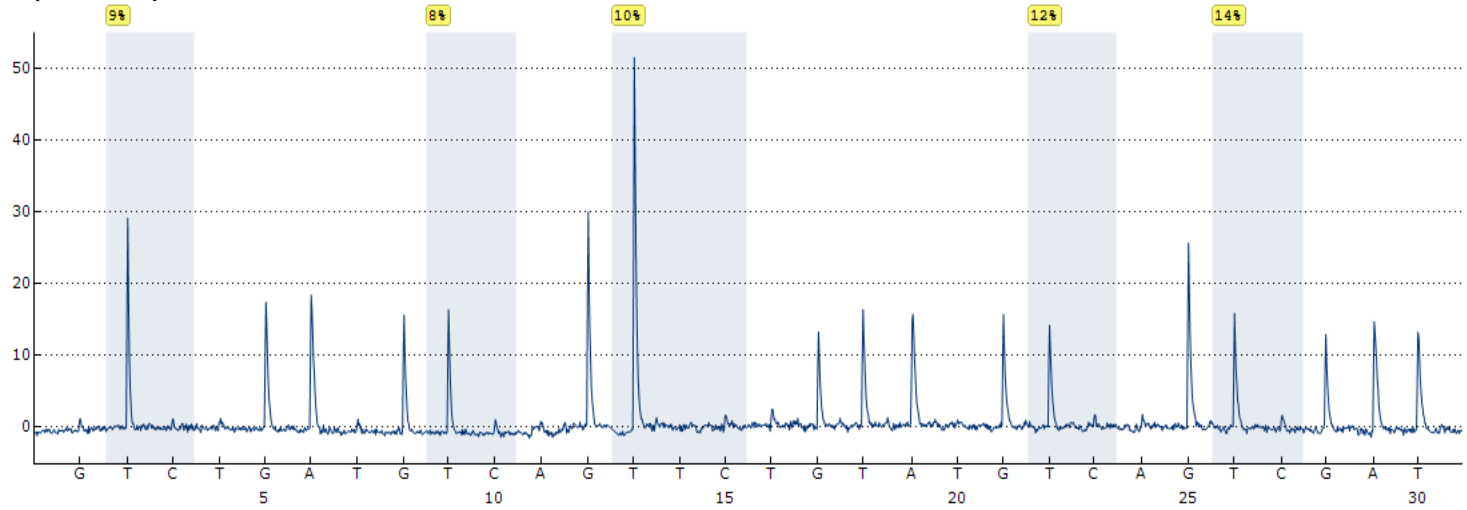

CpG Pyrogram Report Run: 20240801 A

Well: D12
Assay: -2S
Sample ID: 20
Sequence Before Bisulfite Treatment: -
Sequence to analyze: TYGAGYGGTTTYGTAGYGGYGATAGAAATATATATTTTA

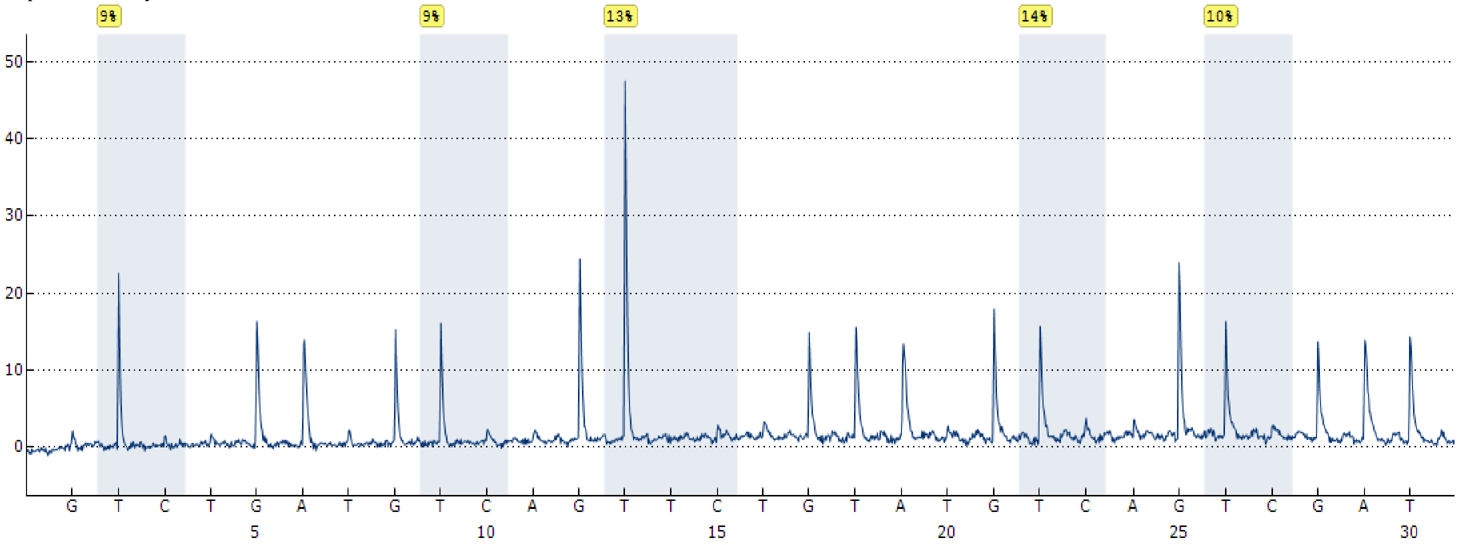

Supplement: Supplementary file 2 [file DataSheet2.zip › Analysis of Methylated Phosphorylation Data(Ca.VS.CON)/大肠癌2S 17-20.pdf]
