# Supplementary material for: Anti-colorectal cancer effects of IRX4 and sensitivity studies to oxaliplatin
Source: Front Immunol. 2026 Jan 21;16:1581244. doi: 10.3389/fimmu.2025.1581244 (PMC12867854; doi:10.3389/fimmu.2025.1581244)

CpG Pyrogram Report Run: 20240801 B

Well: A1
Assay: -2S
Sample ID: 21
Sequence Before Bisulfite Treatment: -
Sequence to analyze: TYGAGYGGTTTYGTAGYGGYGATAGAAATATATATTTTA
Analysis parameters have been edited.

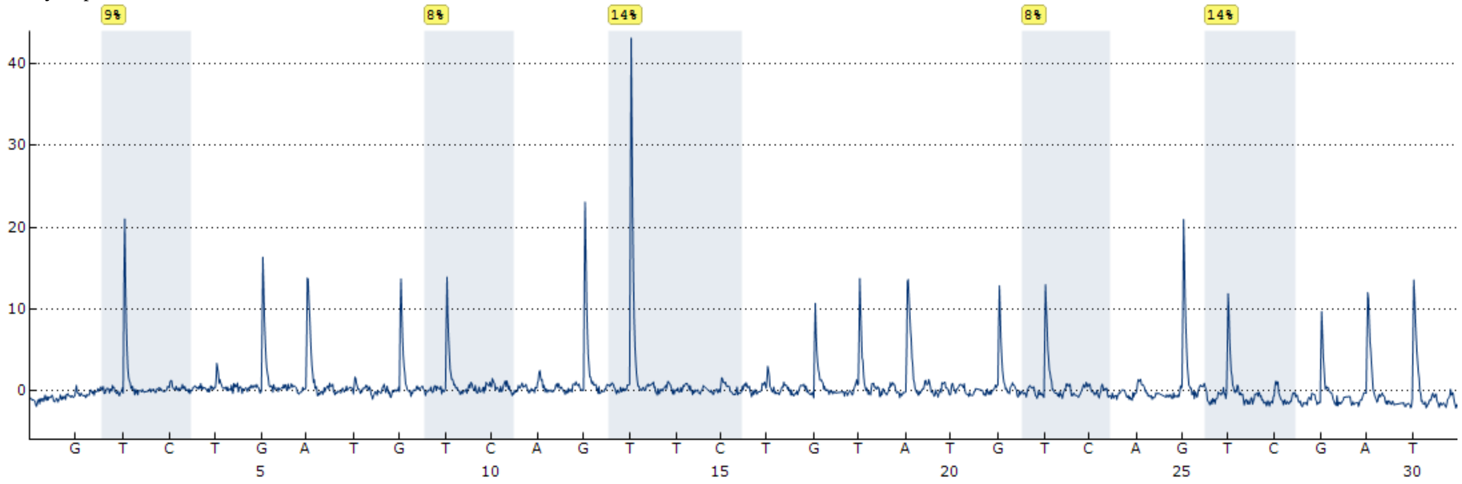

Well: A2
Assay: -2S
Sample ID: 22
Sequence Before Bisulfite Treatment: -
Sequence to analyze: TYGAGYGGTTTYGTAGYGGYGATAGAAATATATATTTTA
Analysis parameters have been edited.

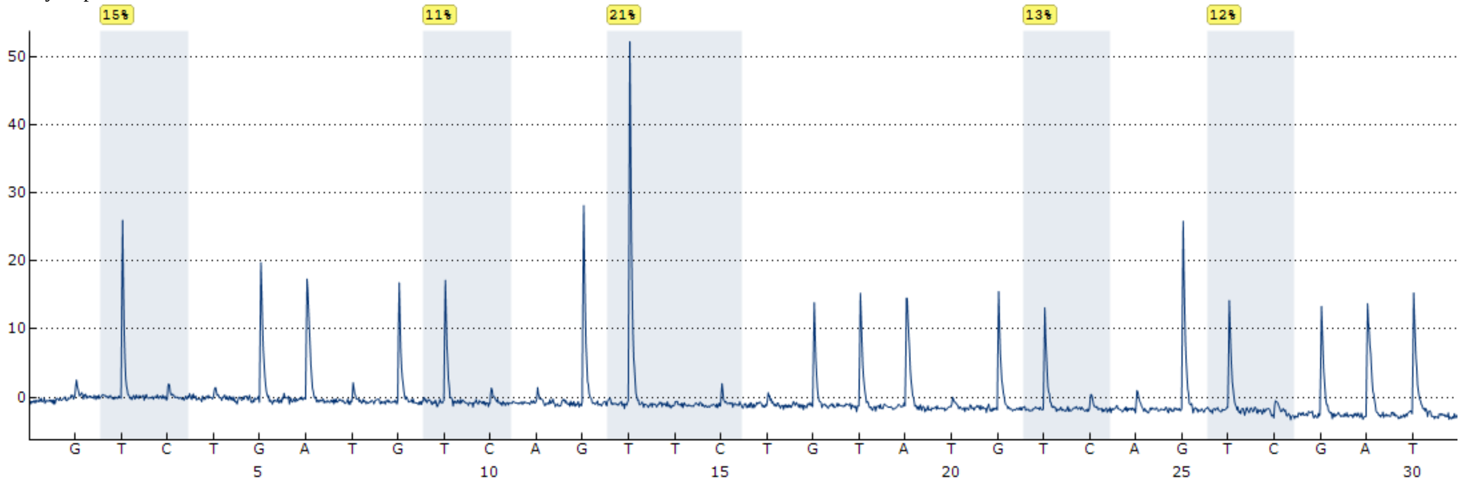

Well: A3
Assay: -2S
Sample ID: 23
Sequence Before Bisulfite Treatment: -
Sequence to analyze: TYGAGYGGTTTYGTAGYGGYGATAGAAATATATATTTTA
Analysis parameters have been edited.

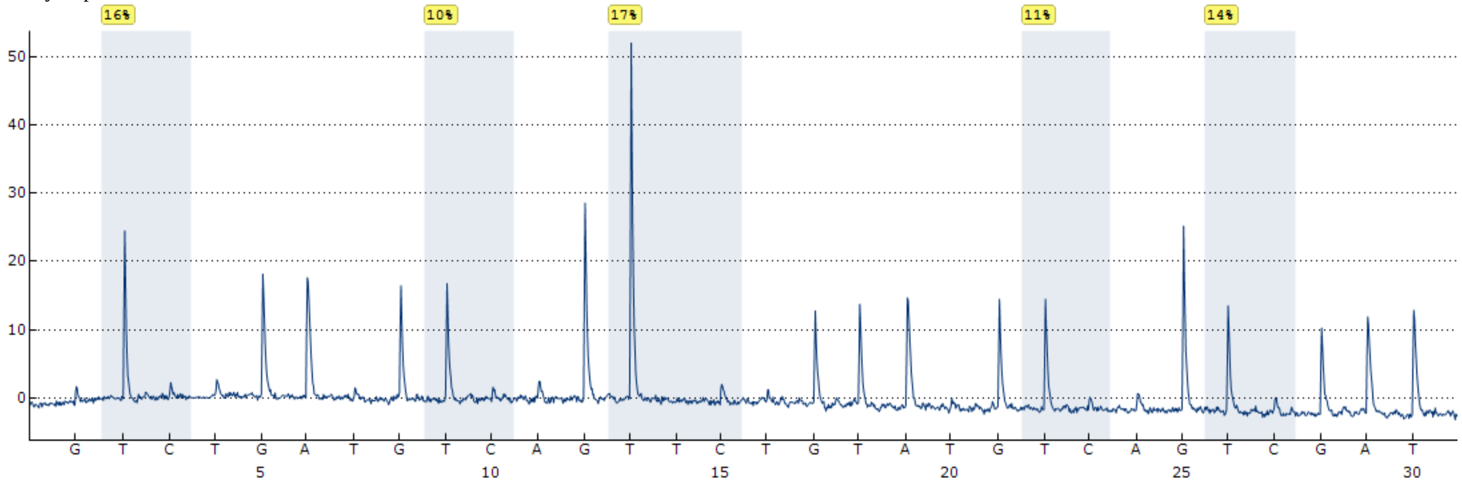

CpG Pyrogram Report Run: 20240801 B

Well: A4
Assay: -2S
Sample ID: 24
Sequence Before Bisulfite Treatment: -
Sequence to analyze: TYGAGYGGTTTYGTAGYGGYGATAGAAATATATATTTTA
Analysis parameters have been edited.

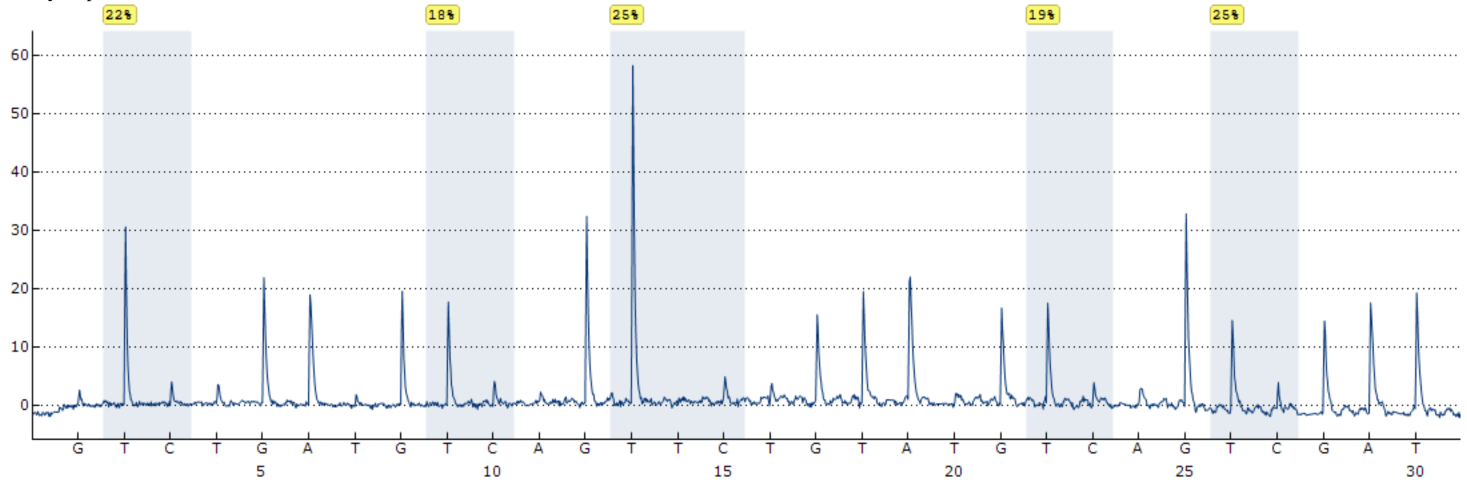

Well: A5
Assay: -2S
Sample ID: 25
Sequence Before Bisulfite Treatment: -
Sequence to analyze: TYGAGYGGTTTYGTAGYGGYGATAGAAATATATATTTTA
Analysis parameters have been edited.

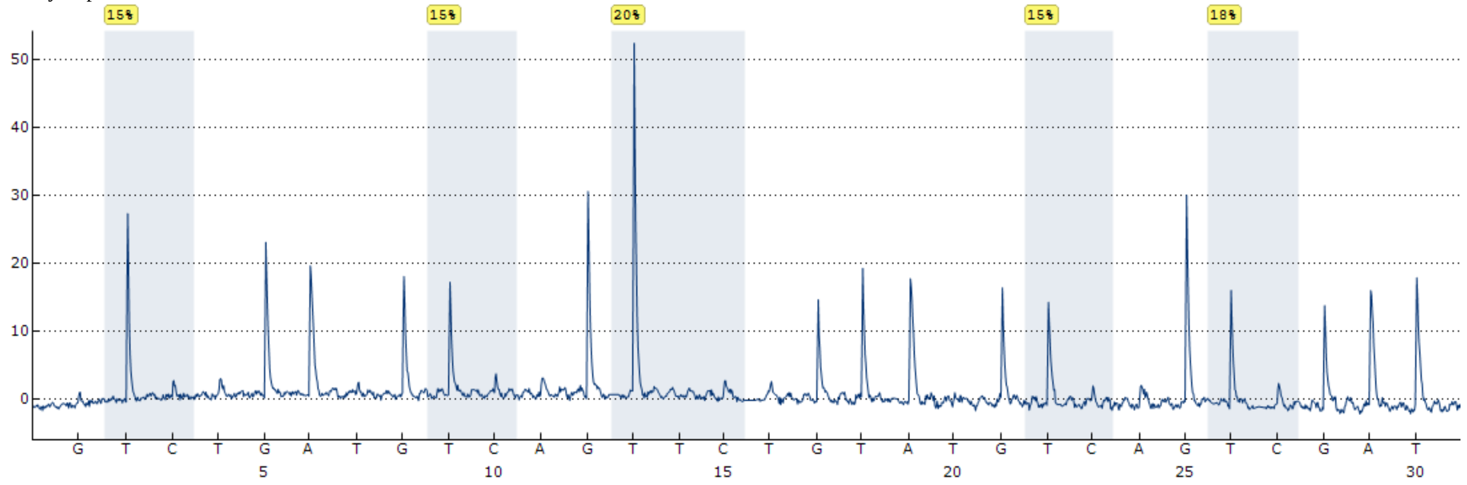

Well: A6
Assay: -2S
Sample ID: 26
Sequence Before Bisulfite Treatment: -
Sequence to analyze: TYGAGYGGTTTYGTAGYGGYGATAGAAATATATATTTTA
Analysis parameters have been edited.

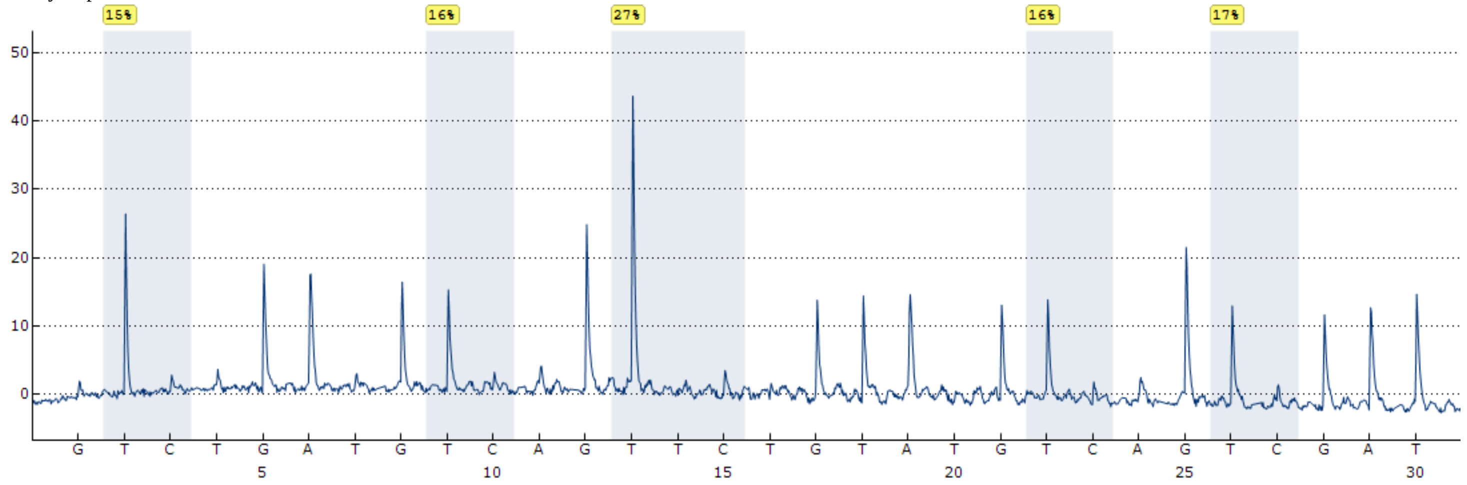

CpG Pyrogram Report Run: 20240801 B

Well: A7
Assay: -2S
Sample ID: 27
Sequence Before Bisulfite Treatment: -
Sequence to analyze: TYGAGYGGTTTYGTAGYGGYGATAGAAATATATATTTTA
Analysis parameters have been edited.

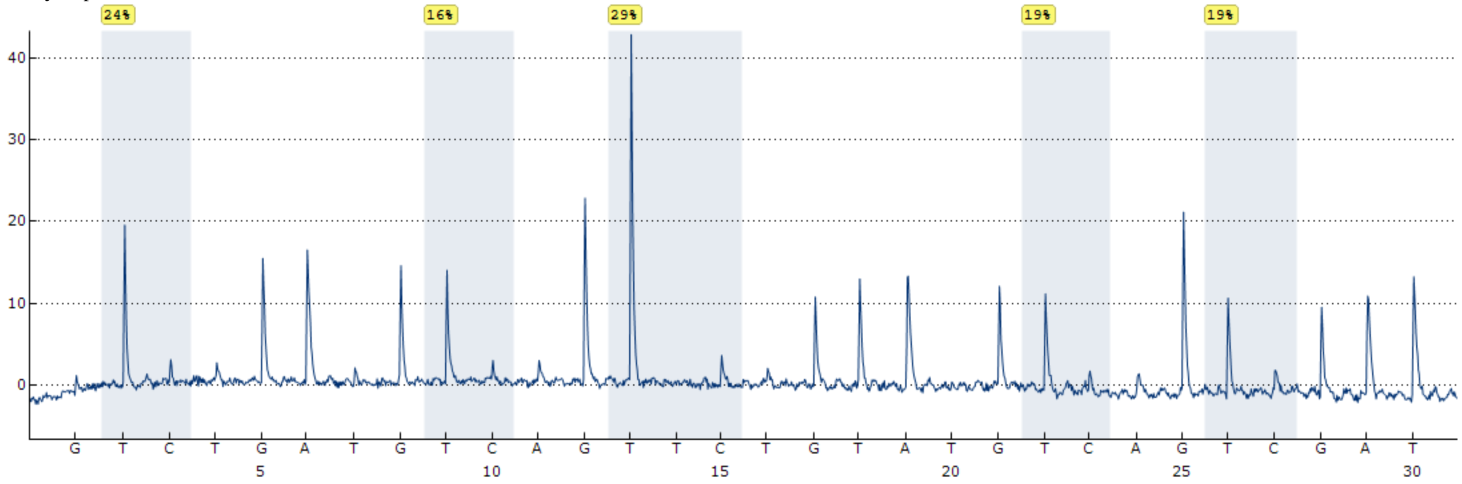

Well: A8
Assay: -2S
Sample ID: 28
Sequence Before Bisulfite Treatment: -
Sequence to analyze: TYGAGYGGTTTYGTAGYGGYGATAGAAATATATATTTTA
Analysis parameters have been edited.

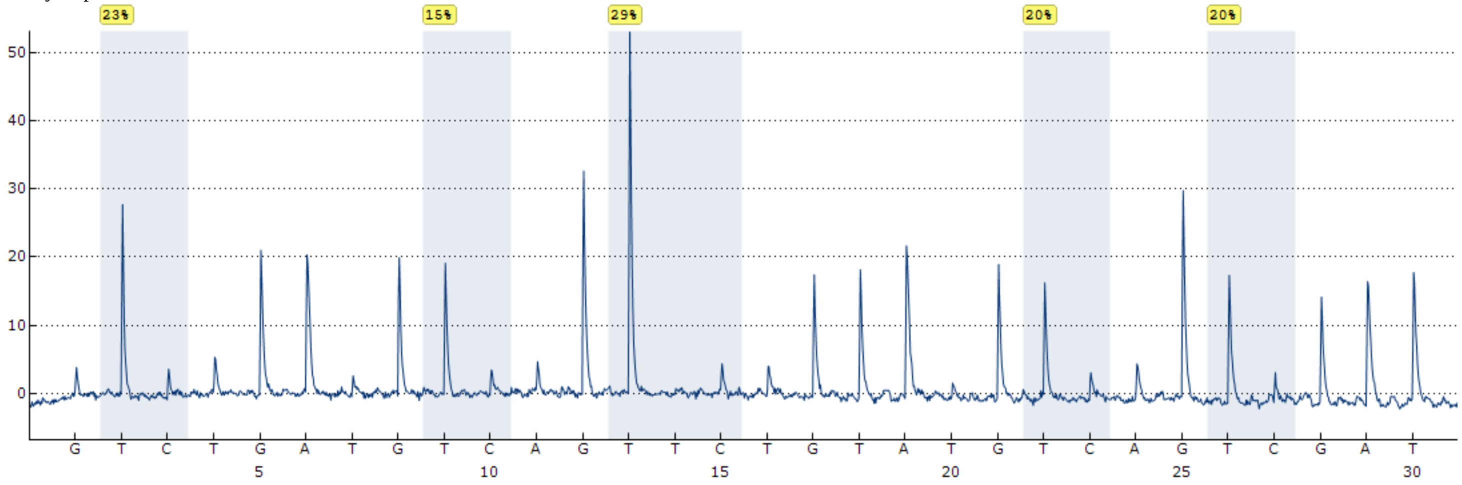

Well: A9
Assay: -2S
Sample ID: 29
Sequence Before Bisulfite Treatment: -
Sequence to analyze: TYGAGYGGTTTYGTAGYGGYGATAGAAATATATATTTTA
Analysis parameters have been edited.

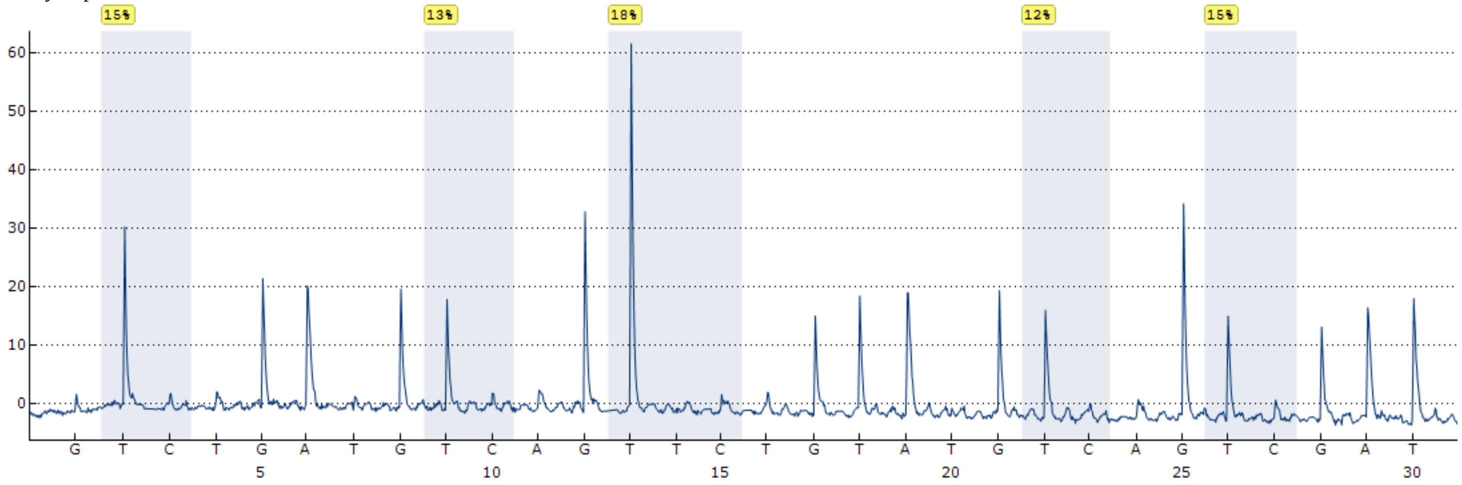

CpG Pyrogram Report Run: 20240801 B

Well: A10
Assay: -2S
Sample ID: 30
Sequence Before Bisulfite Treatment: -
Sequence to analyze: TYGAGYGGTTTYGTAGYGGYGATAGAAATATATATTTTA
Analysis parameters have been edited.

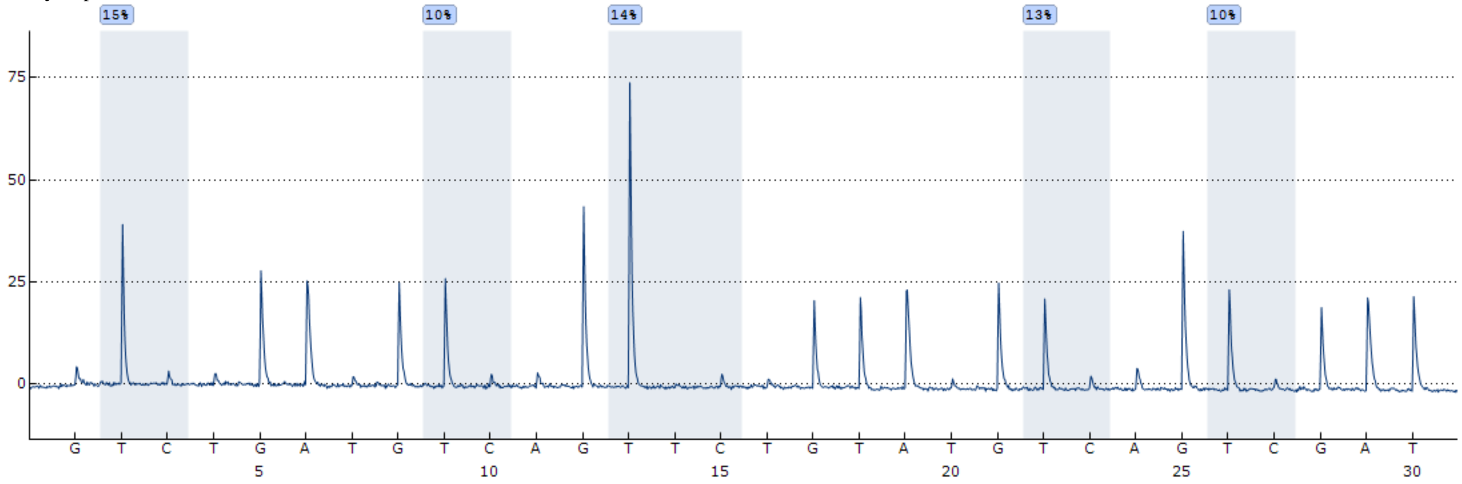

Well: A11
Assay: -2S
Sample ID: 31
Sequence Before Bisulfite Treatment: -
Sequence to analyze: TYGAGYGGTTTYGTAGYGGYGATAGAAATATATATTTTA
Analysis parameters have been edited.

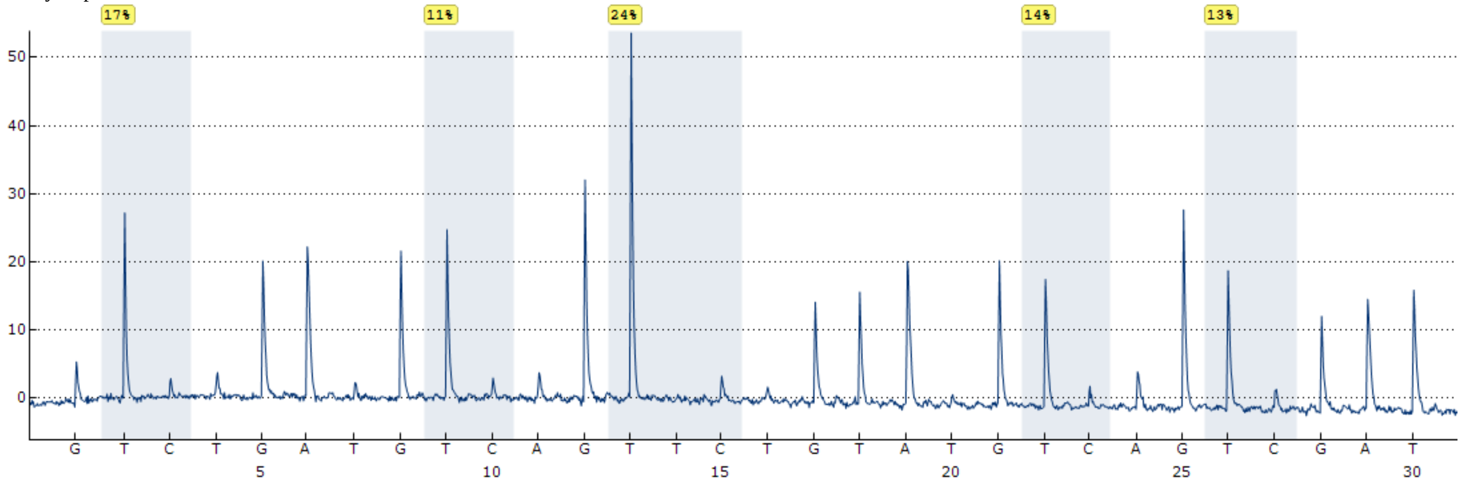

Well: A12
Assay: -2S
Sample ID: 32
Sequence Before Bisulfite Treatment: -
Sequence to analyze: TYGAGYGGTTTYGTAGYGGYGATAGAAATATATATTTTA
Analysis parameters have been edited.

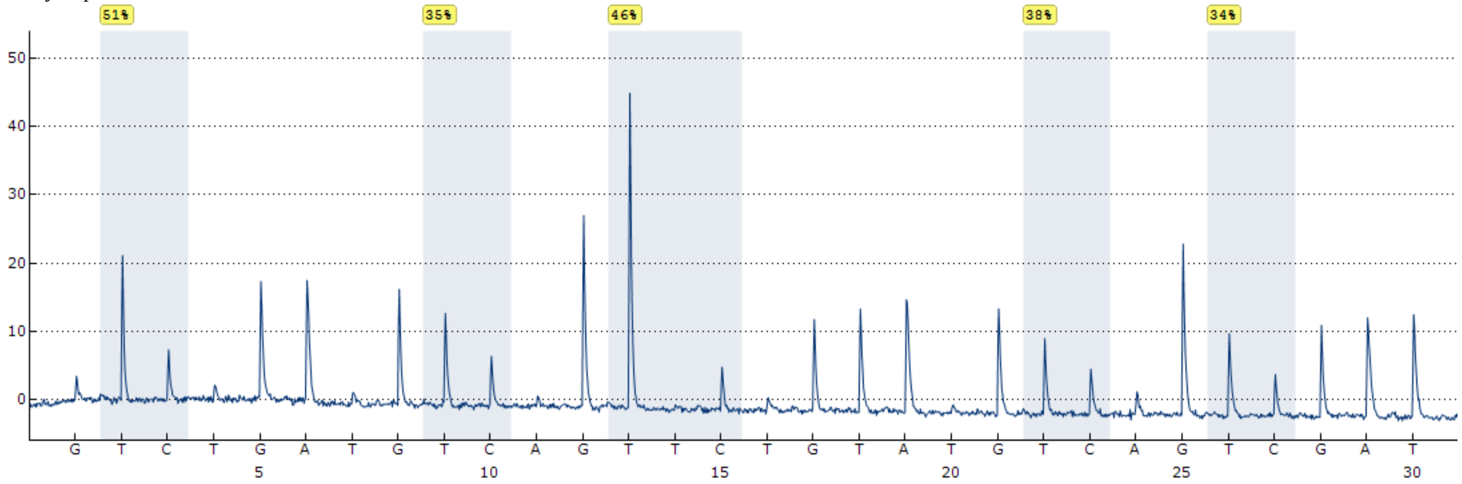

CpG Pyrogram Report Run: 20240801 B

Well: B1
Assay: -2S
Sample ID: 33
Sequence Before Bisulfite Treatment: -
Sequence to analyze: TYGAGYGGTTTYGTAGYGGYGATAGAAATATATATTTTA
Analysis parameters have been edited.

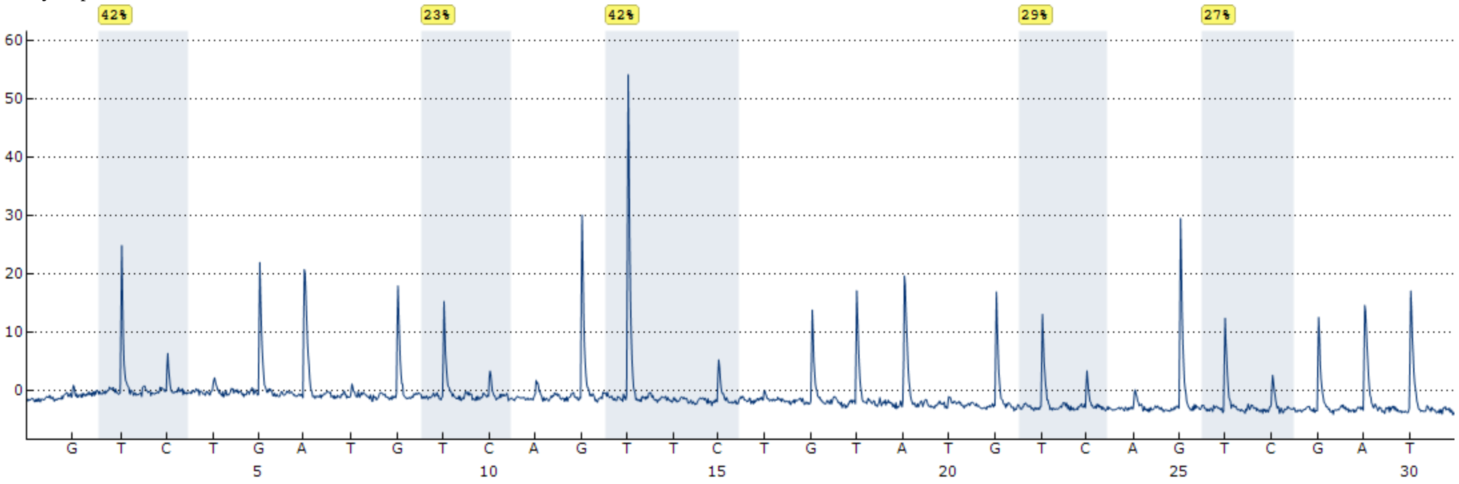

Well: B2
Assay: -2S
Sample ID: 34
Sequence Before Bisulfite Treatment: -
Sequence to analyze: TYGAGYGGTTTYGTAGYGGYGATAGAAATATATATTTTA
Analysis parameters have been edited.

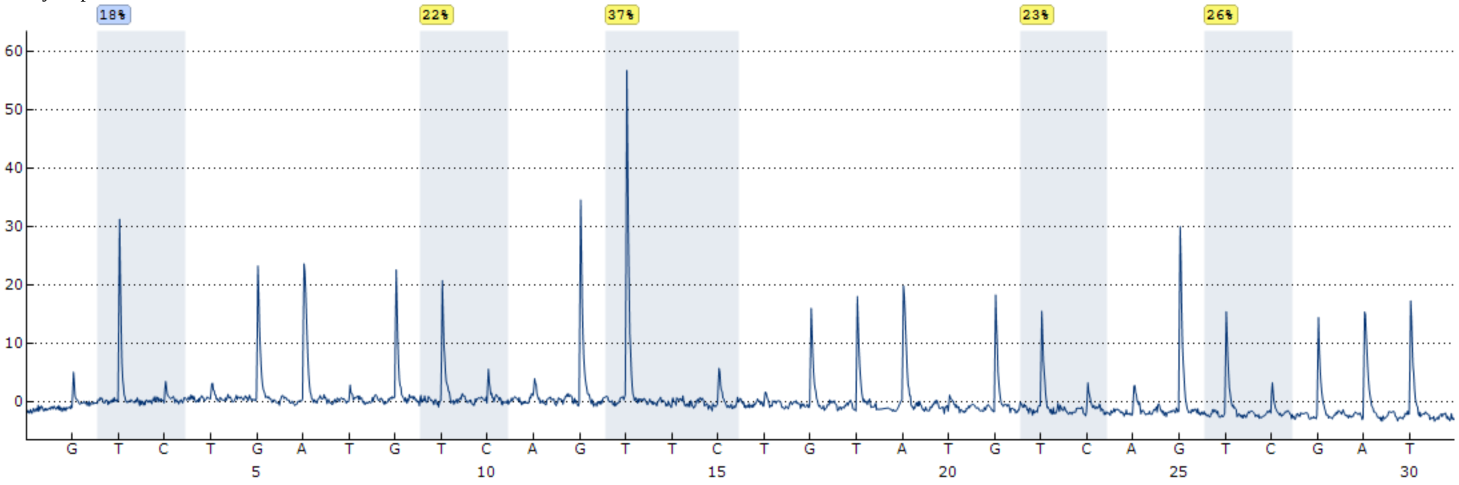

Well: B3
Assay: -2S
Sample ID: 35
Sequence Before Bisulfite Treatment: -
Sequence to analyze: TYGAGYGGTTTYGTAGYGGYGATAGAAATATATATTTTA
Analysis parameters have been edited.

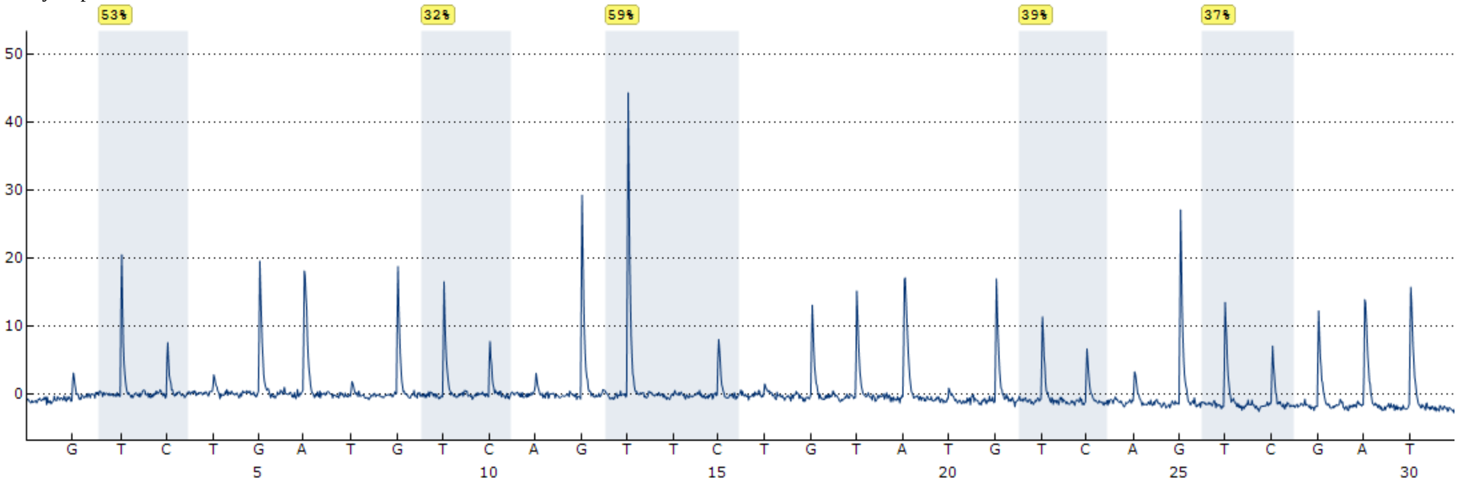

CpG Pyrogram Report Run: 20240801 B

Well: B4
Assay: -2S
Sample ID: 36
Sequence Before Bisulfite Treatment: -
Sequence to analyze: TYGAGYGGTTTYGTAGYGGYGATAGAAATATATATTTTA
Analysis parameters have been edited.

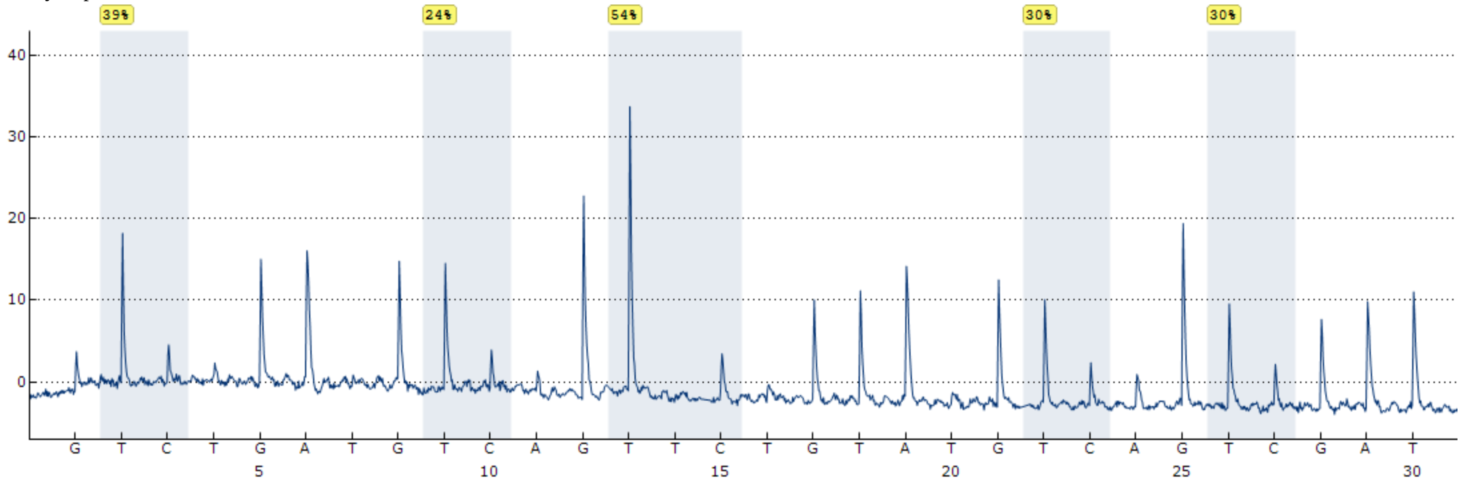

Well: B5
Assay: -2S
Sample ID: 37
Sequence Before Bisulfite Treatment: -
Sequence to analyze: TYGAGYGGTTTYGTAGYGGYGATAGAAATATATATTTTA
Analysis parameters have been edited.

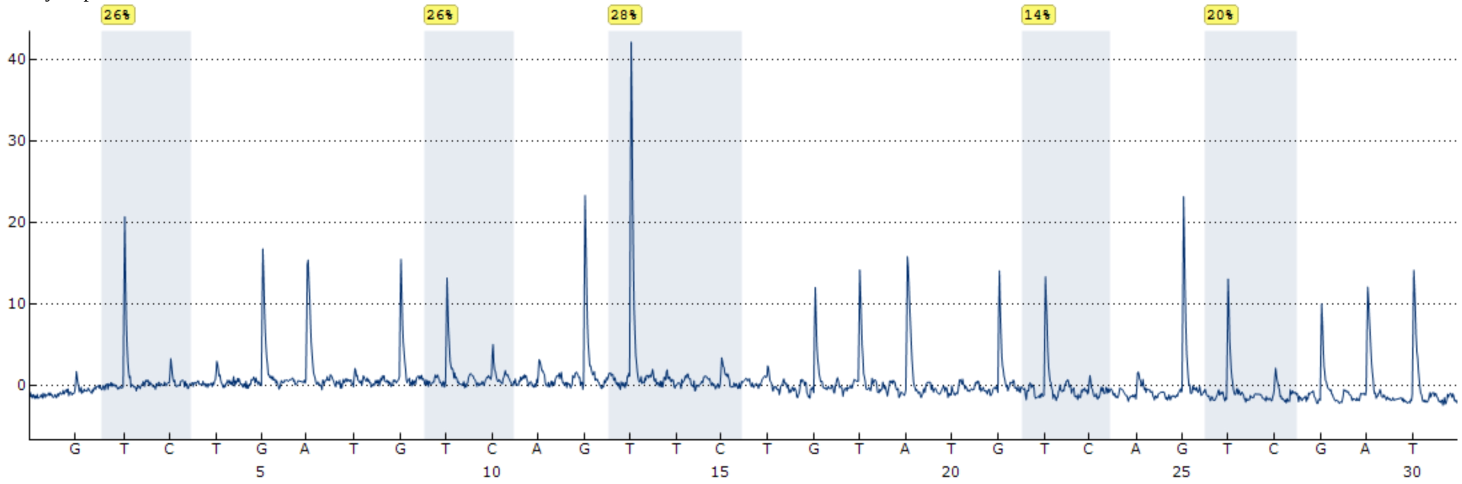

Well: B6
Assay: -2S
Sample ID: 38
Sequence Before Bisulfite Treatment: -
Sequence to analyze: TYGAGYGGTTTYGTAGYGGYGATAGAAATATATATTTTA
Analysis parameters have been edited.

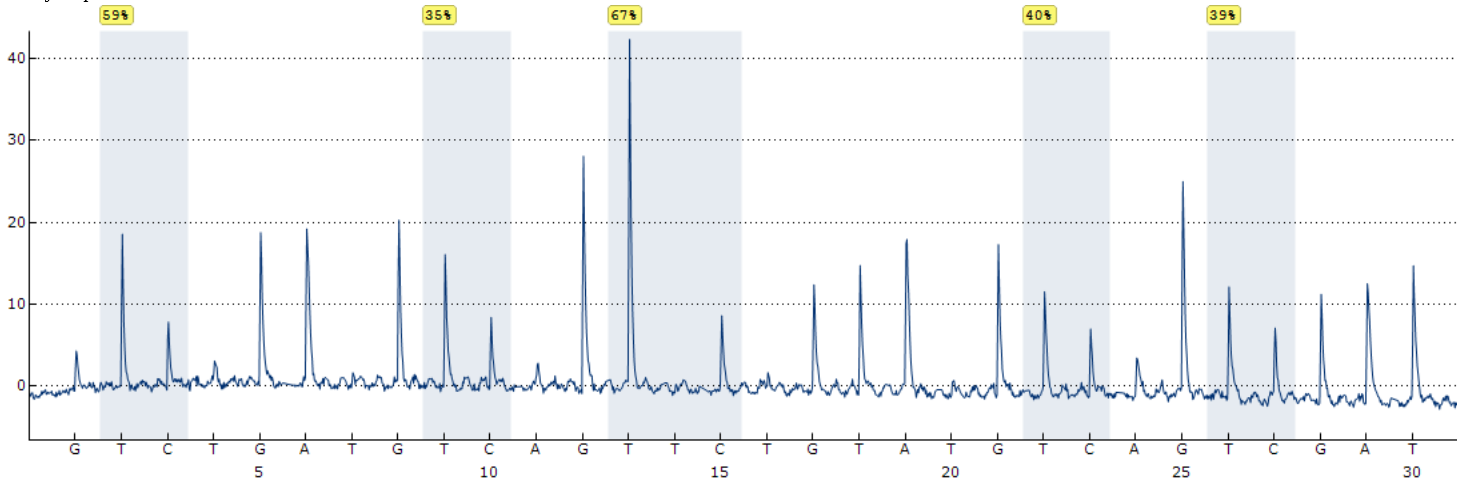

CpG Pyrogram Report Run: 20240801 B

Well: B7
Assay: -2S
Sample ID: 39
Sequence Before Bisulfite Treatment: -
Sequence to analyze: TYGAGYGGTTTYGTAGYGGYGATAGAAATATATATTTTA
Analysis parameters have been edited.

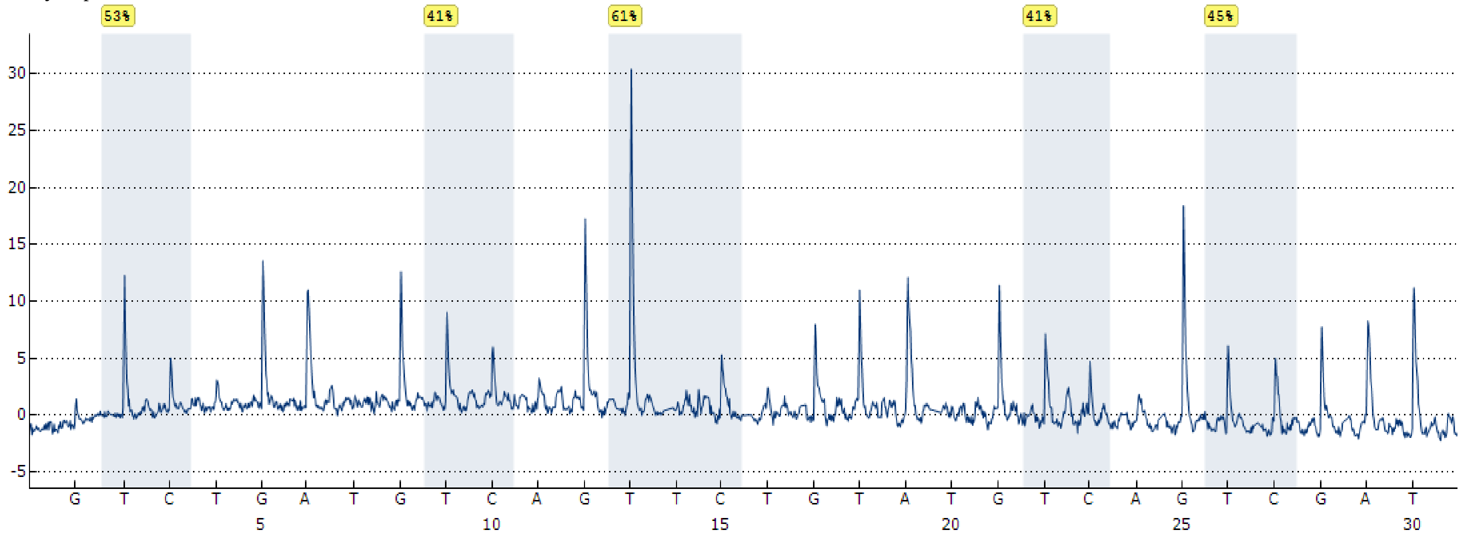

Supplement: Supplementary file 2 [file DataSheet2.zip › Analysis of Methylated Phosphorylation Data(Ca.VS.CON)/大肠癌2S 21-39.pdf]
