# Supplementary figures and images for: Anti-colorectal cancer effects of IRX4 and sensitivity studies to oxaliplatin (part 1 of 2)
Source: Front Immunol. 2026 Jan 21;16:1581244. doi: 10.3389/fimmu.2025.1581244 (PMC12867854; doi:10.3389/fimmu.2025.1581244)

## Ca vs CON

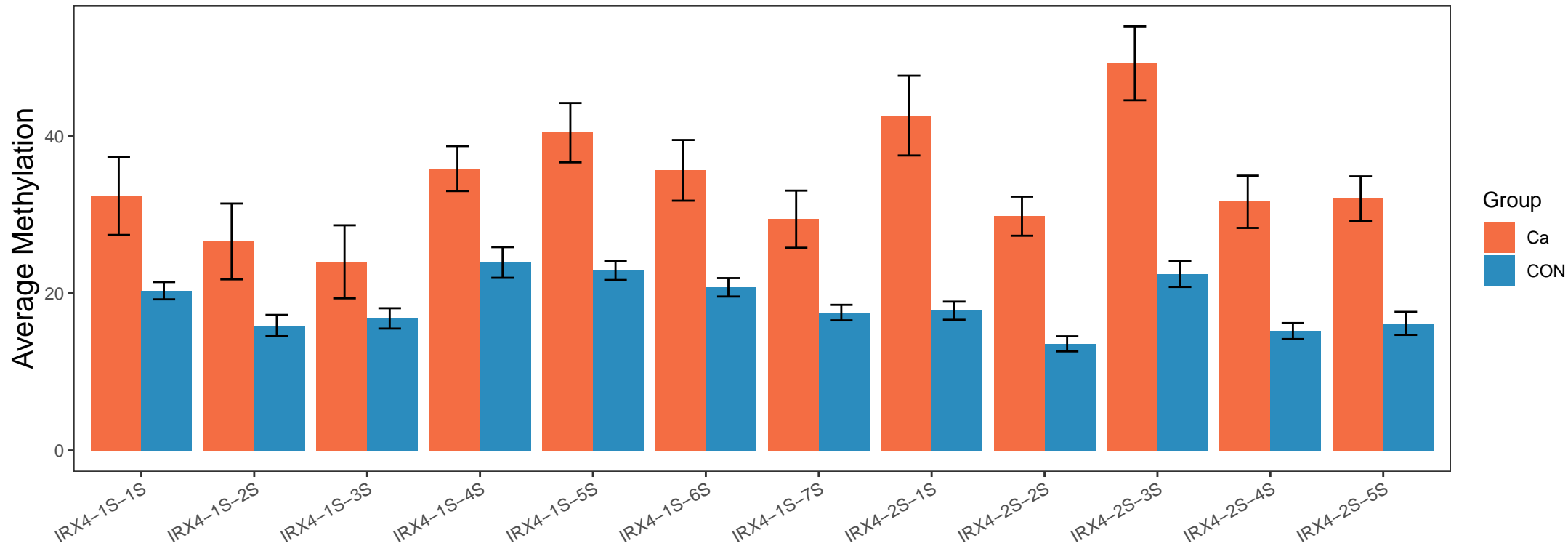

Supplement: Supplementary file 2 [file DataSheet2.zip › Analysis of Methylated Phosphorylation Data(Ca.VS.CON)/ALL_barplot.Ca.VS.CON.pdf]

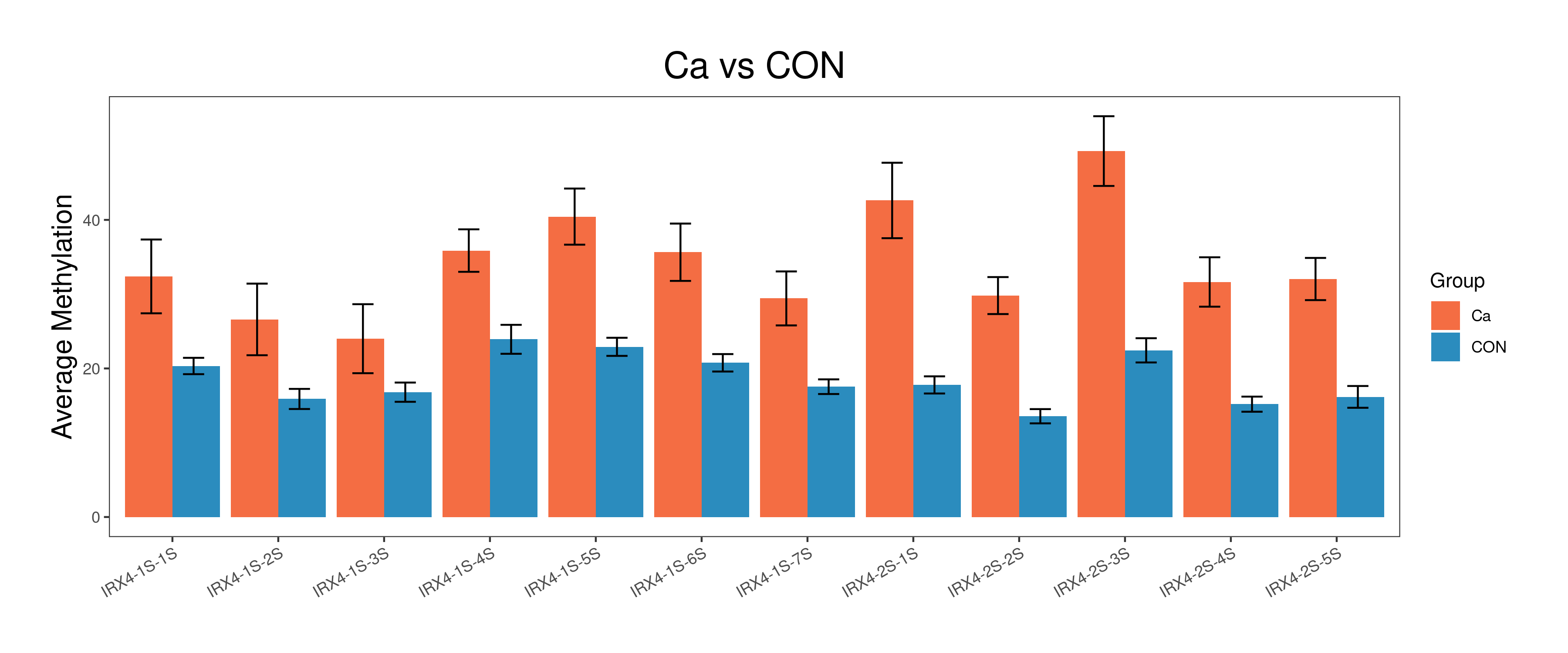

Supplement: Supplementary file 2 [file DataSheet2.zip › Analysis of Methylated Phosphorylation Data(Ca.VS.CON)/ALL_barplot.Ca.VS.CON.png]

# Ca vs CON

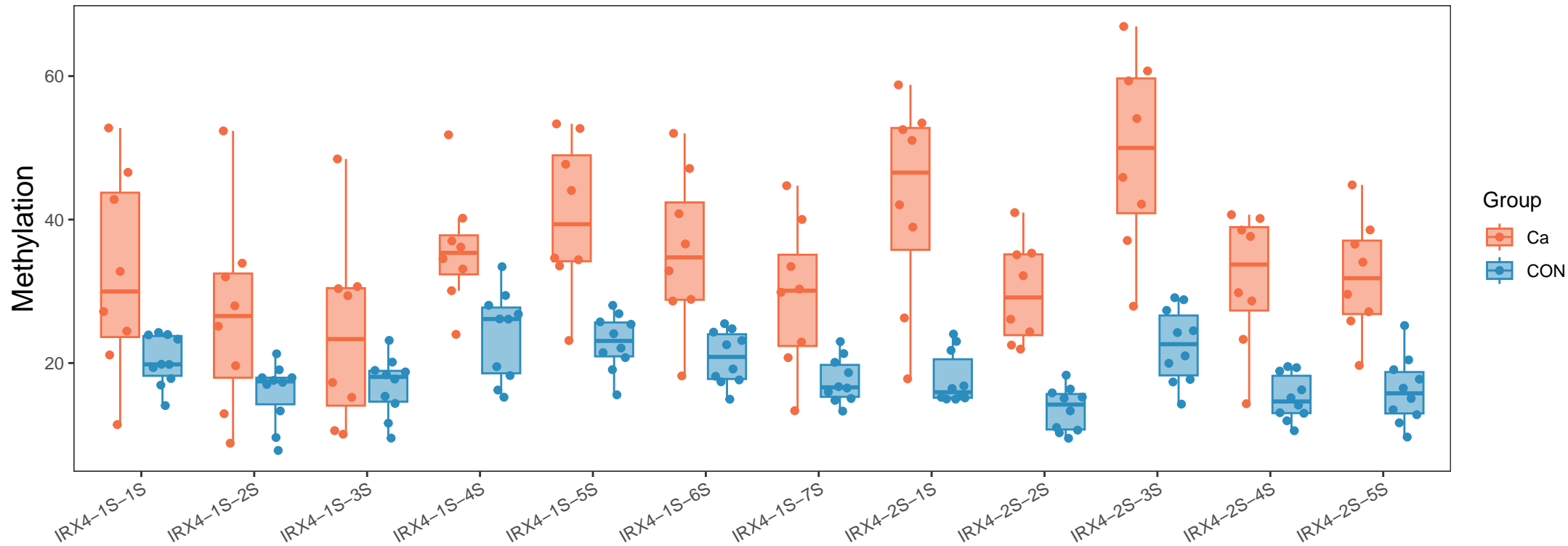

Supplement: Supplementary file 2 [file DataSheet2.zip › Analysis of Methylated Phosphorylation Data(Ca.VS.CON)/ALL_boxplot.Ca.VS.CON.pdf]

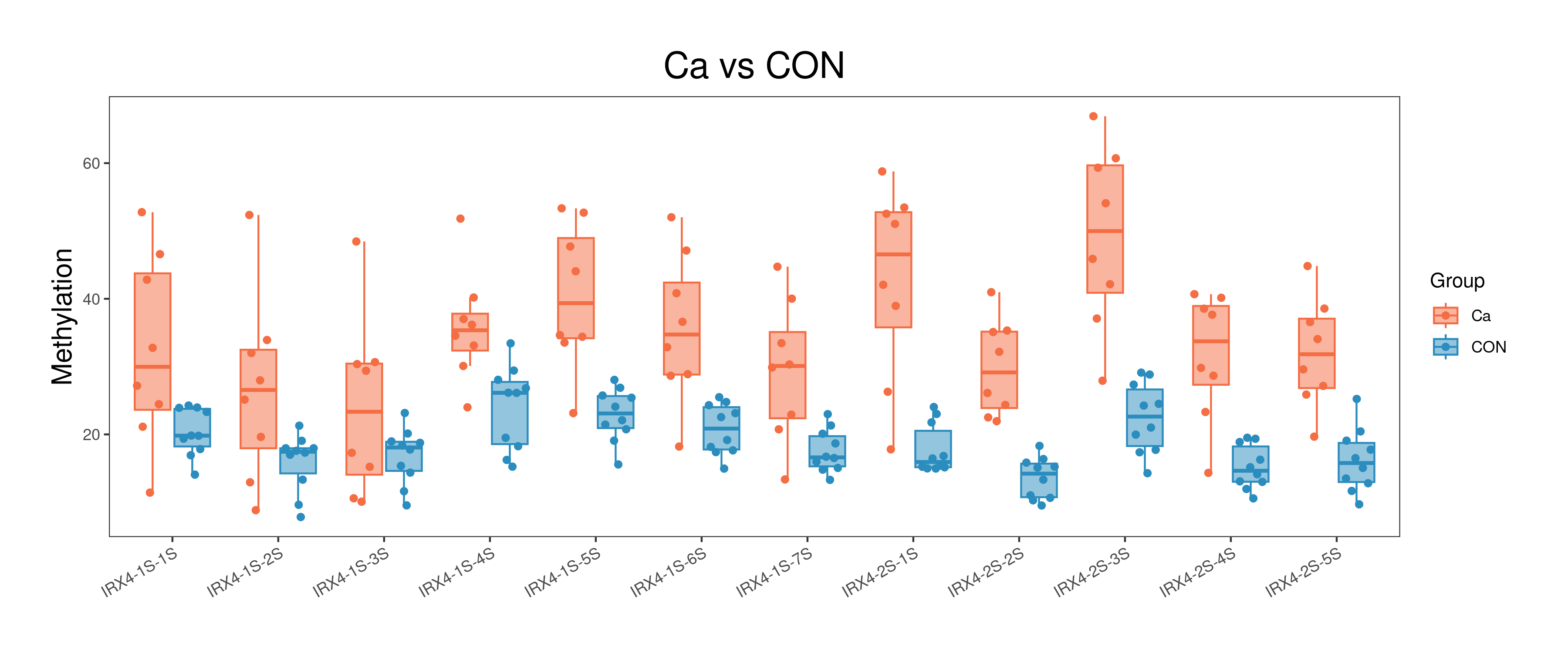

Supplement: Supplementary file 2 [file DataSheet2.zip › Analysis of Methylated Phosphorylation Data(Ca.VS.CON)/ALL_boxplot.Ca.VS.CON.png]

# IRX4-1S-1S

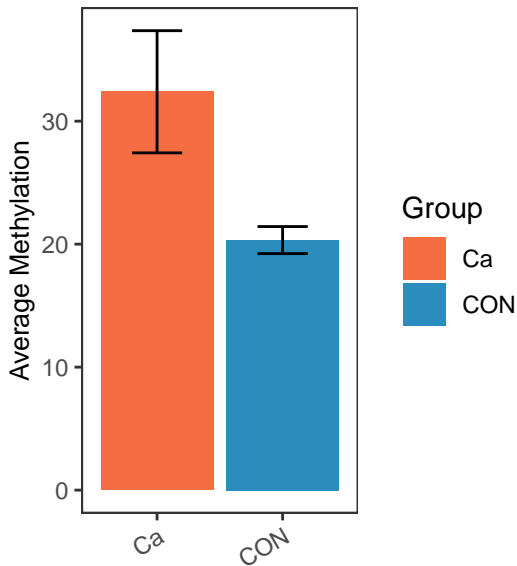

Supplement: Supplementary file 2 [file DataSheet2.zip › Analysis of Methylated Phosphorylation Data(Ca.VS.CON)/barplot/IRX4-1S-1S_barplot.pdf]

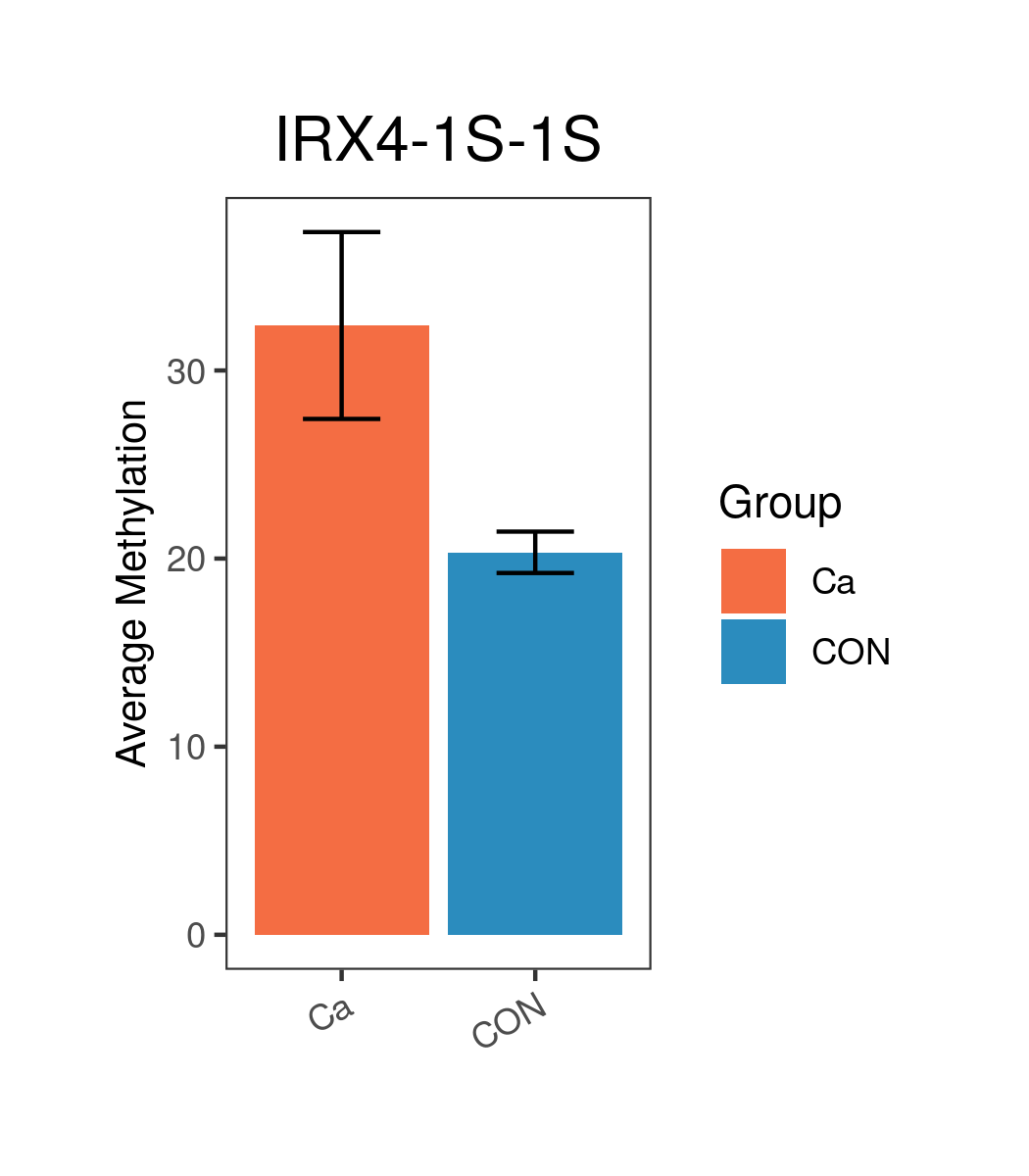

Supplement: Supplementary file 2 [file DataSheet2.zip › Analysis of Methylated Phosphorylation Data(Ca.VS.CON)/barplot/IRX4-1S-1S_barplot.png]

# IRX4-1S-2S

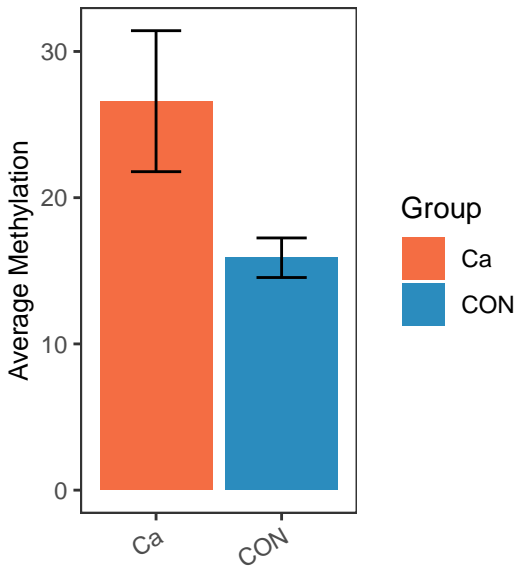

Supplement: Supplementary file 2 [file DataSheet2.zip › Analysis of Methylated Phosphorylation Data(Ca.VS.CON)/barplot/IRX4-1S-2S_barplot.pdf]

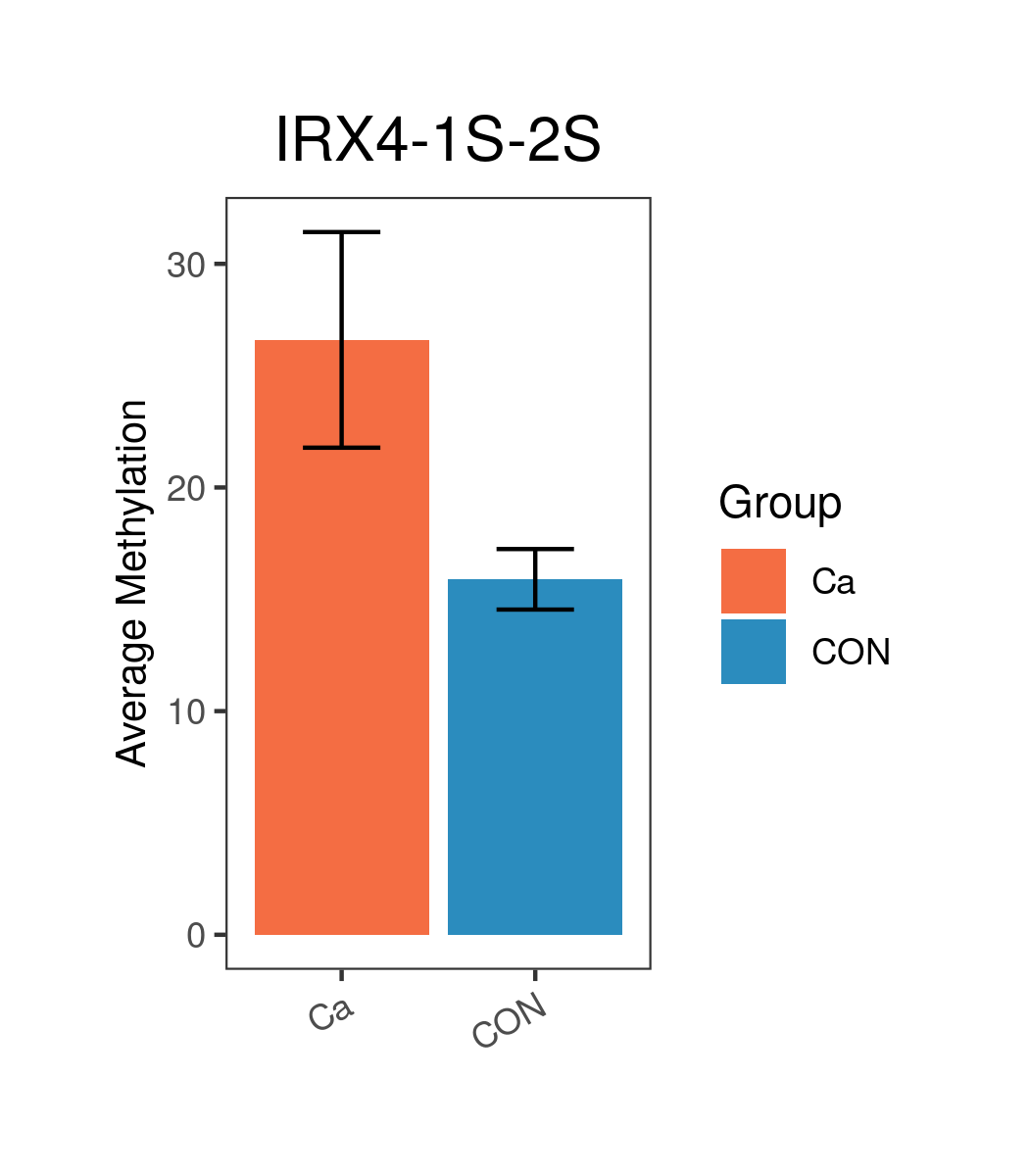

Supplement: Supplementary file 2 [file DataSheet2.zip › Analysis of Methylated Phosphorylation Data(Ca.VS.CON)/barplot/IRX4-1S-2S_barplot.png]

# IRX4-1S-3S

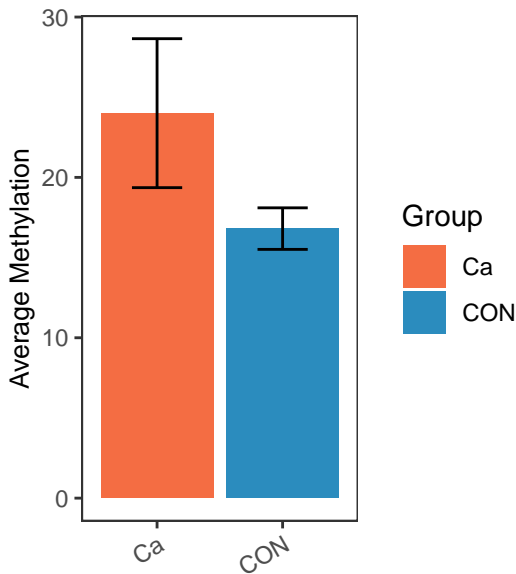

Supplement: Supplementary file 2 [file DataSheet2.zip › Analysis of Methylated Phosphorylation Data(Ca.VS.CON)/barplot/IRX4-1S-3S_barplot.pdf]

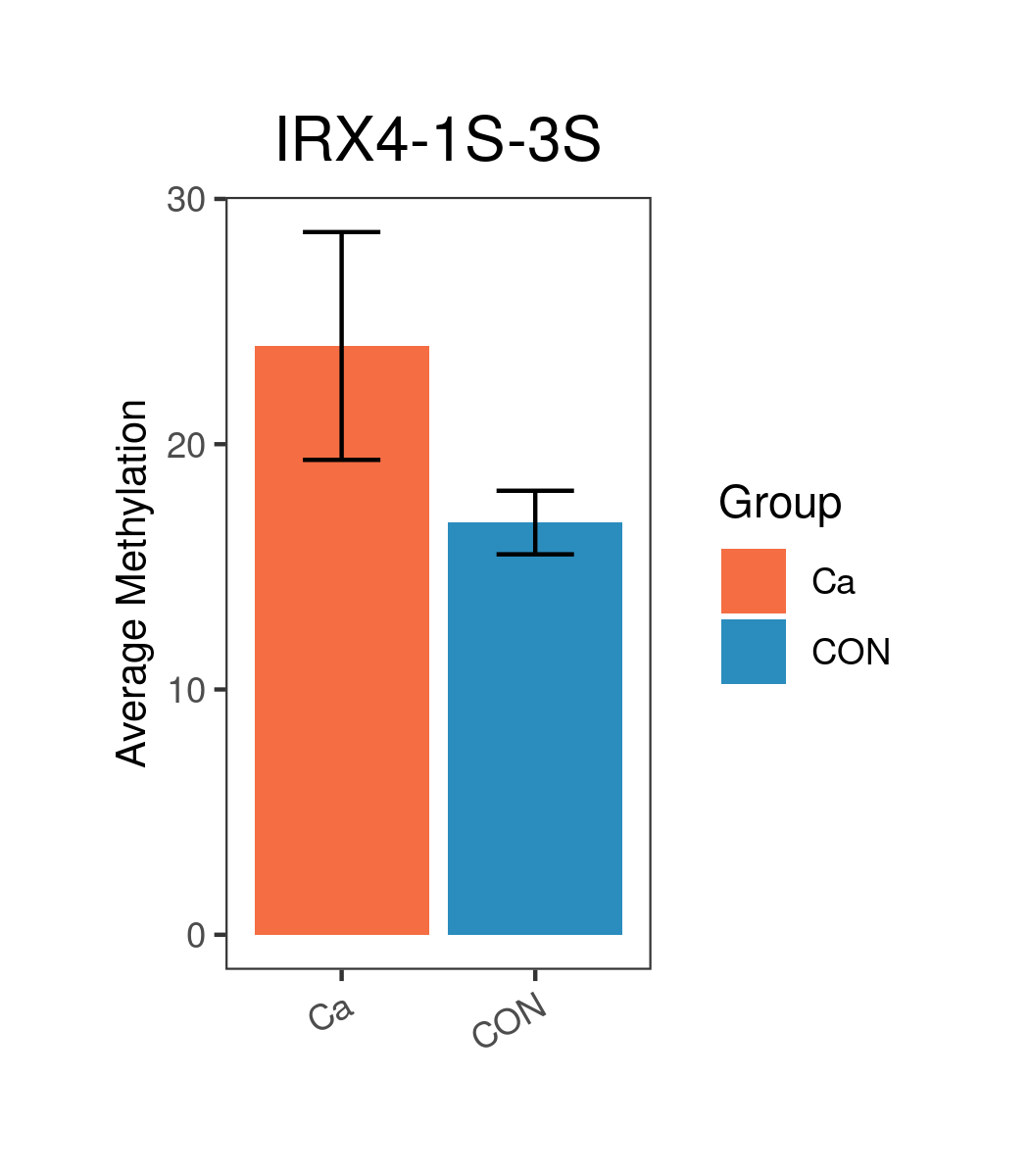

Supplement: Supplementary file 2 [file DataSheet2.zip › Analysis of Methylated Phosphorylation Data(Ca.VS.CON)/barplot/IRX4-1S-3S_barplot.png]

# IRX4-1S-4S

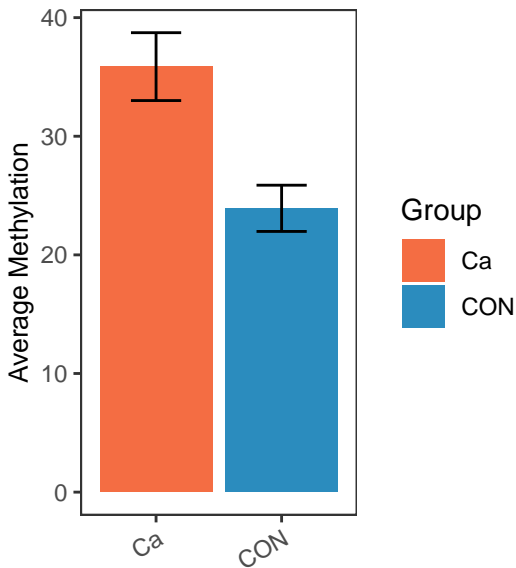

Supplement: Supplementary file 2 [file DataSheet2.zip › Analysis of Methylated Phosphorylation Data(Ca.VS.CON)/barplot/IRX4-1S-4S_barplot.pdf]

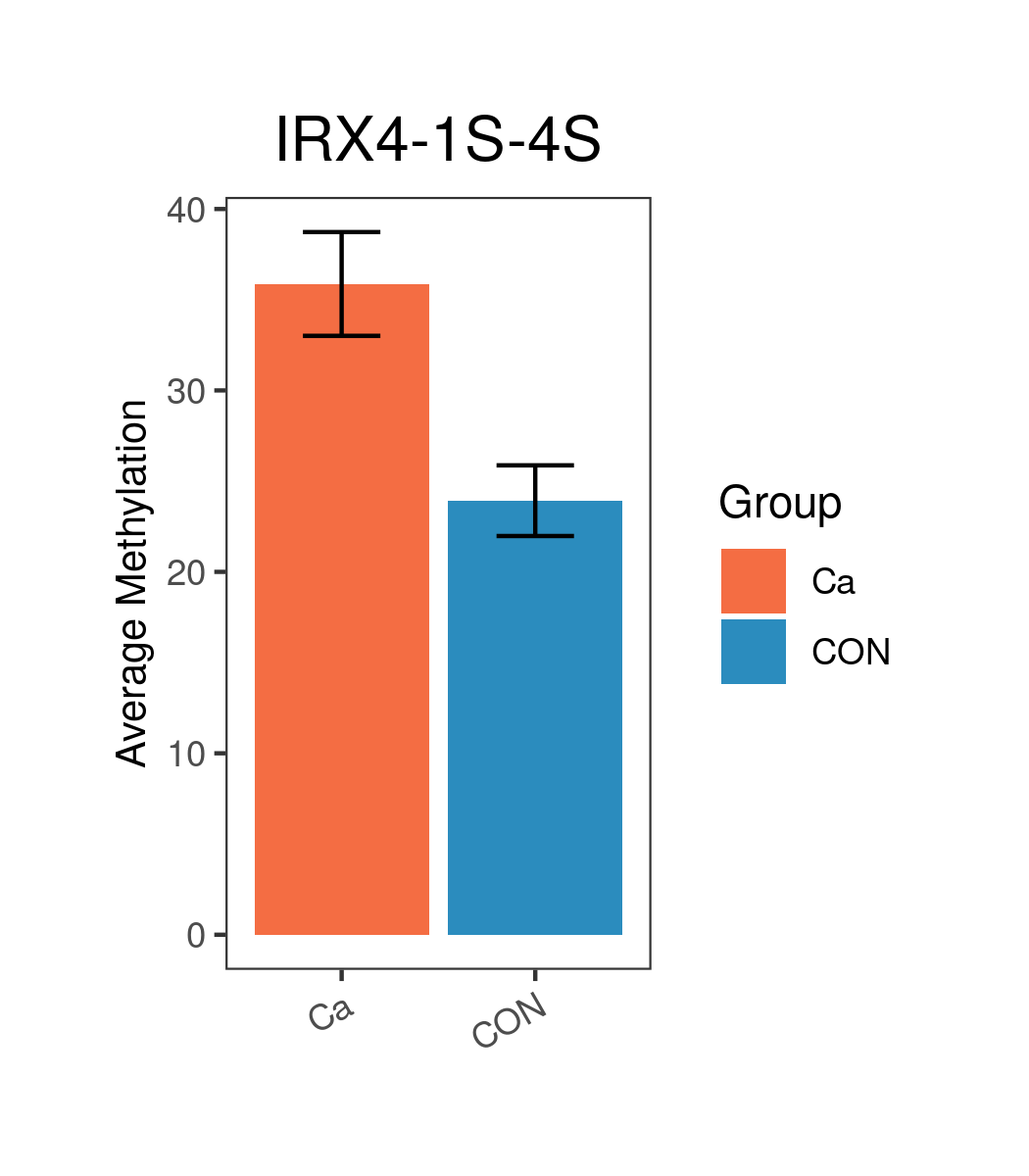

Supplement: Supplementary file 2 [file DataSheet2.zip › Analysis of Methylated Phosphorylation Data(Ca.VS.CON)/barplot/IRX4-1S-4S_barplot.png]

# IRX4-1S-5S

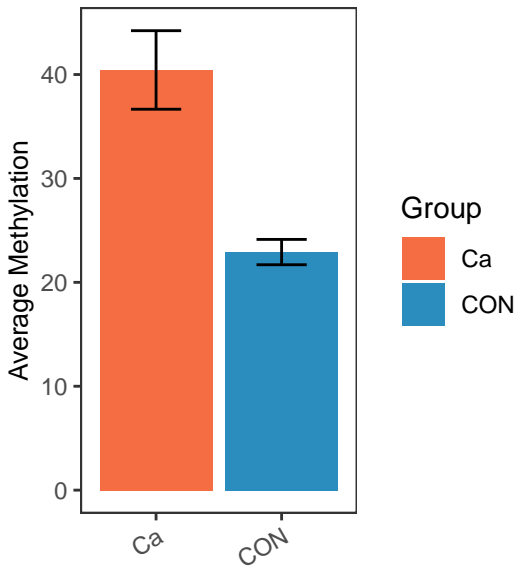

Supplement: Supplementary file 2 [file DataSheet2.zip › Analysis of Methylated Phosphorylation Data(Ca.VS.CON)/barplot/IRX4-1S-5S_barplot.pdf]

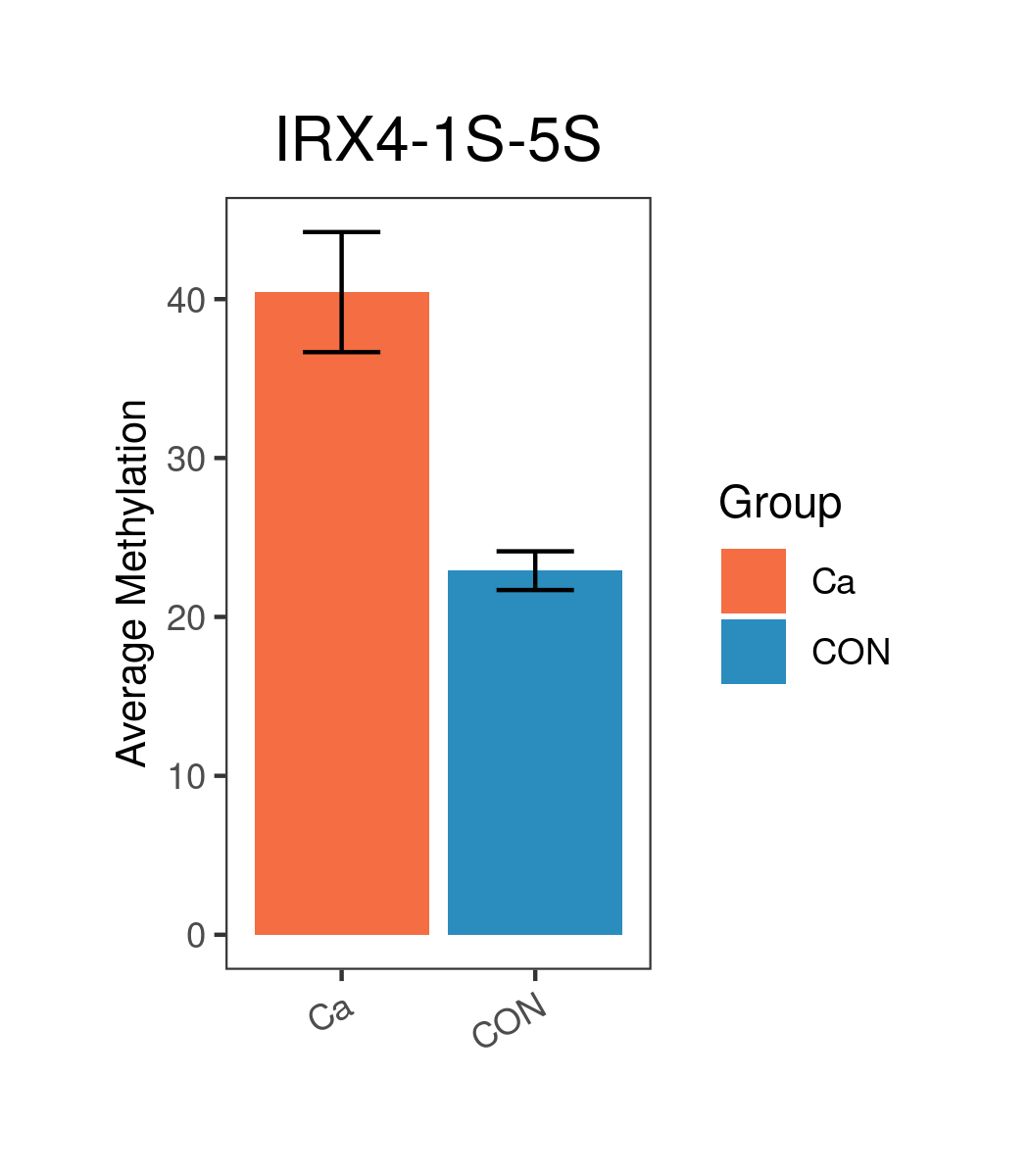

Supplement: Supplementary file 2 [file DataSheet2.zip › Analysis of Methylated Phosphorylation Data(Ca.VS.CON)/barplot/IRX4-1S-5S_barplot.png]

# IRX4-1S-6S

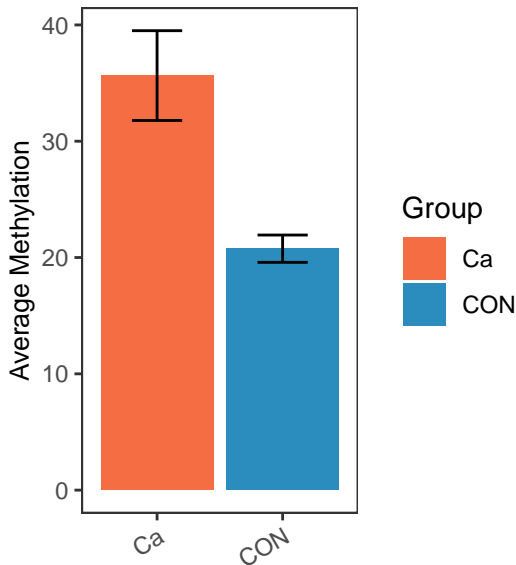

Supplement: Supplementary file 2 [file DataSheet2.zip › Analysis of Methylated Phosphorylation Data(Ca.VS.CON)/barplot/IRX4-1S-6S_barplot.pdf]

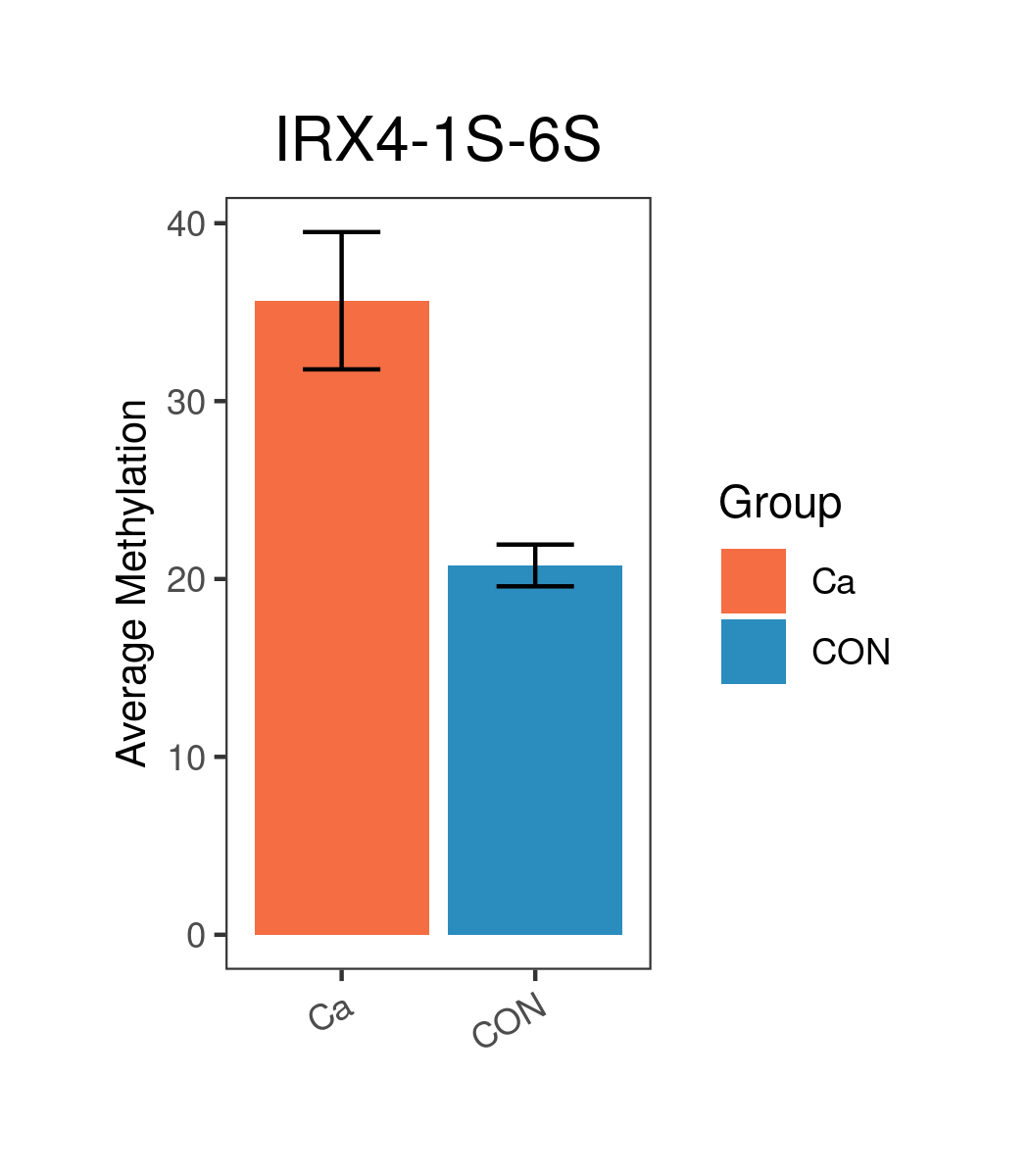

Supplement: Supplementary file 2 [file DataSheet2.zip › Analysis of Methylated Phosphorylation Data(Ca.VS.CON)/barplot/IRX4-1S-6S_barplot.png]

# IRX4-1S-7S

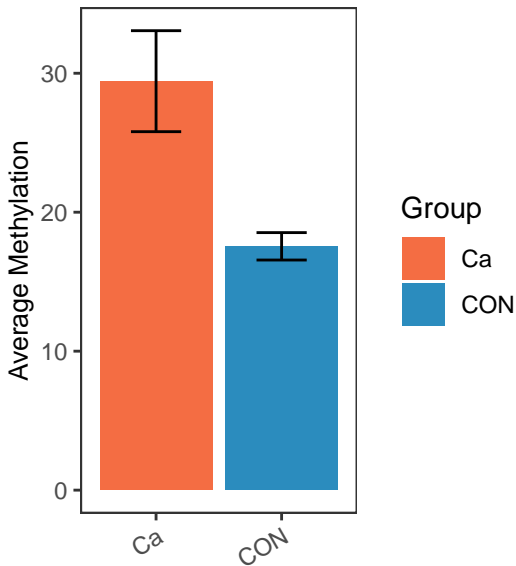

Supplement: Supplementary file 2 [file DataSheet2.zip › Analysis of Methylated Phosphorylation Data(Ca.VS.CON)/barplot/IRX4-1S-7S_barplot.pdf]

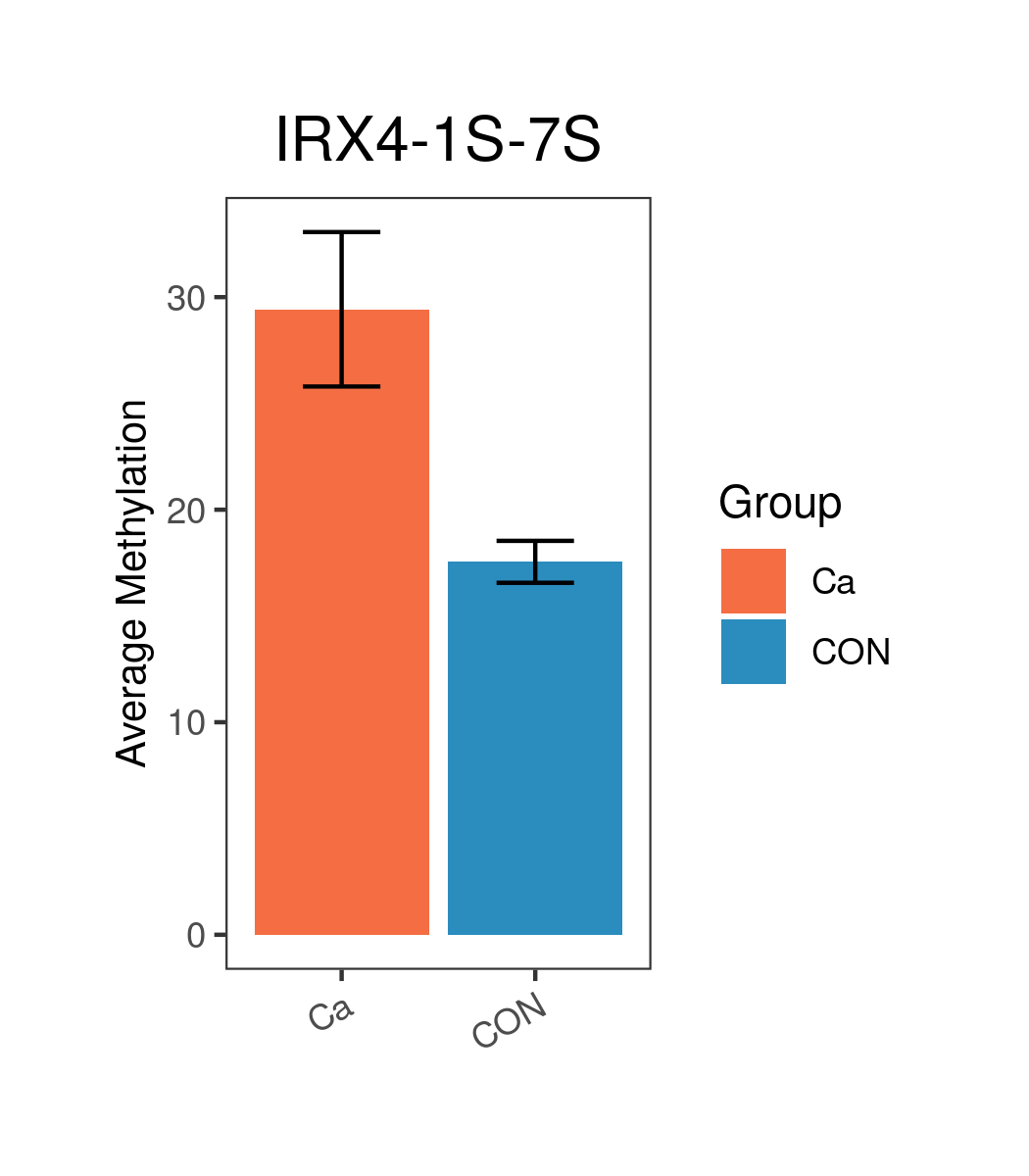

Supplement: Supplementary file 2 [file DataSheet2.zip › Analysis of Methylated Phosphorylation Data(Ca.VS.CON)/barplot/IRX4-1S-7S_barplot.png]

# IRX4-2S-1S

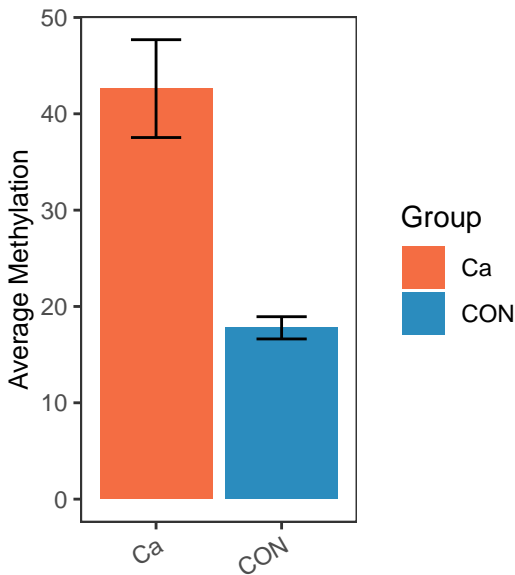

Supplement: Supplementary file 2 [file DataSheet2.zip › Analysis of Methylated Phosphorylation Data(Ca.VS.CON)/barplot/IRX4-2S-1S_barplot.pdf]

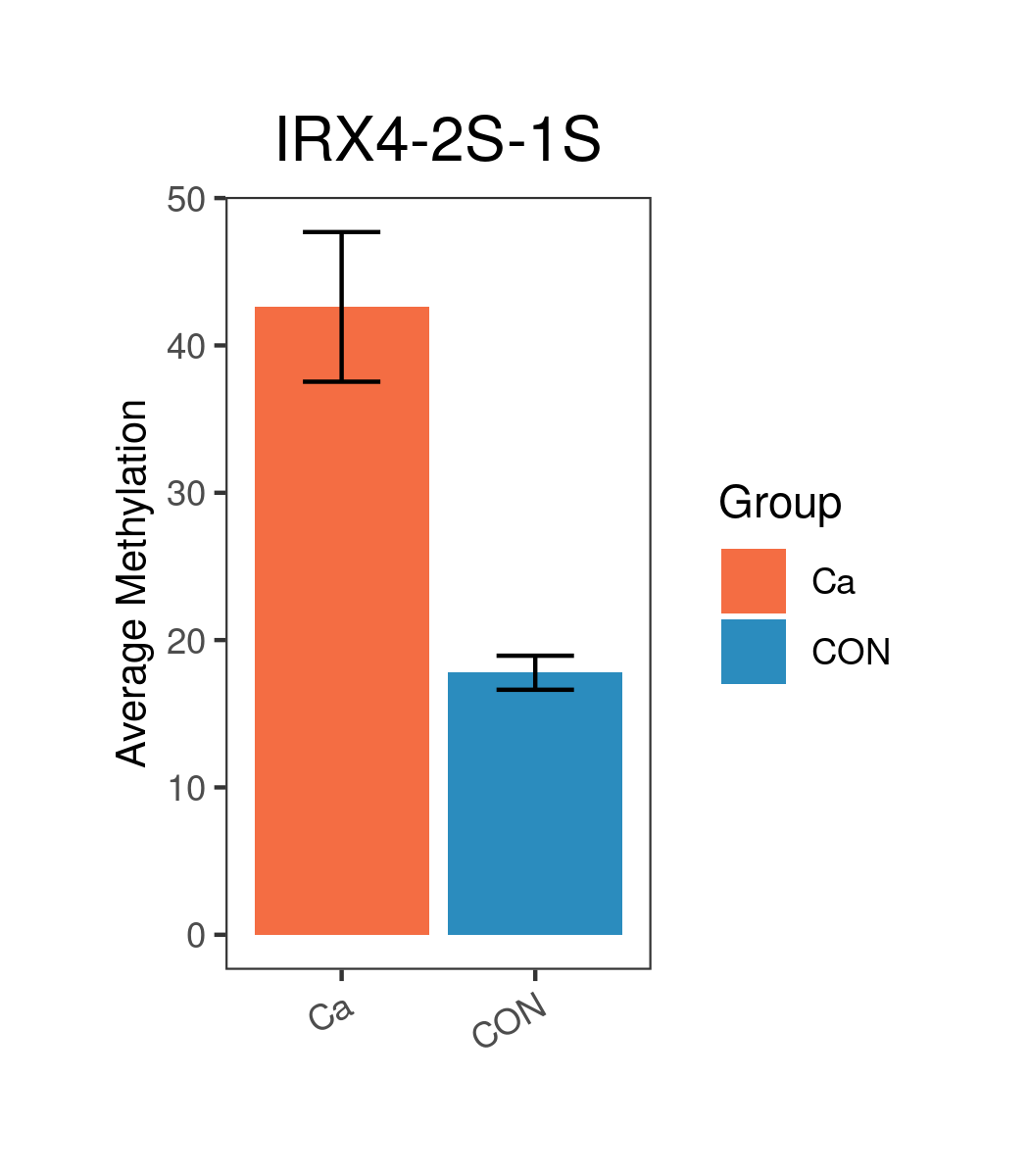

Supplement: Supplementary file 2 [file DataSheet2.zip › Analysis of Methylated Phosphorylation Data(Ca.VS.CON)/barplot/IRX4-2S-1S_barplot.png]

# IRX4-2S-2S

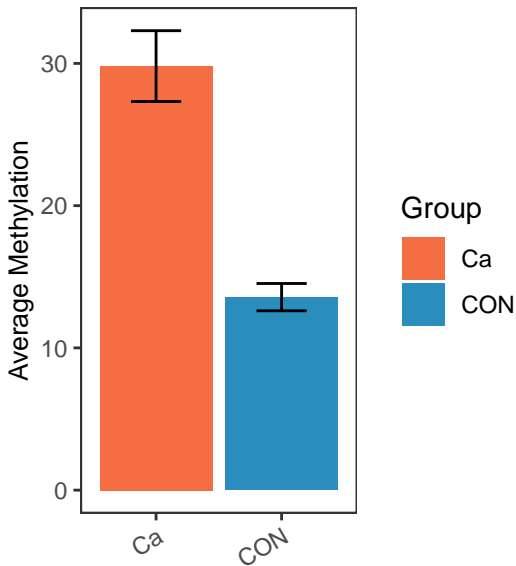

Supplement: Supplementary file 2 [file DataSheet2.zip › Analysis of Methylated Phosphorylation Data(Ca.VS.CON)/barplot/IRX4-2S-2S_barplot.pdf]

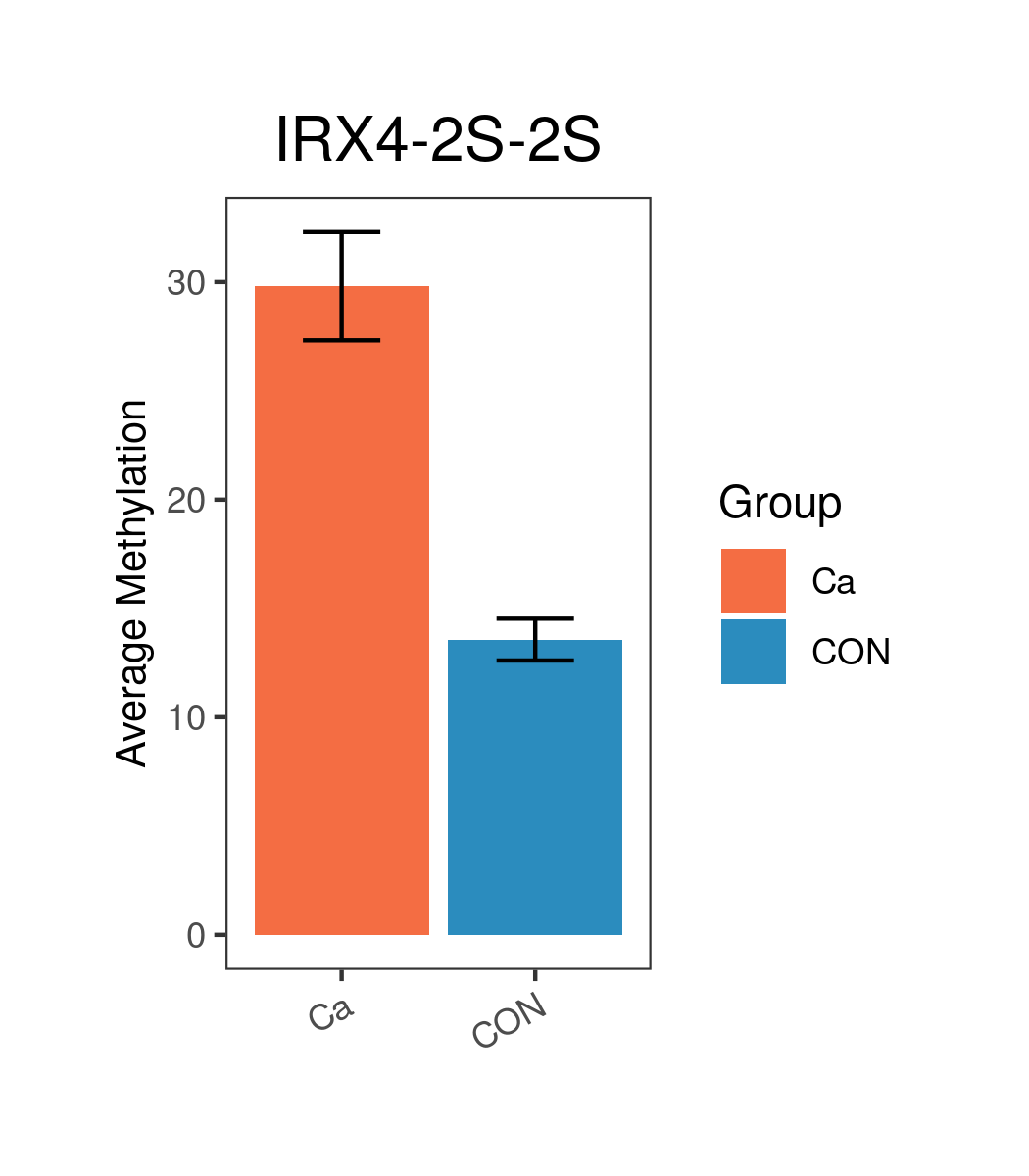

Supplement: Supplementary file 2 [file DataSheet2.zip › Analysis of Methylated Phosphorylation Data(Ca.VS.CON)/barplot/IRX4-2S-2S_barplot.png]

# IRX4-2S-3S

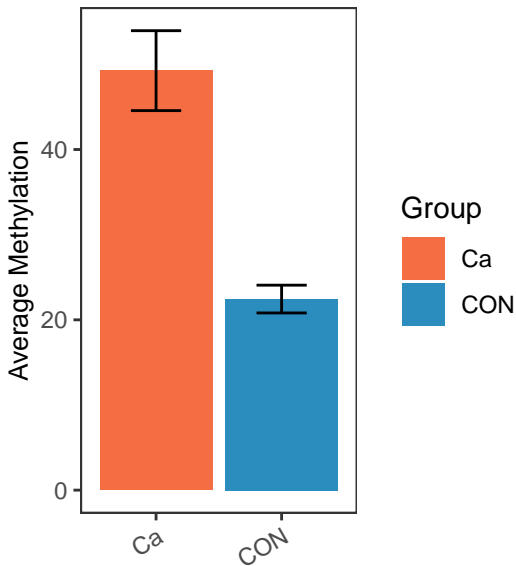

Supplement: Supplementary file 2 [file DataSheet2.zip › Analysis of Methylated Phosphorylation Data(Ca.VS.CON)/barplot/IRX4-2S-3S_barplot.pdf]

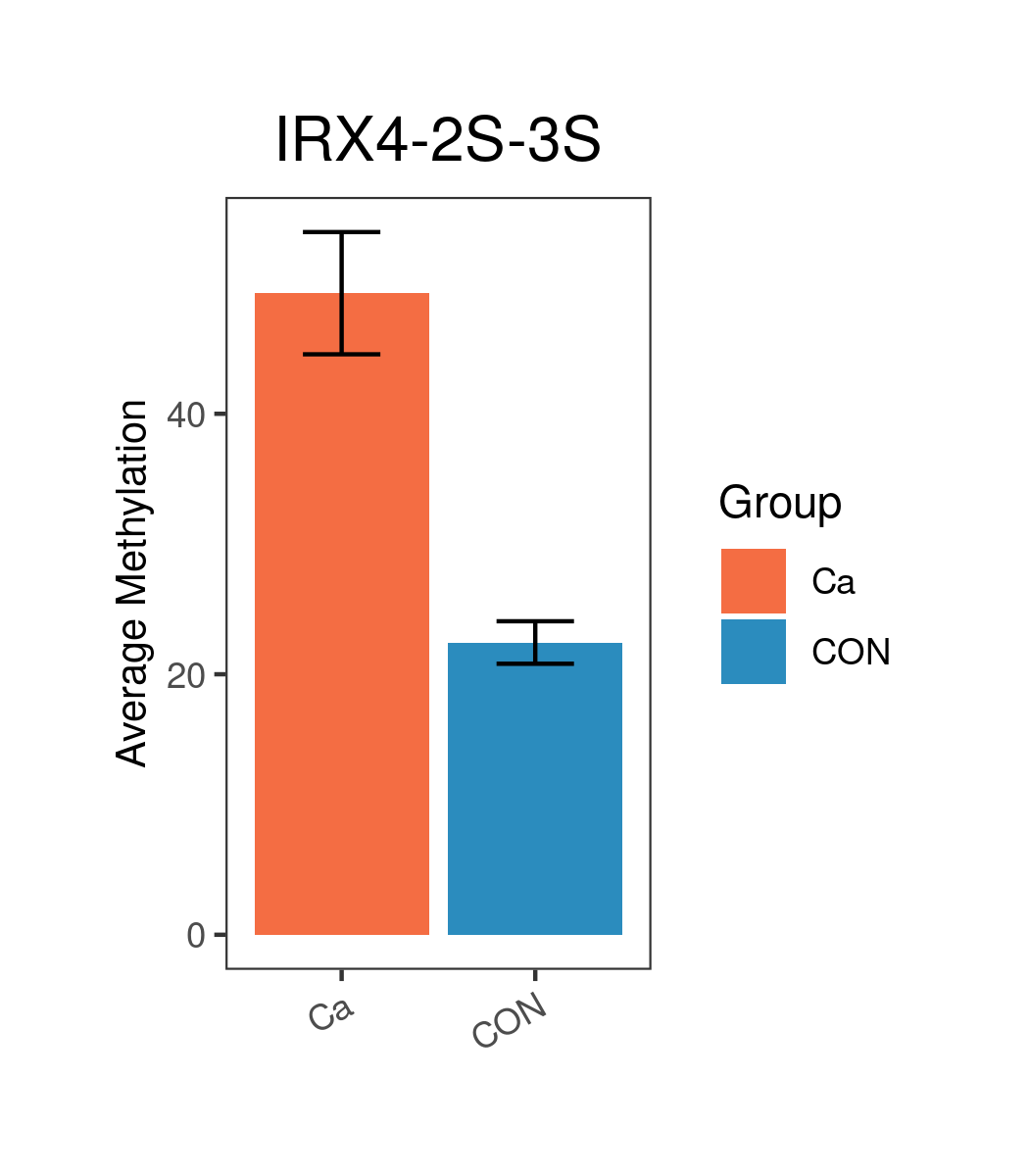

Supplement: Supplementary file 2 [file DataSheet2.zip › Analysis of Methylated Phosphorylation Data(Ca.VS.CON)/barplot/IRX4-2S-3S_barplot.png]

# IRX4-2S-4S

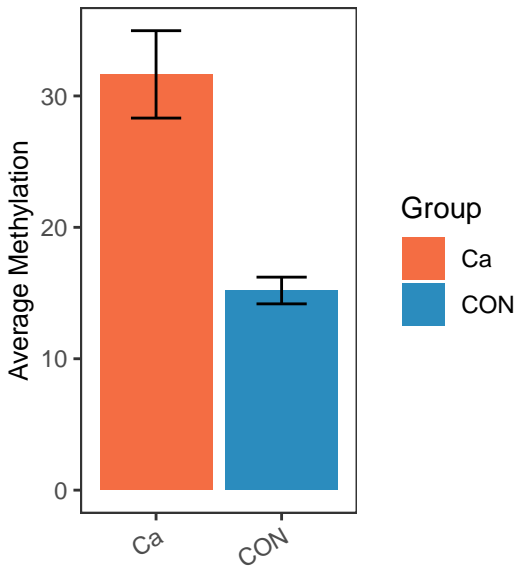

Supplement: Supplementary file 2 [file DataSheet2.zip › Analysis of Methylated Phosphorylation Data(Ca.VS.CON)/barplot/IRX4-2S-4S_barplot.pdf]

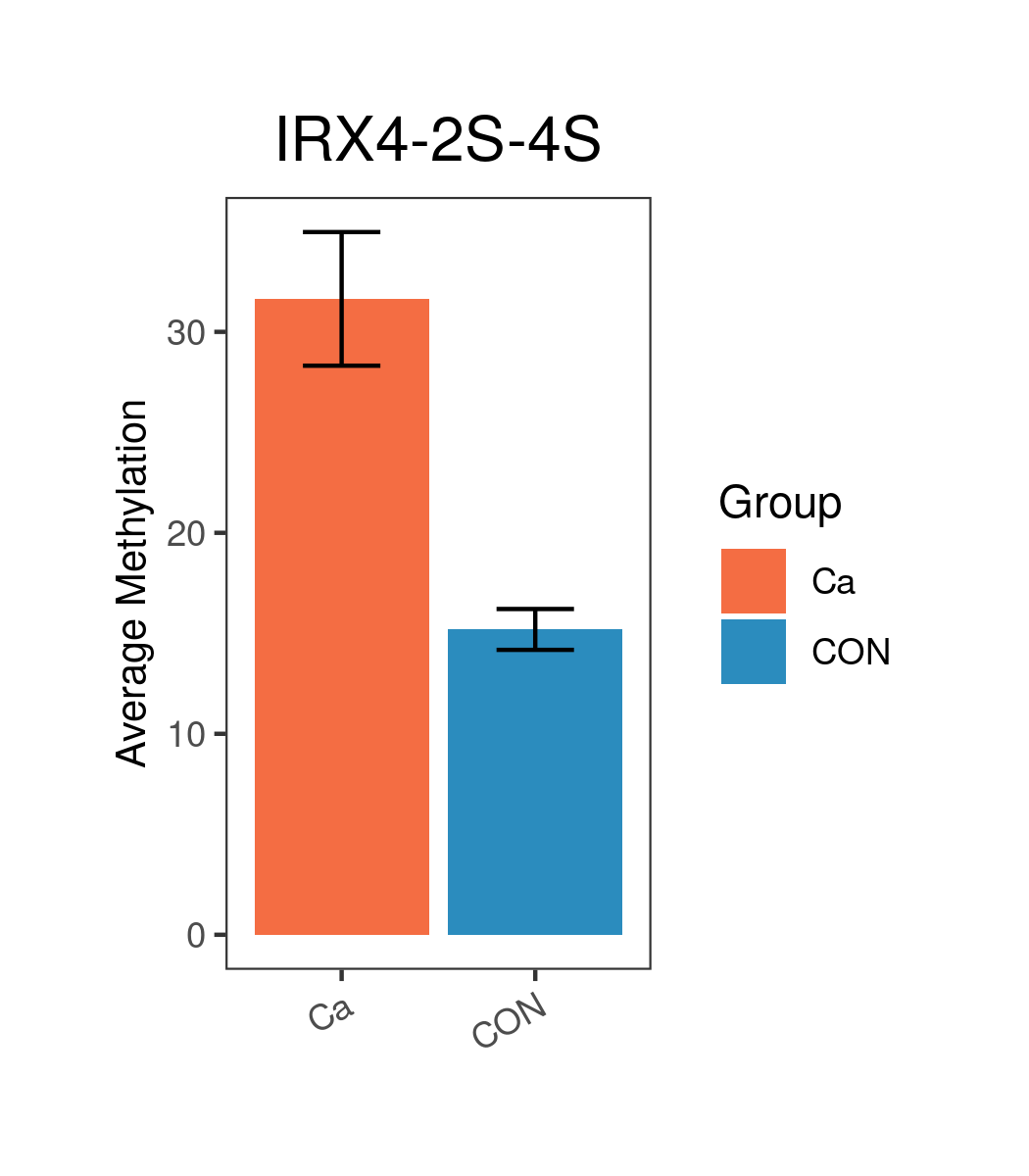

Supplement: Supplementary file 2 [file DataSheet2.zip › Analysis of Methylated Phosphorylation Data(Ca.VS.CON)/barplot/IRX4-2S-4S_barplot.png]

# IRX4-2S-5S

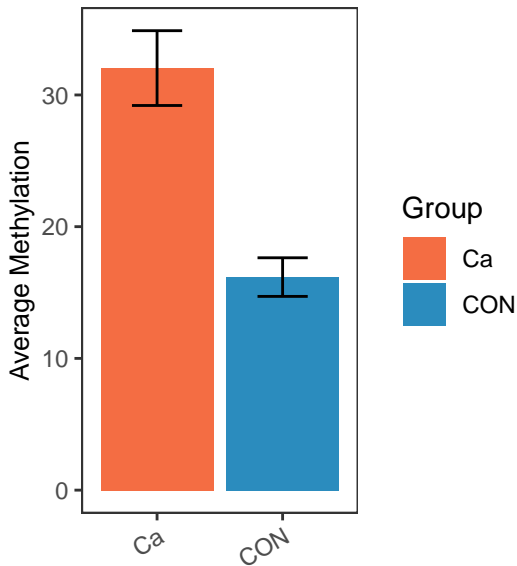

Supplement: Supplementary file 2 [file DataSheet2.zip › Analysis of Methylated Phosphorylation Data(Ca.VS.CON)/barplot/IRX4-2S-5S_barplot.pdf]

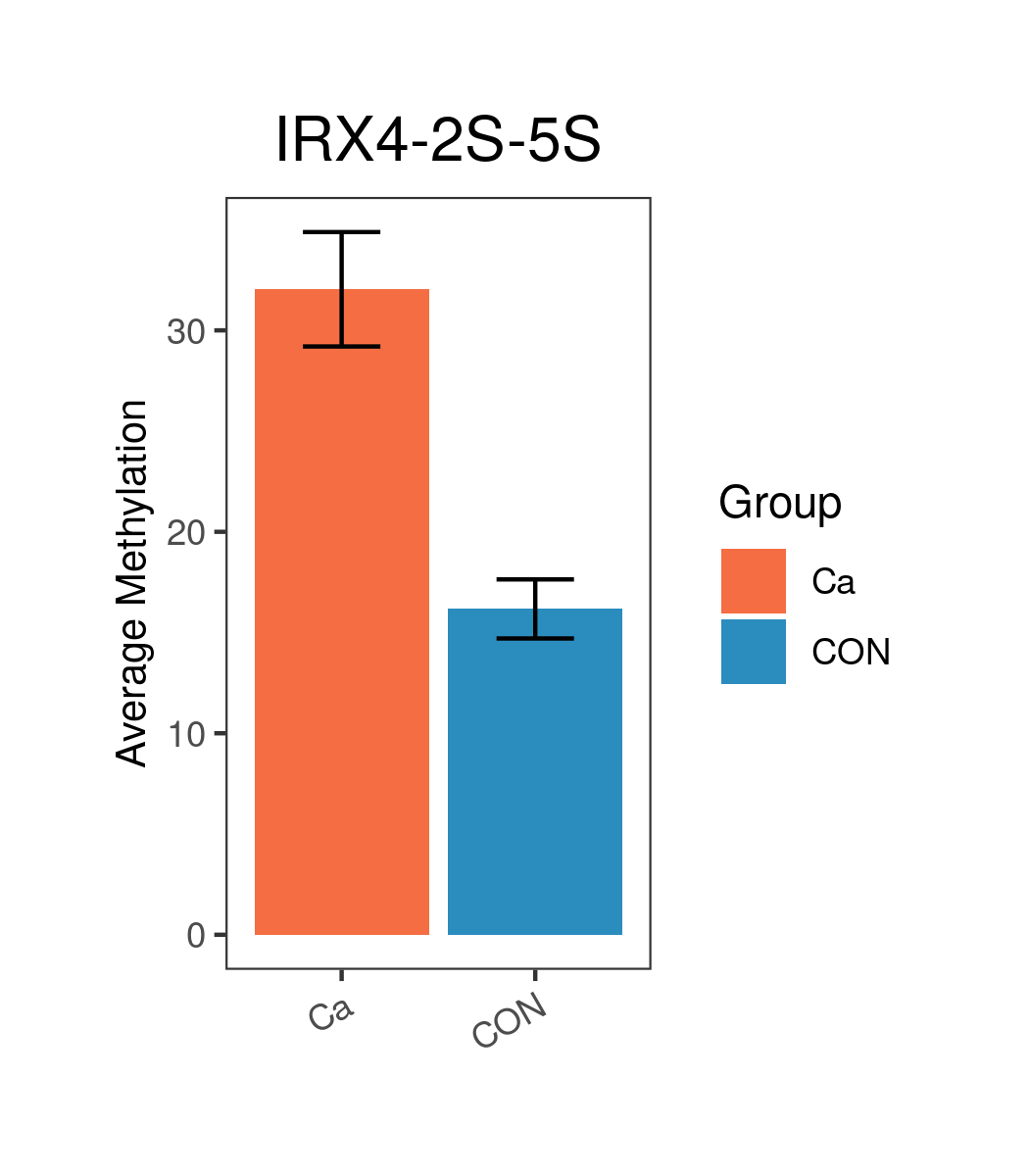

Supplement: Supplementary file 2 [file DataSheet2.zip › Analysis of Methylated Phosphorylation Data(Ca.VS.CON)/barplot/IRX4-2S-5S_barplot.png]

# IRX4-1S-1S

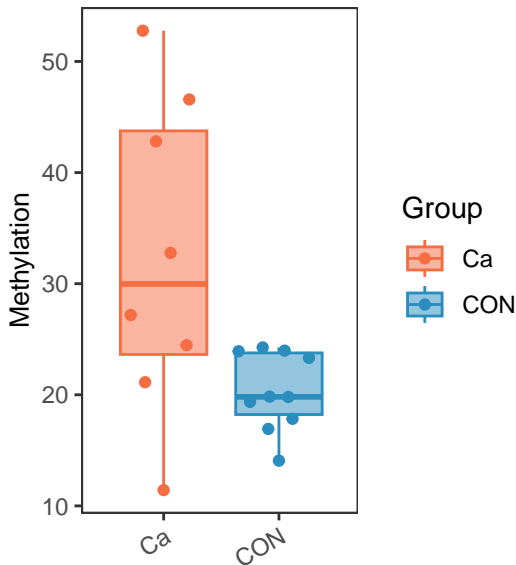

Supplement: Supplementary file 2 [file DataSheet2.zip › Analysis of Methylated Phosphorylation Data(Ca.VS.CON)/boxplot/IRX4-1S-1S_boxplot.pdf]

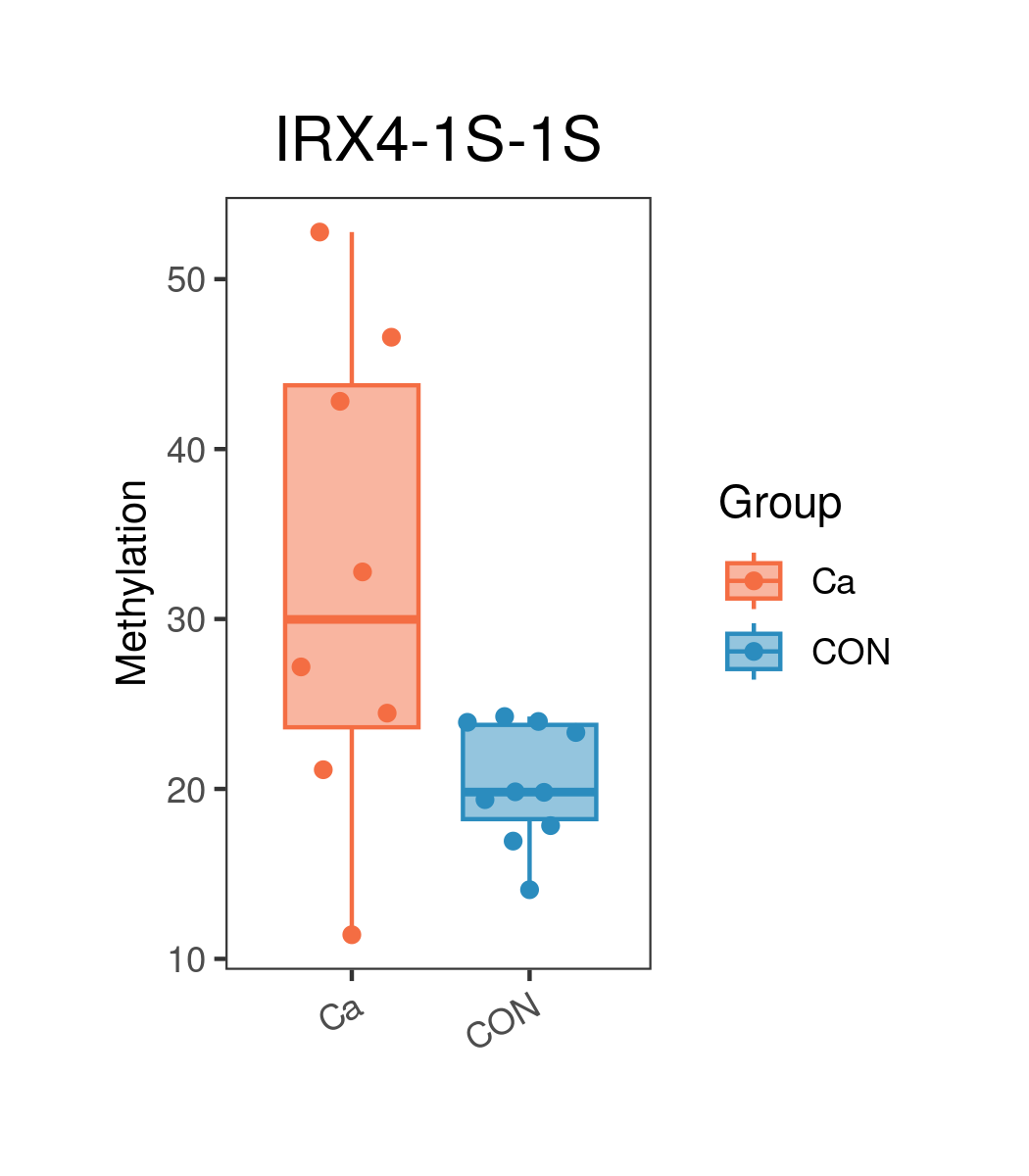

Supplement: Supplementary file 2 [file DataSheet2.zip › Analysis of Methylated Phosphorylation Data(Ca.VS.CON)/boxplot/IRX4-1S-1S_boxplot.png]

# IRX4-1S-2S

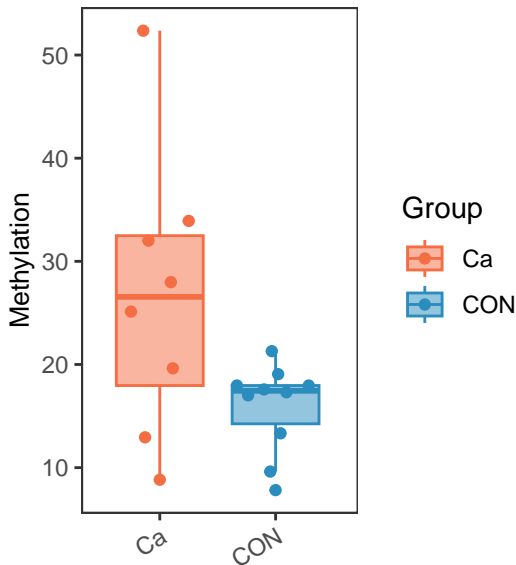

Supplement: Supplementary file 2 [file DataSheet2.zip › Analysis of Methylated Phosphorylation Data(Ca.VS.CON)/boxplot/IRX4-1S-2S_boxplot.pdf]

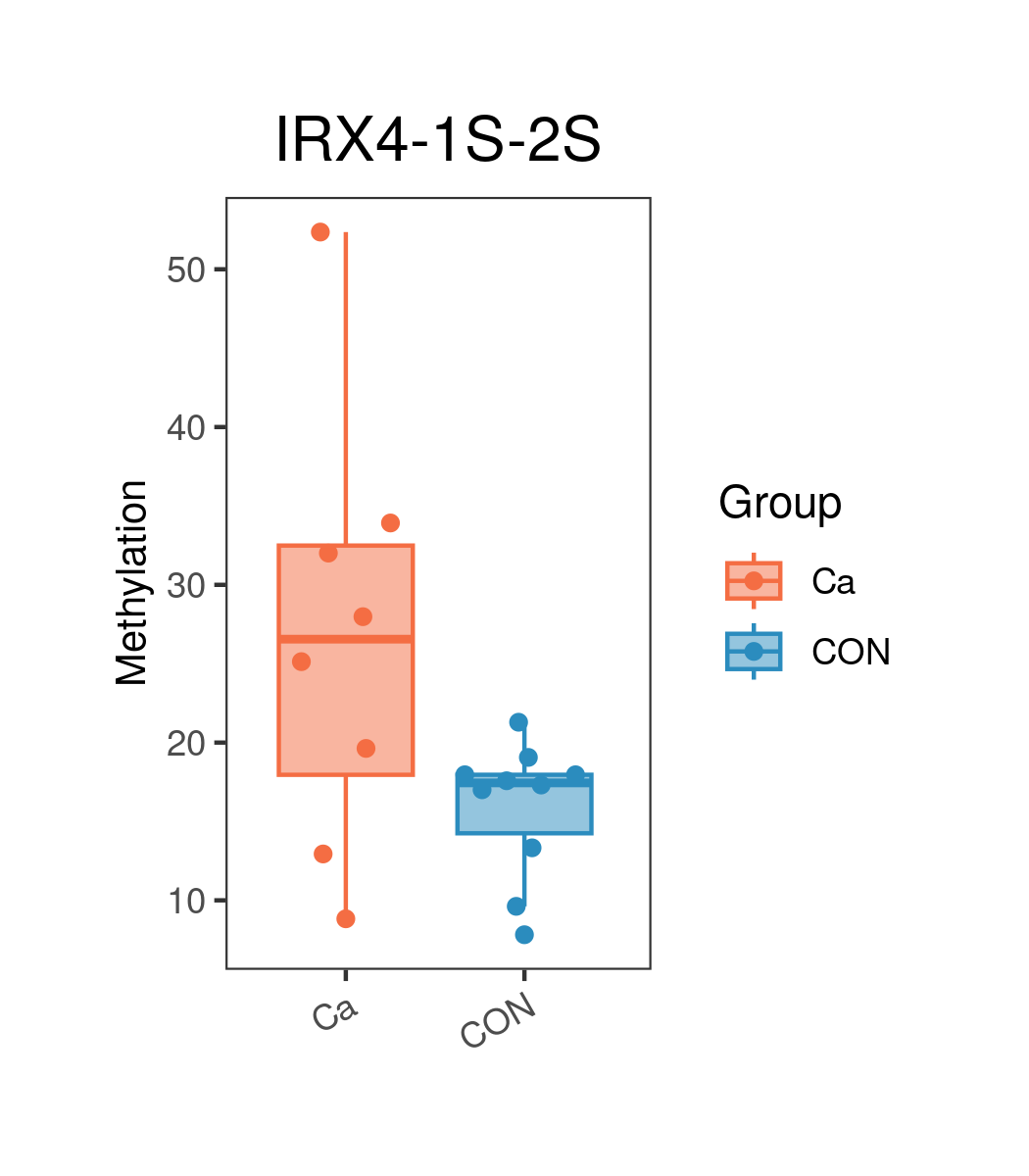

Supplement: Supplementary file 2 [file DataSheet2.zip › Analysis of Methylated Phosphorylation Data(Ca.VS.CON)/boxplot/IRX4-1S-2S_boxplot.png]

# IRX4-1S-3S

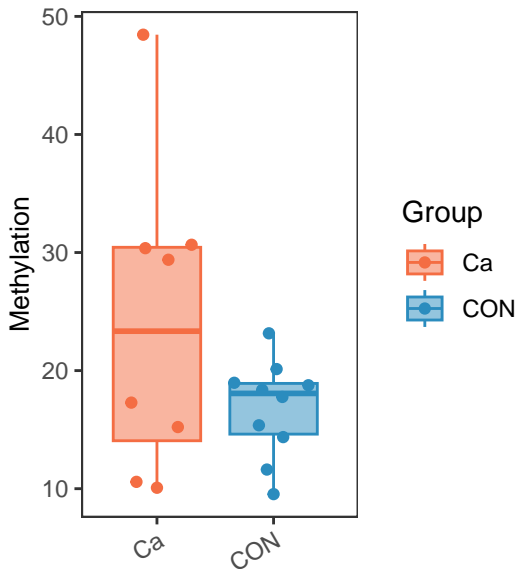

Supplement: Supplementary file 2 [file DataSheet2.zip › Analysis of Methylated Phosphorylation Data(Ca.VS.CON)/boxplot/IRX4-1S-3S_boxplot.pdf]

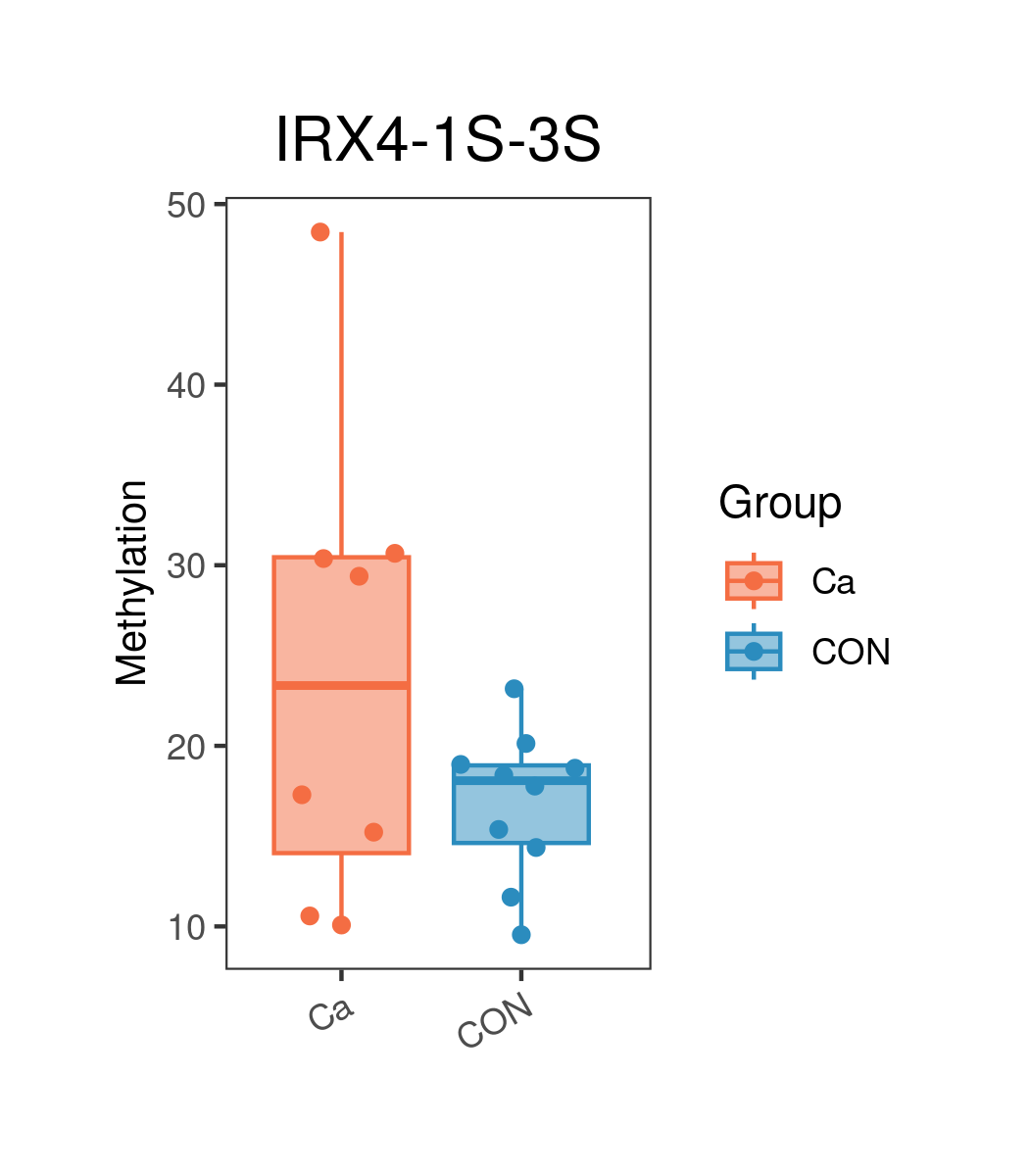

Supplement: Supplementary file 2 [file DataSheet2.zip › Analysis of Methylated Phosphorylation Data(Ca.VS.CON)/boxplot/IRX4-1S-3S_boxplot.png]

# IRX4-1S-4S

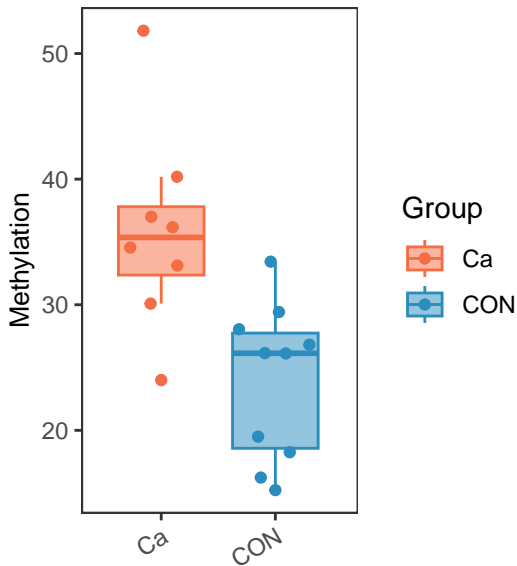

Supplement: Supplementary file 2 [file DataSheet2.zip › Analysis of Methylated Phosphorylation Data(Ca.VS.CON)/boxplot/IRX4-1S-4S_boxplot.pdf]

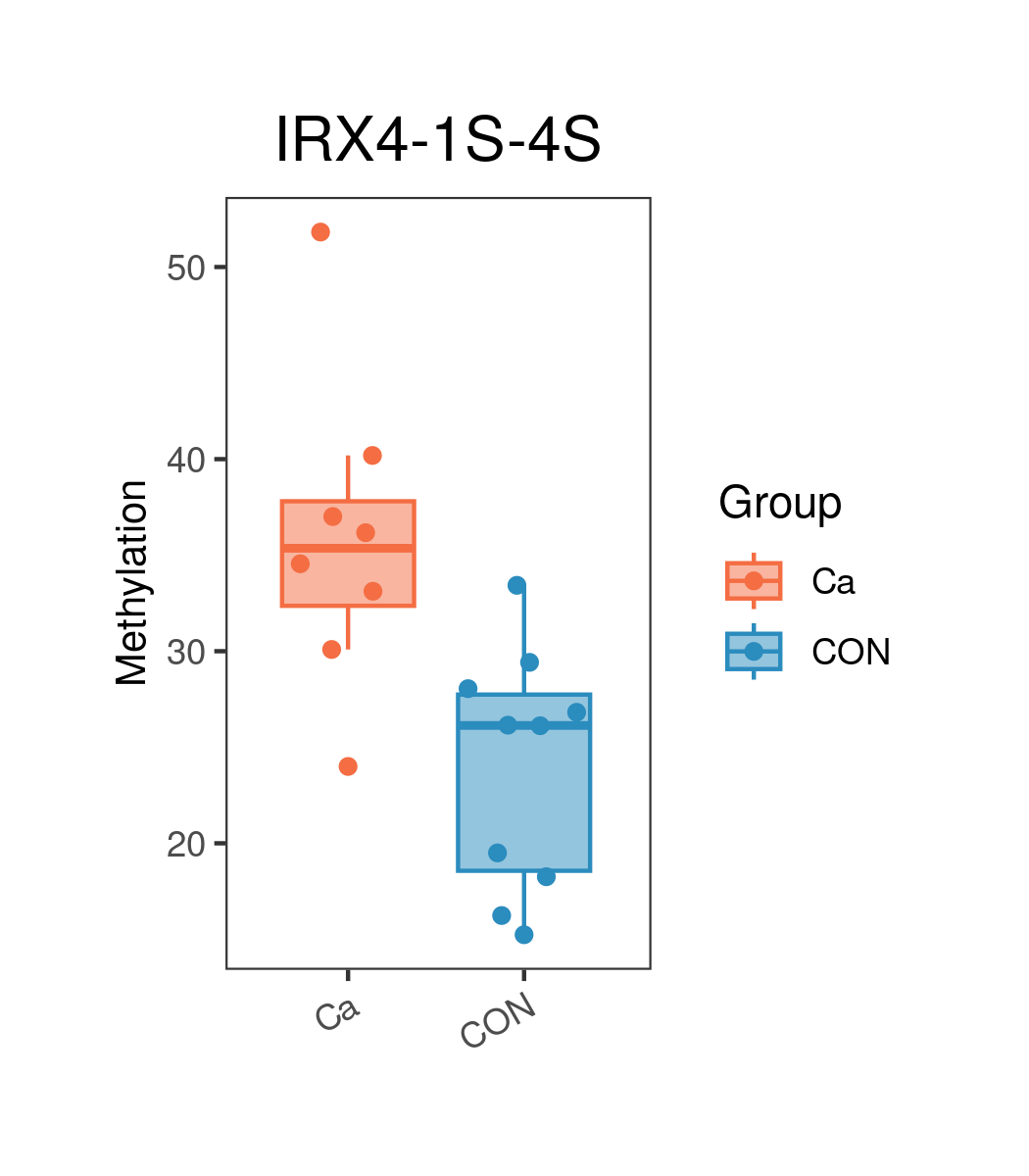

Supplement: Supplementary file 2 [file DataSheet2.zip › Analysis of Methylated Phosphorylation Data(Ca.VS.CON)/boxplot/IRX4-1S-4S_boxplot.png]

# IRX4-1S-5S

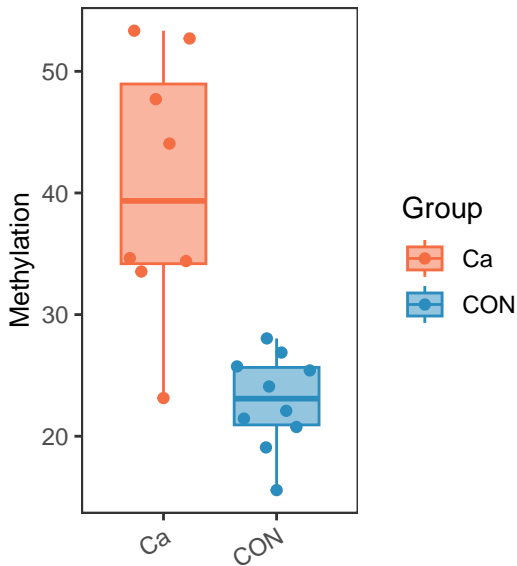

Supplement: Supplementary file 2 [file DataSheet2.zip › Analysis of Methylated Phosphorylation Data(Ca.VS.CON)/boxplot/IRX4-1S-5S_boxplot.pdf]

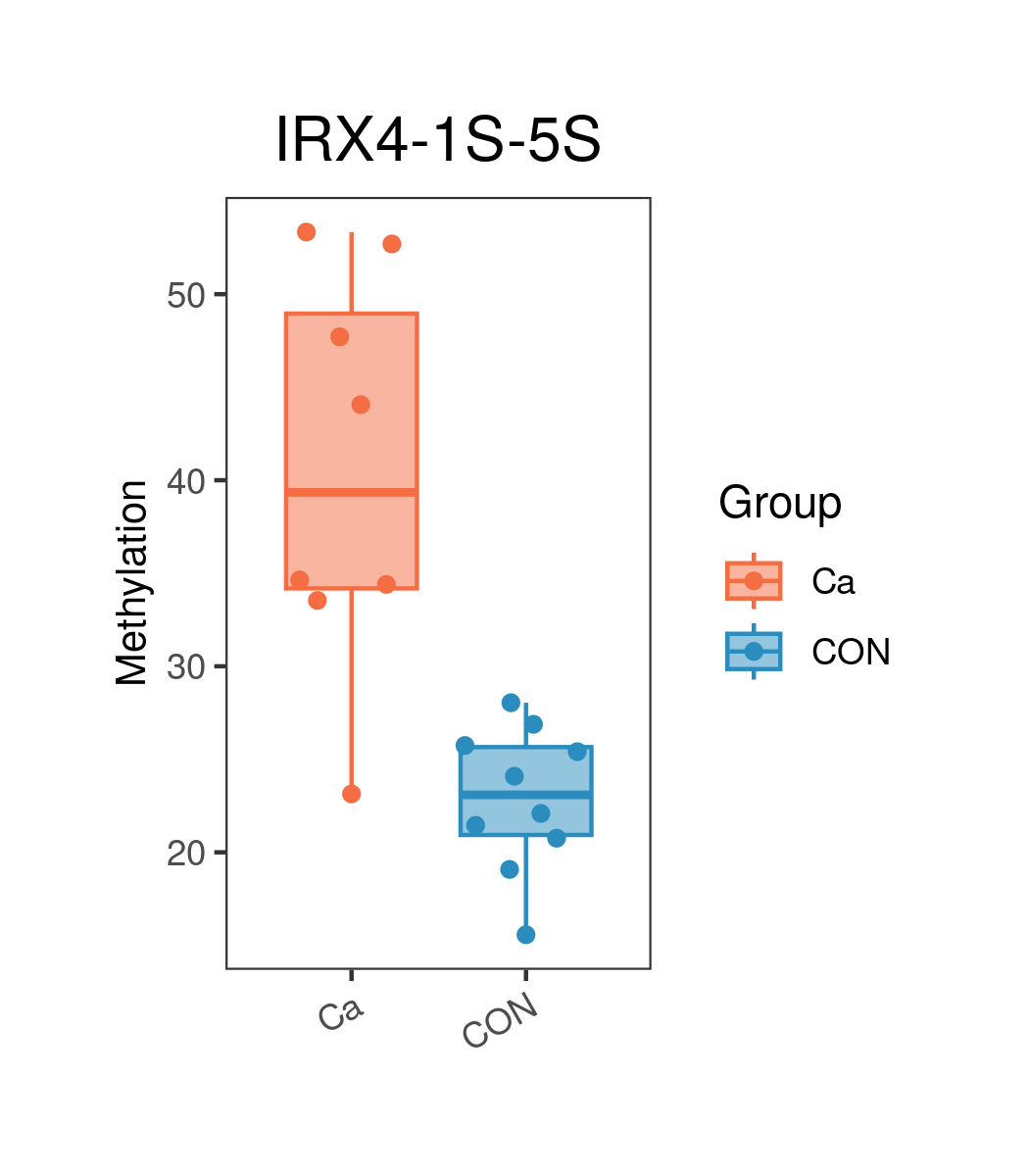

Supplement: Supplementary file 2 [file DataSheet2.zip › Analysis of Methylated Phosphorylation Data(Ca.VS.CON)/boxplot/IRX4-1S-5S_boxplot.png]

# IRX4-1S-6S

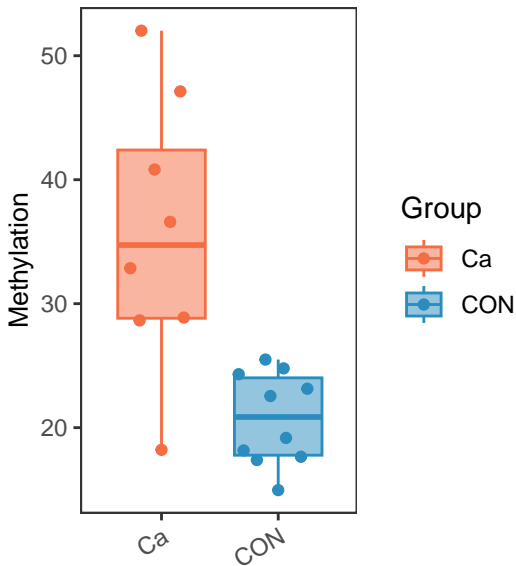

Supplement: Supplementary file 2 [file DataSheet2.zip › Analysis of Methylated Phosphorylation Data(Ca.VS.CON)/boxplot/IRX4-1S-6S_boxplot.pdf]

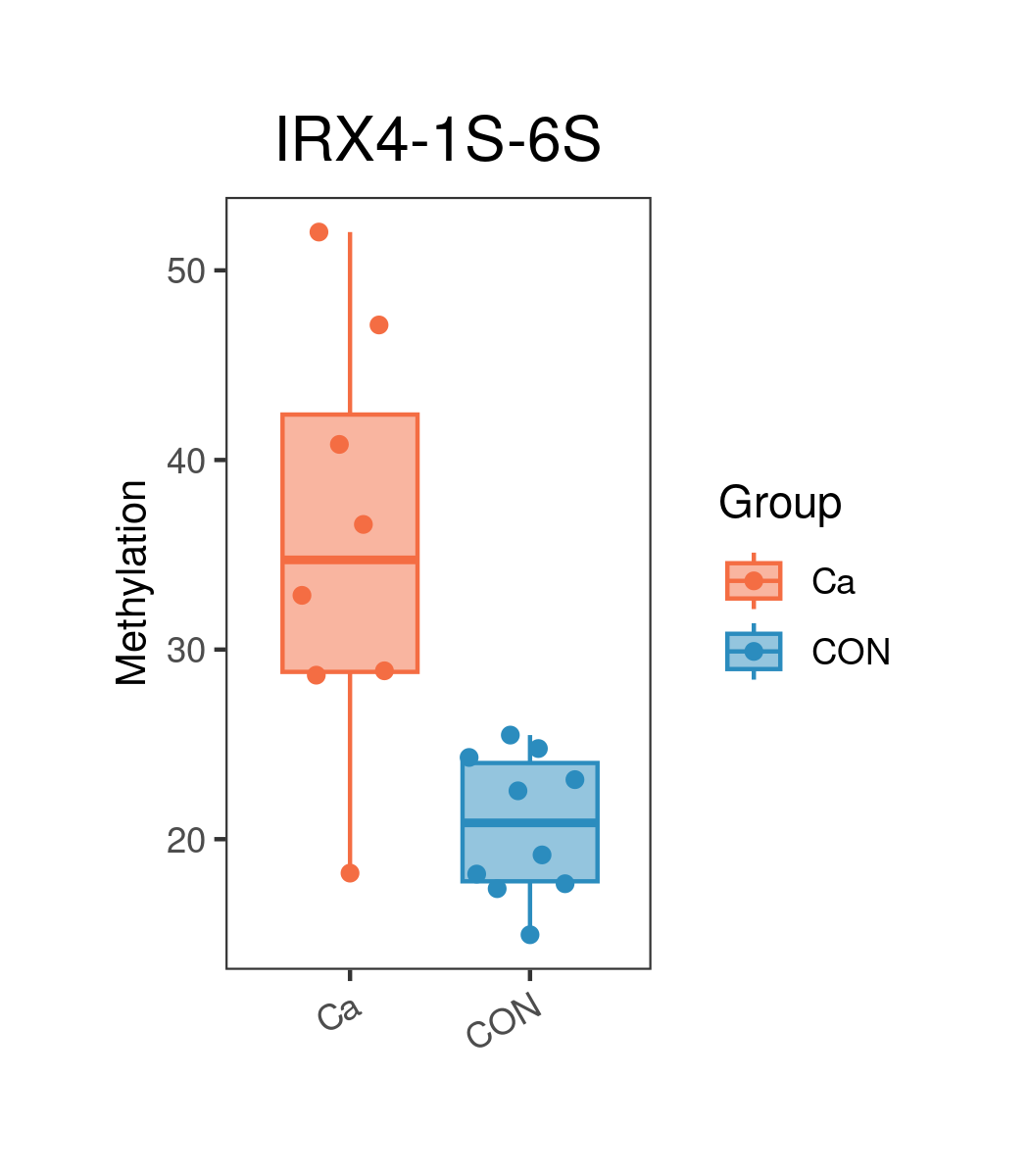

Supplement: Supplementary file 2 [file DataSheet2.zip › Analysis of Methylated Phosphorylation Data(Ca.VS.CON)/boxplot/IRX4-1S-6S_boxplot.png]

# IRX4-1S-7S

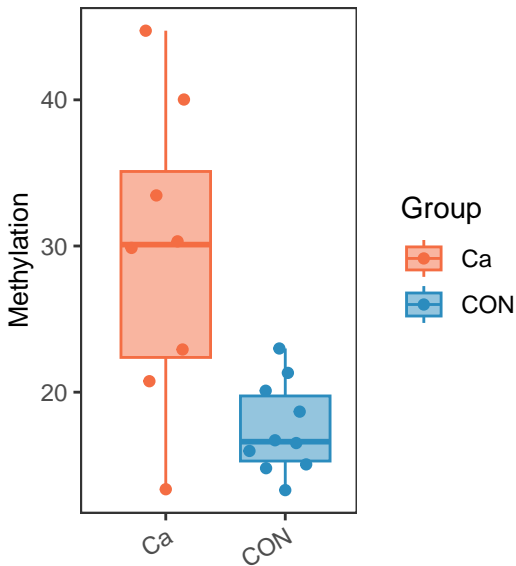

Supplement: Supplementary file 2 [file DataSheet2.zip › Analysis of Methylated Phosphorylation Data(Ca.VS.CON)/boxplot/IRX4-1S-7S_boxplot.pdf]

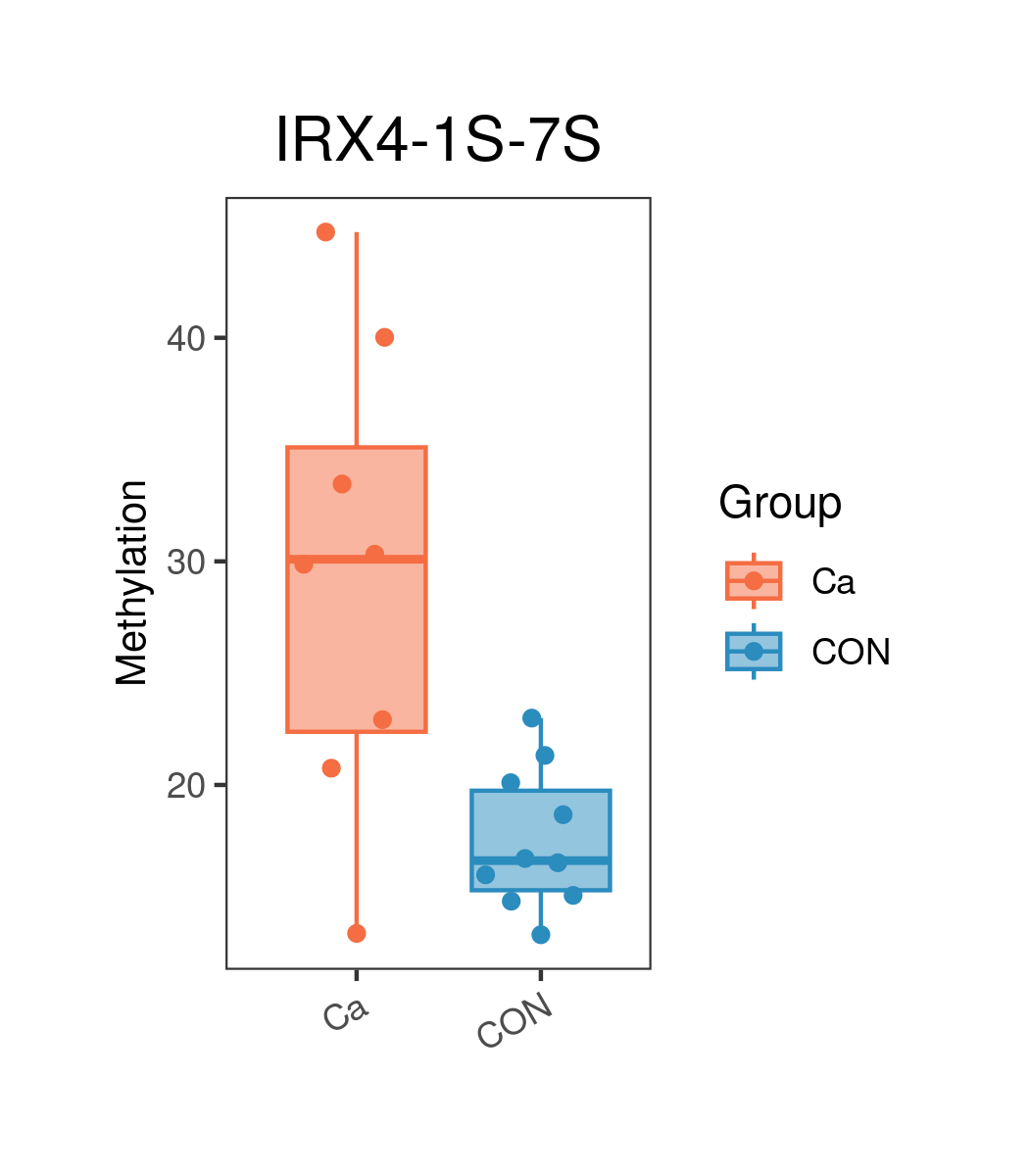

Supplement: Supplementary file 2 [file DataSheet2.zip › Analysis of Methylated Phosphorylation Data(Ca.VS.CON)/boxplot/IRX4-1S-7S_boxplot.png]

# IRX4-2S-1S

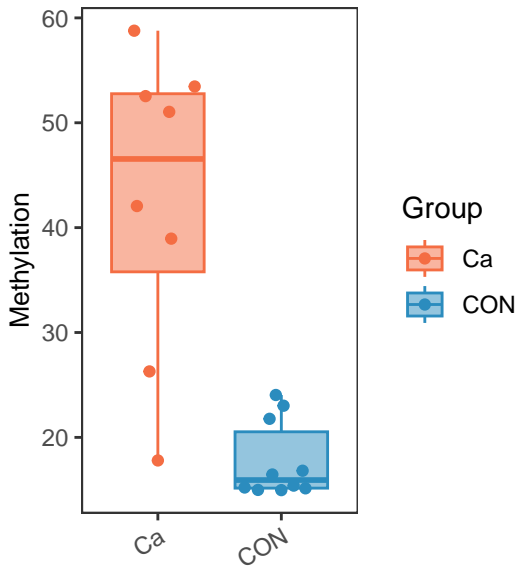

Supplement: Supplementary file 2 [file DataSheet2.zip › Analysis of Methylated Phosphorylation Data(Ca.VS.CON)/boxplot/IRX4-2S-1S_boxplot.pdf]

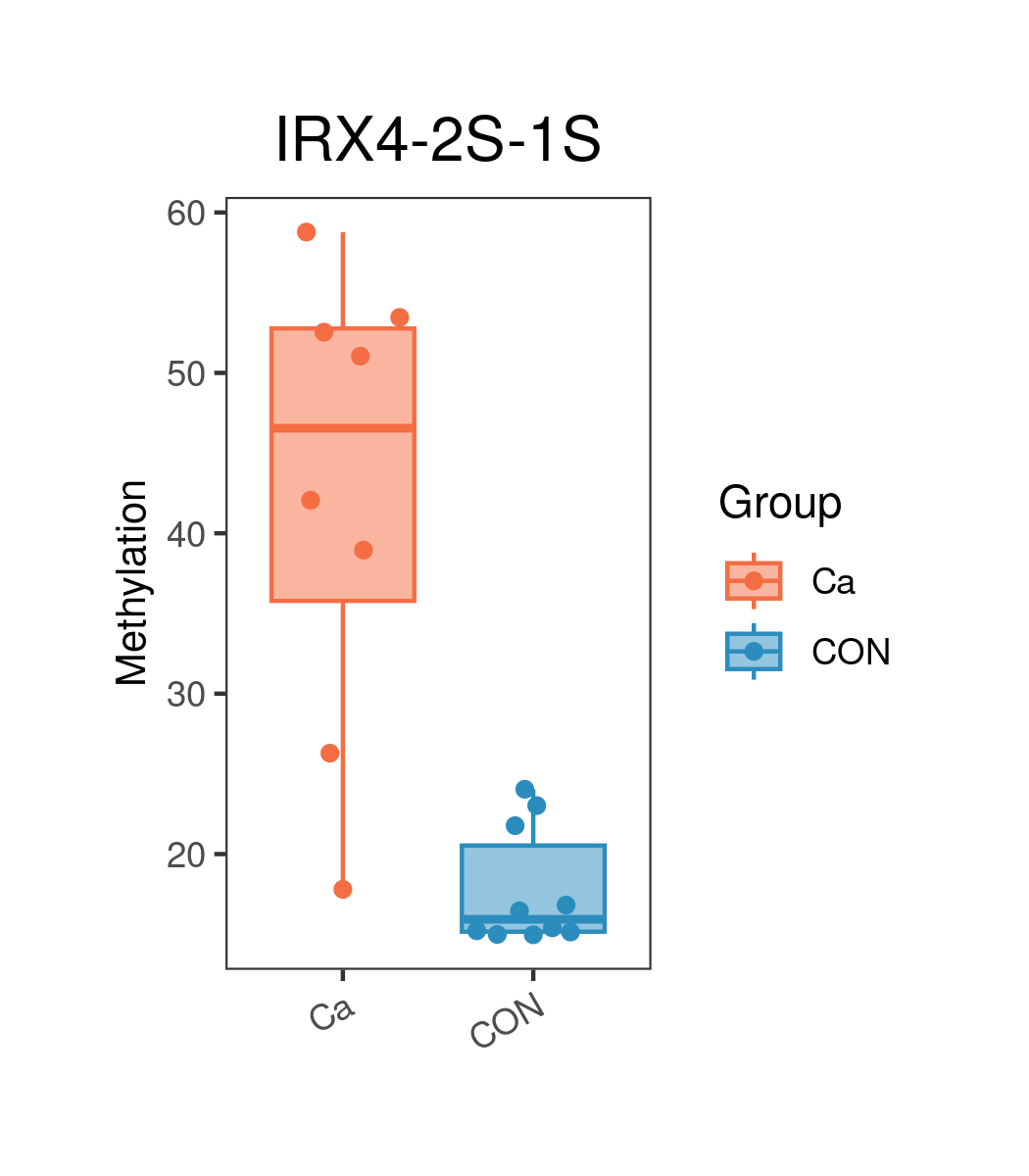

Supplement: Supplementary file 2 [file DataSheet2.zip › Analysis of Methylated Phosphorylation Data(Ca.VS.CON)/boxplot/IRX4-2S-1S_boxplot.png]

# IRX4-2S-2S

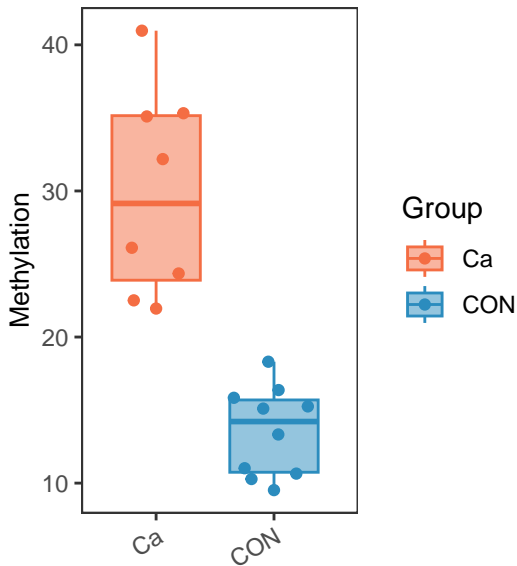

Supplement: Supplementary file 2 [file DataSheet2.zip › Analysis of Methylated Phosphorylation Data(Ca.VS.CON)/boxplot/IRX4-2S-2S_boxplot.pdf]

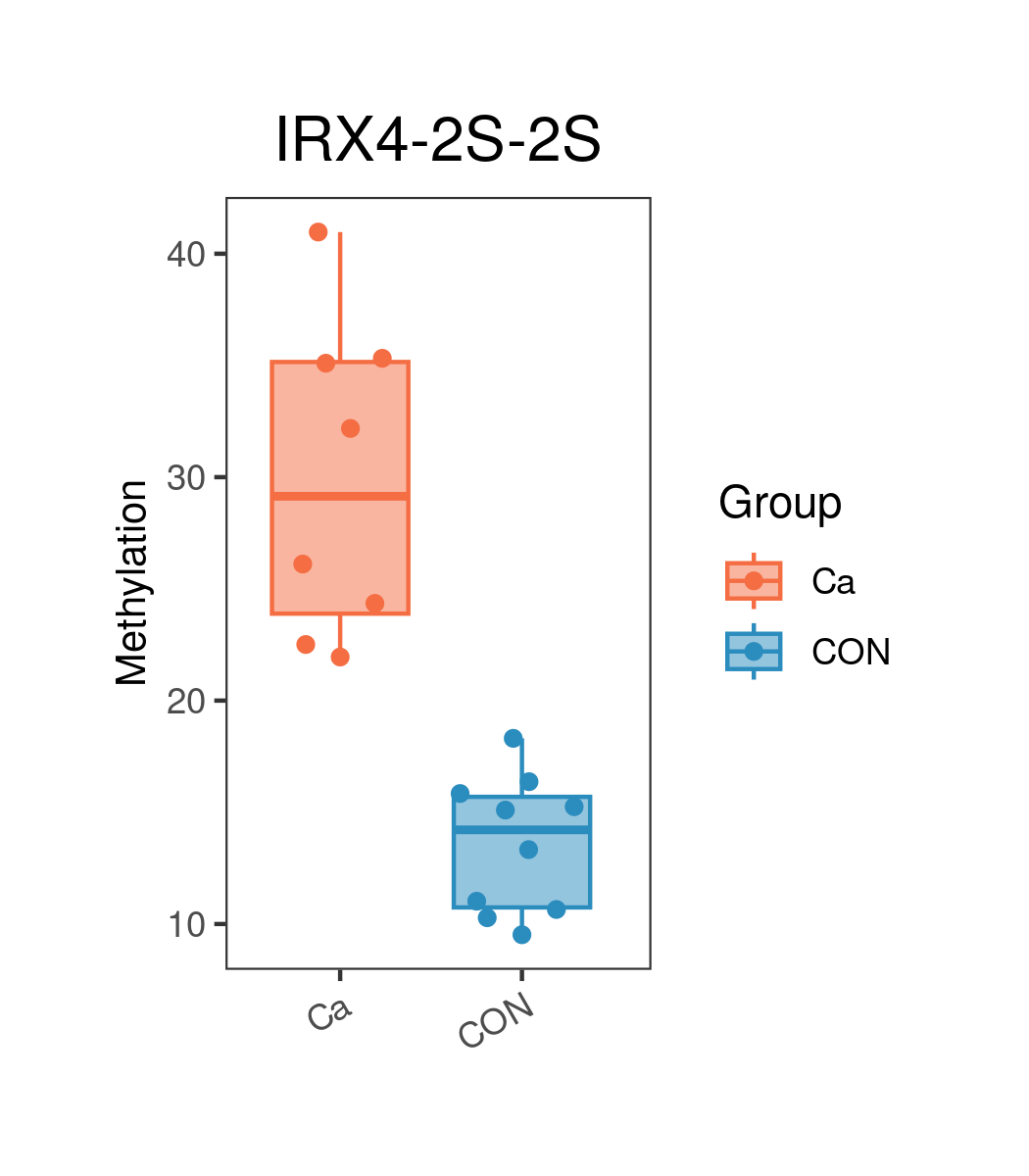

Supplement: Supplementary file 2 [file DataSheet2.zip › Analysis of Methylated Phosphorylation Data(Ca.VS.CON)/boxplot/IRX4-2S-2S_boxplot.png]

# IRX4-2S-3S

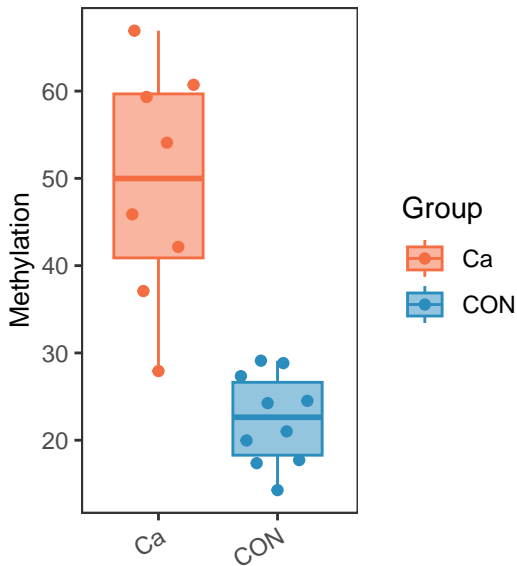

Supplement: Supplementary file 2 [file DataSheet2.zip › Analysis of Methylated Phosphorylation Data(Ca.VS.CON)/boxplot/IRX4-2S-3S_boxplot.pdf]

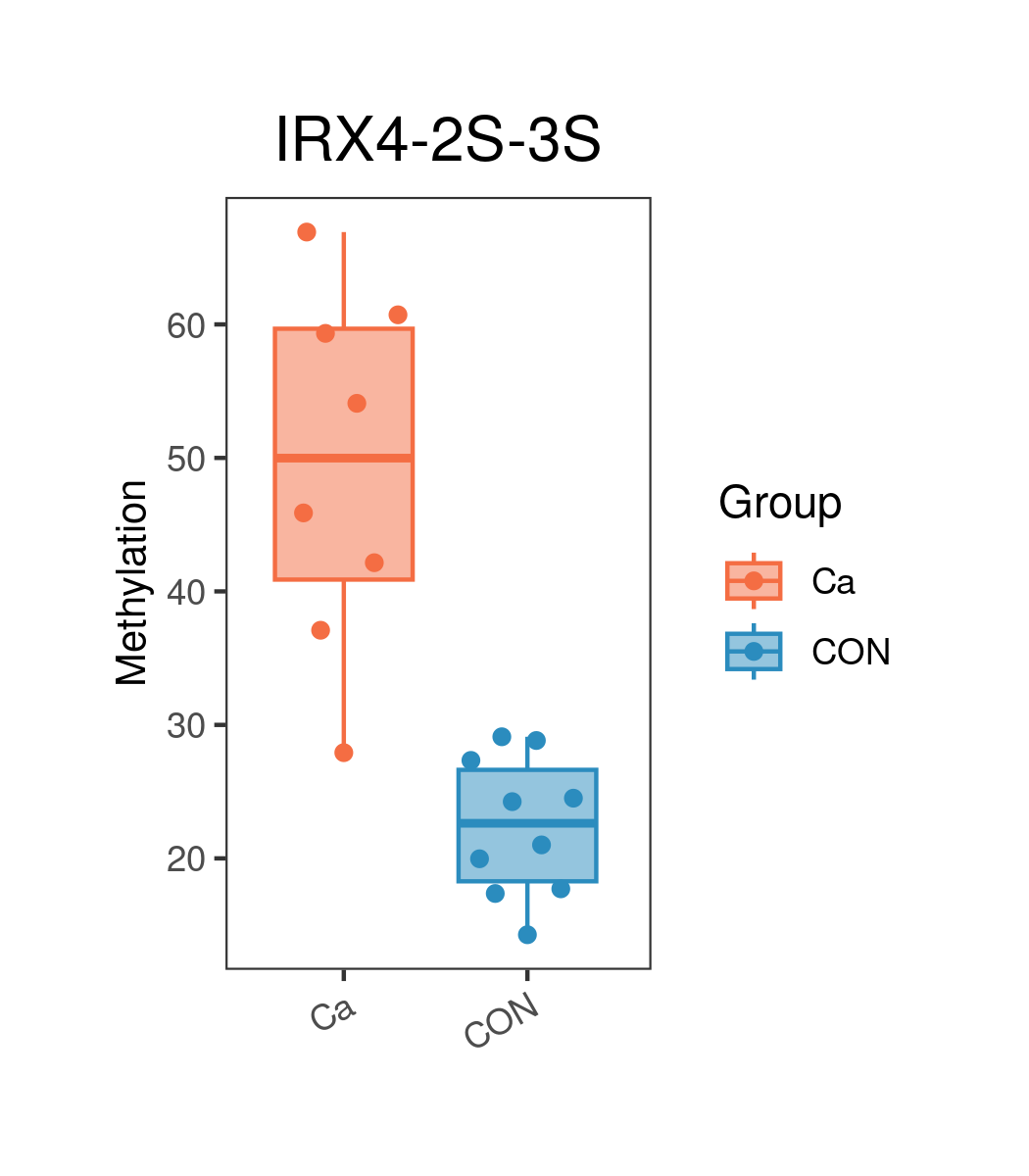

Supplement: Supplementary file 2 [file DataSheet2.zip › Analysis of Methylated Phosphorylation Data(Ca.VS.CON)/boxplot/IRX4-2S-3S_boxplot.png]

# IRX4-2S-4S

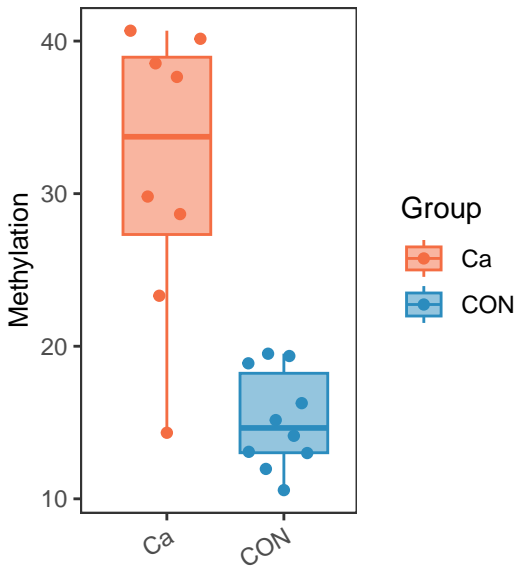

Supplement: Supplementary file 2 [file DataSheet2.zip › Analysis of Methylated Phosphorylation Data(Ca.VS.CON)/boxplot/IRX4-2S-4S_boxplot.pdf]

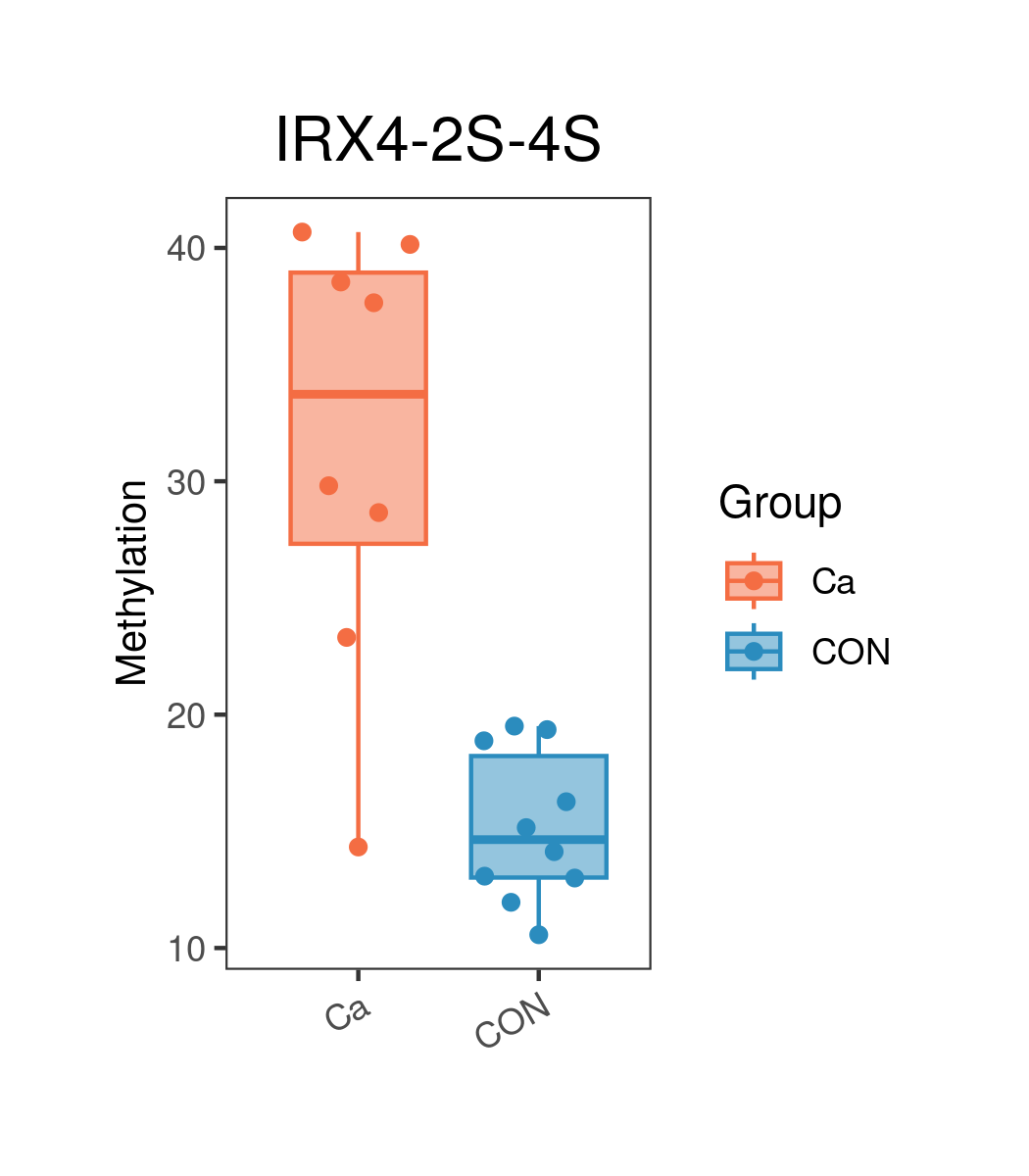

Supplement: Supplementary file 2 [file DataSheet2.zip › Analysis of Methylated Phosphorylation Data(Ca.VS.CON)/boxplot/IRX4-2S-4S_boxplot.png]

# IRX4-2S-5S

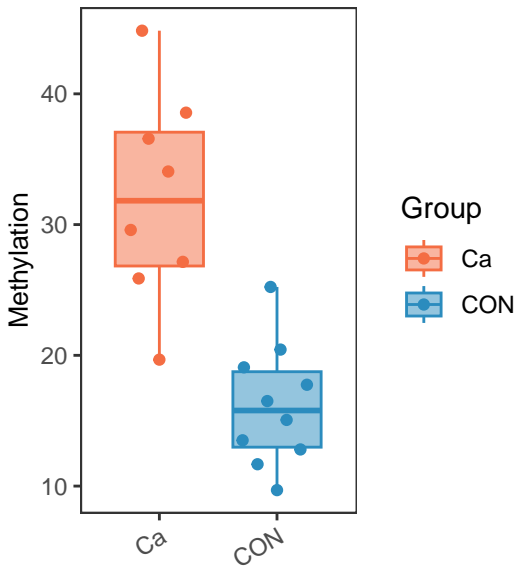

Supplement: Supplementary file 2 [file DataSheet2.zip › Analysis of Methylated Phosphorylation Data(Ca.VS.CON)/boxplot/IRX4-2S-5S_boxplot.pdf]

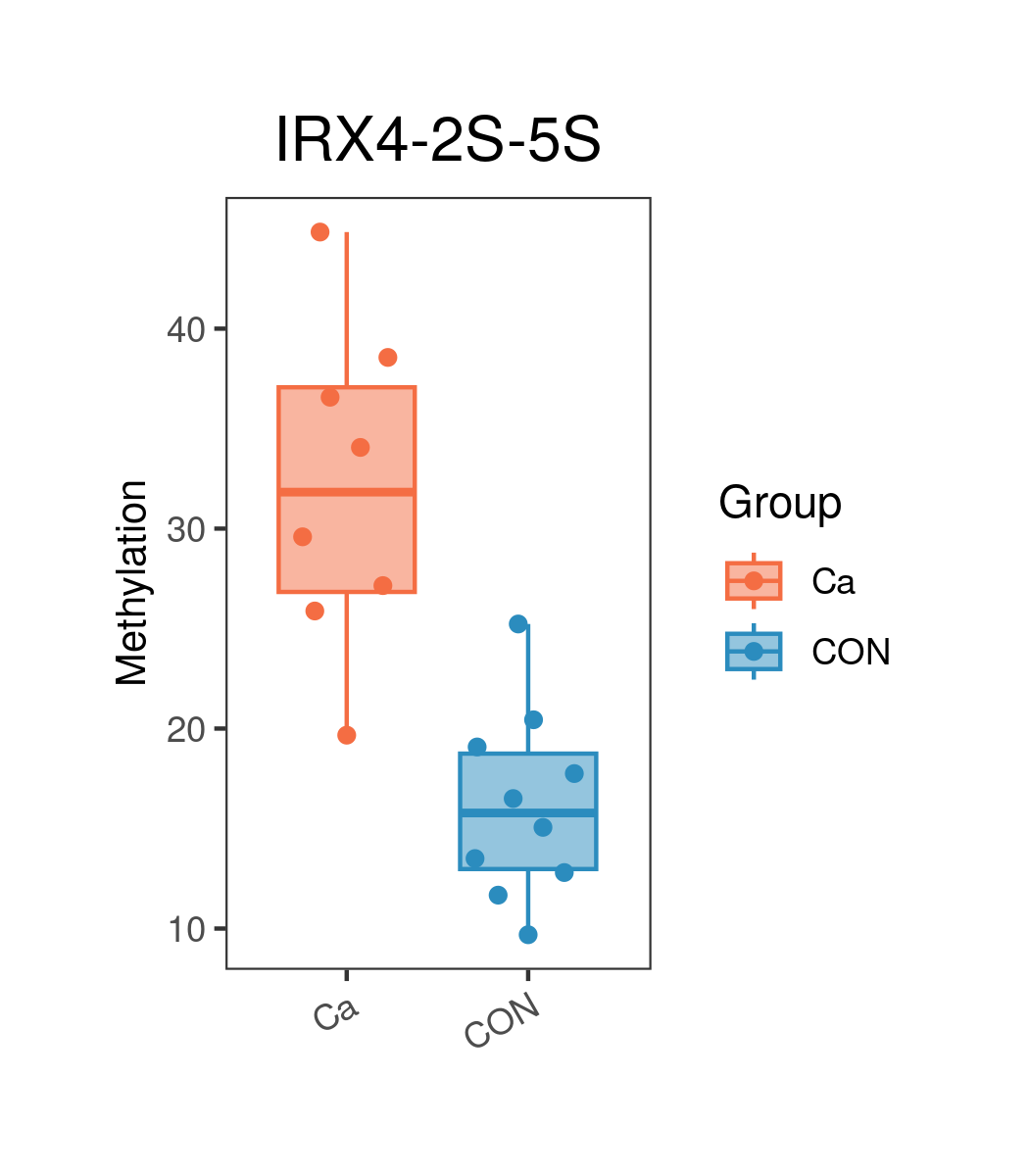

Supplement: Supplementary file 2 [file DataSheet2.zip › Analysis of Methylated Phosphorylation Data(Ca.VS.CON)/boxplot/IRX4-2S-5S_boxplot.png]

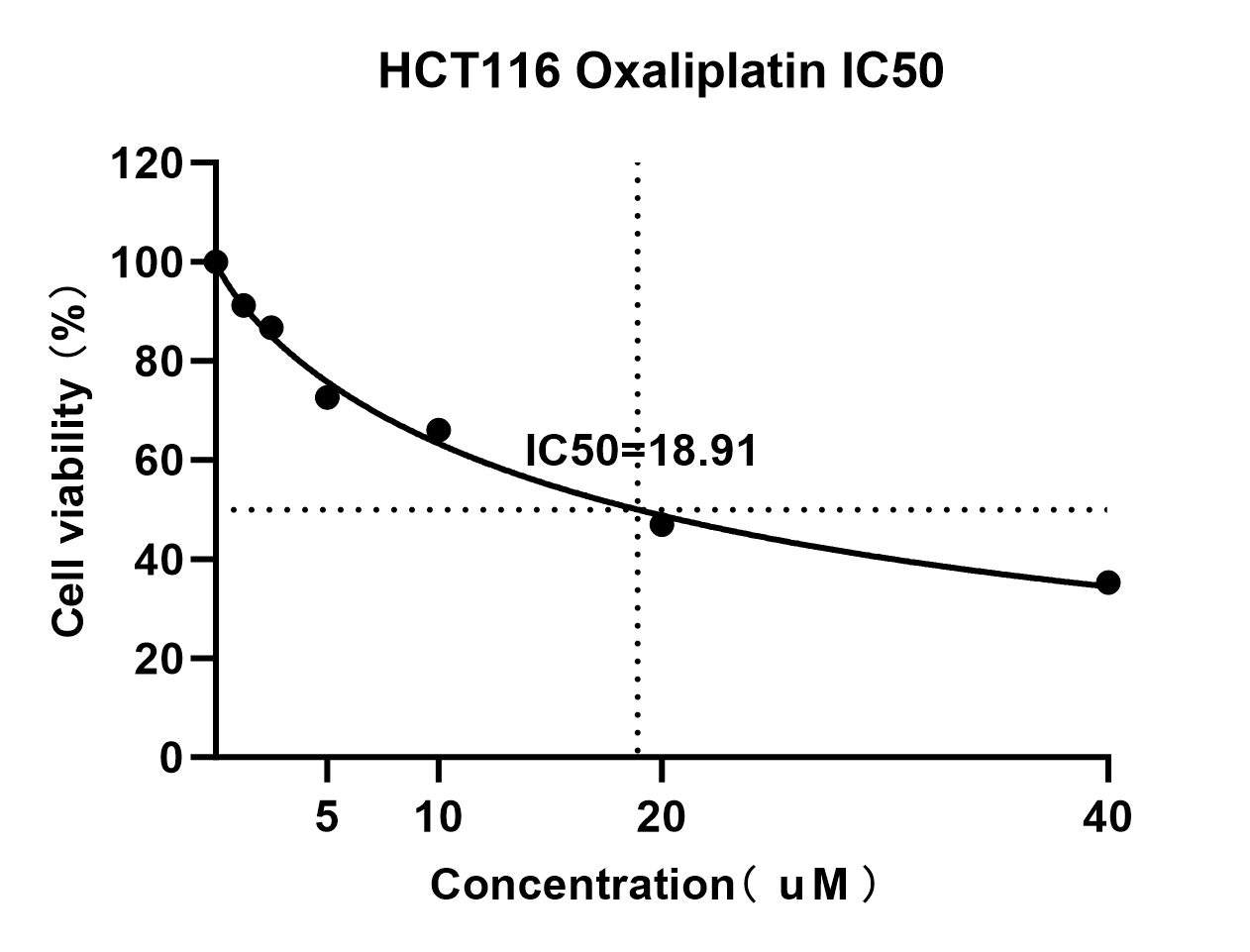

Supplement: Supplementary file 3 [file DataSheet3.zip › CCK8/1-IC50/HCT116 Oxaliplatin IC50.tif]

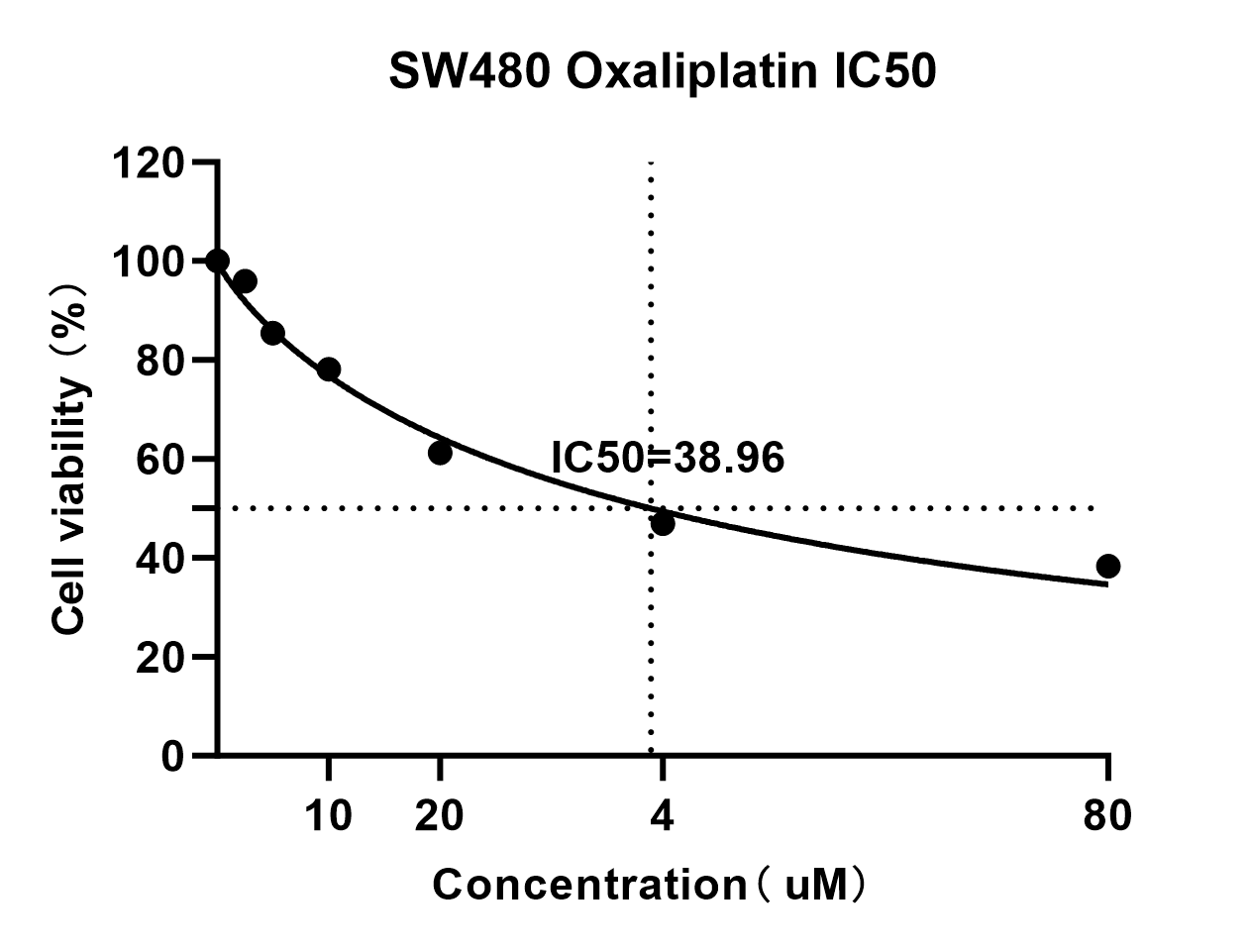

Supplement: Supplementary file 3 [file DataSheet3.zip › CCK8/1-IC50/SW480 Oxaliplatin IC50.tif]

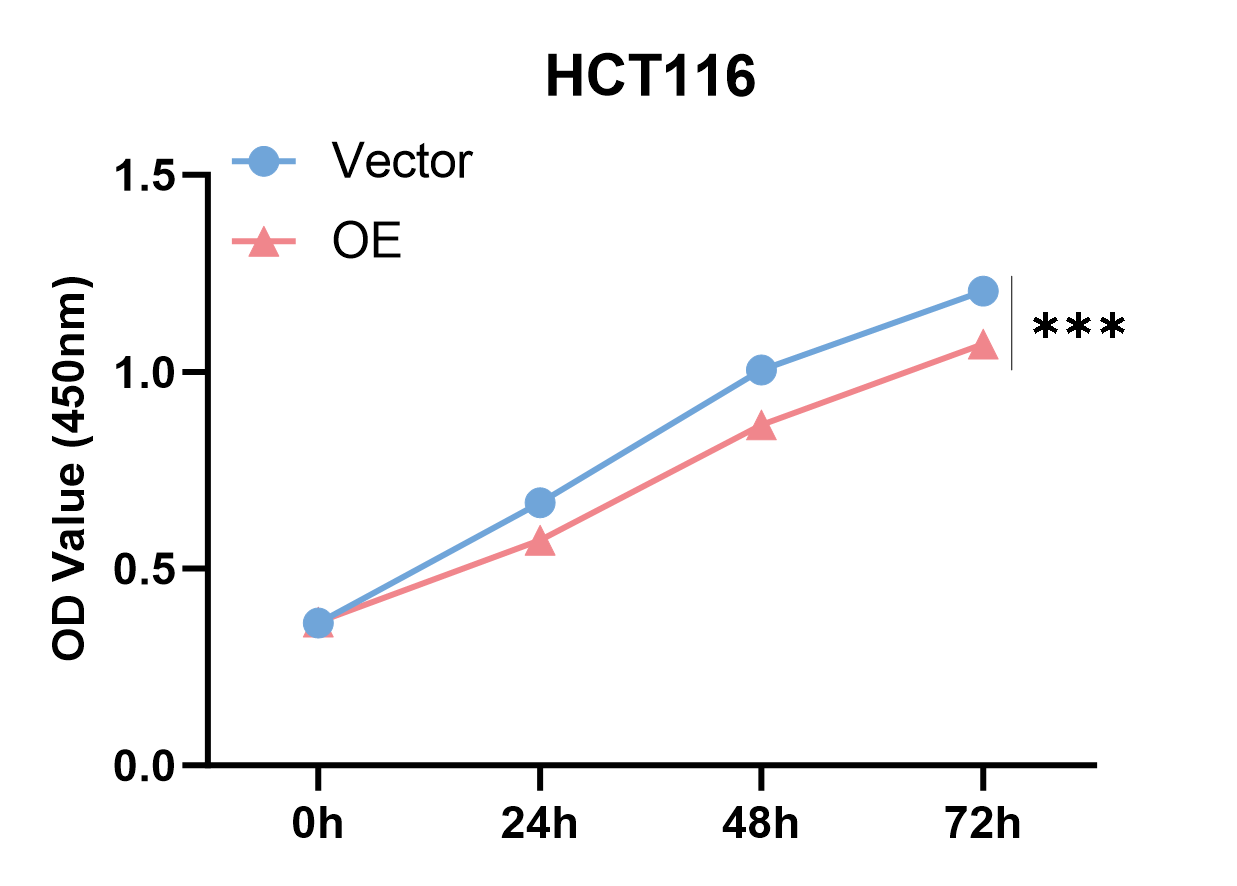

Supplement: Supplementary file 3 [file DataSheet3.zip › CCK8/Data 1.tif]

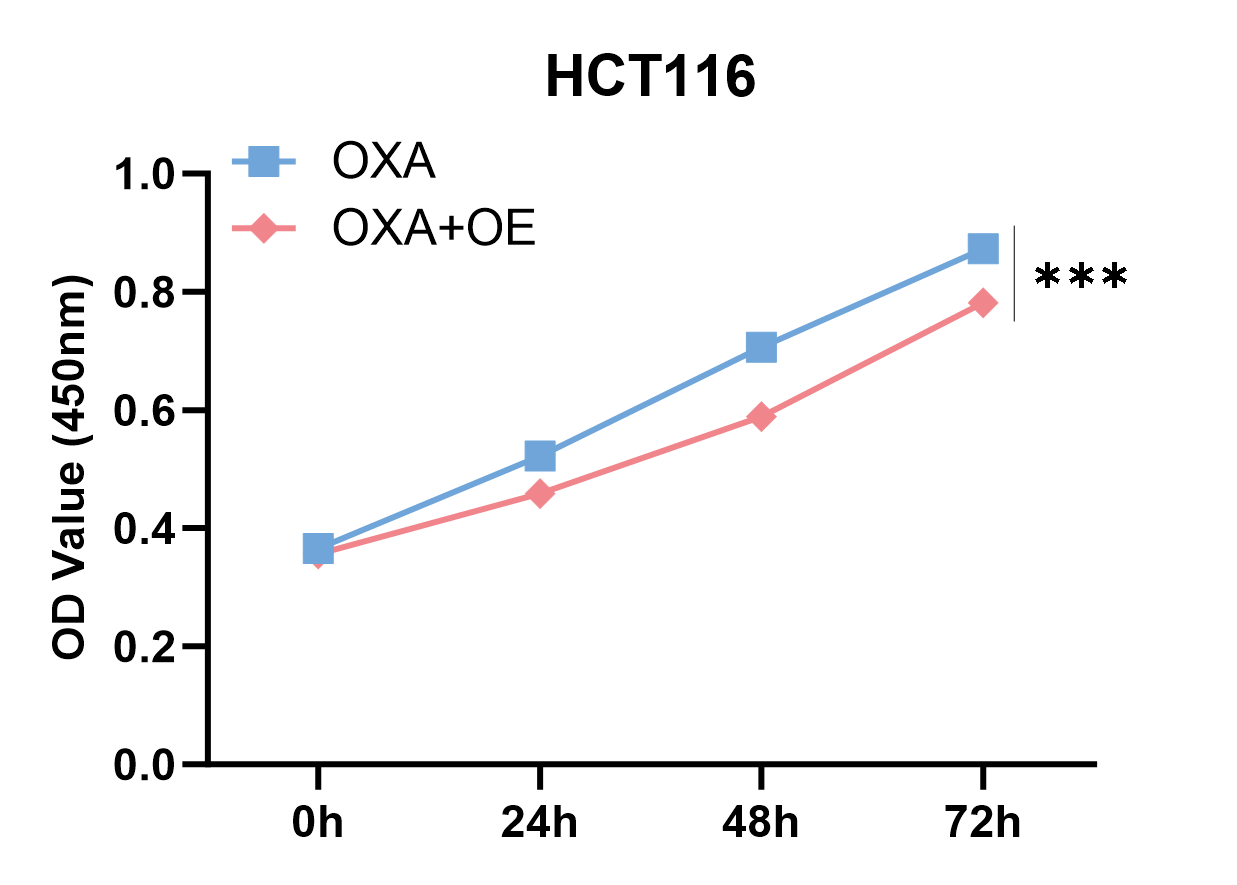

Supplement: Supplementary file 3 [file DataSheet3.zip › CCK8/Data 2.tif]

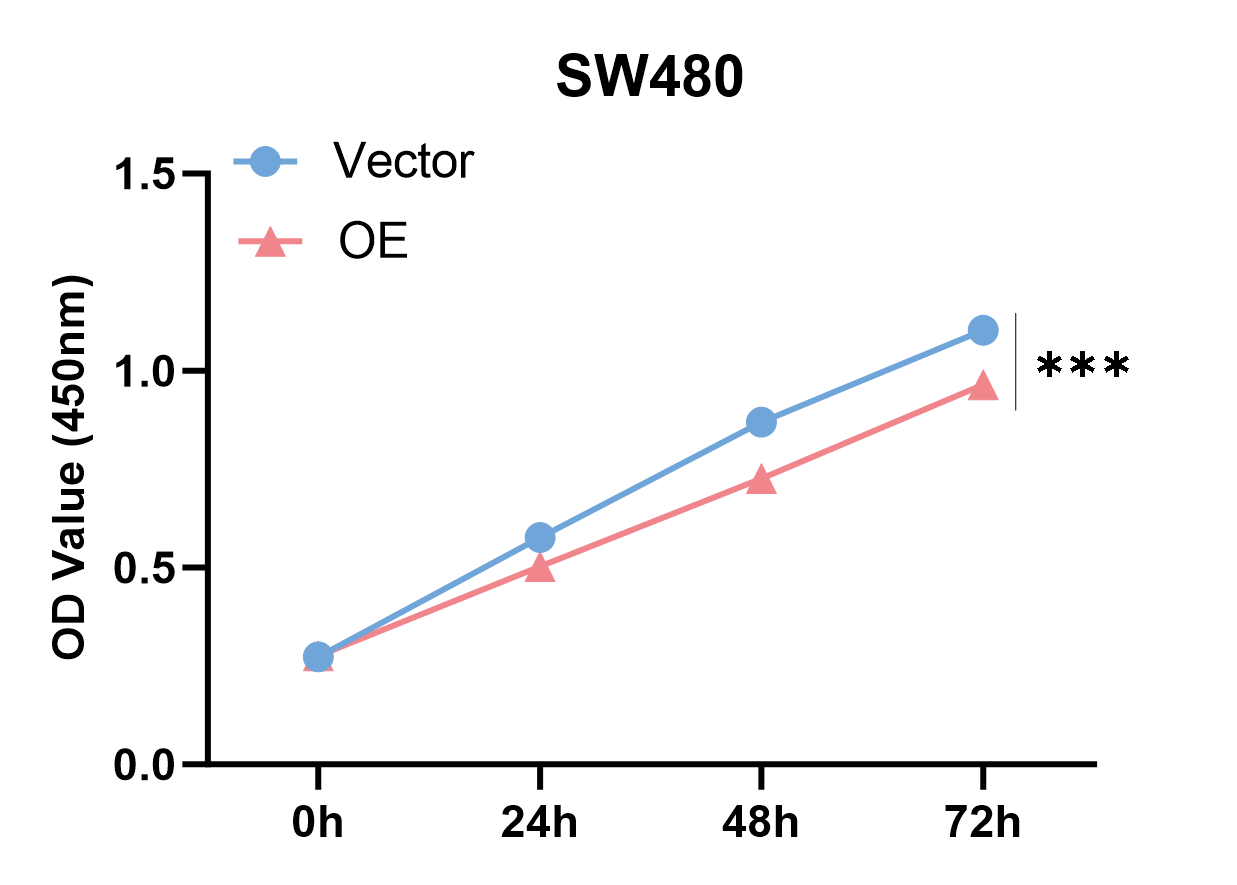

Supplement: Supplementary file 3 [file DataSheet3.zip › CCK8/Data 3.tif]

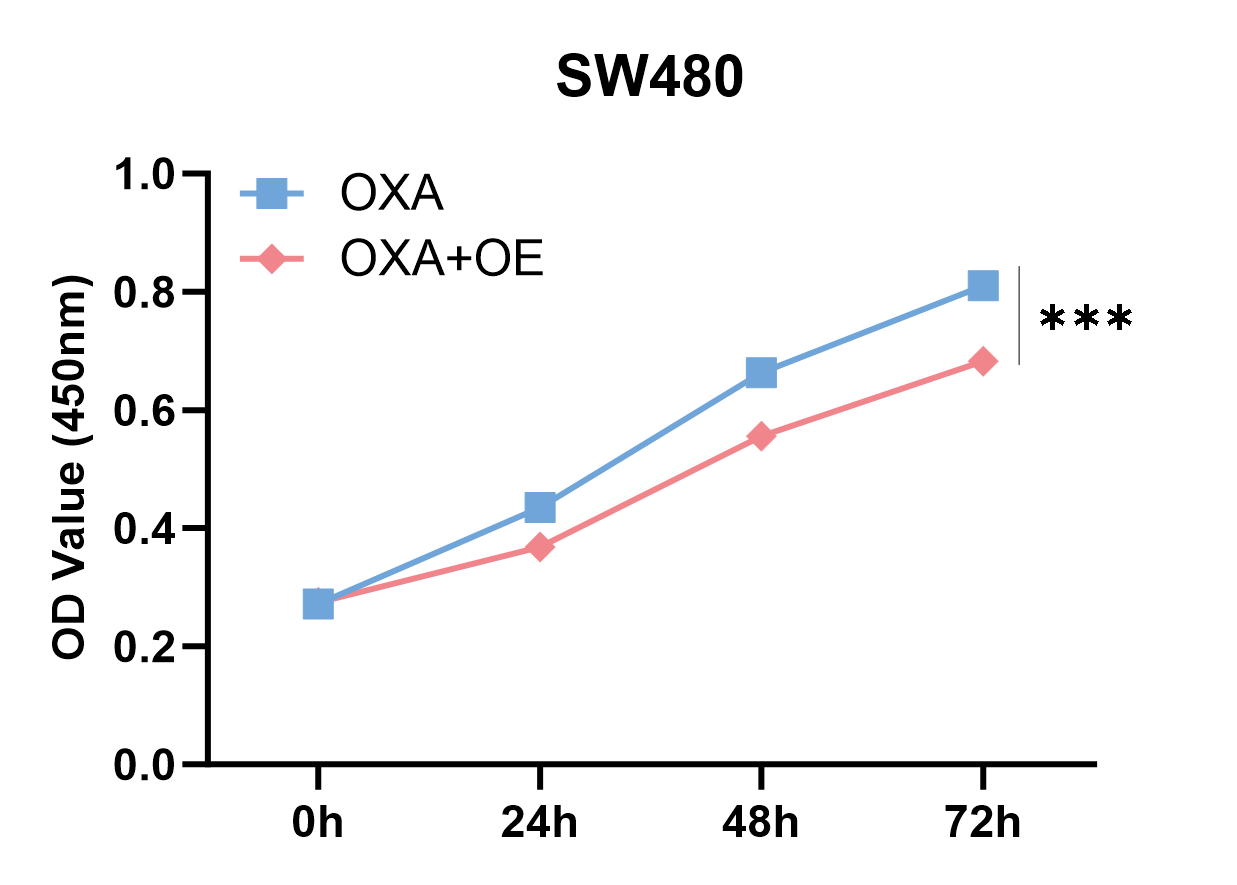

Supplement: Supplementary file 3 [file DataSheet3.zip › CCK8/Data4.tif]

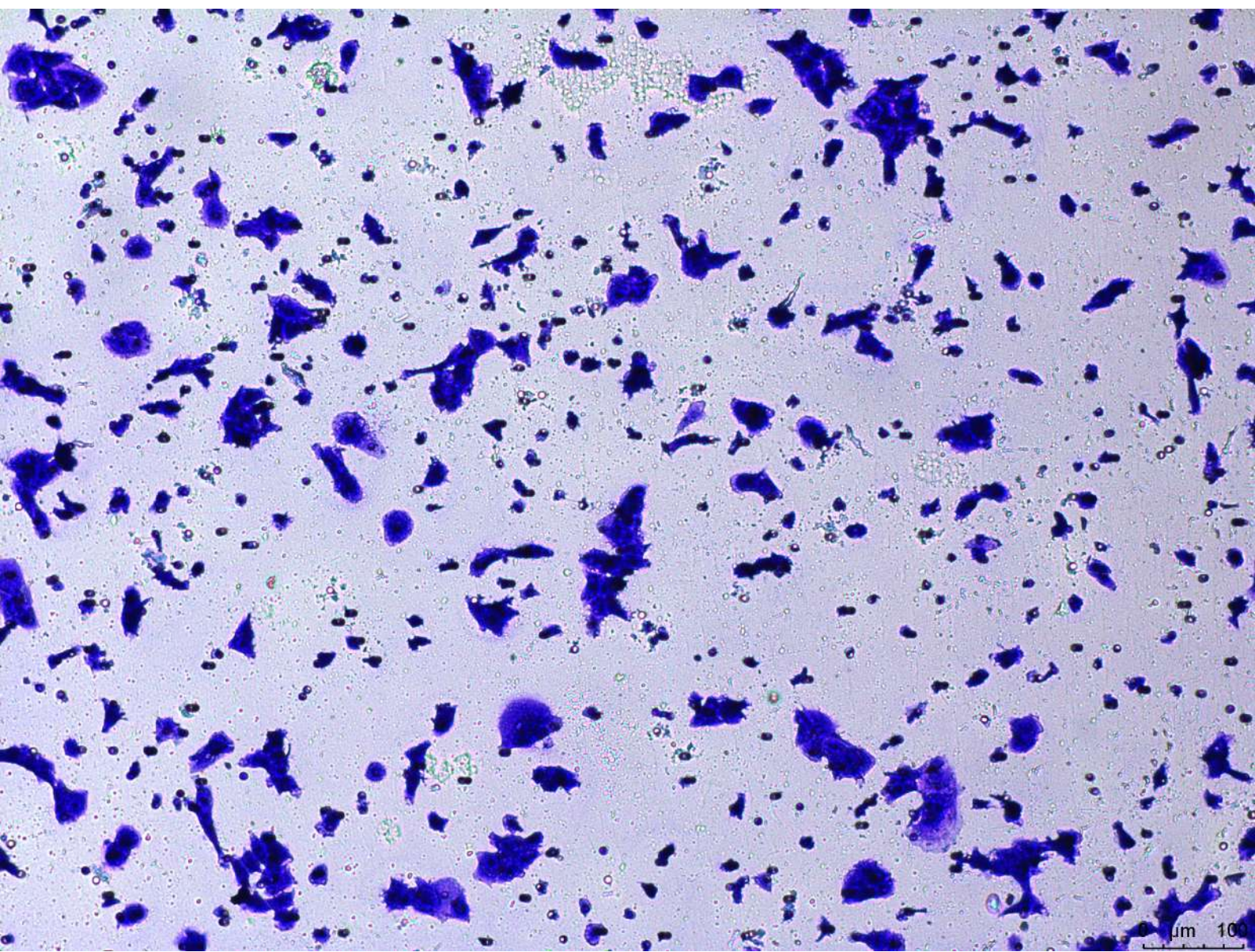

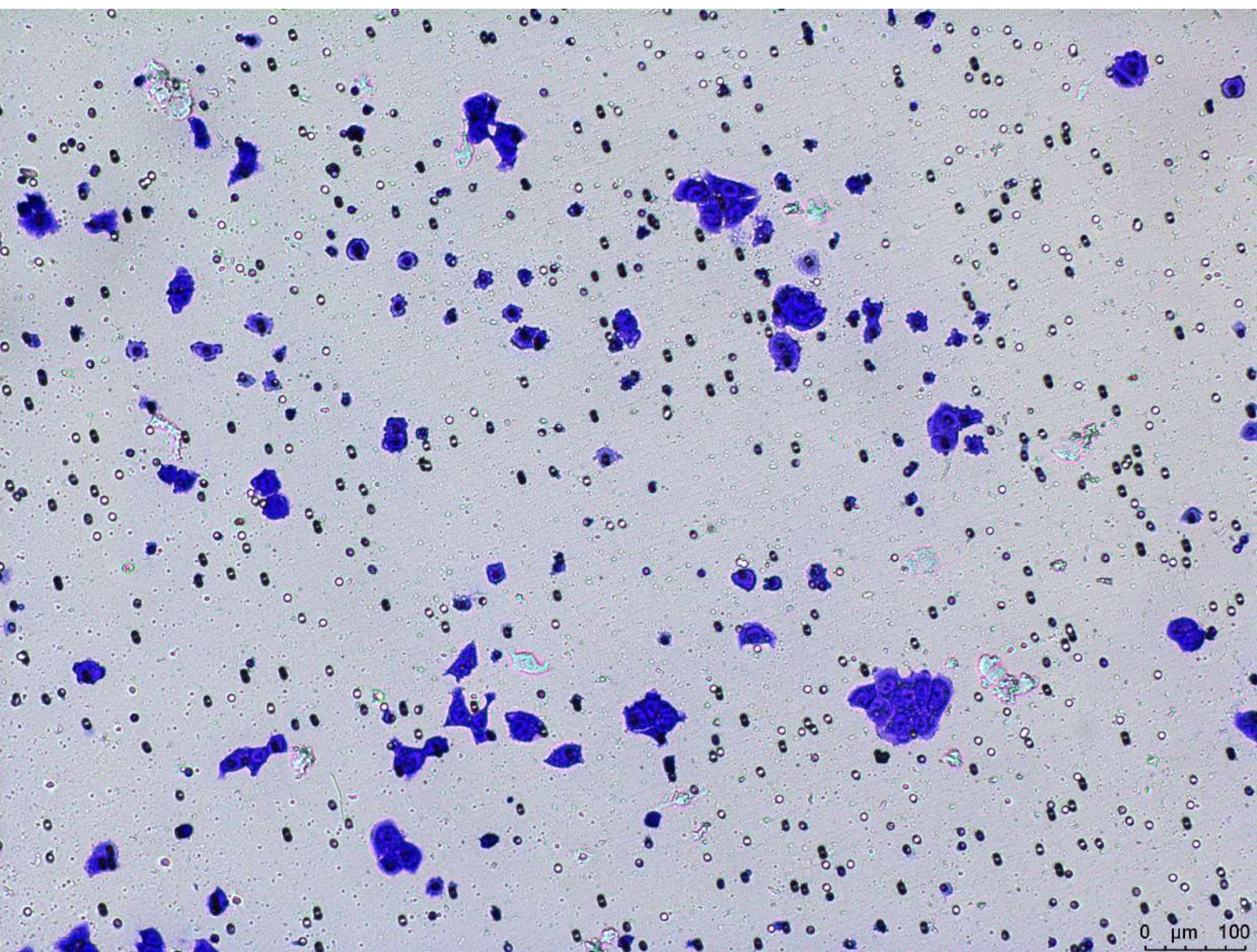

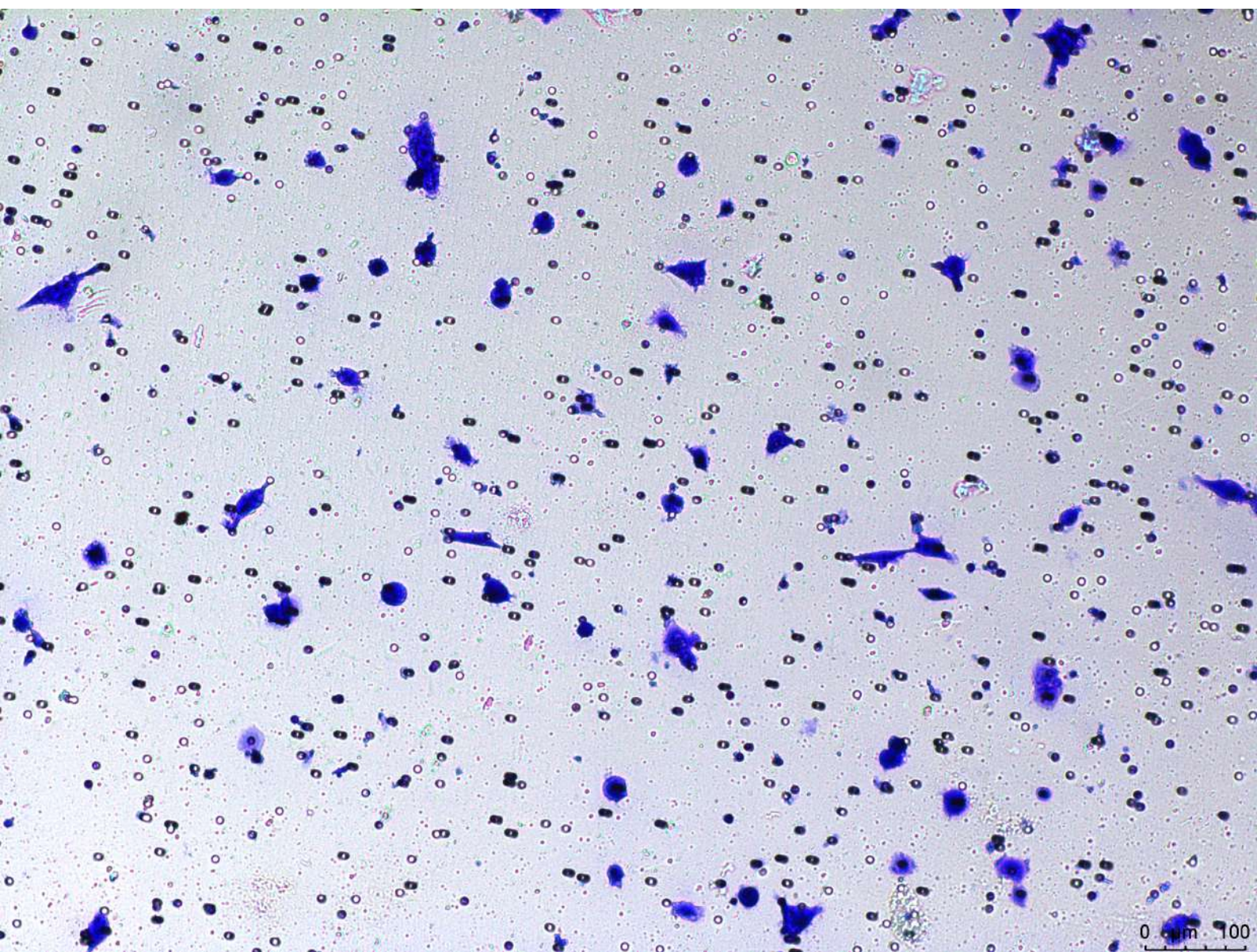

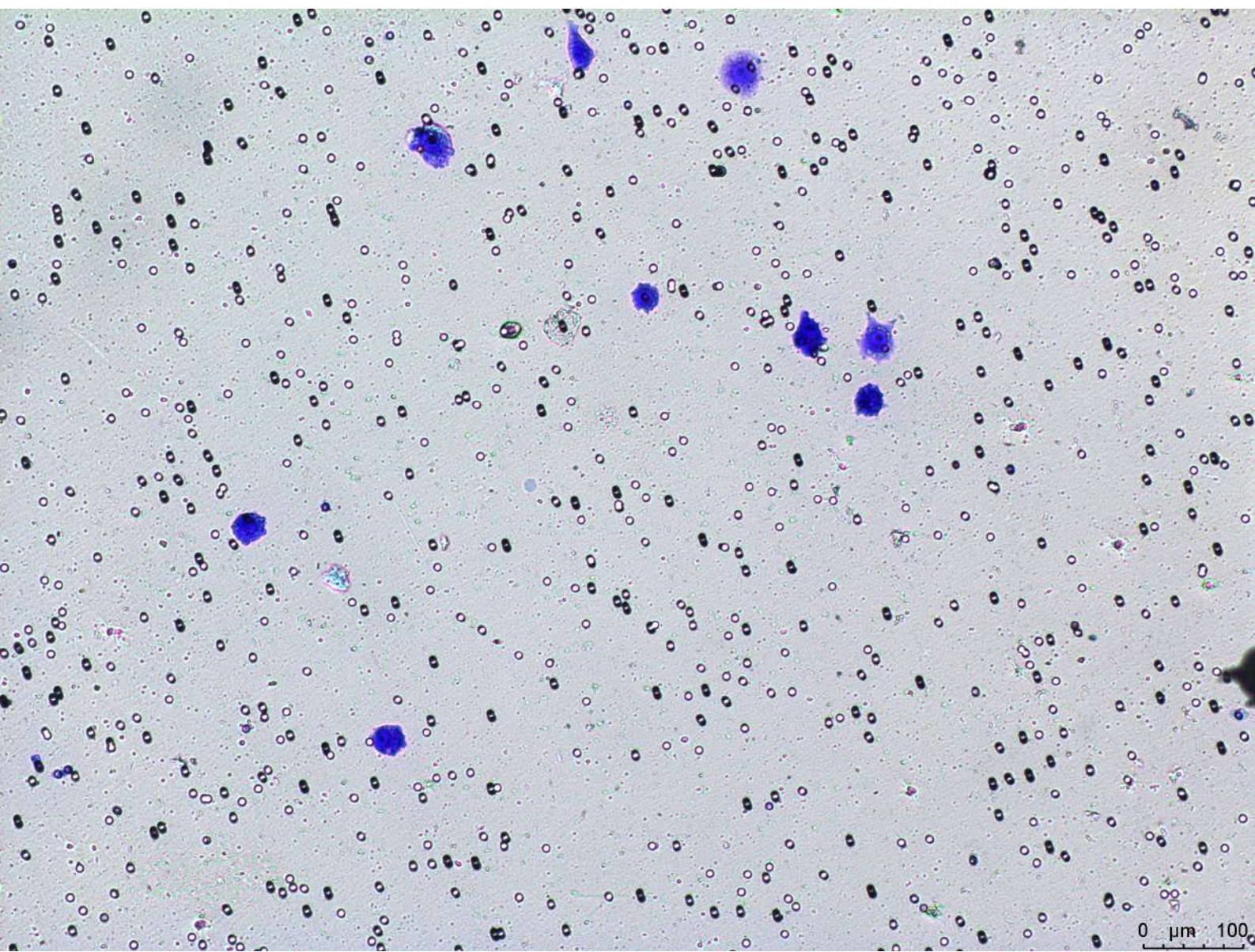

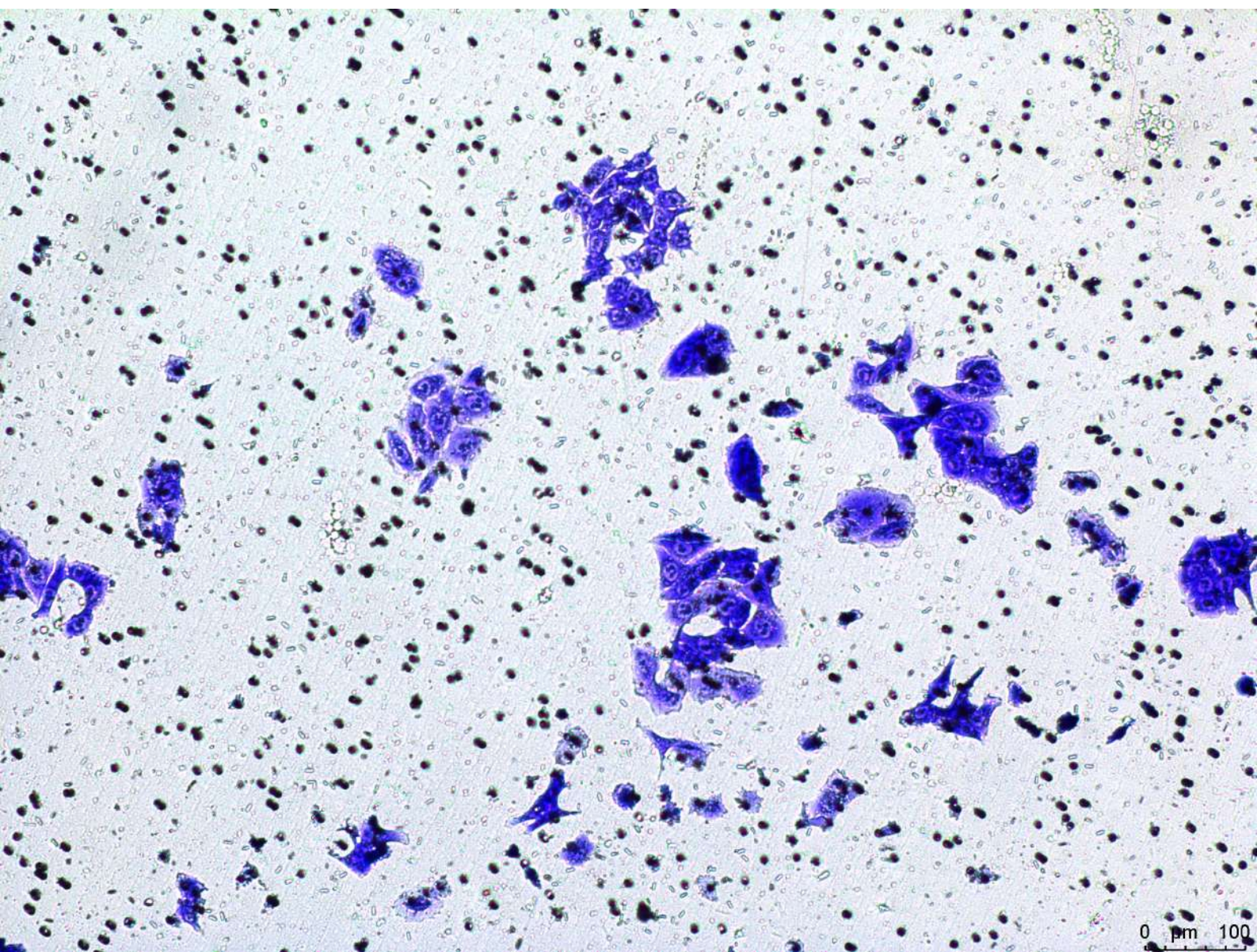

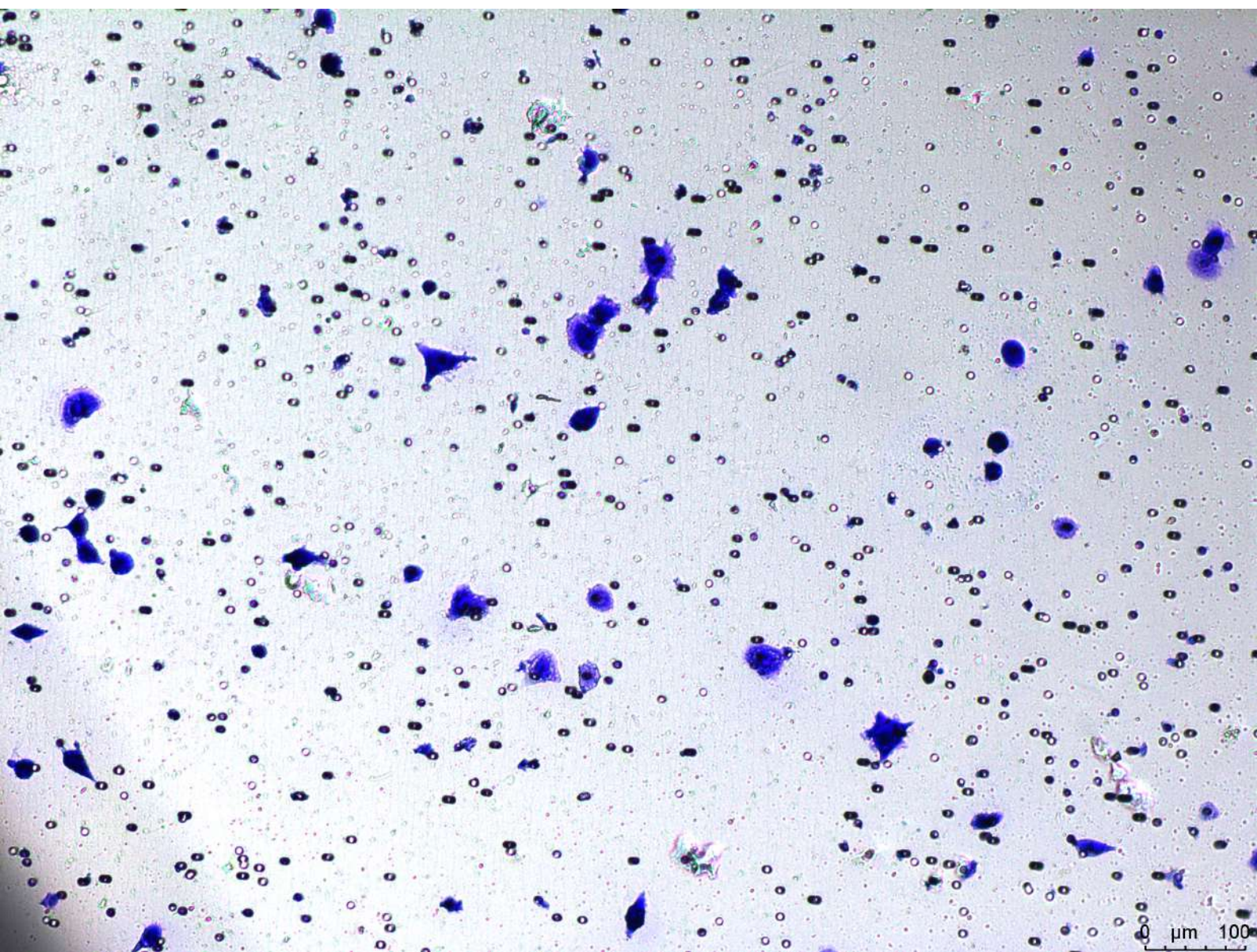

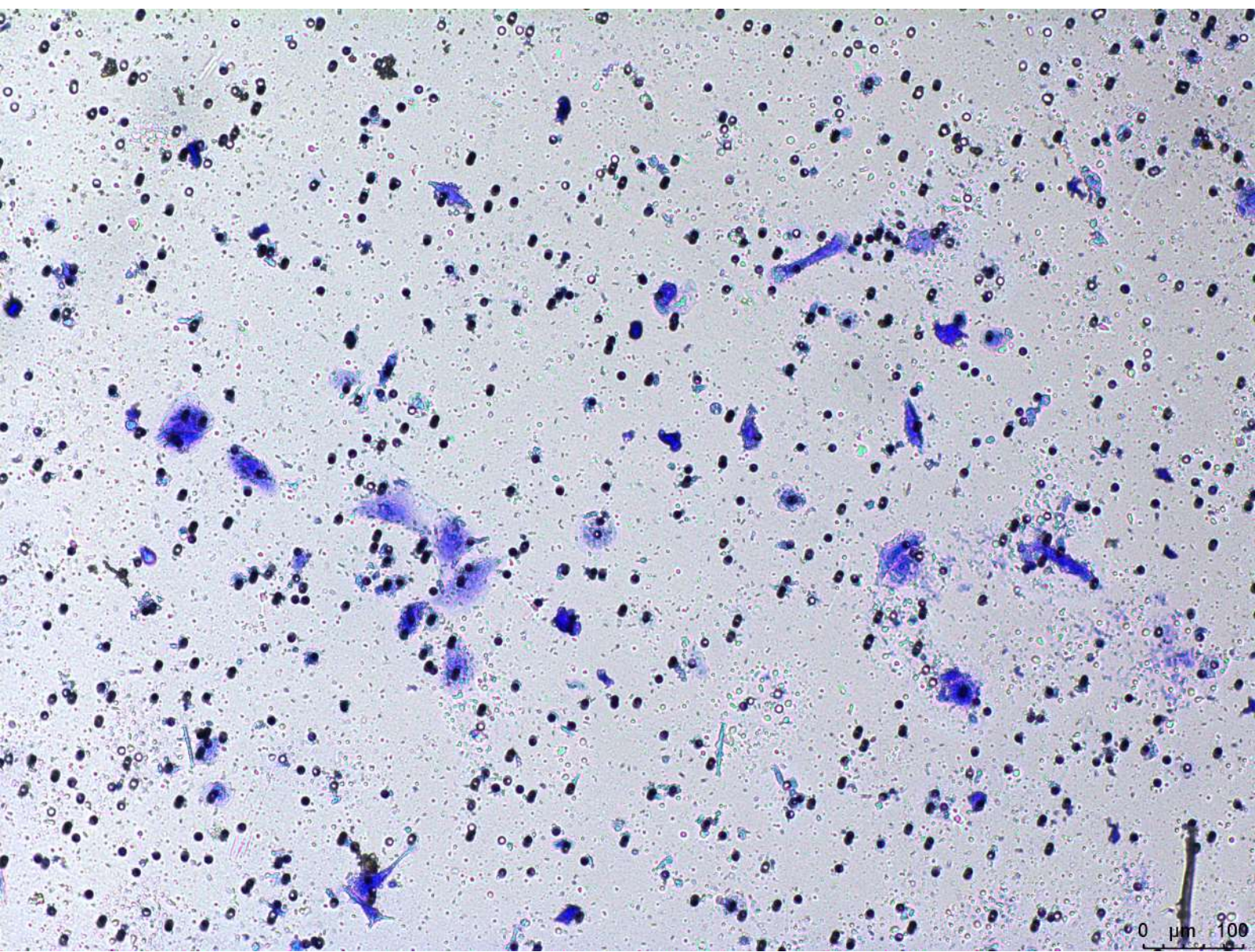

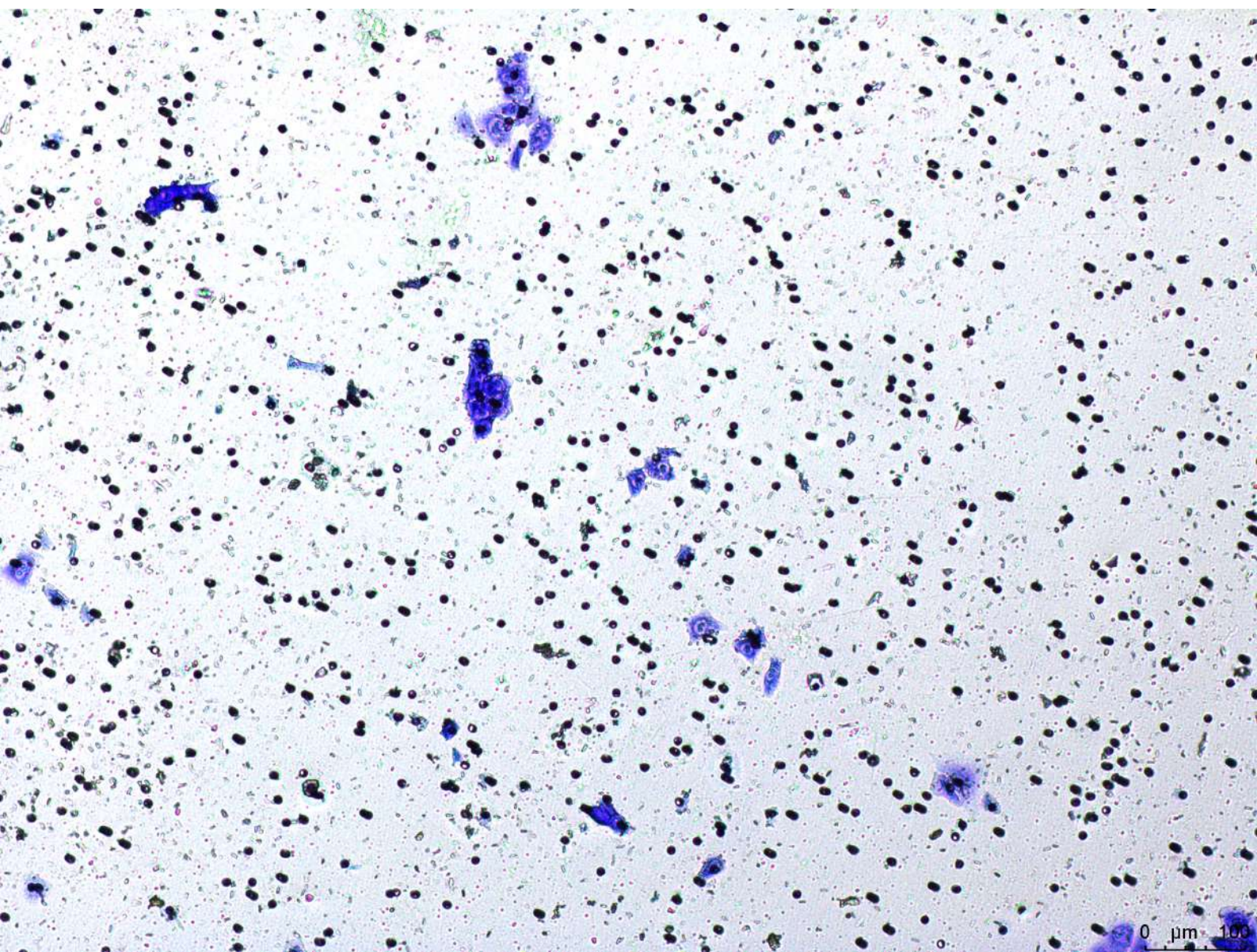

Supplement: Supplementary file 4 [file DataSheet4.zip › Cell invasion/Cell invasion.pdf]

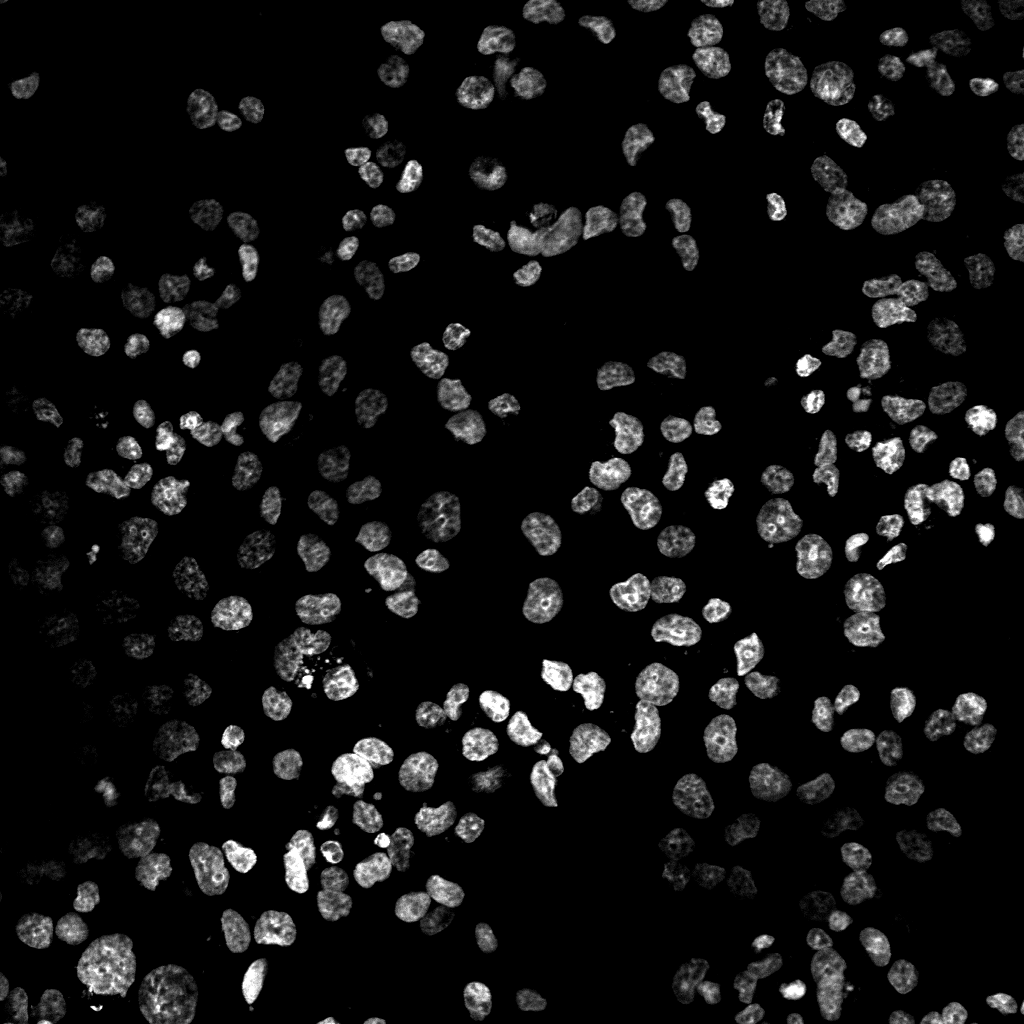

Supplement: Supplementary file 6 [file DataSheet6.zip › EDU/HCT116-EDU/+OXA/14.tif]

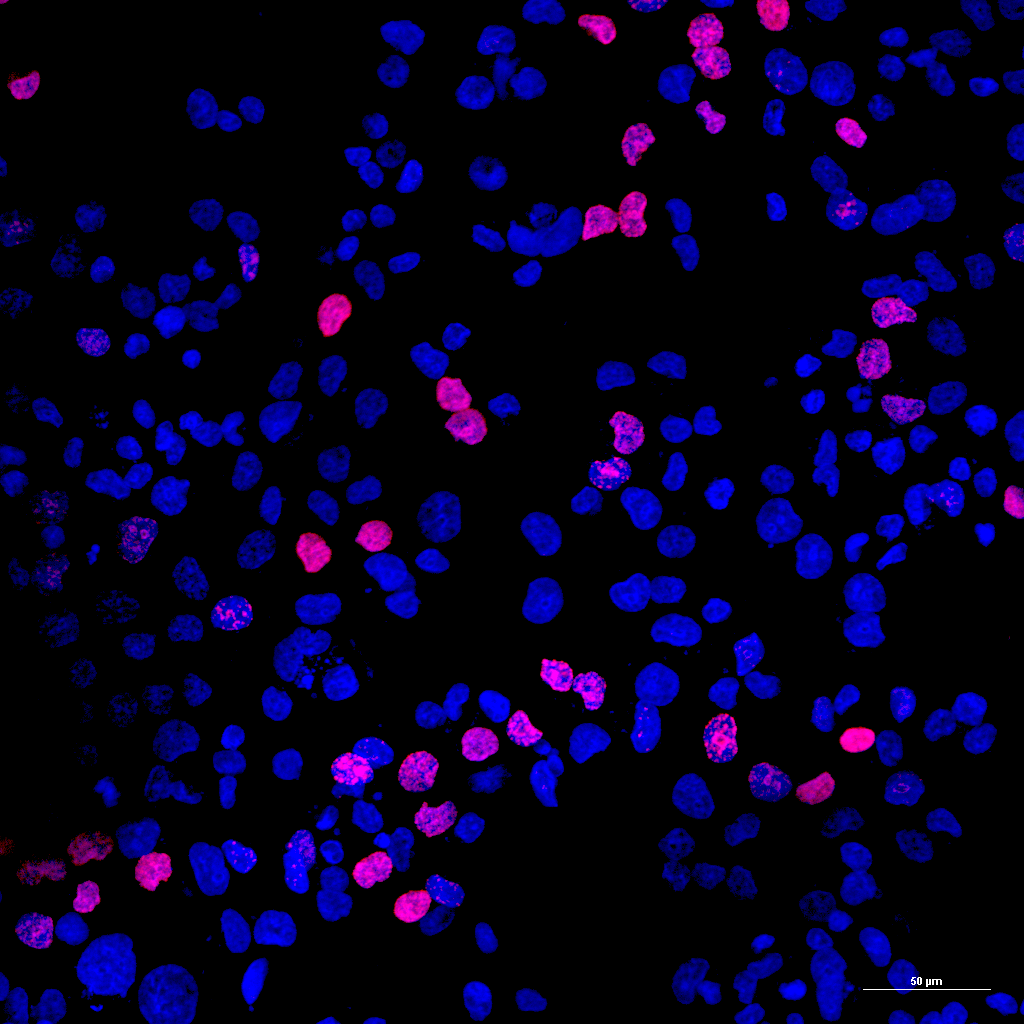

Supplement: Supplementary file 6 [file DataSheet6.zip › EDU/HCT116-EDU/+OXA/14_RGB.tif]

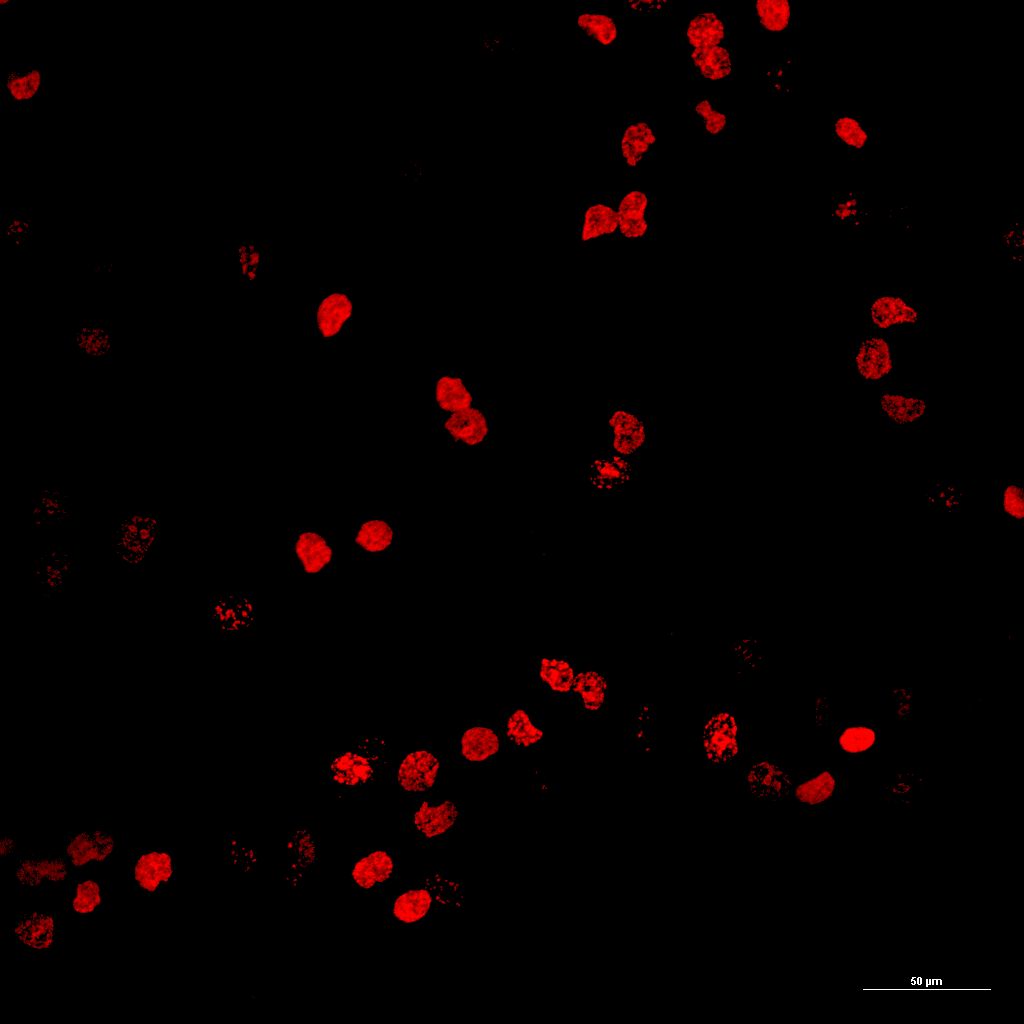

Supplement: Supplementary file 6 [file DataSheet6.zip › EDU/HCT116-EDU/+OXA/14_RGB_Alexa Fluor 594 cadaverine_H2O.tif]

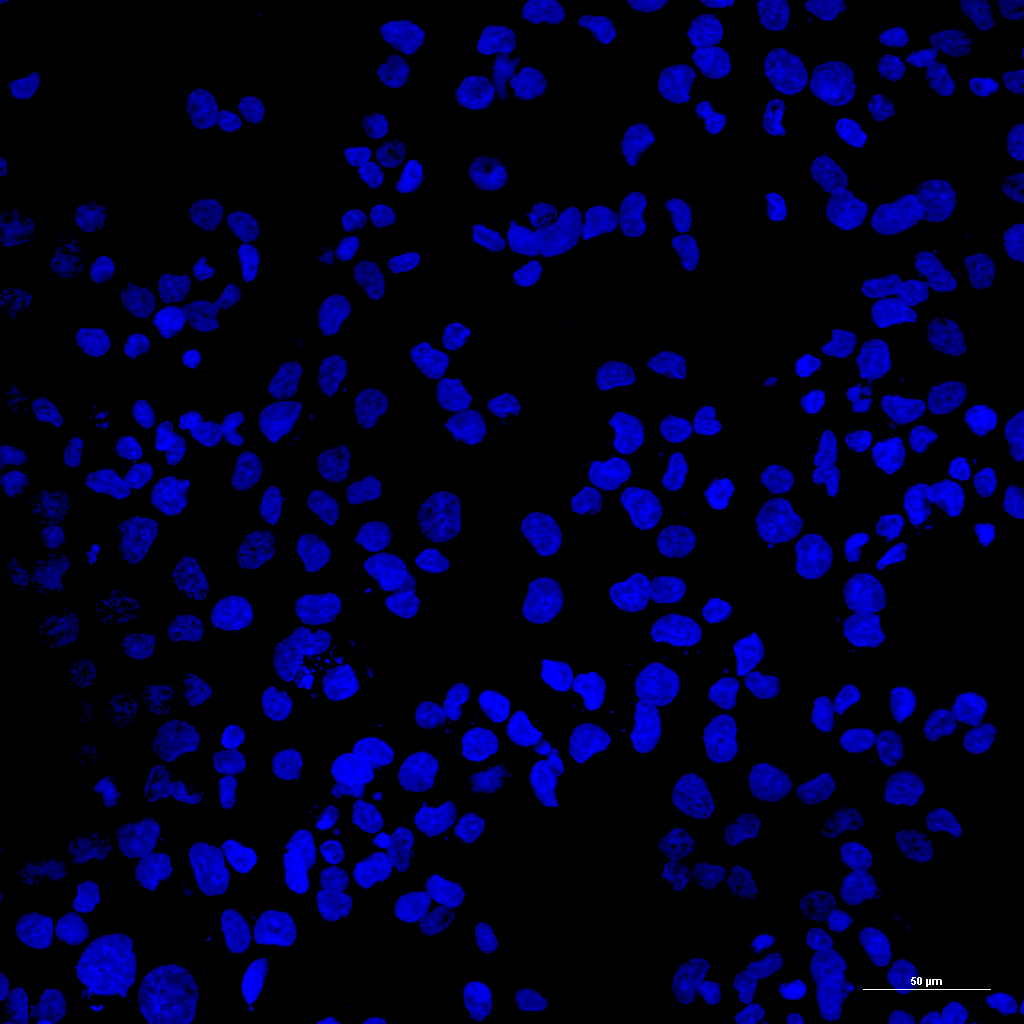

Supplement: Supplementary file 6 [file DataSheet6.zip › EDU/HCT116-EDU/+OXA/14_RGB_DAPI.tif]

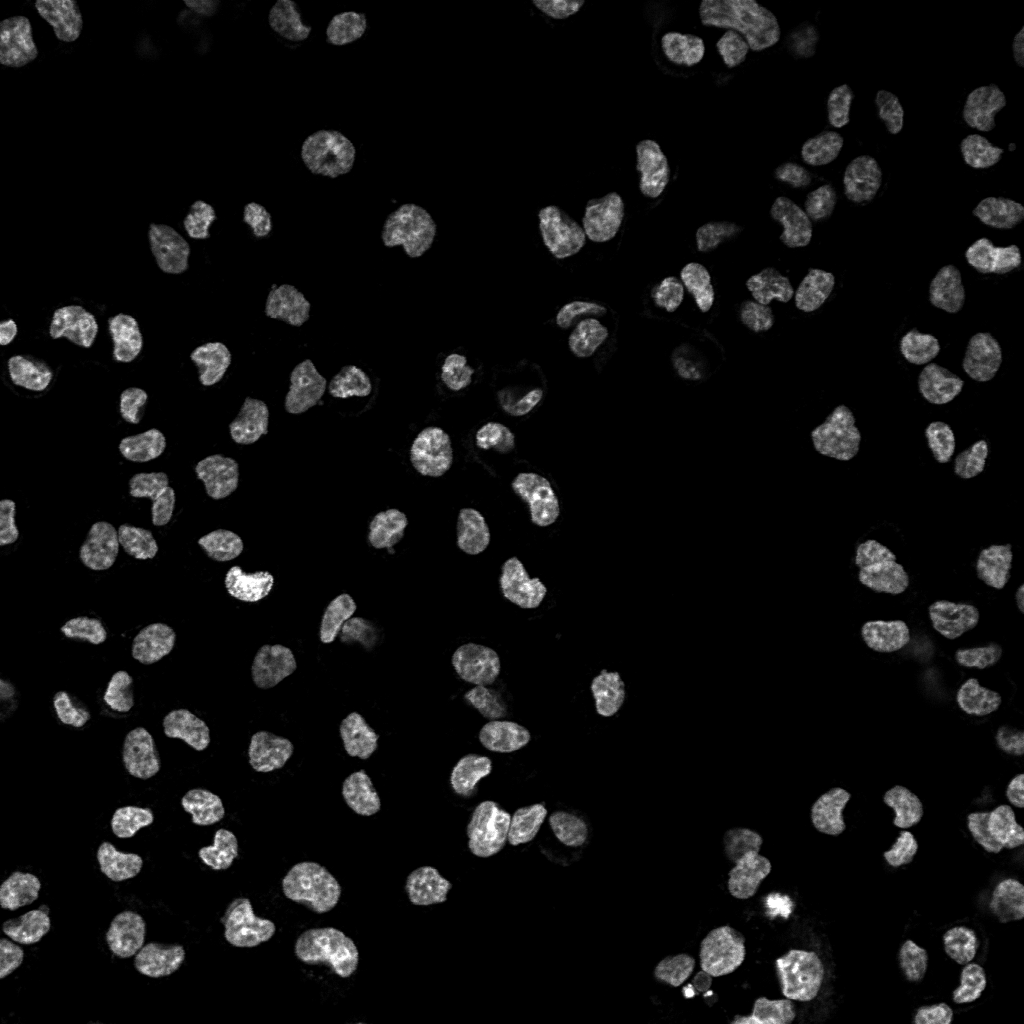

Supplement: Supplementary file 6 [file DataSheet6.zip › EDU/HCT116-EDU/+OXA+OE/3.tif]

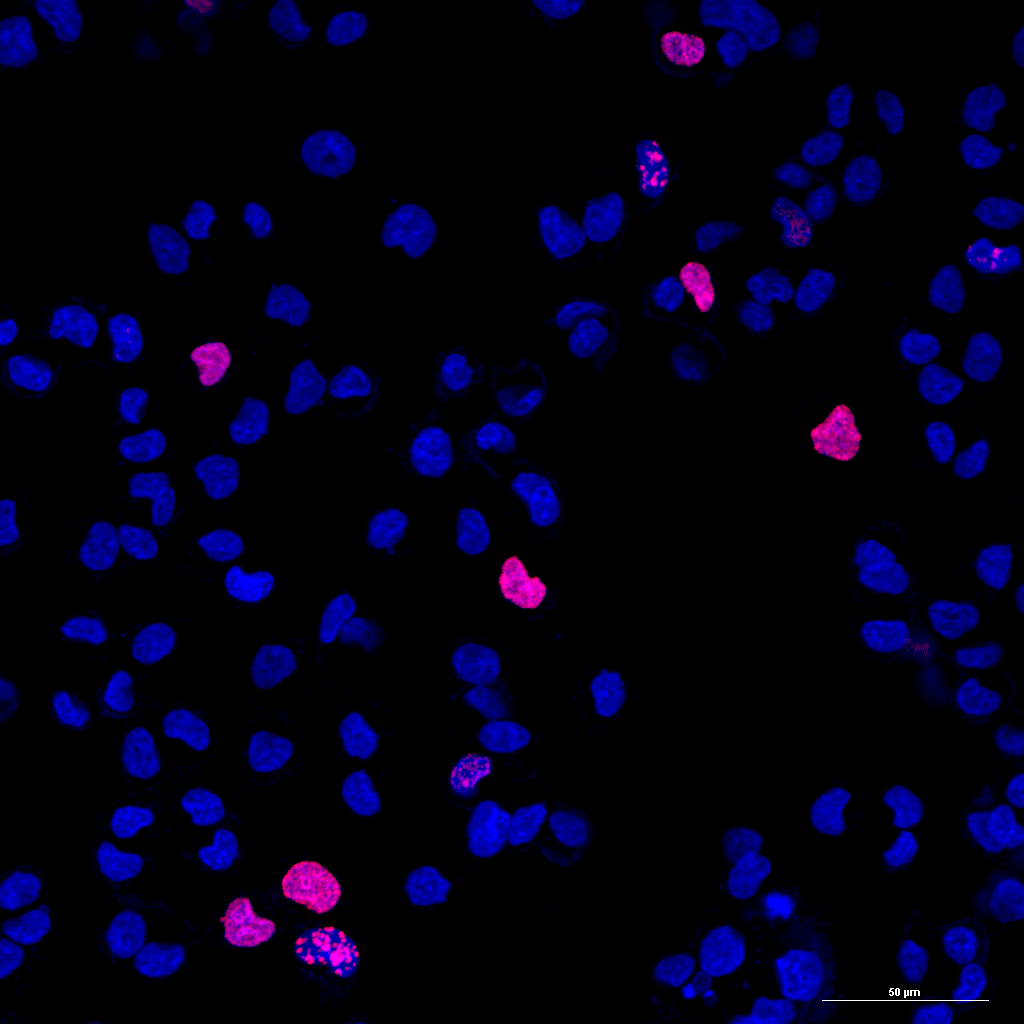

Supplement: Supplementary file 6 [file DataSheet6.zip › EDU/HCT116-EDU/+OXA+OE/3_RGB.tif]

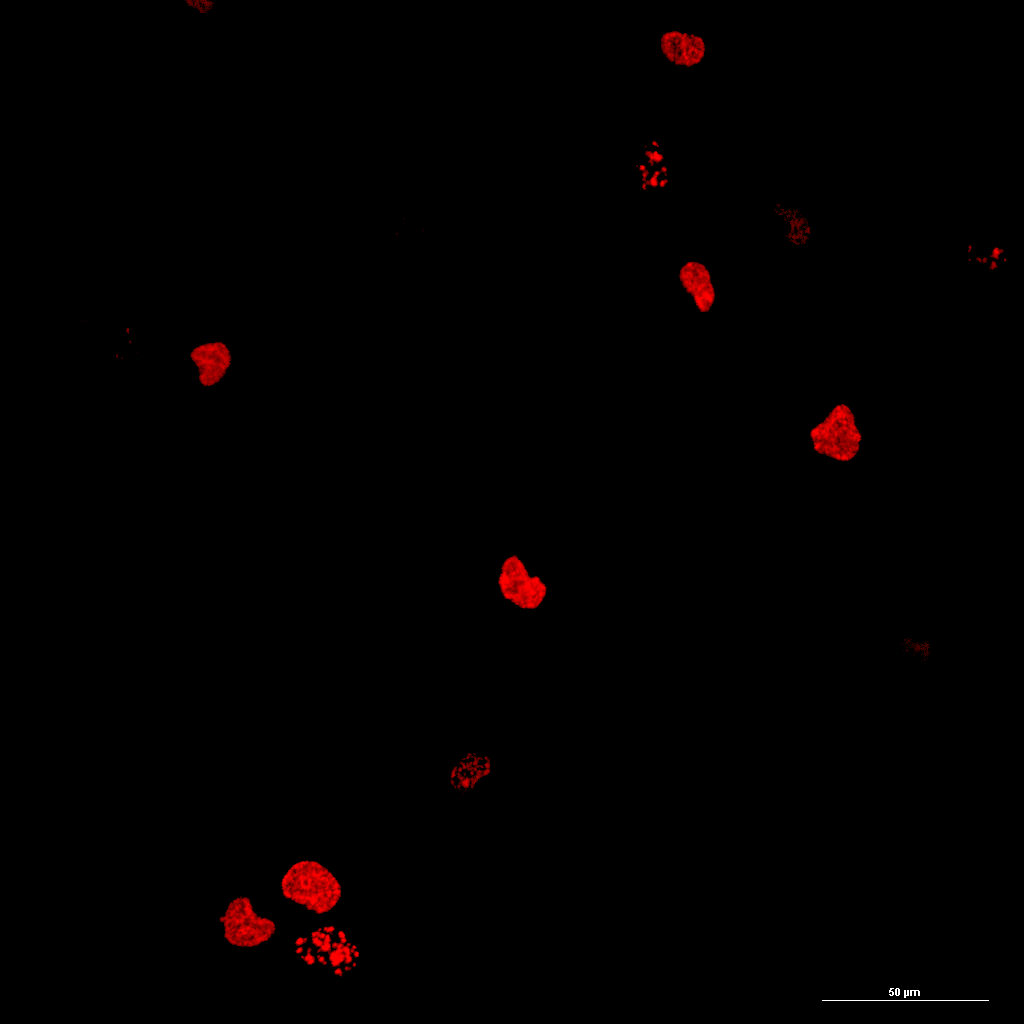

Supplement: Supplementary file 6 [file DataSheet6.zip › EDU/HCT116-EDU/+OXA+OE/3_RGB_Alexa Fluor 594 cadaverine_H2O.tif]

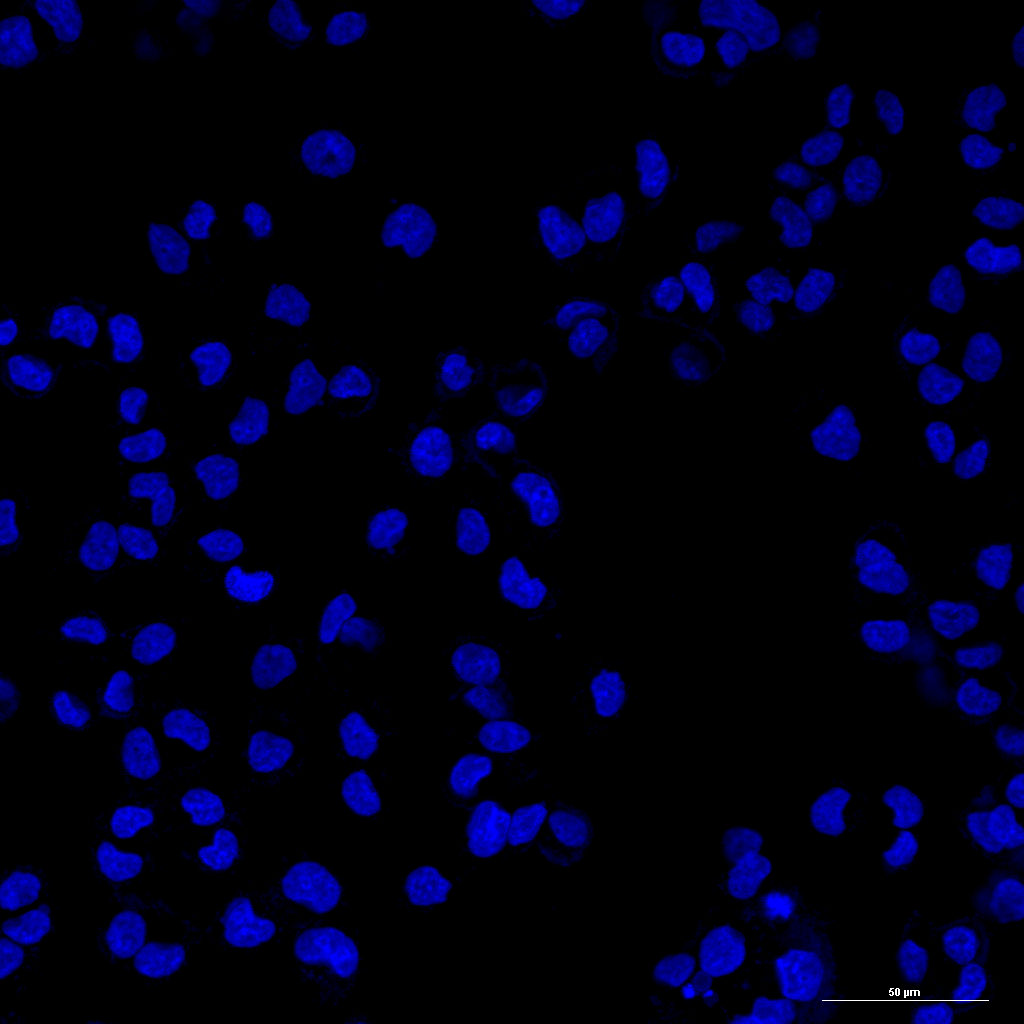

Supplement: Supplementary file 6 [file DataSheet6.zip › EDU/HCT116-EDU/+OXA+OE/3_RGB_DAPI.tif]

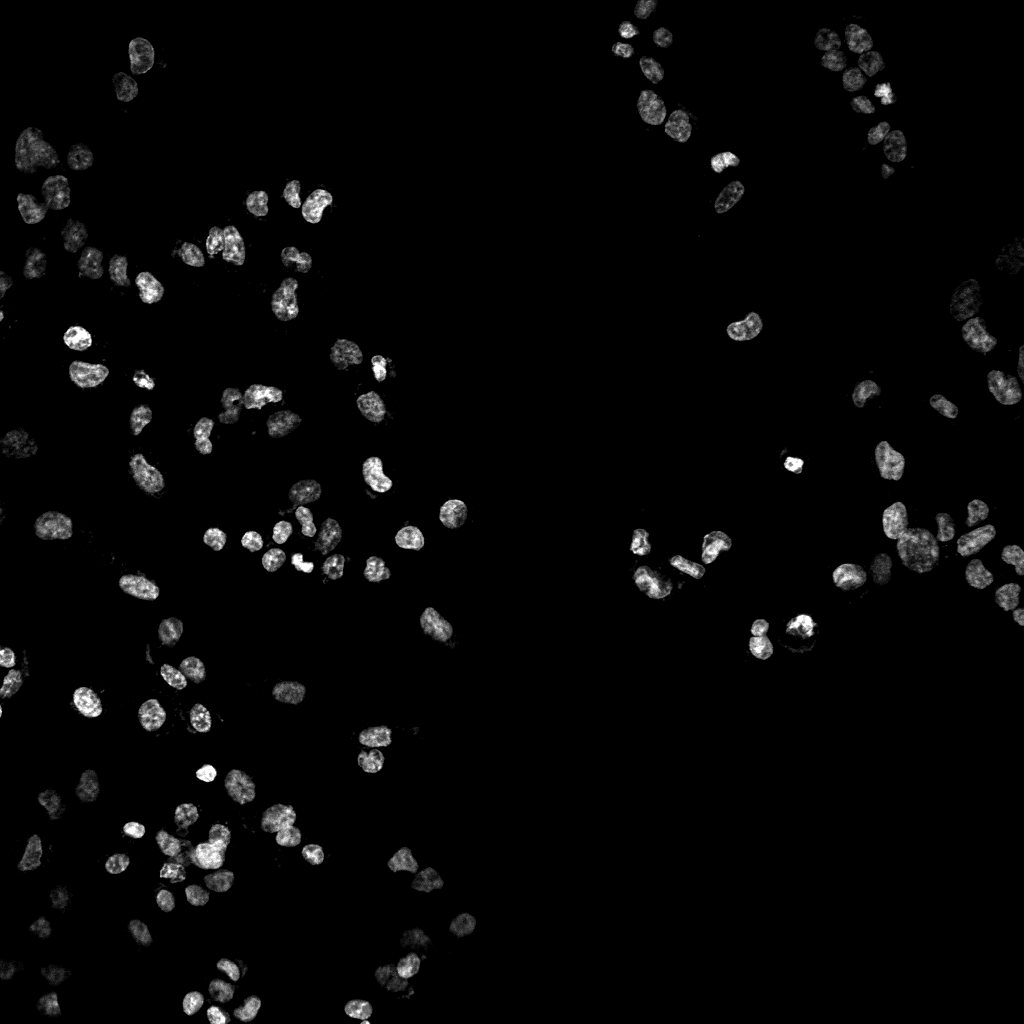

Supplement: Supplementary file 6 [file DataSheet6.zip › EDU/HCT116-EDU/OE/19.tif]

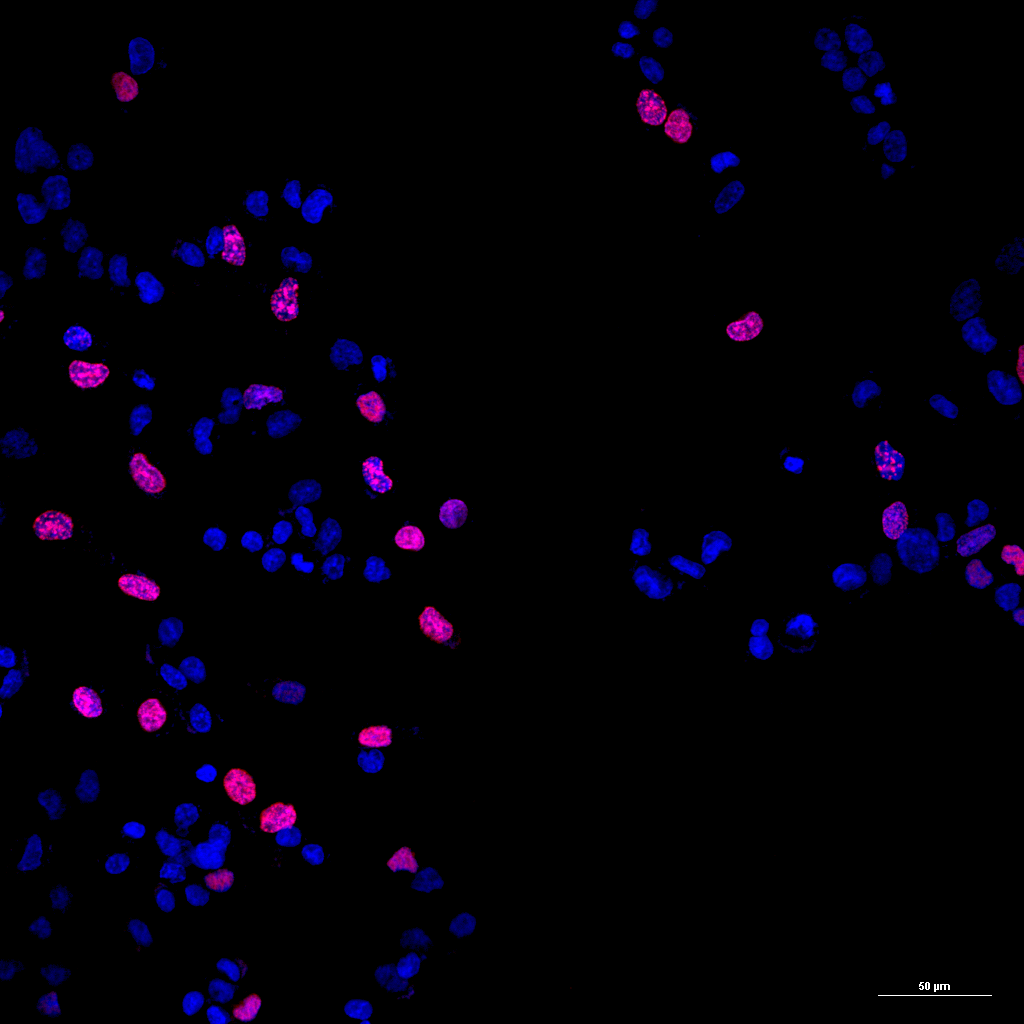

Supplement: Supplementary file 6 [file DataSheet6.zip › EDU/HCT116-EDU/OE/19_RGB.tif]

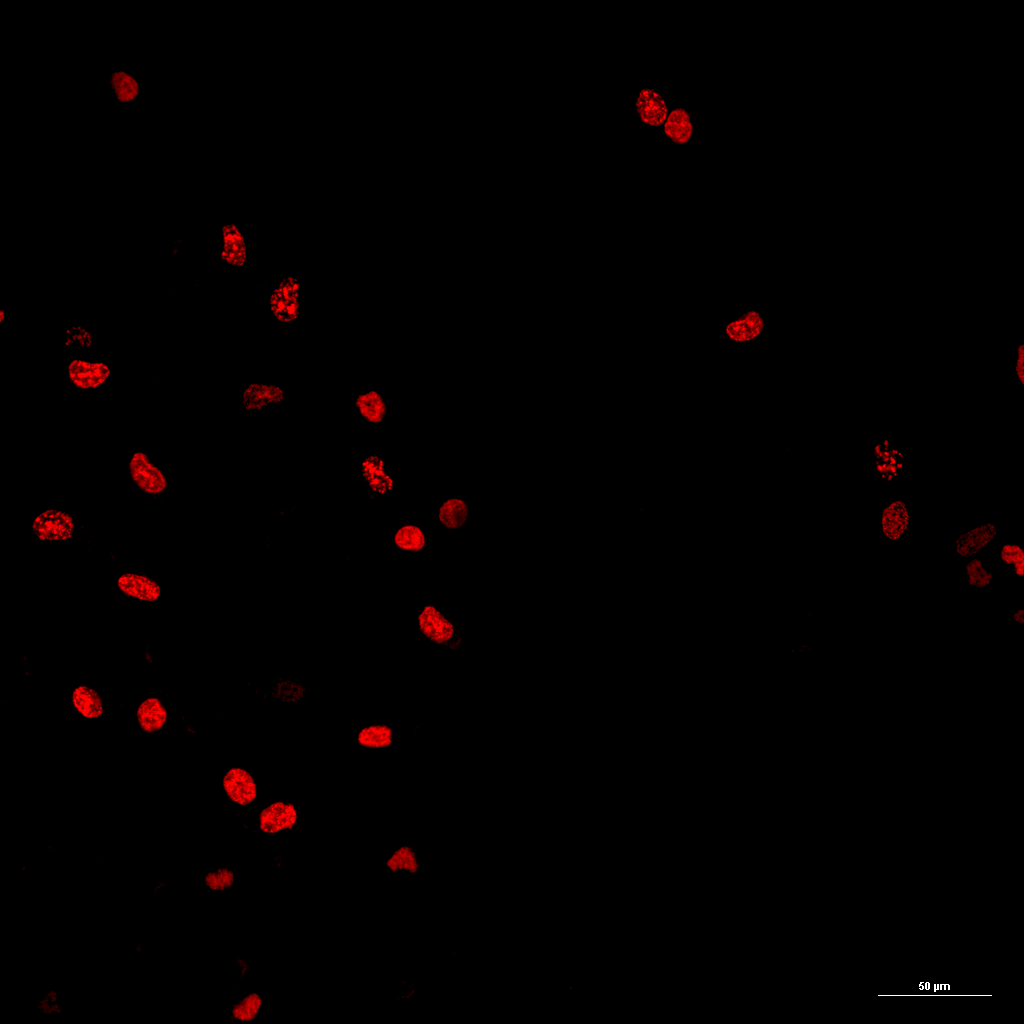

Supplement: Supplementary file 6 [file DataSheet6.zip › EDU/HCT116-EDU/OE/19_RGB_Alexa Fluor 594 cadaverine_H2O.tif]

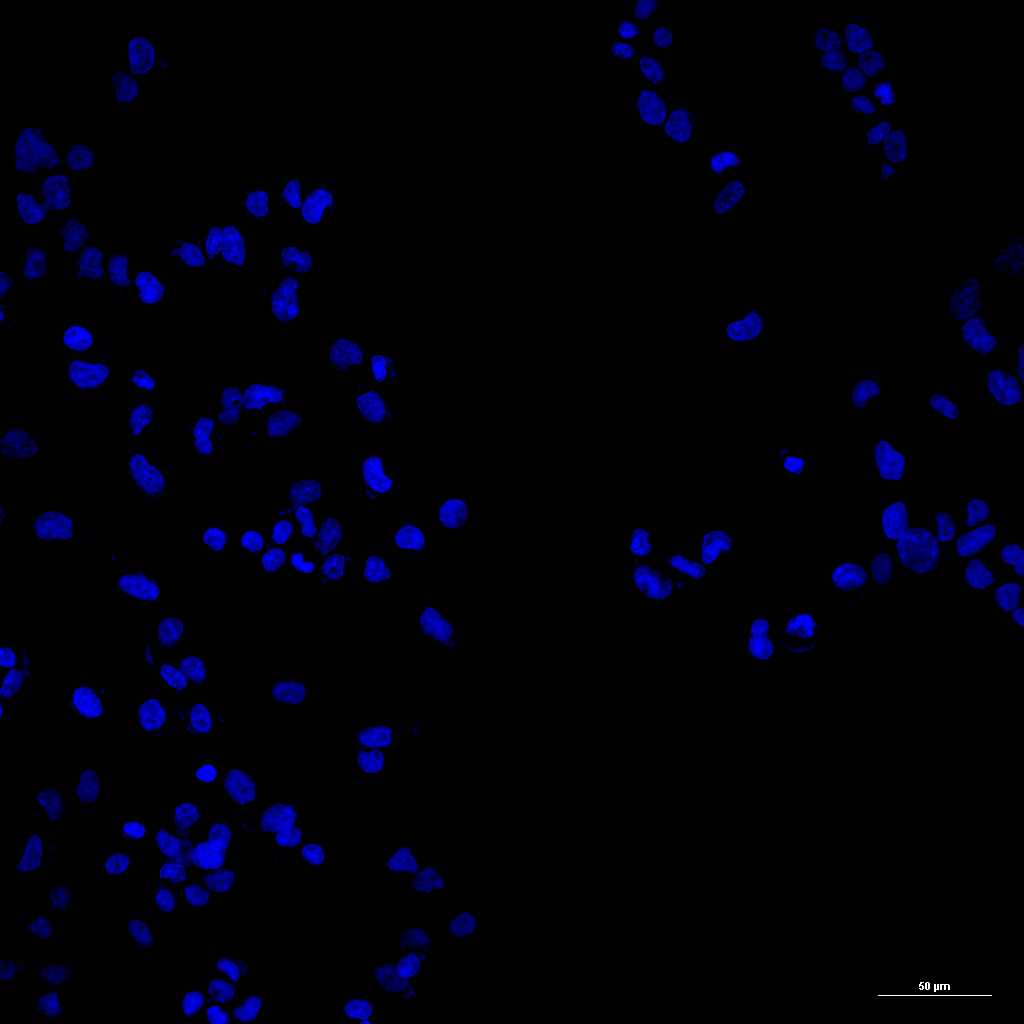

Supplement: Supplementary file 6 [file DataSheet6.zip › EDU/HCT116-EDU/OE/19_RGB_DAPI.tif]

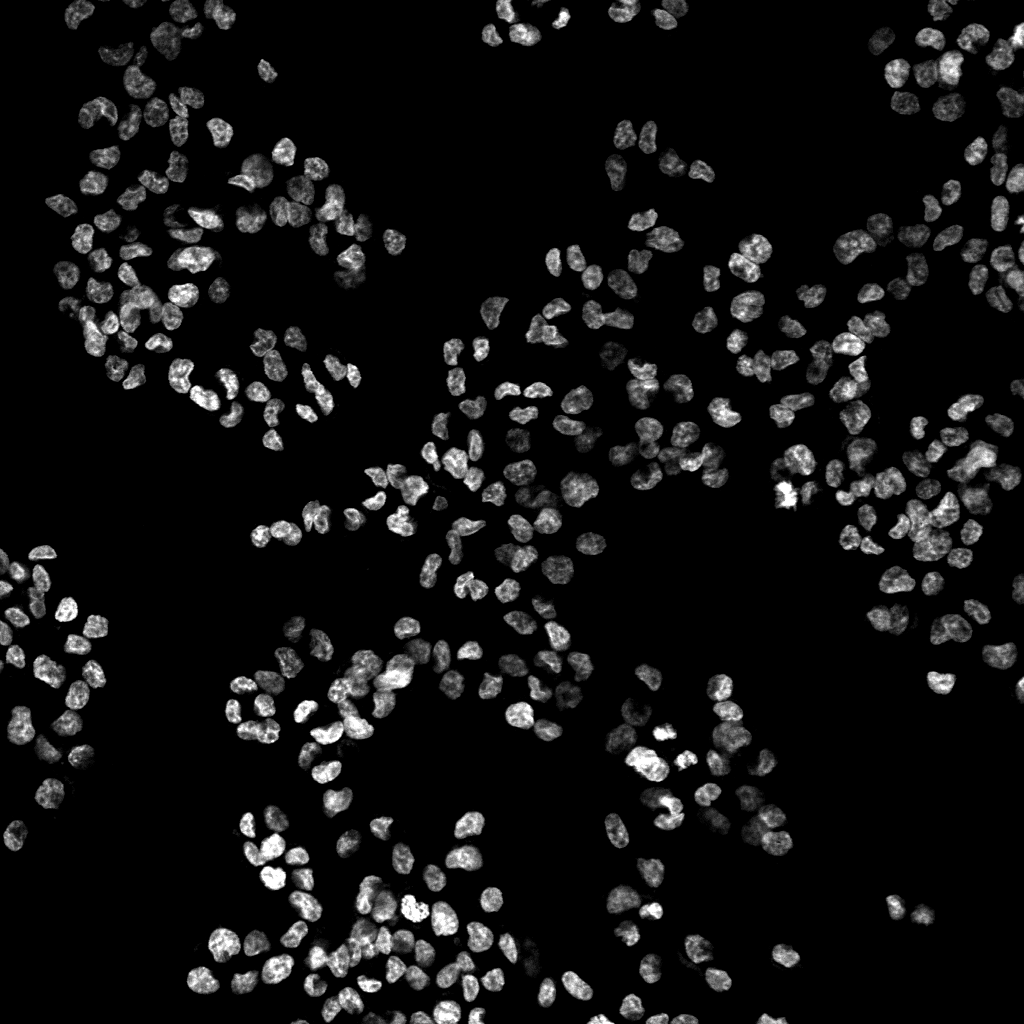

Supplement: Supplementary file 6 [file DataSheet6.zip › EDU/HCT116-EDU/Vector/7.tif]

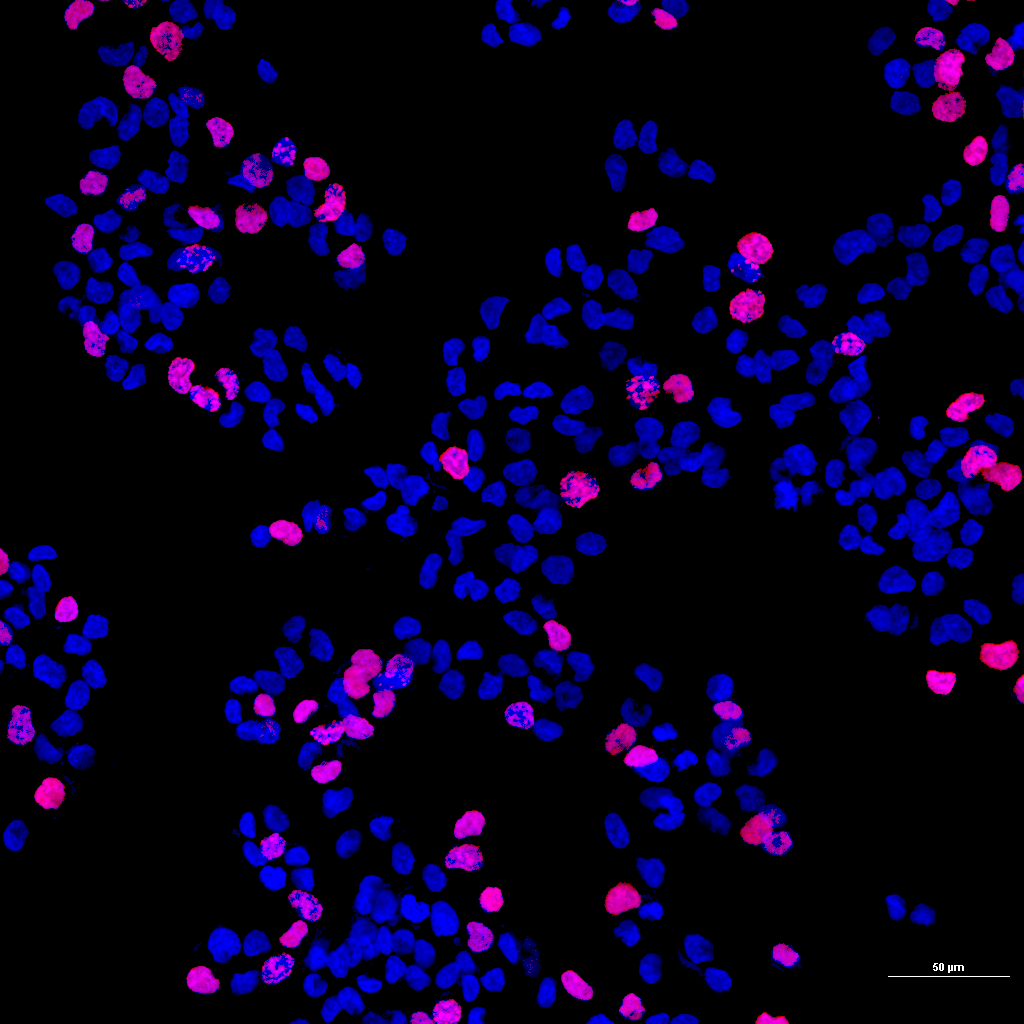

Supplement: Supplementary file 6 [file DataSheet6.zip › EDU/HCT116-EDU/Vector/7_RGB.tif]

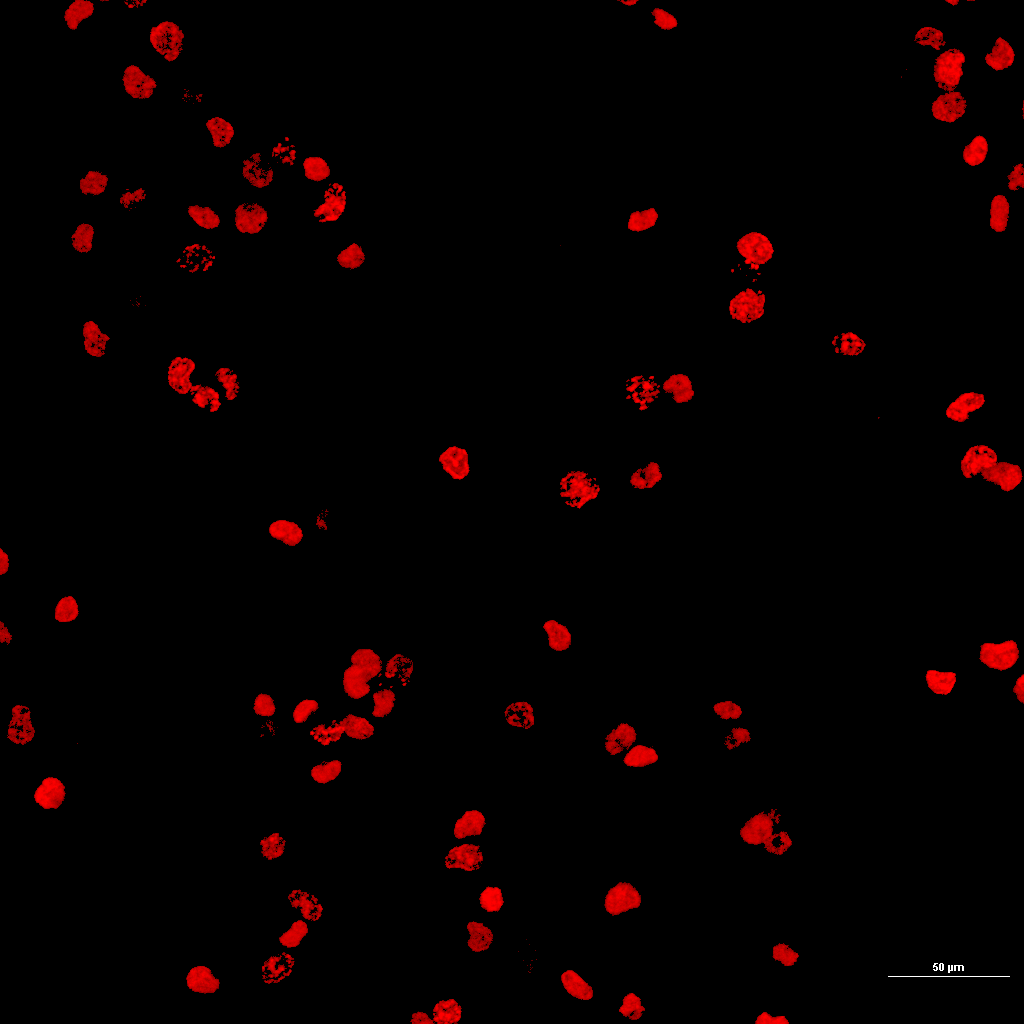

Supplement: Supplementary file 6 [file DataSheet6.zip › EDU/HCT116-EDU/Vector/7_RGB_Alexa Fluor 594 cadaverine_H2O.tif]

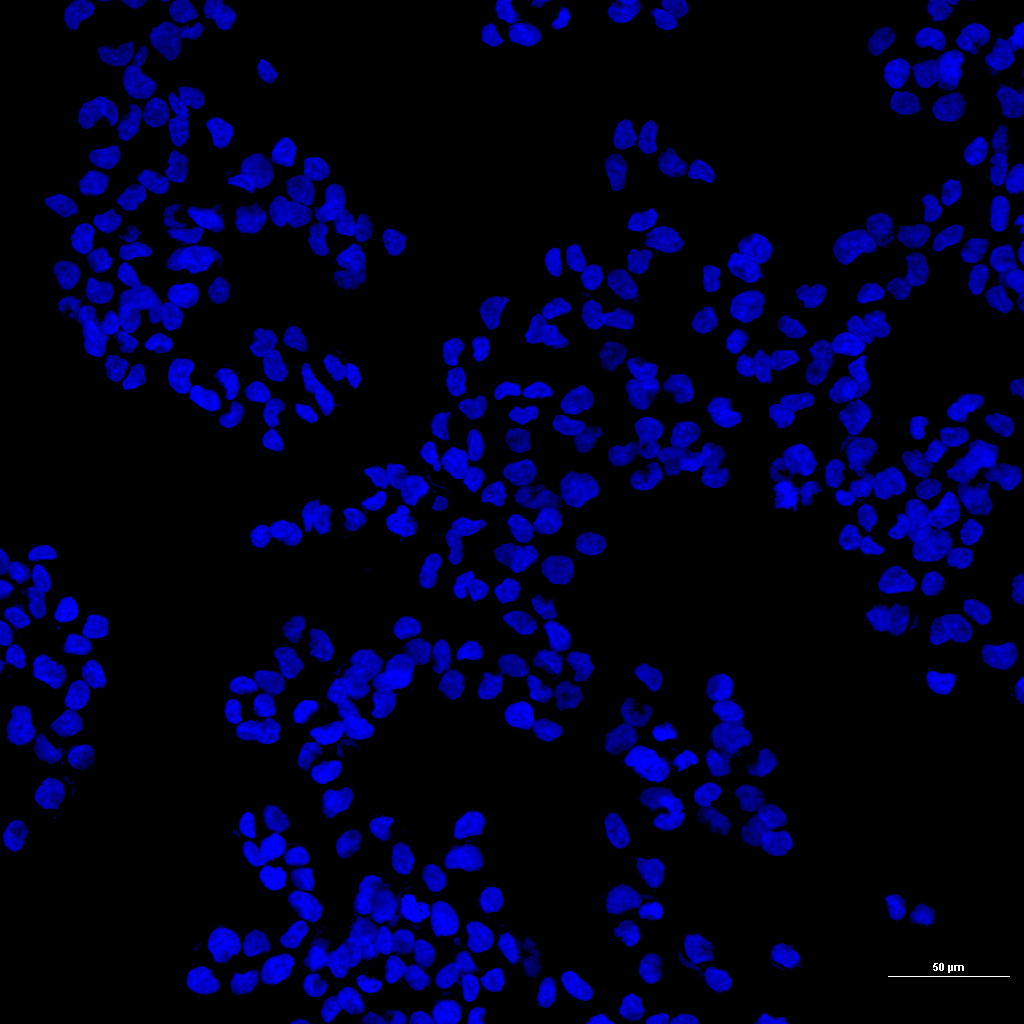

Supplement: Supplementary file 6 [file DataSheet6.zip › EDU/HCT116-EDU/Vector/7_RGB_DAPI.tif]

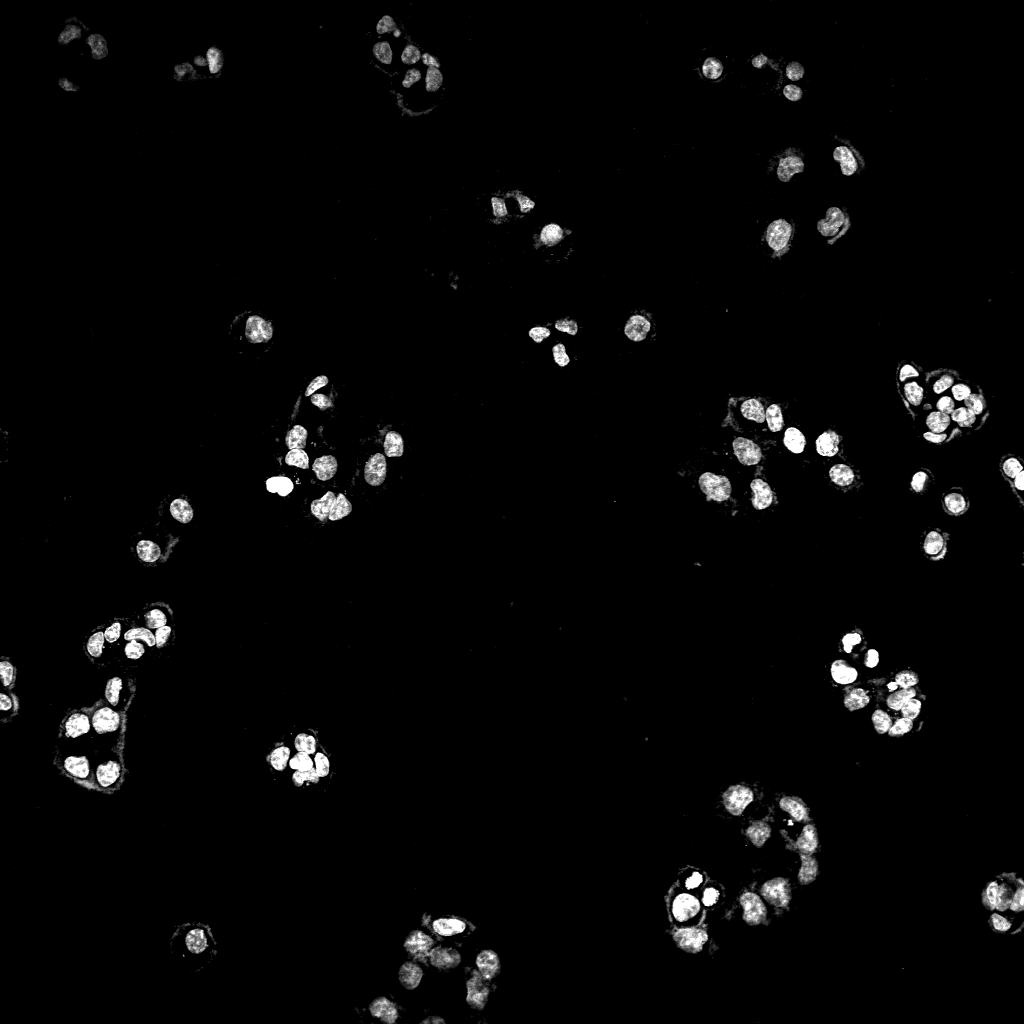

Supplement: Supplementary file 6 [file DataSheet6.zip › EDU/SW480-EDU/+OXA/6.tif]

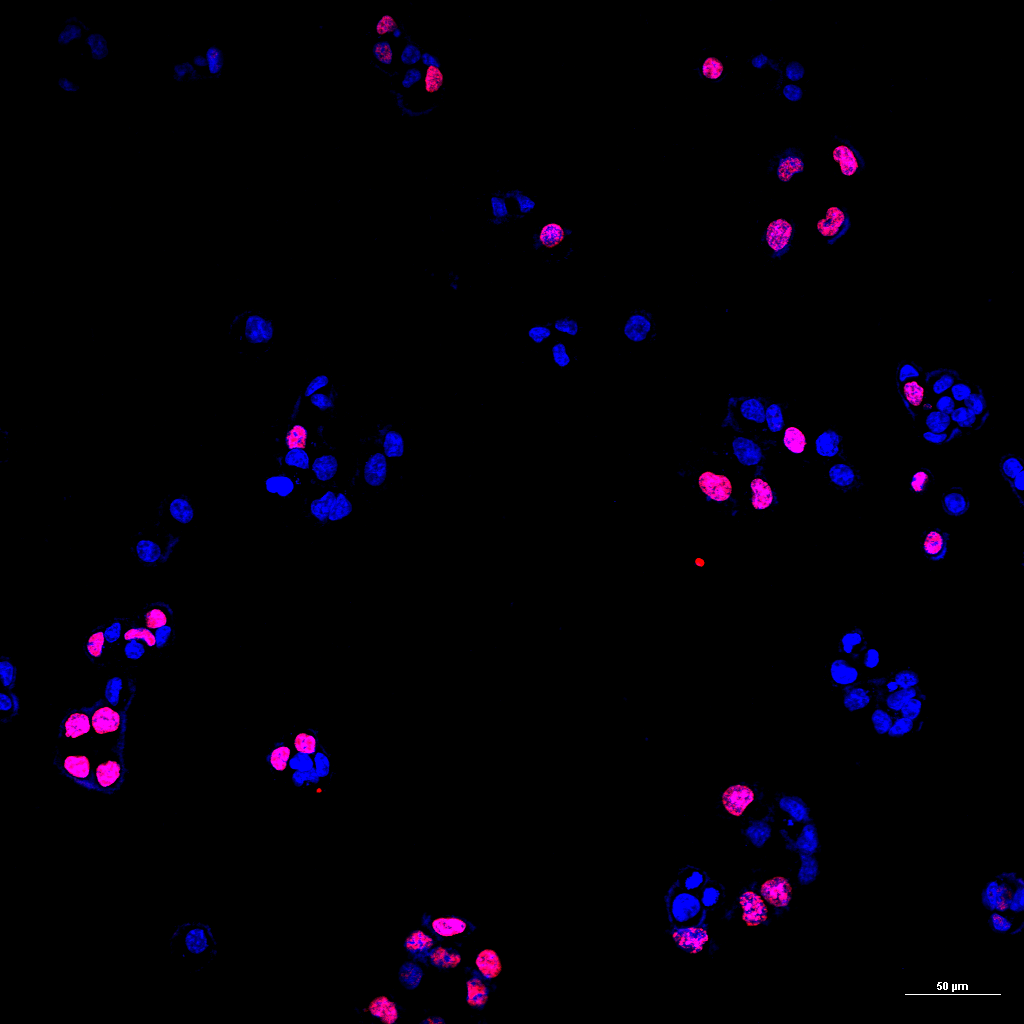

Supplement: Supplementary file 6 [file DataSheet6.zip › EDU/SW480-EDU/+OXA/6_RGB.tif]

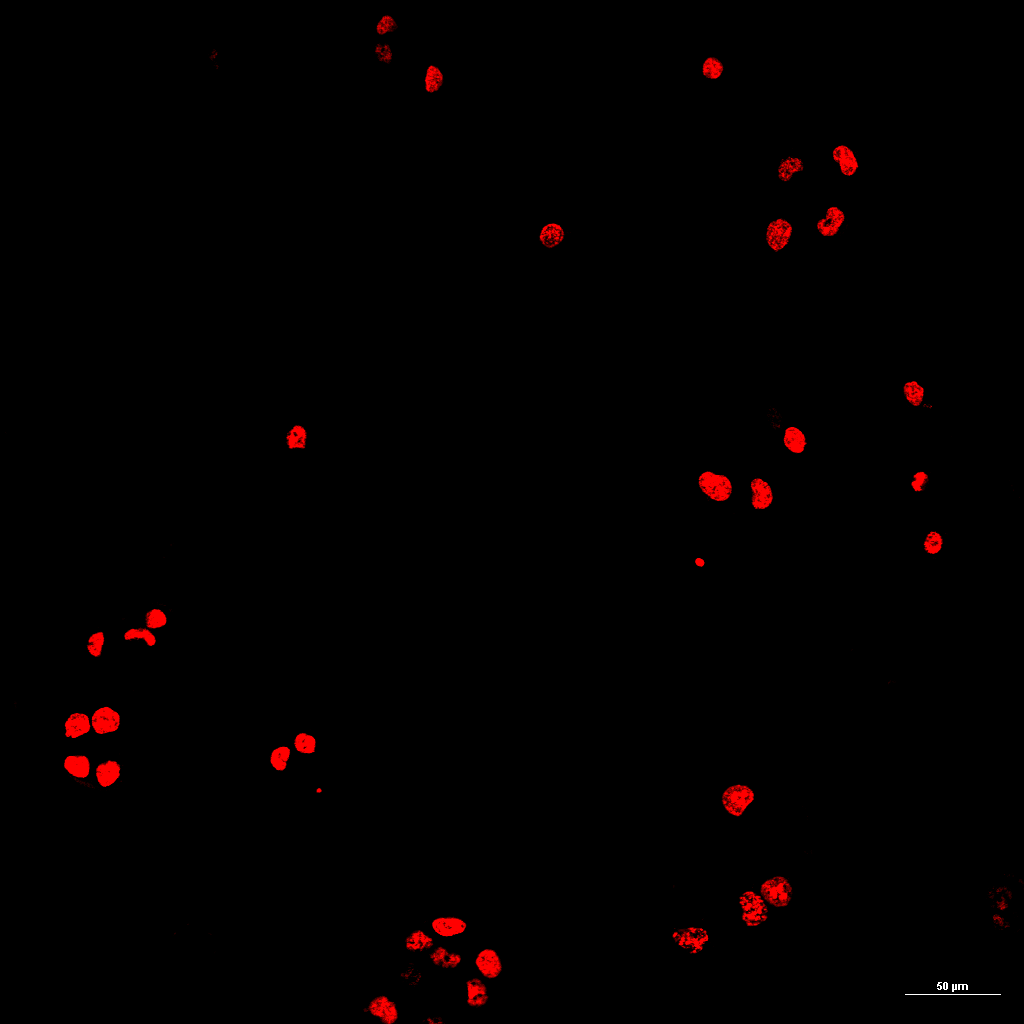

Supplement: Supplementary file 6 [file DataSheet6.zip › EDU/SW480-EDU/+OXA/6_RGB_Alexa Fluor 594 cadaverine_H2O.tif]

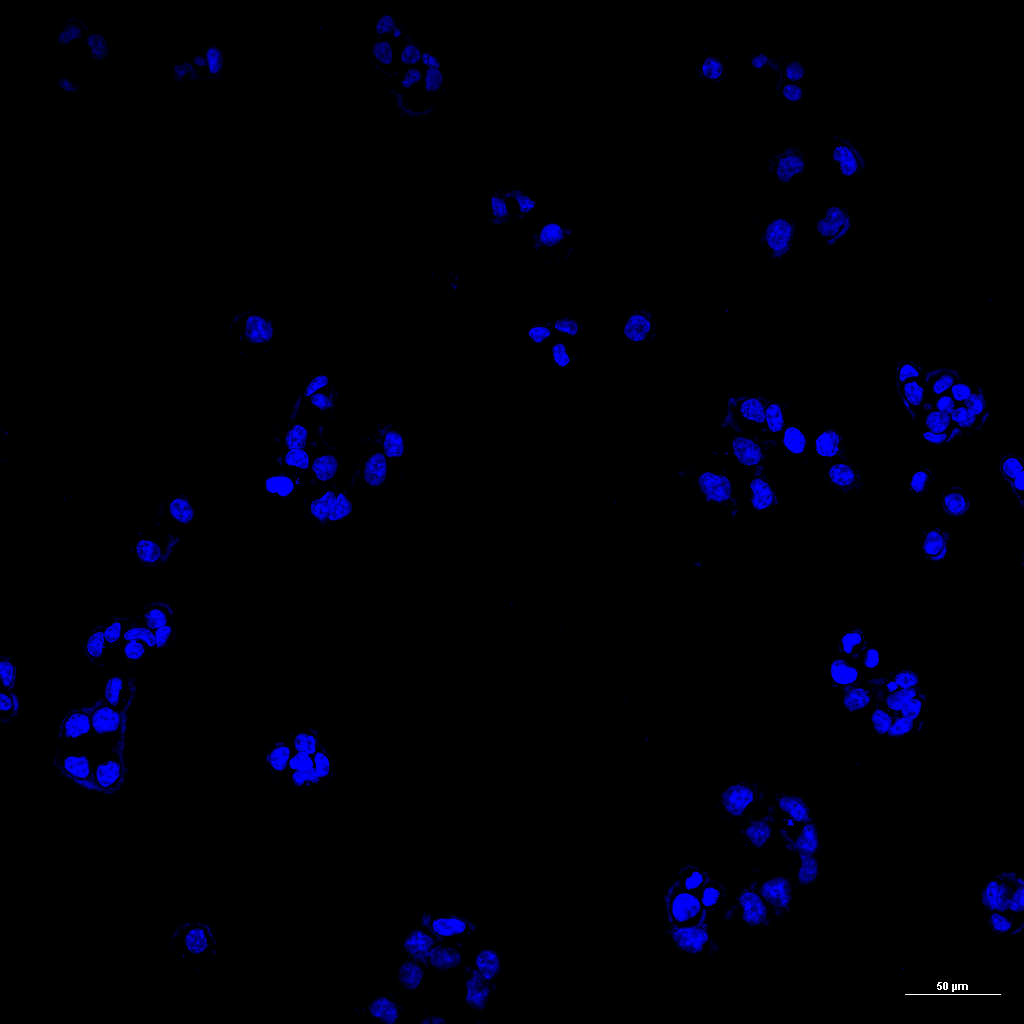

Supplement: Supplementary file 6 [file DataSheet6.zip › EDU/SW480-EDU/+OXA/6_RGB_DAPI.tif]

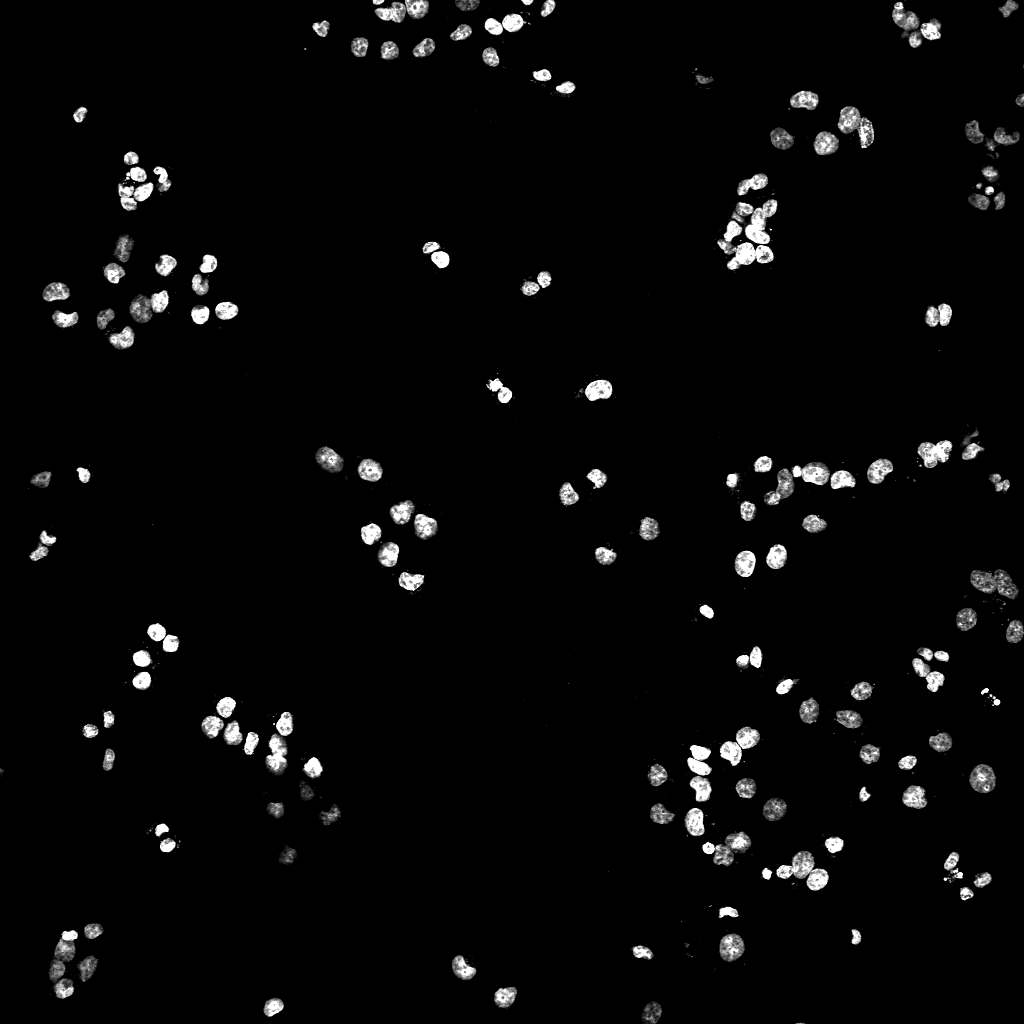

Supplement: Supplementary file 6 [file DataSheet6.zip › EDU/SW480-EDU/+OXA+OE/3.tif]

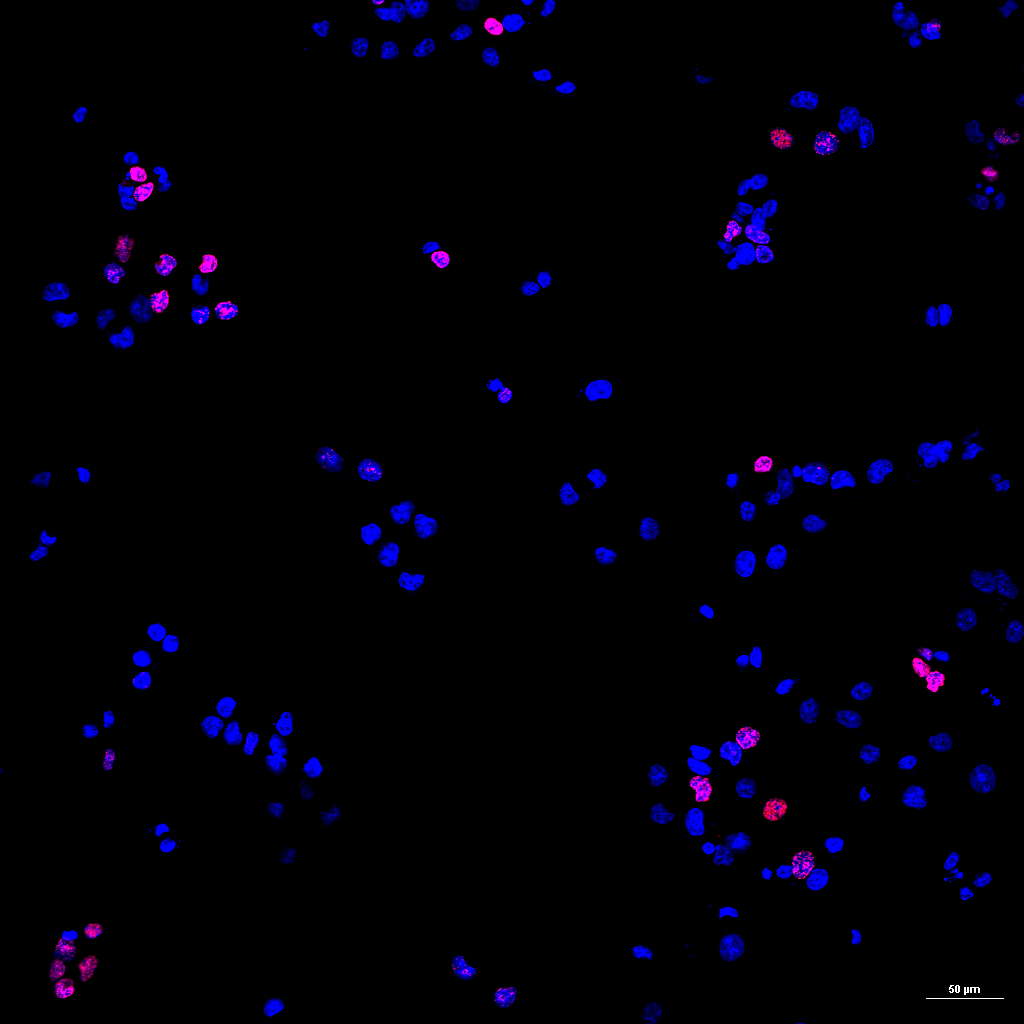

Supplement: Supplementary file 6 [file DataSheet6.zip › EDU/SW480-EDU/+OXA+OE/3_RGB.tif]

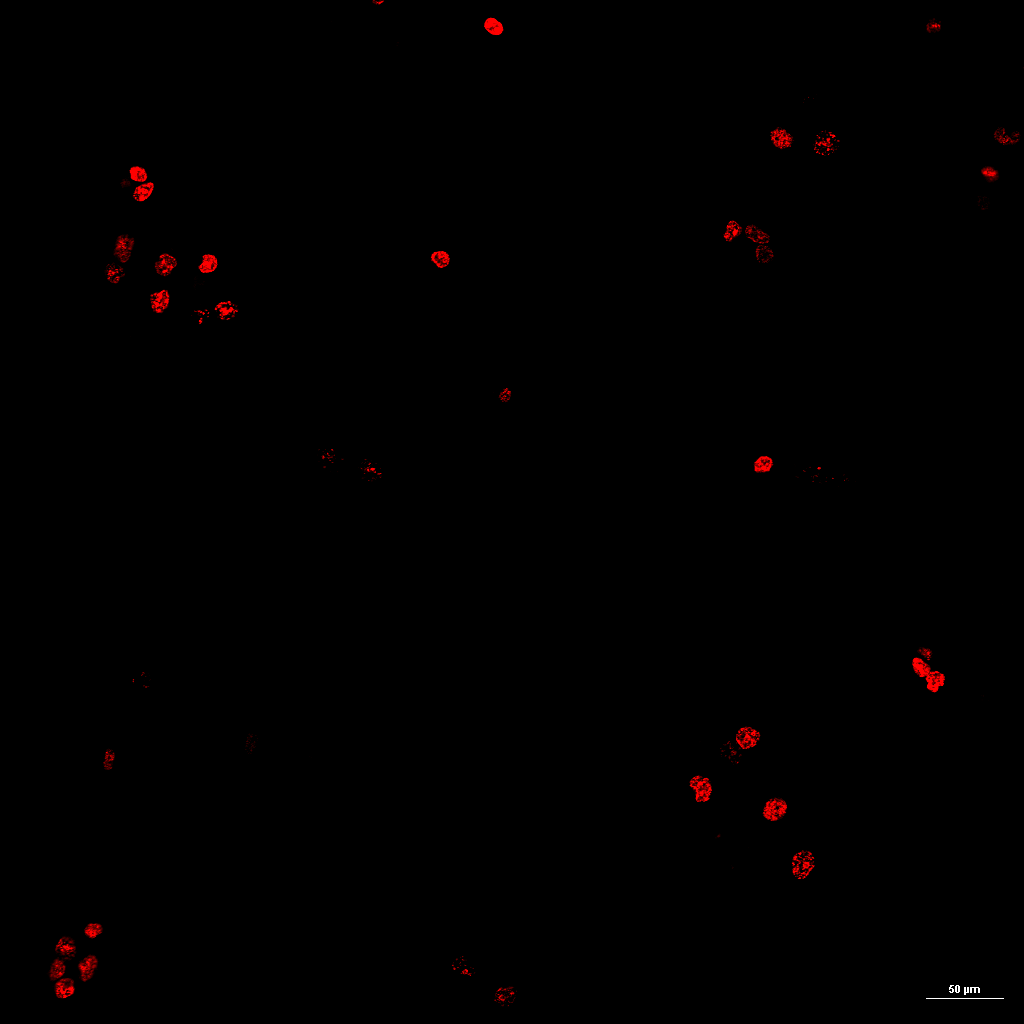

Supplement: Supplementary file 6 [file DataSheet6.zip › EDU/SW480-EDU/+OXA+OE/3_RGB_Alexa Fluor 594 cadaverine_H2O.tif]

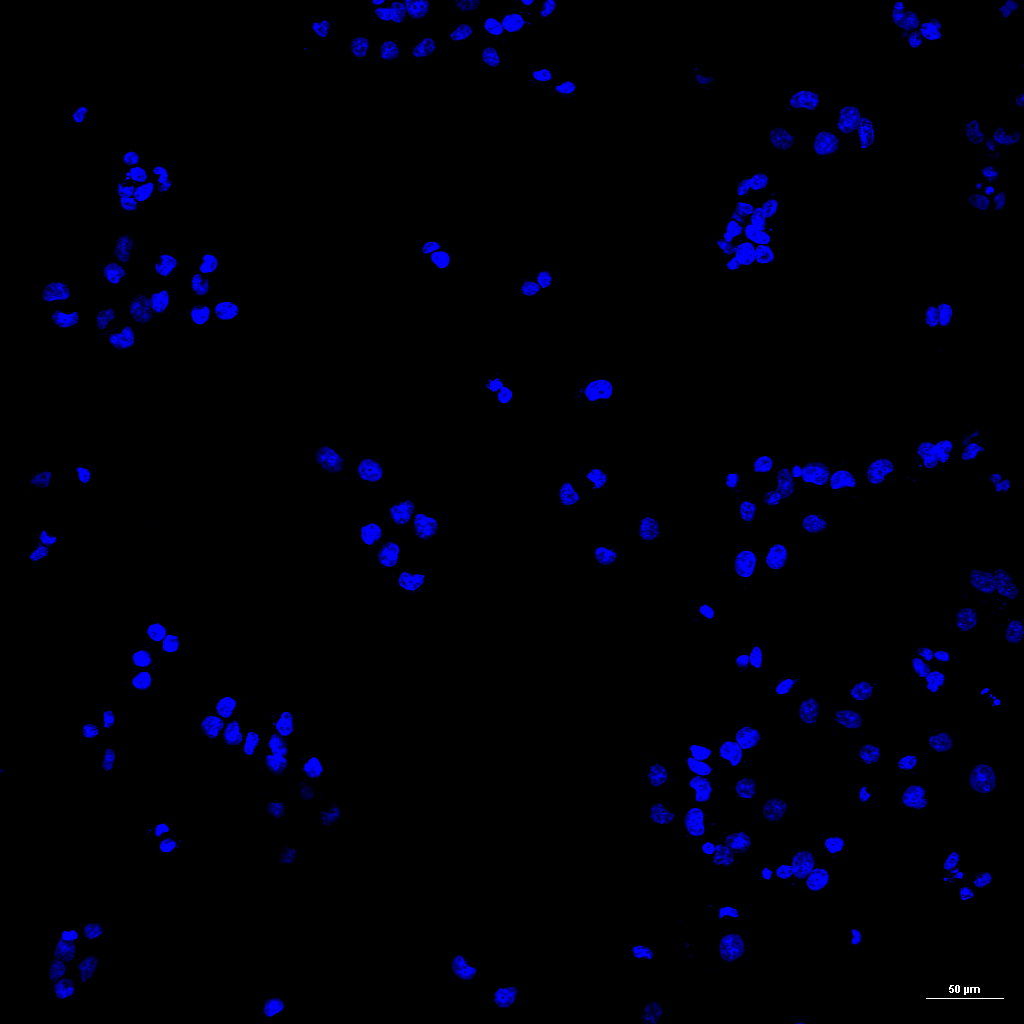

Supplement: Supplementary file 6 [file DataSheet6.zip › EDU/SW480-EDU/+OXA+OE/3_RGB_DAPI.tif]

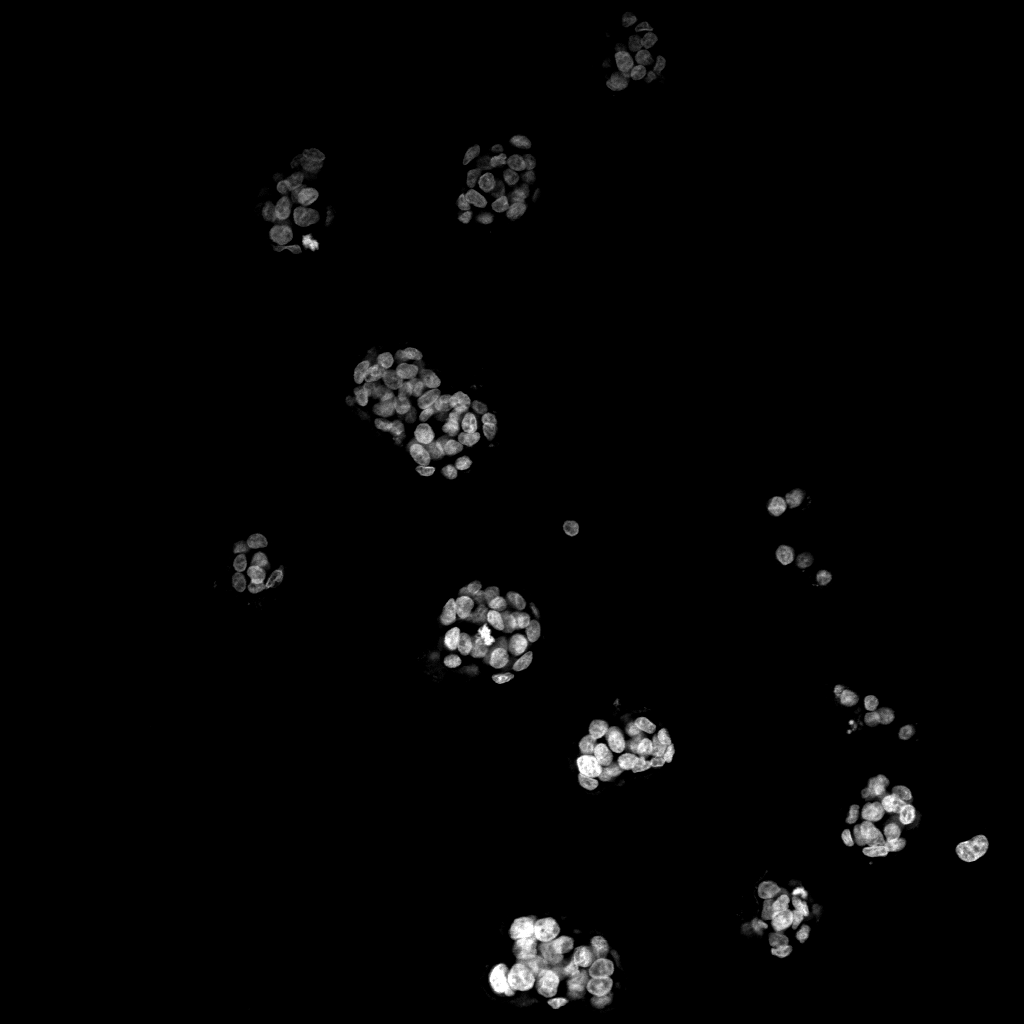

Supplement: Supplementary file 6 [file DataSheet6.zip › EDU/SW480-EDU/OE/3.tif]

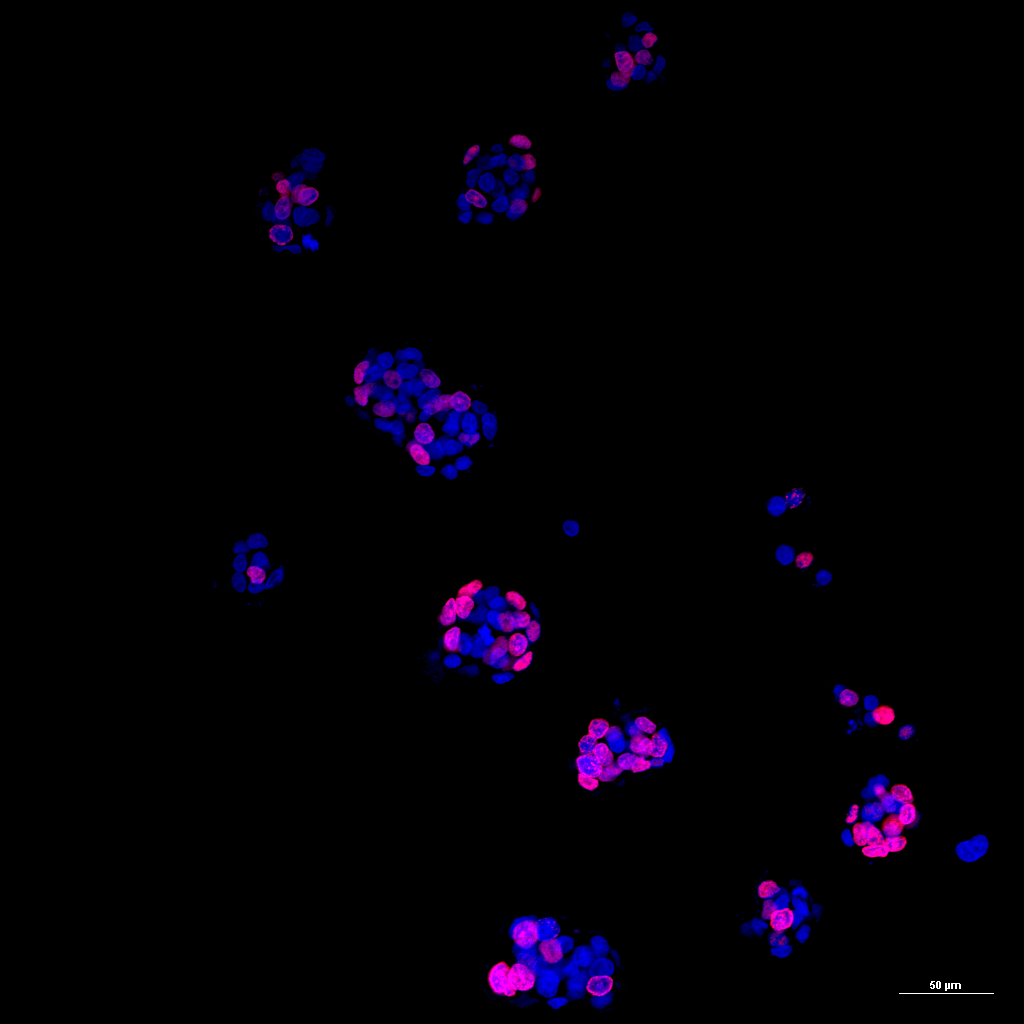

Supplement: Supplementary file 6 [file DataSheet6.zip › EDU/SW480-EDU/OE/3_RGB.tif]

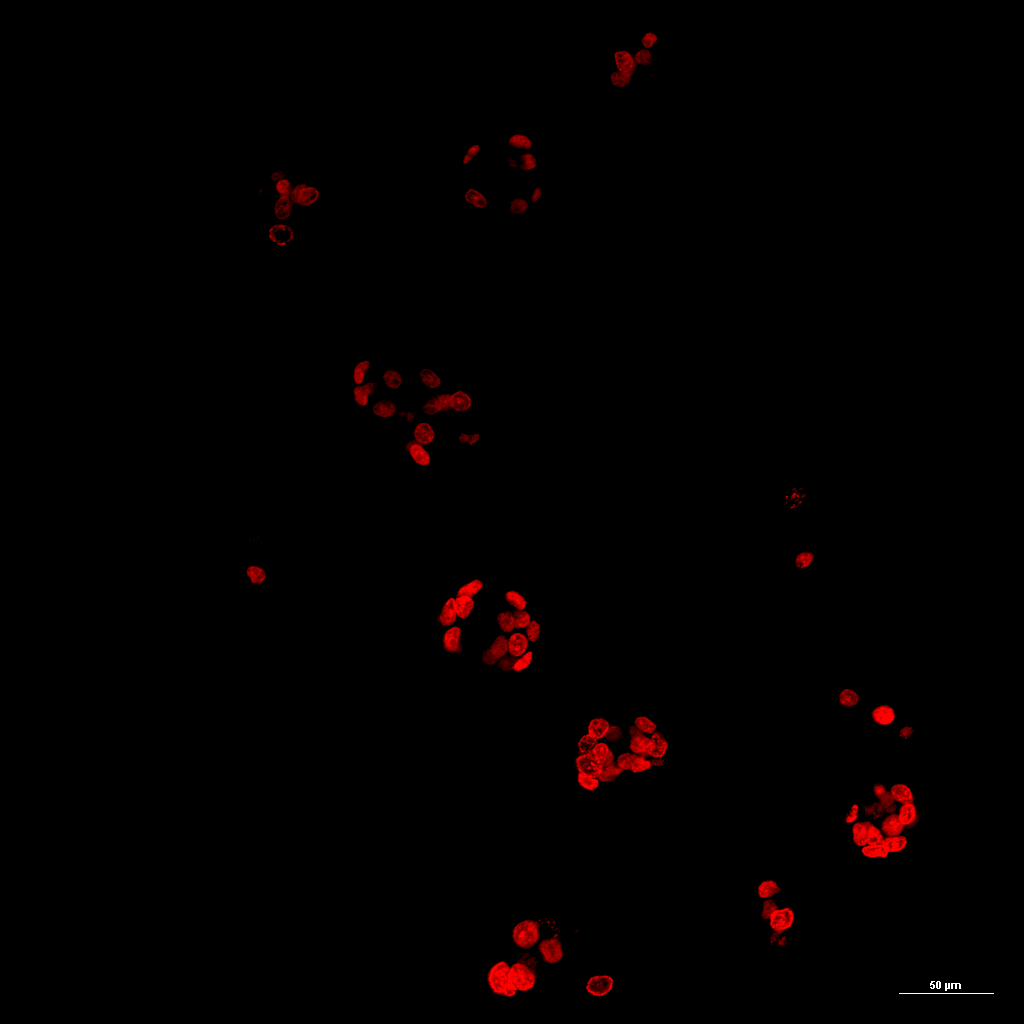

Supplement: Supplementary file 6 [file DataSheet6.zip › EDU/SW480-EDU/OE/3_RGB_Alexa Fluor 594 cadaverine_H2O.tif]

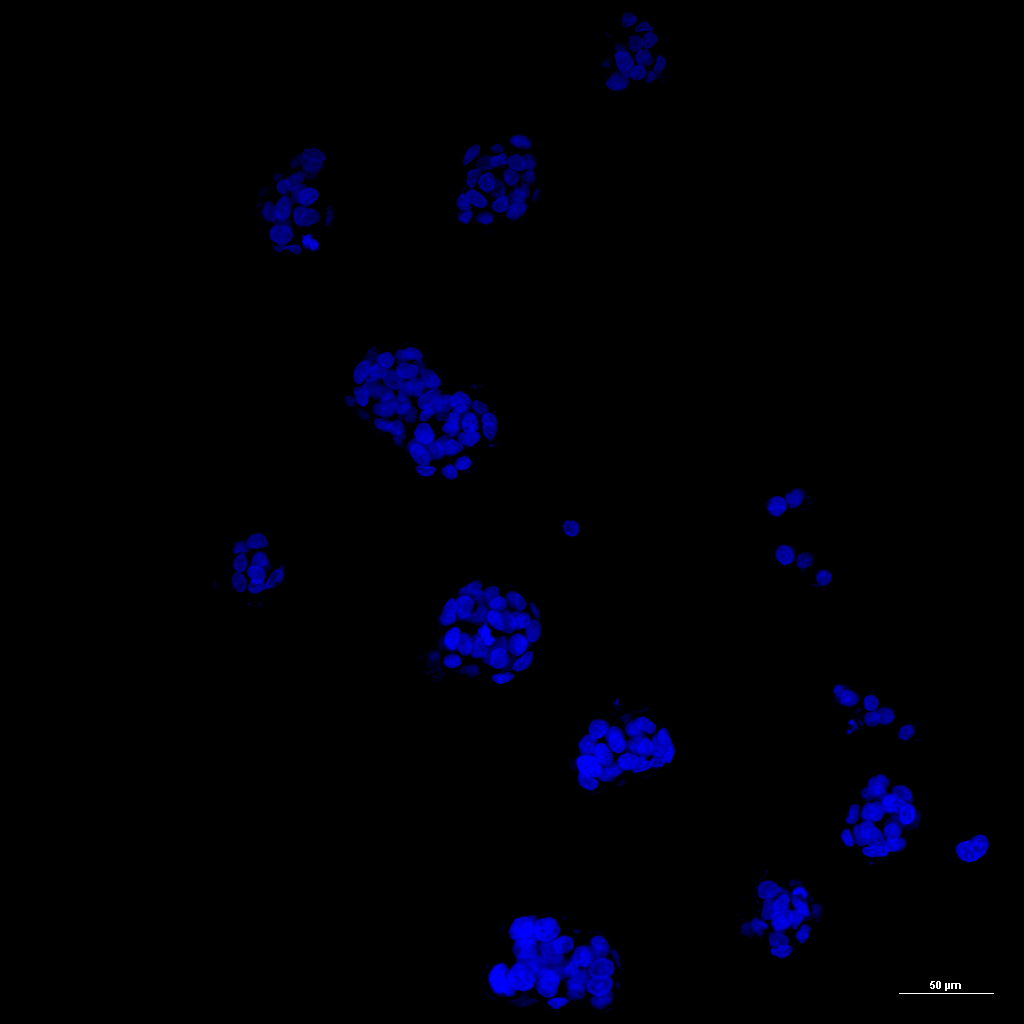

Supplement: Supplementary file 6 [file DataSheet6.zip › EDU/SW480-EDU/OE/3_RGB_DAPI.tif]

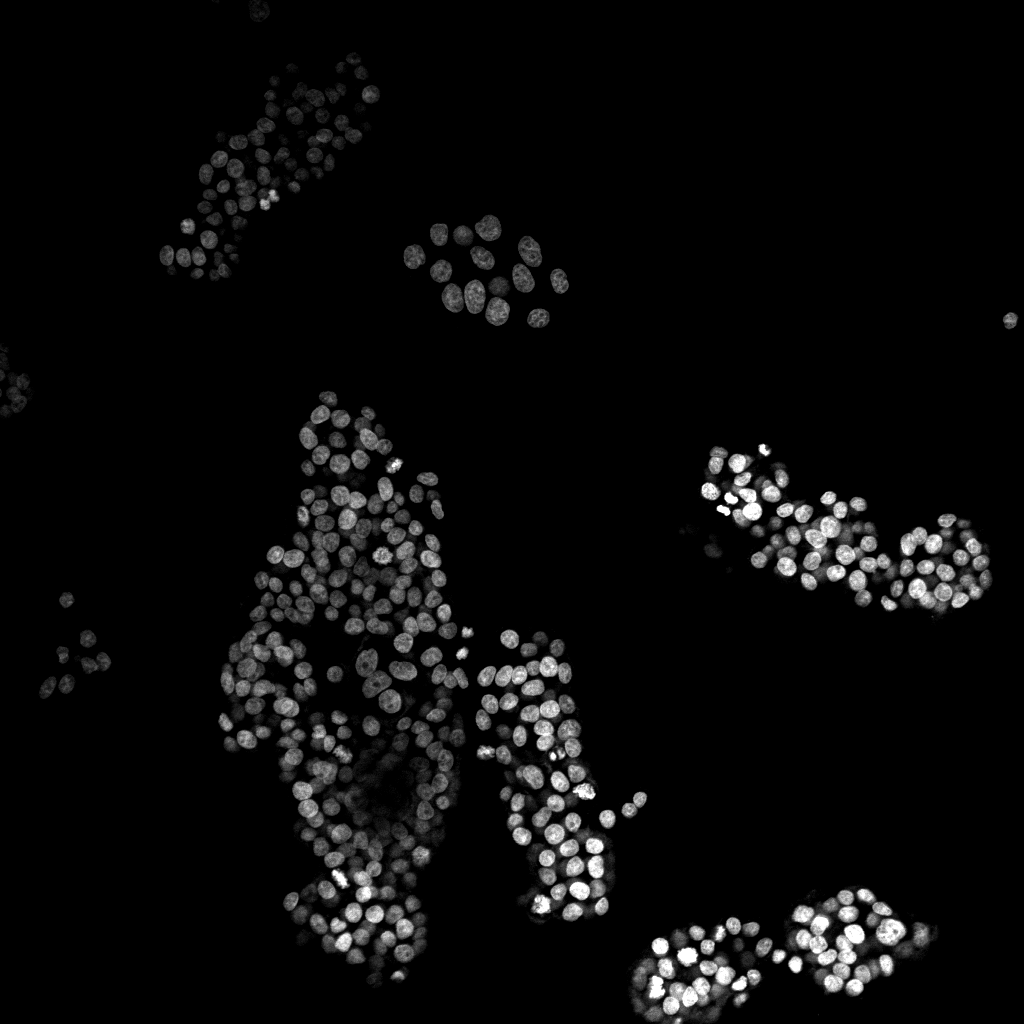

Supplement: Supplementary file 6 [file DataSheet6.zip › EDU/SW480-EDU/Vector/4.tif]

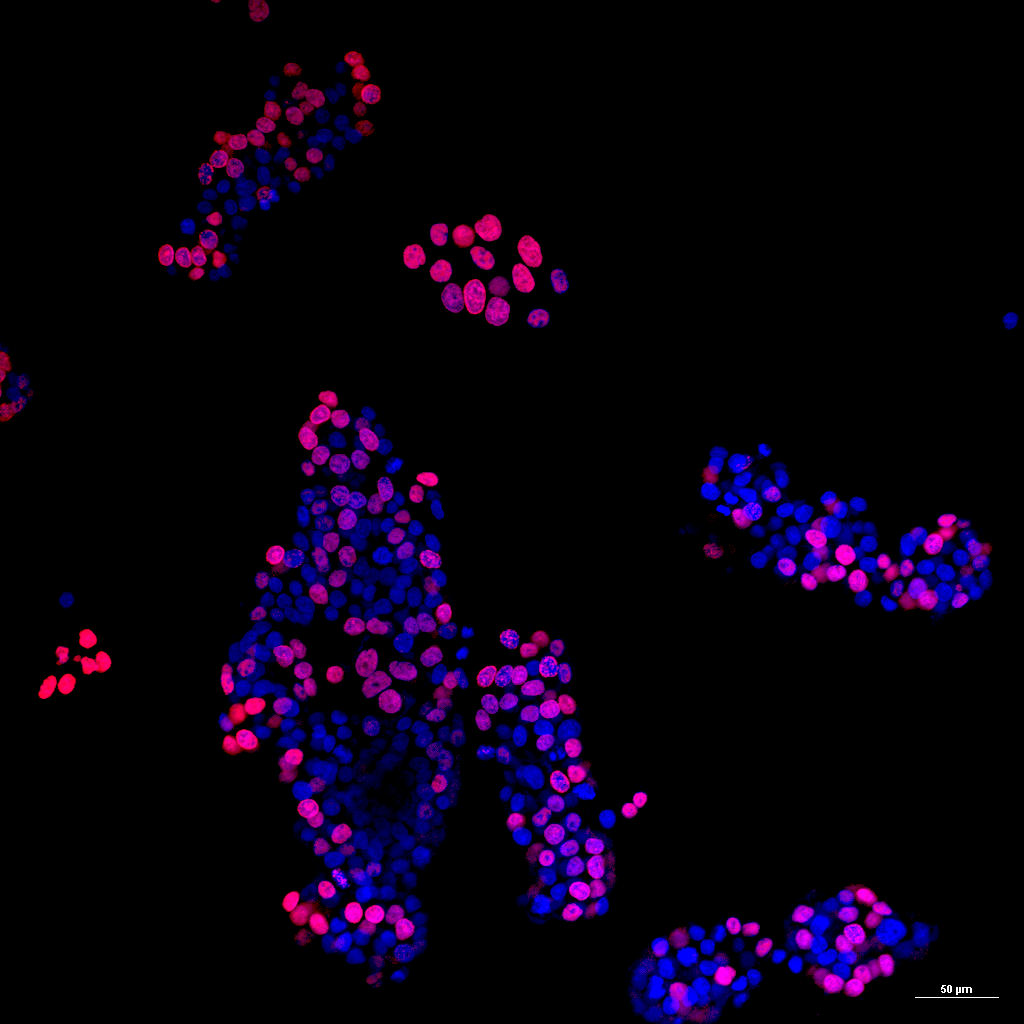

Supplement: Supplementary file 6 [file DataSheet6.zip › EDU/SW480-EDU/Vector/4_RGB.tif]

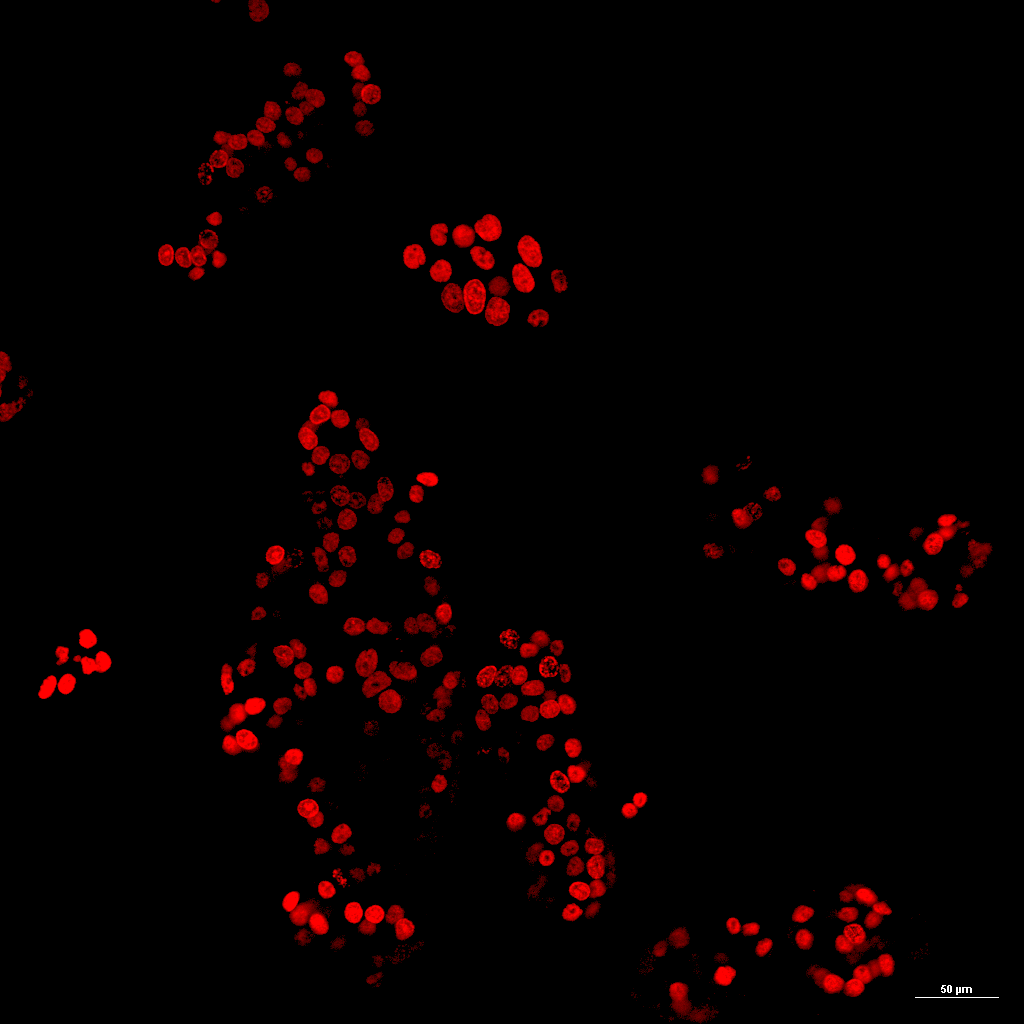

Supplement: Supplementary file 6 [file DataSheet6.zip › EDU/SW480-EDU/Vector/4_RGB_Alexa Fluor 594 cadaverine_H2O.tif]

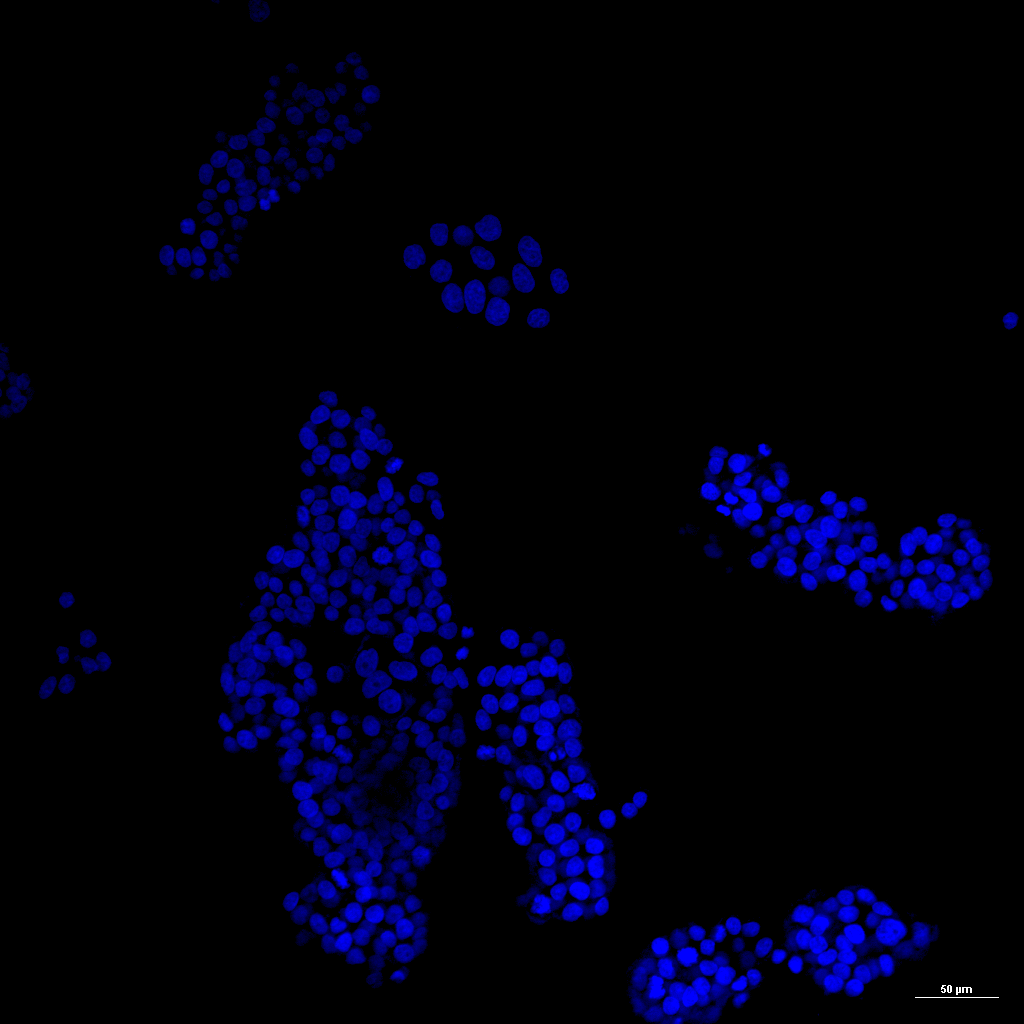

Supplement: Supplementary file 6 [file DataSheet6.zip › EDU/SW480-EDU/Vector/4_RGB_DAPI.tif]

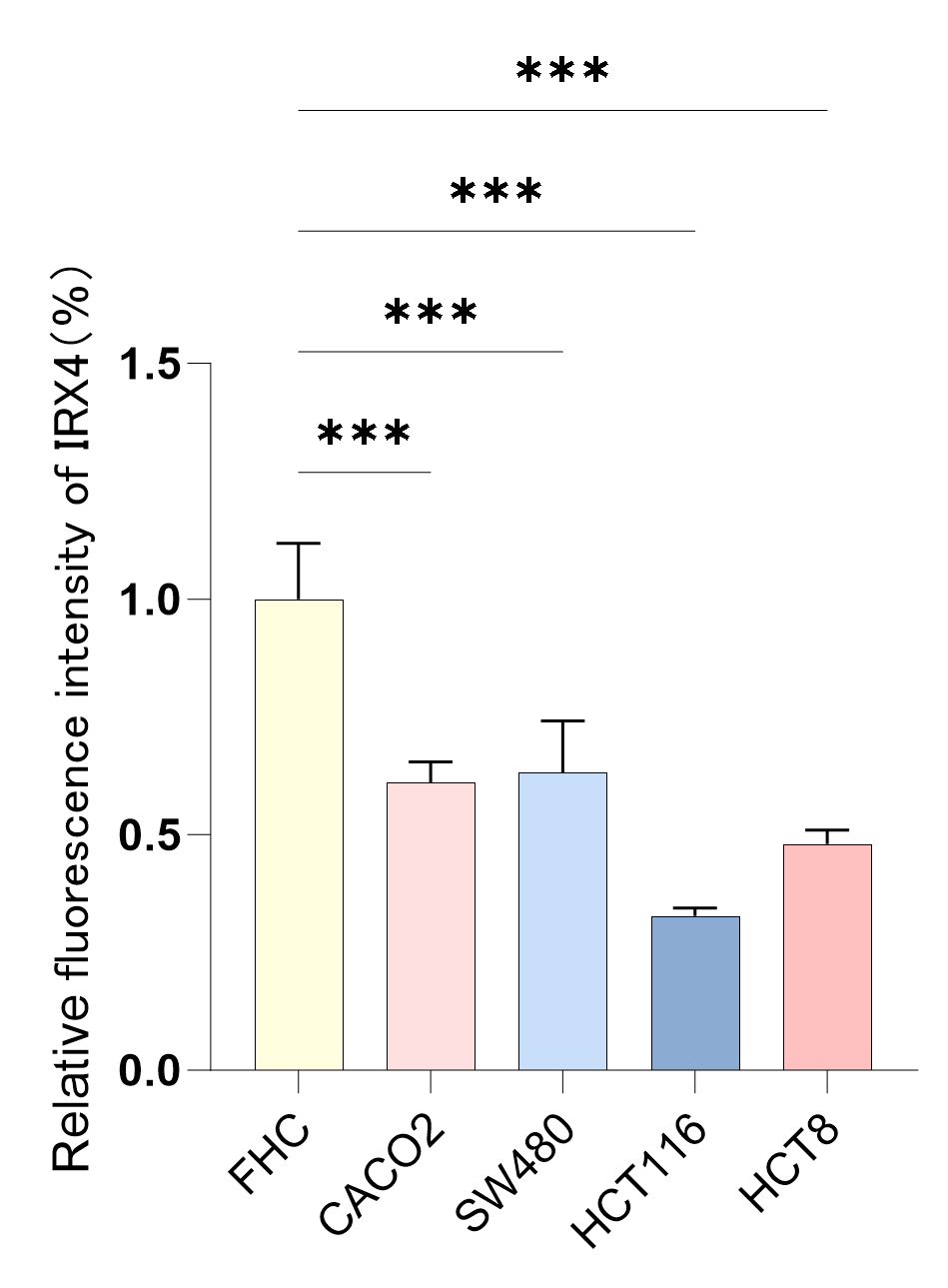

Supplement: Supplementary file 8 [file DataSheet8.zip › IF/Data 1.tif]

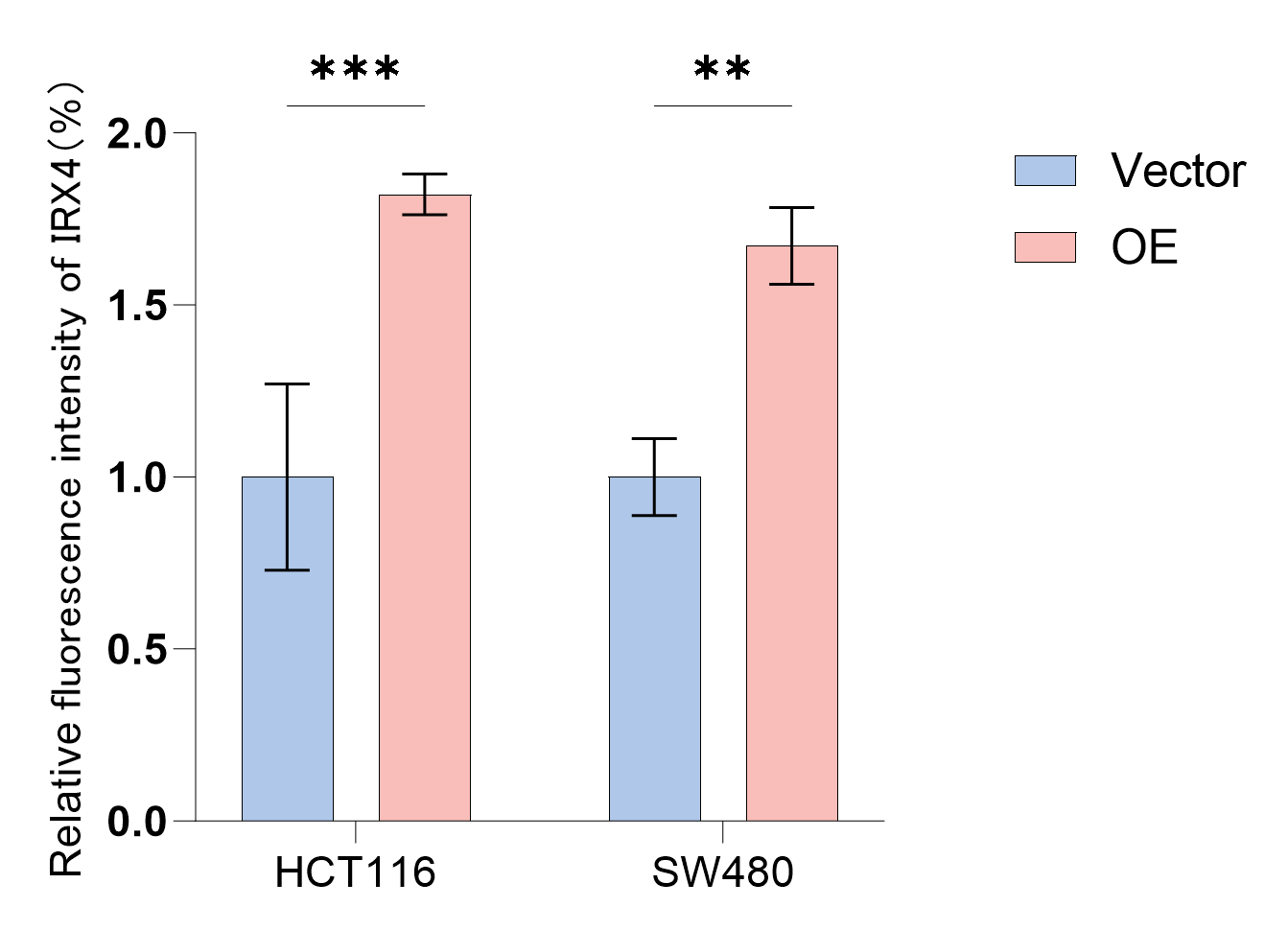

Supplement: Supplementary file 8 [file DataSheet8.zip › IF/Data 2.tif]

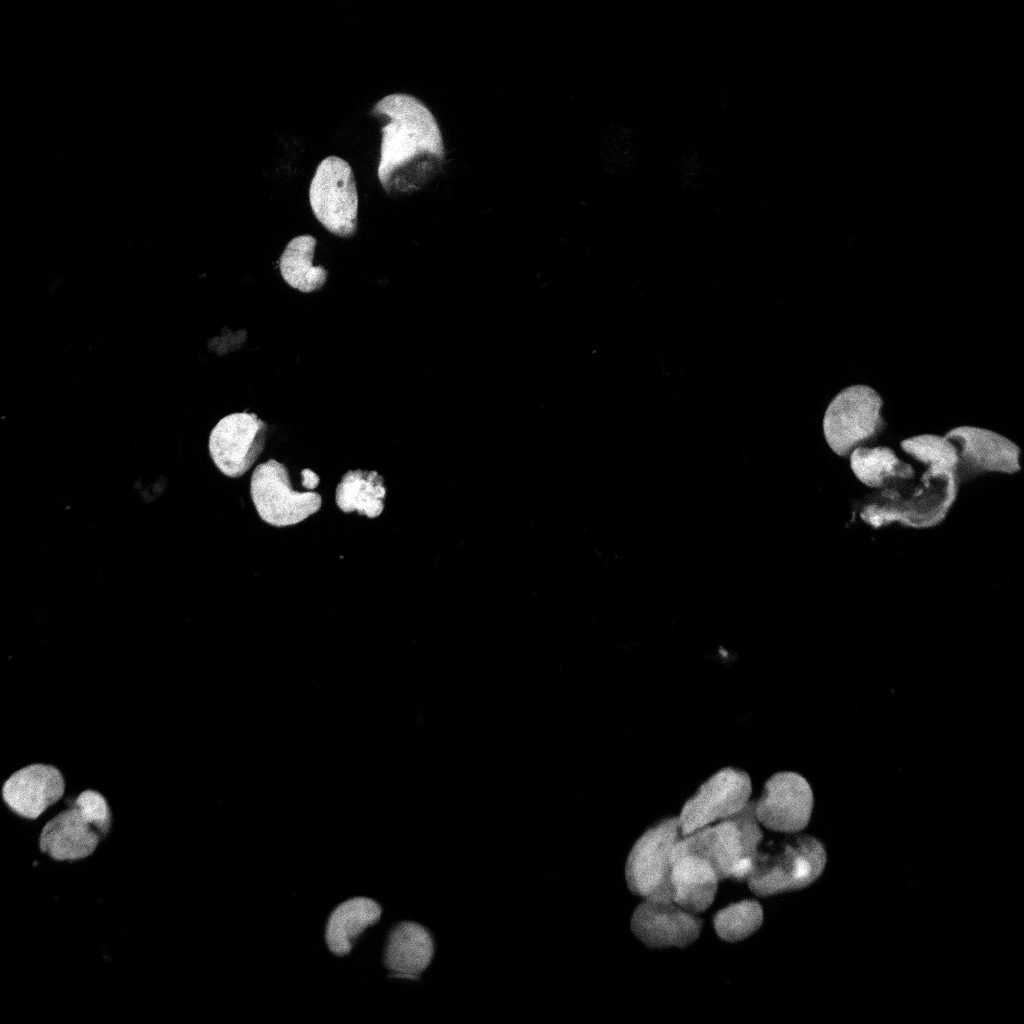

Supplement: Supplementary file 8 [file DataSheet8.zip › IF/IF-Cell expression/CACO2/3.tif]

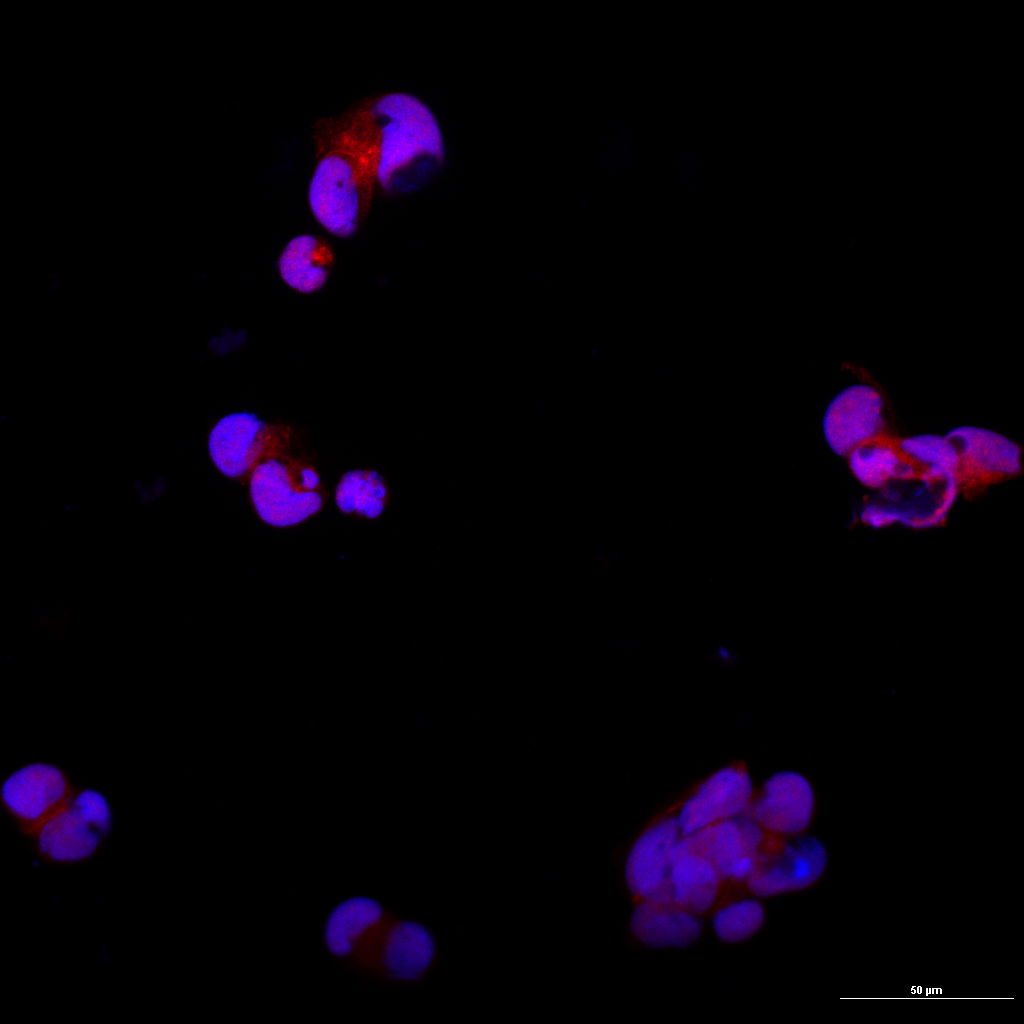

Supplement: Supplementary file 8 [file DataSheet8.zip › IF/IF-Cell expression/CACO2/3_RGB.tif]

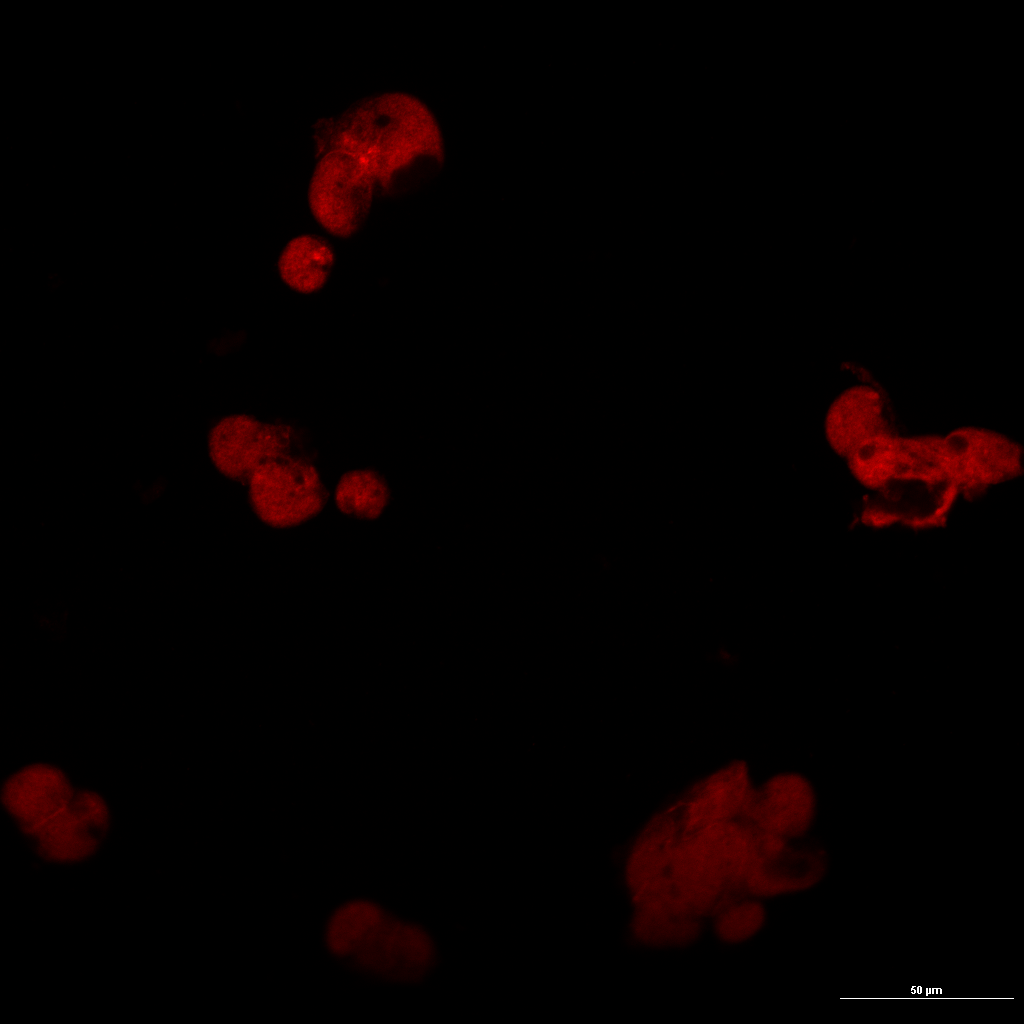

Supplement: Supplementary file 8 [file DataSheet8.zip › IF/IF-Cell expression/CACO2/3_RGB_Alexa Fluor 594 cadaverine_H2O.tif]

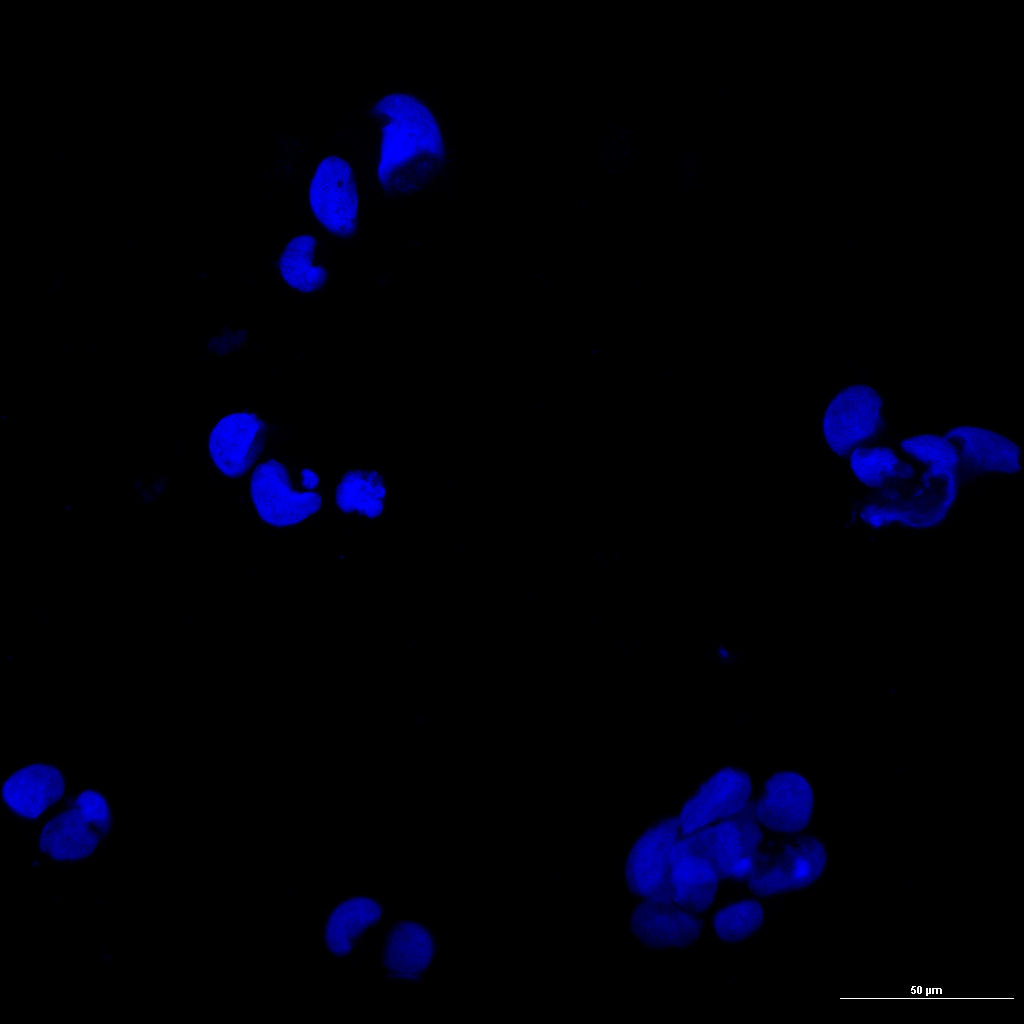

Supplement: Supplementary file 8 [file DataSheet8.zip › IF/IF-Cell expression/CACO2/3_RGB_DAPI.tif]

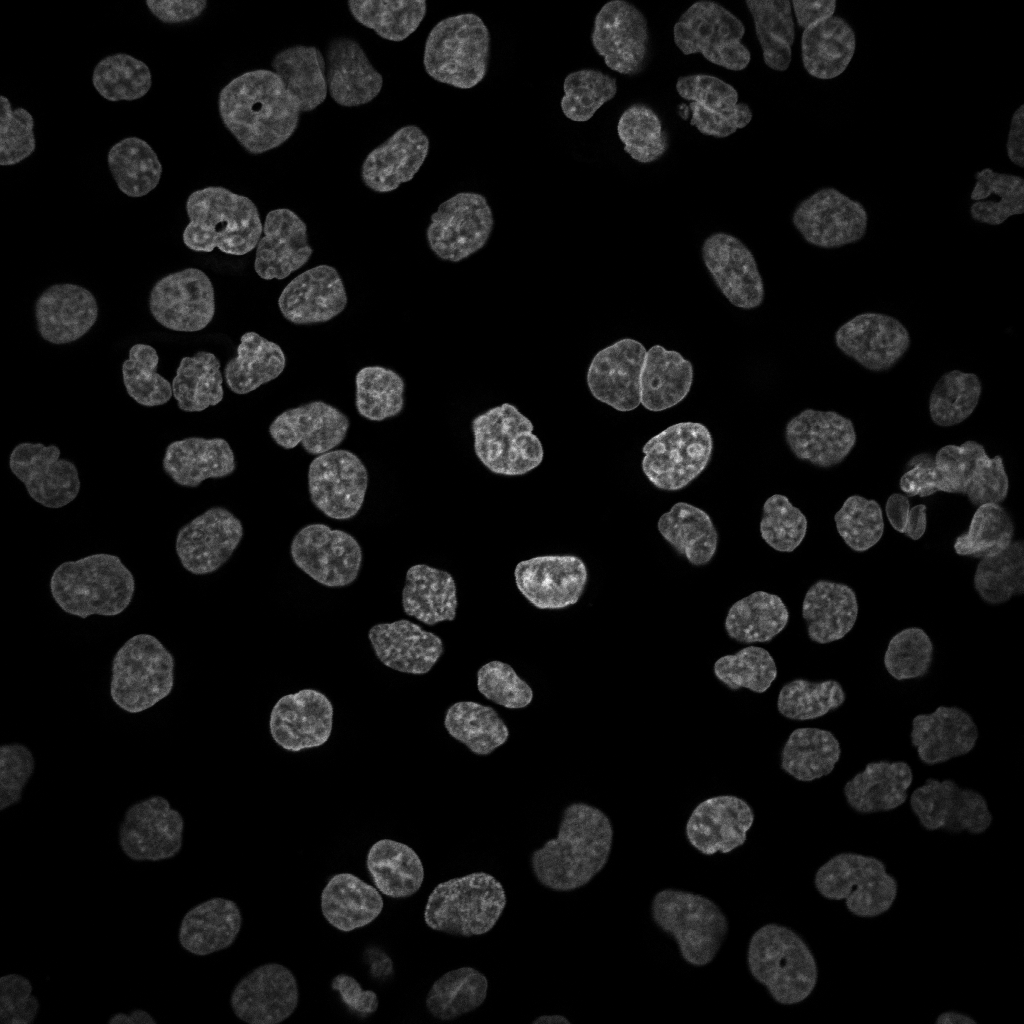

Supplement: Supplementary file 8 [file DataSheet8.zip › IF/IF-Cell expression/FHC/3.tif]

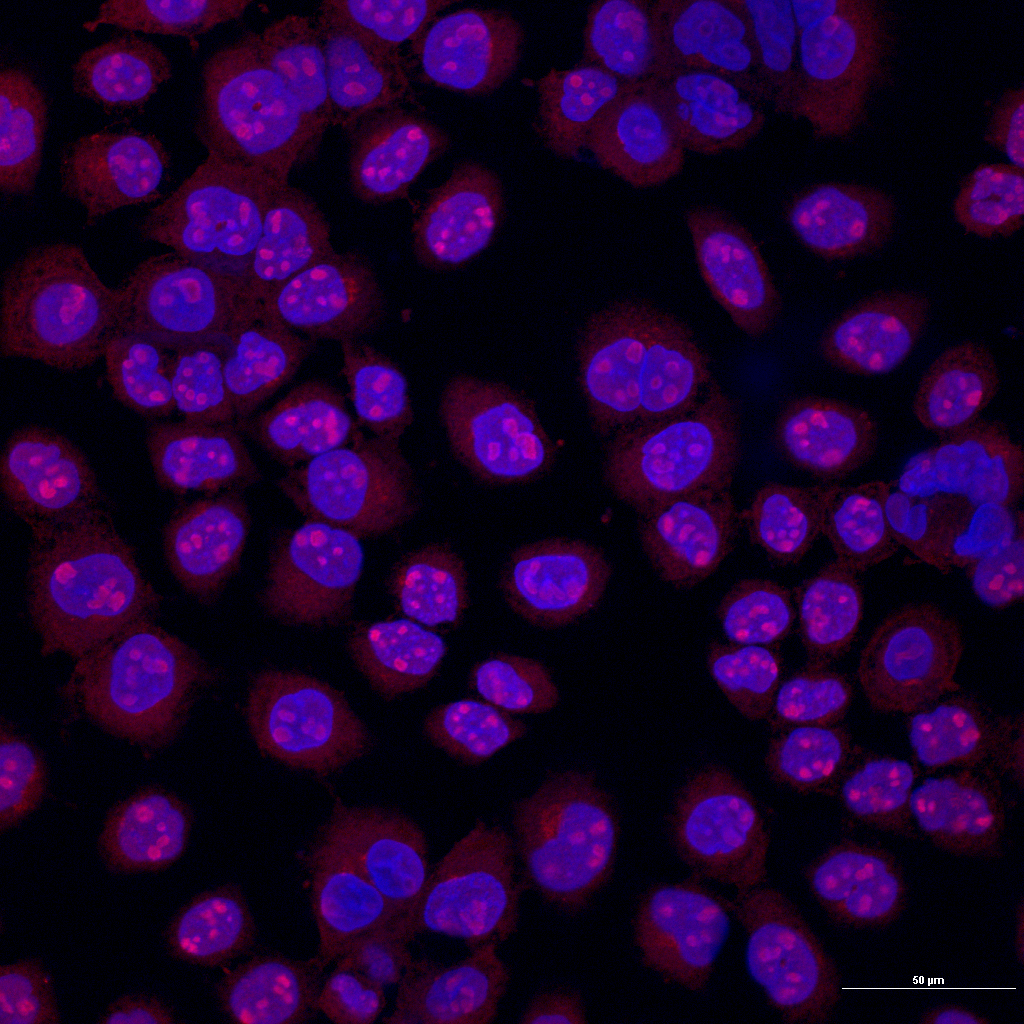

Supplement: Supplementary file 8 [file DataSheet8.zip › IF/IF-Cell expression/FHC/3_RGB.tif]

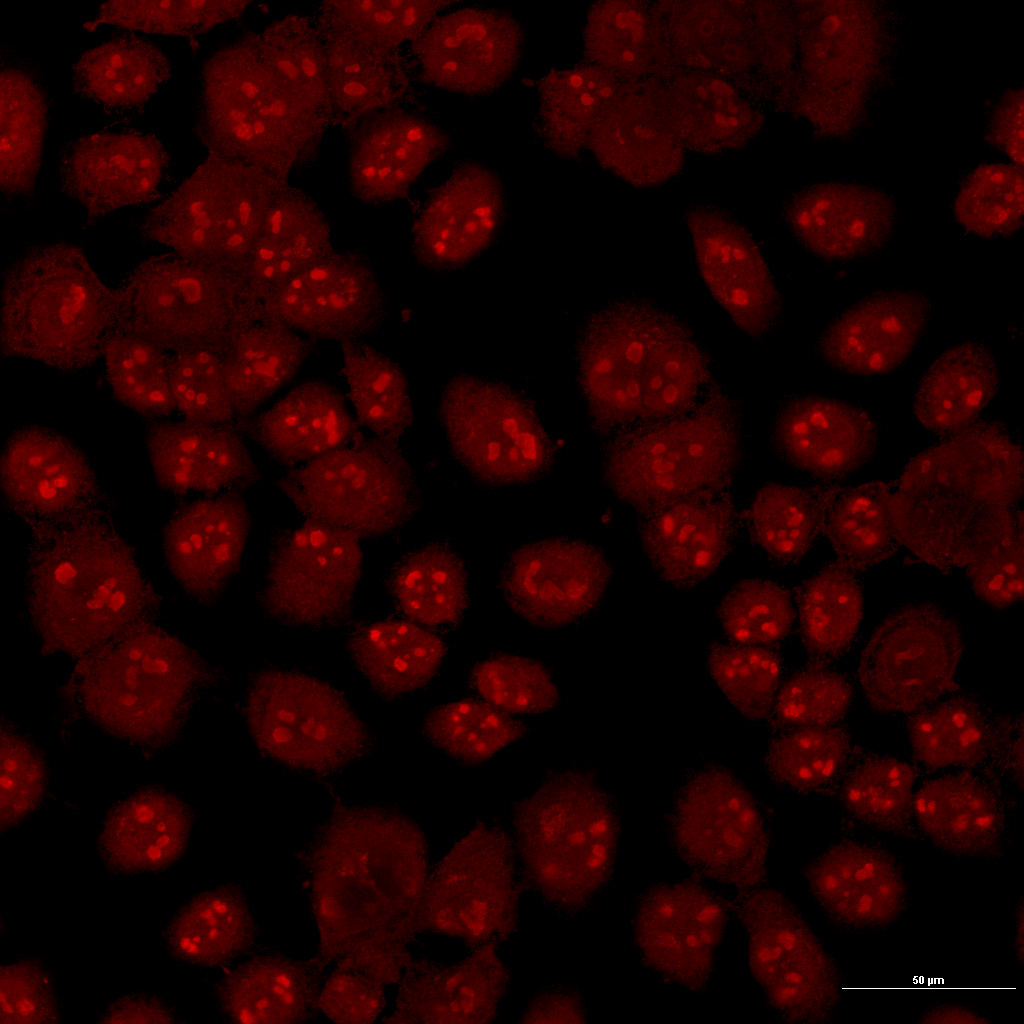

Supplement: Supplementary file 8 [file DataSheet8.zip › IF/IF-Cell expression/FHC/3_RGB_Alexa Fluor 594 cadaverine_H2O.tif]
