# Supplementary figures and images for: Anti-colorectal cancer effects of IRX4 and sensitivity studies to oxaliplatin (part 2 of 2)
Source: Front Immunol. 2026 Jan 21;16:1581244. doi: 10.3389/fimmu.2025.1581244 (PMC12867854; doi:10.3389/fimmu.2025.1581244)

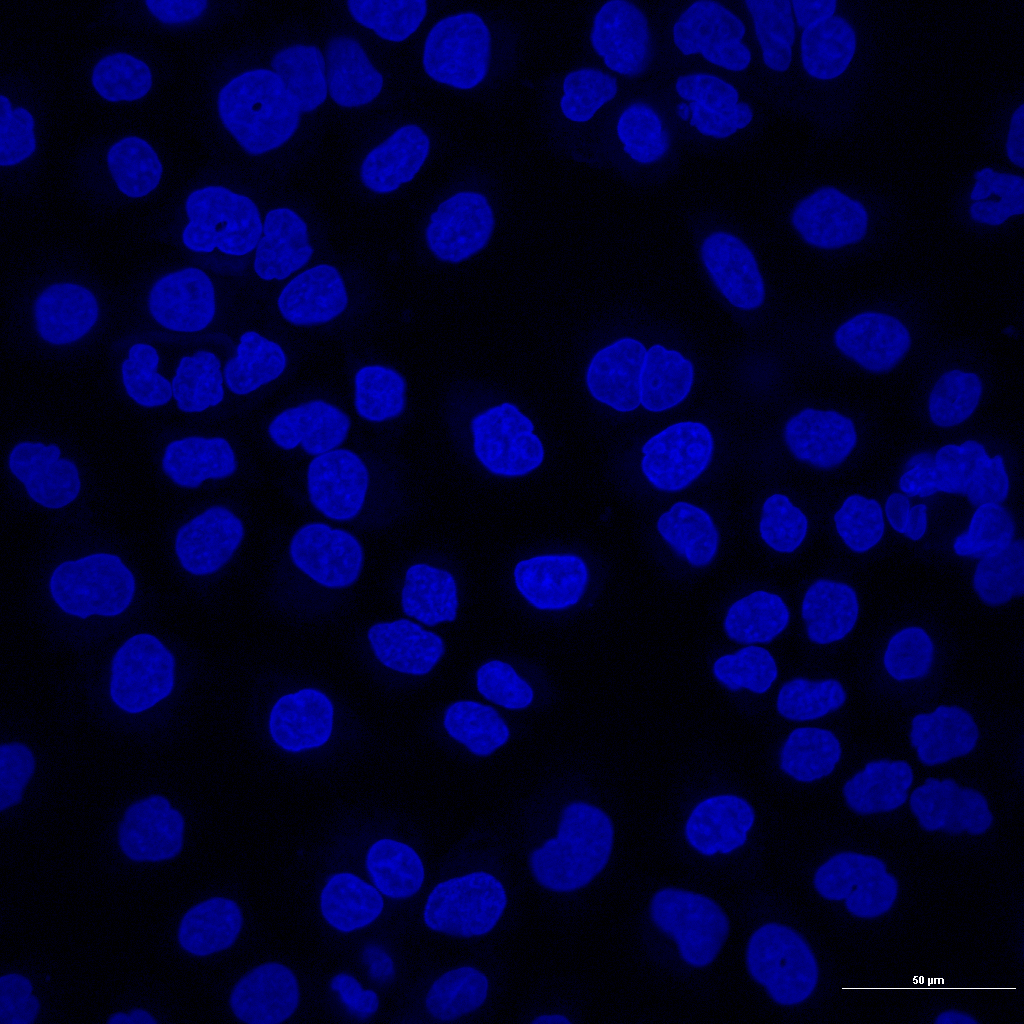

Supplement: Supplementary file 8 [file DataSheet8.zip › IF/IF-Cell expression/FHC/3_RGB_DAPI.tif]

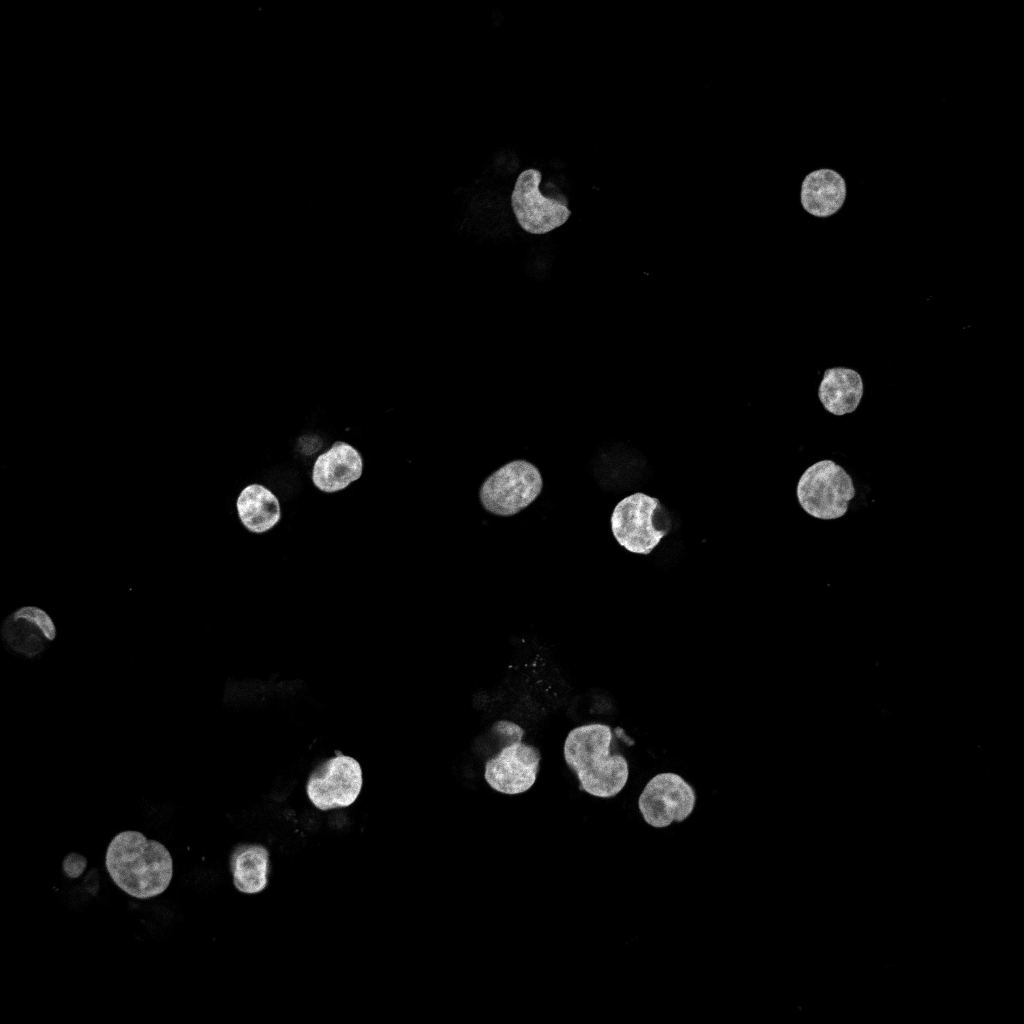

Supplement: Supplementary file 8 [file DataSheet8.zip › IF/IF-Cell expression/HCT116/12.tif]

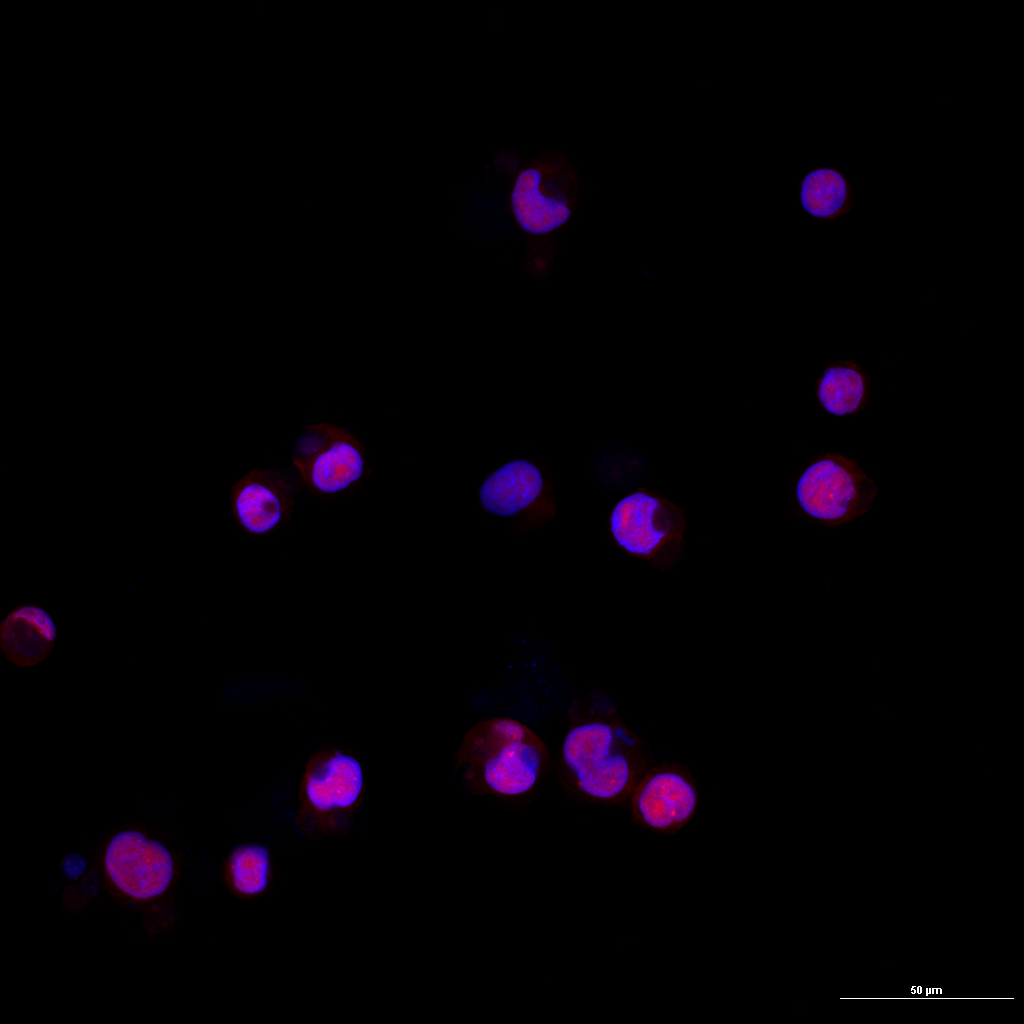

Supplement: Supplementary file 8 [file DataSheet8.zip › IF/IF-Cell expression/HCT116/12_RGB.tif]

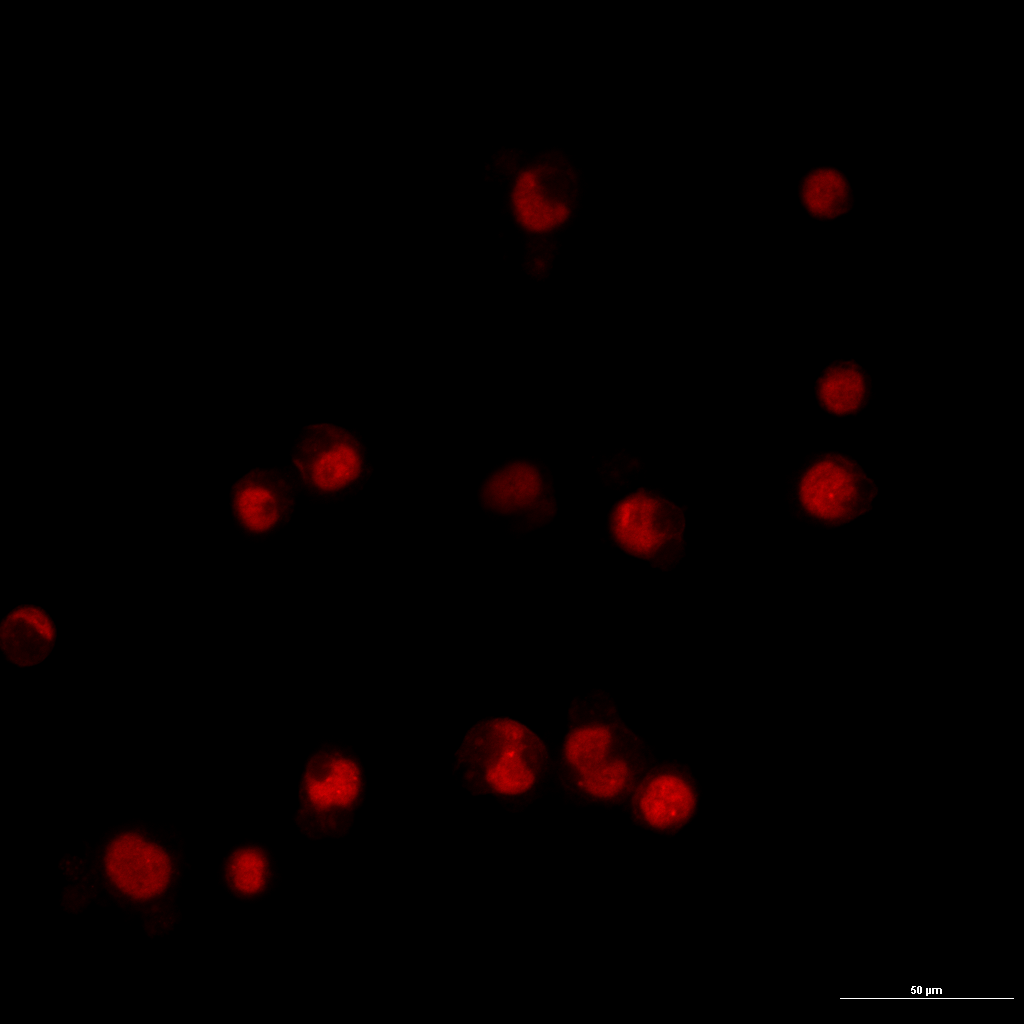

Supplement: Supplementary file 8 [file DataSheet8.zip › IF/IF-Cell expression/HCT116/12_RGB_Alexa Fluor 594 cadaverine_H2O.tif]

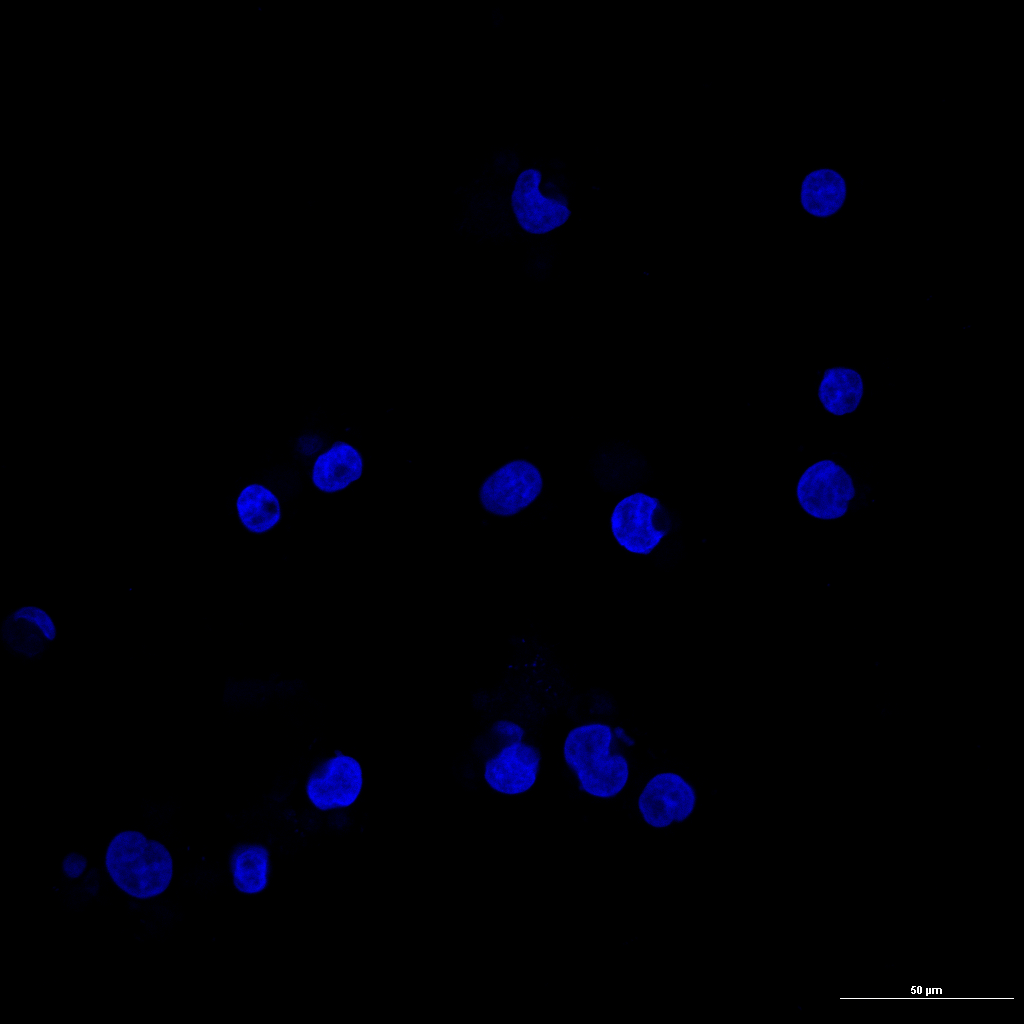

Supplement: Supplementary file 8 [file DataSheet8.zip › IF/IF-Cell expression/HCT116/12_RGB_DAPI.tif]

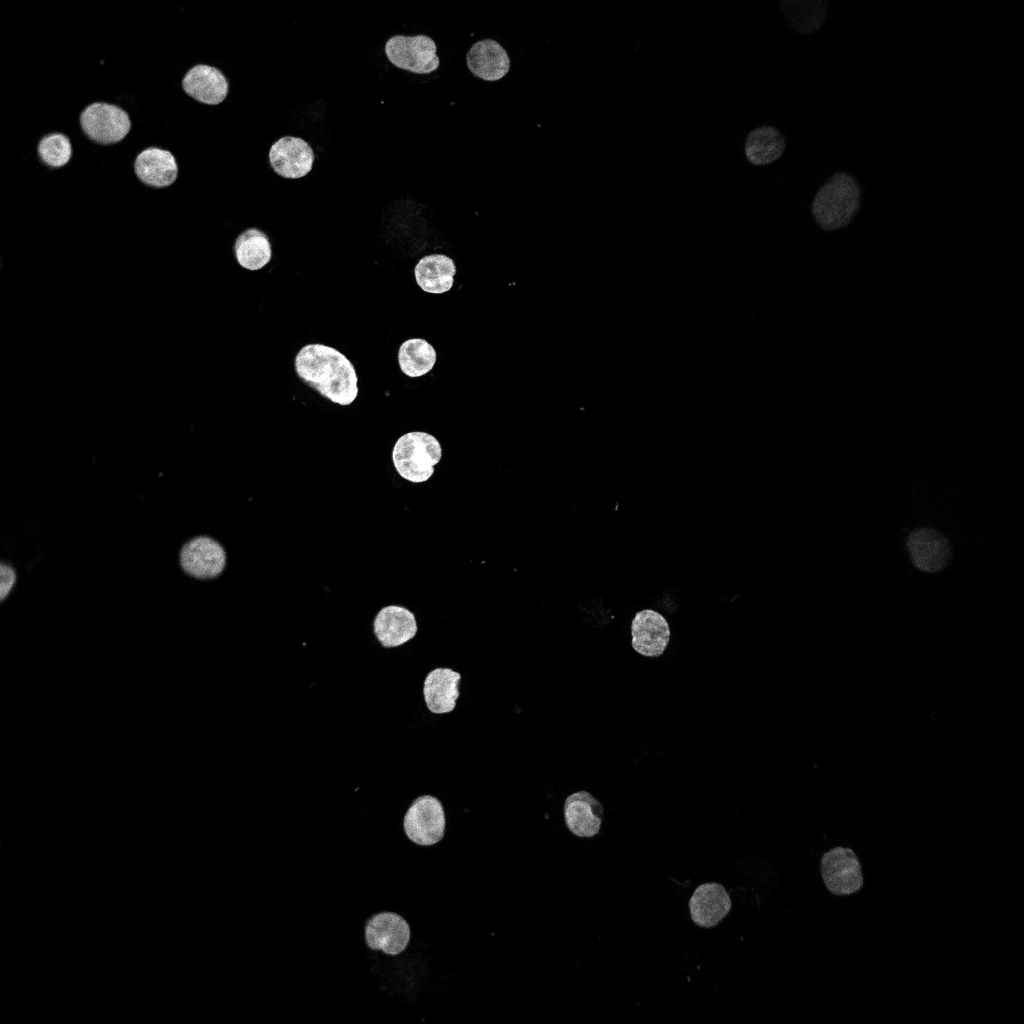

Supplement: Supplementary file 8 [file DataSheet8.zip › IF/IF-Cell expression/HCT8/1.tif]

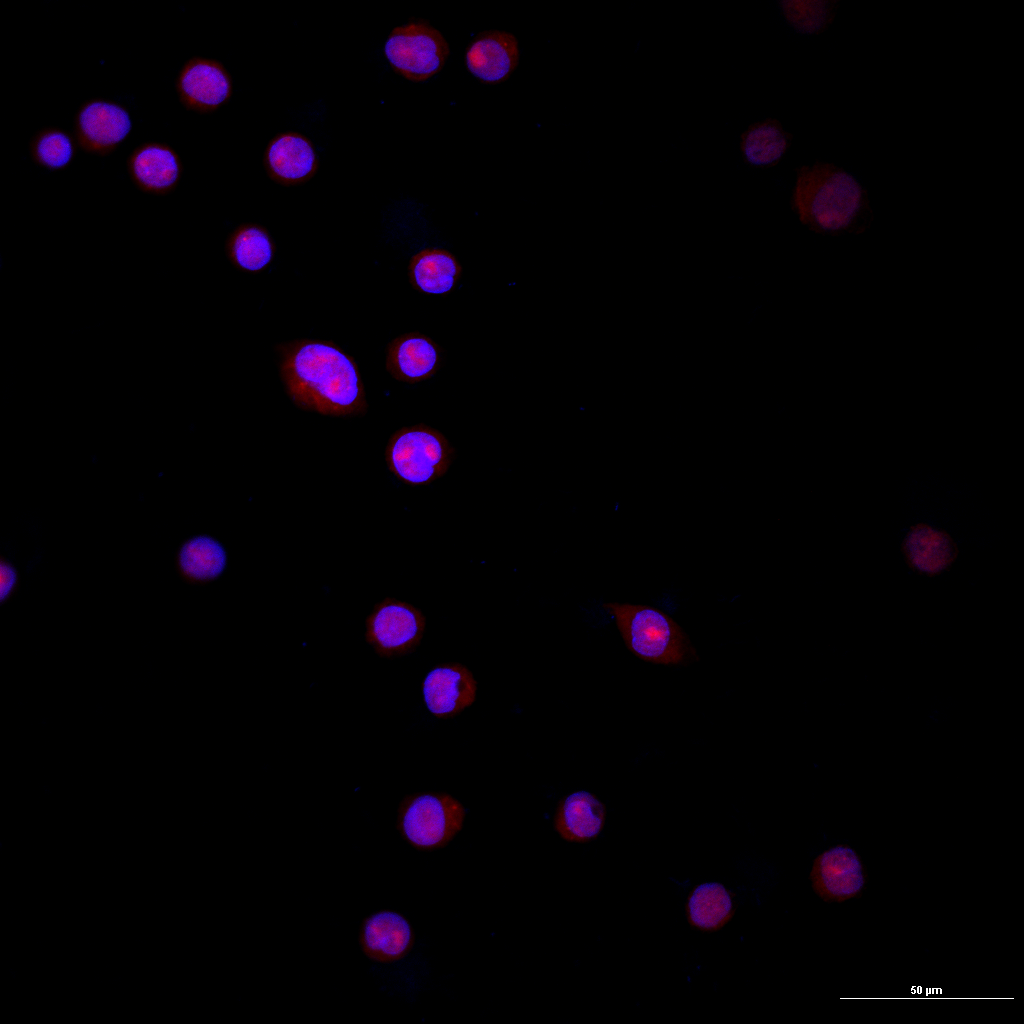

Supplement: Supplementary file 8 [file DataSheet8.zip › IF/IF-Cell expression/HCT8/1_RGB.tif]

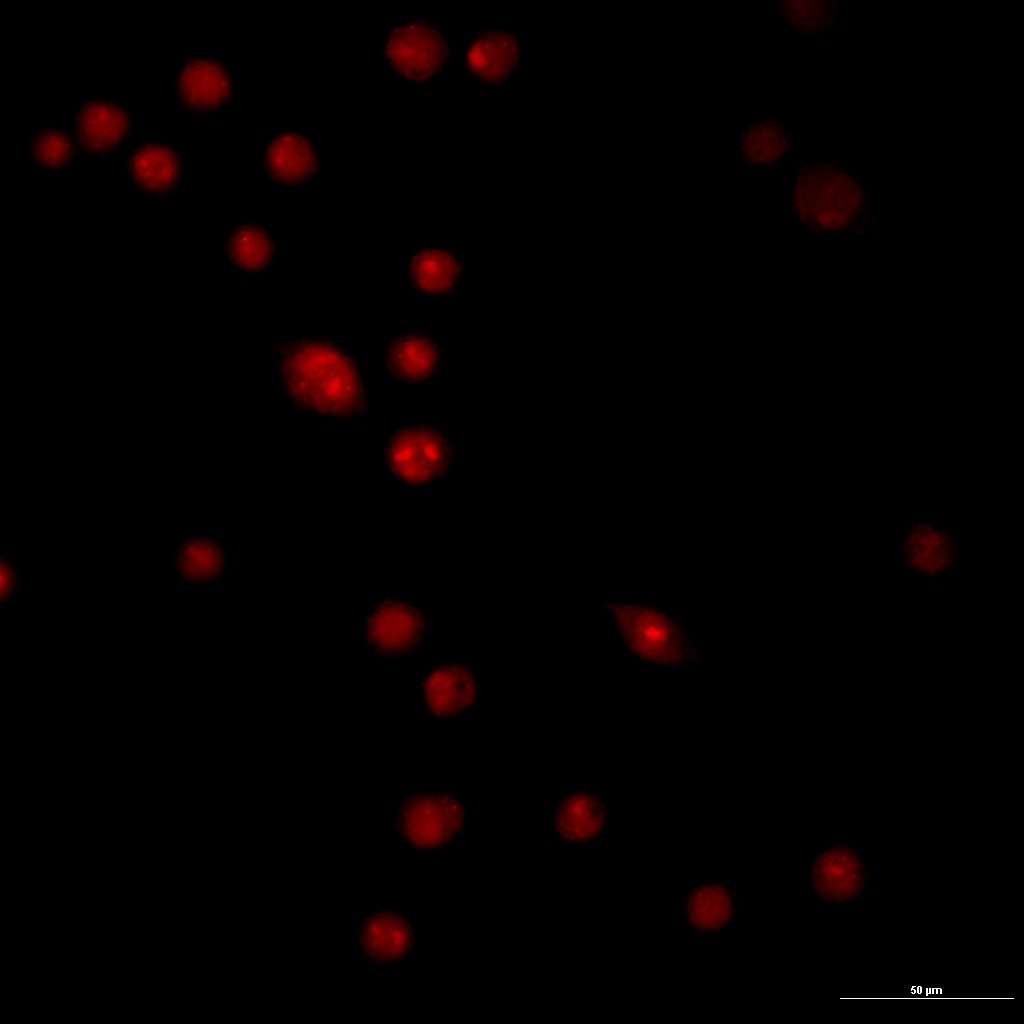

Supplement: Supplementary file 8 [file DataSheet8.zip › IF/IF-Cell expression/HCT8/1_RGB_Alexa Fluor 594 cadaverine_H2O.tif]

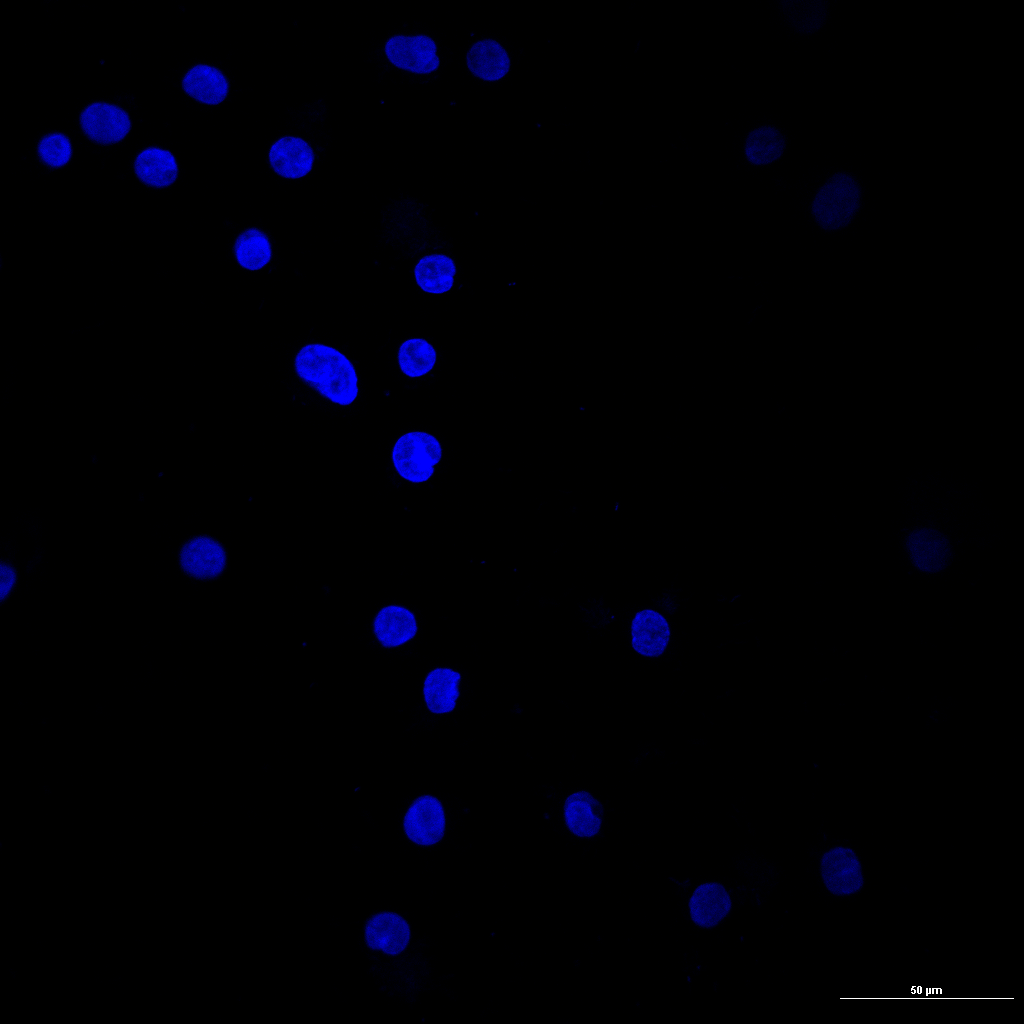

Supplement: Supplementary file 8 [file DataSheet8.zip › IF/IF-Cell expression/HCT8/1_RGB_DAPI.tif]

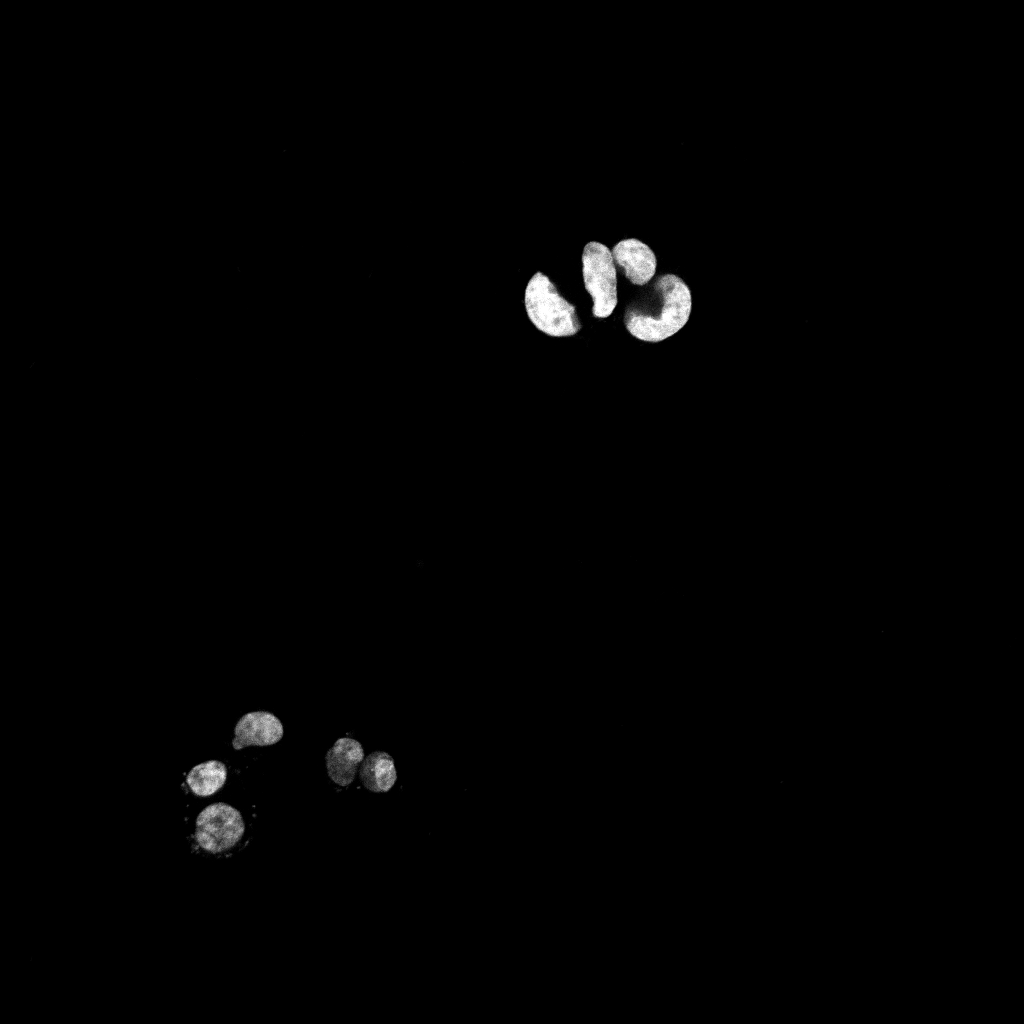

Supplement: Supplementary file 8 [file DataSheet8.zip › IF/IF-Cell expression/SW480/3.tif]

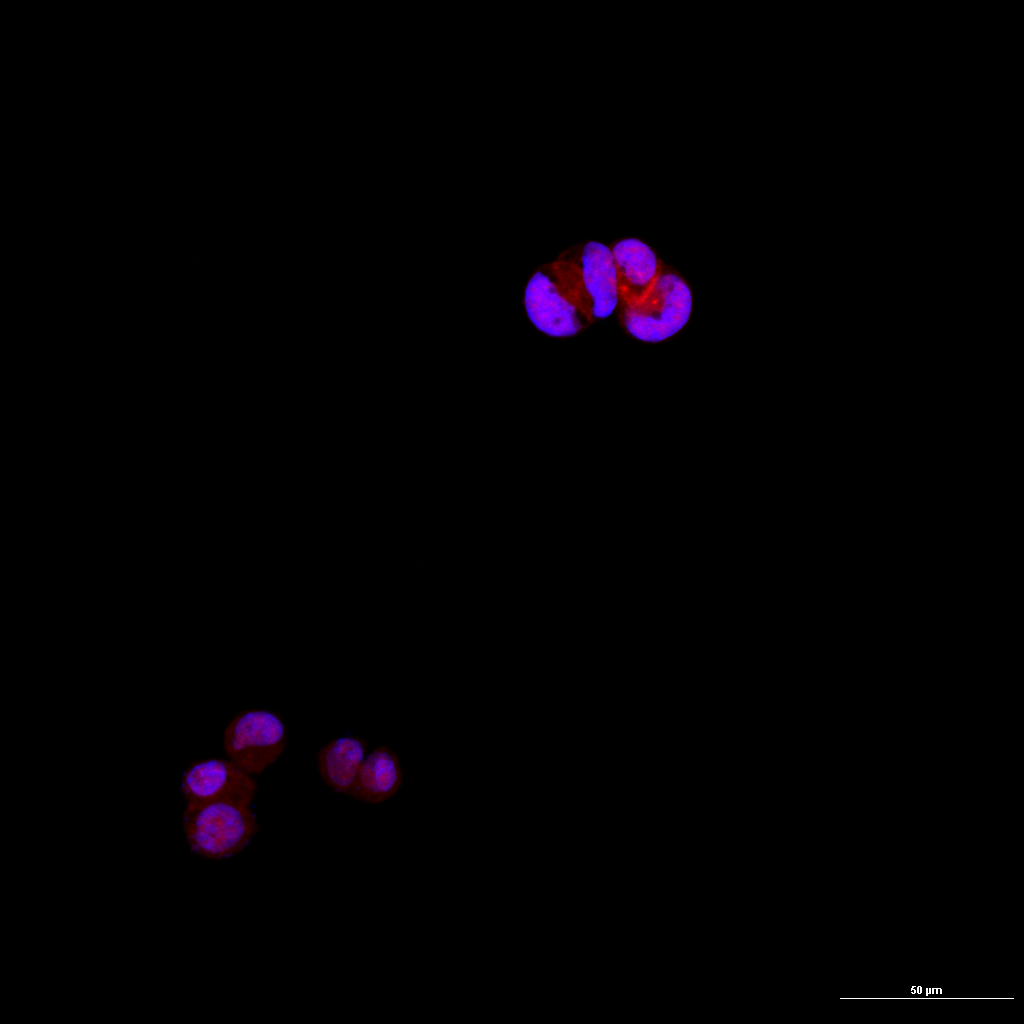

Supplement: Supplementary file 8 [file DataSheet8.zip › IF/IF-Cell expression/SW480/3_RGB.tif]

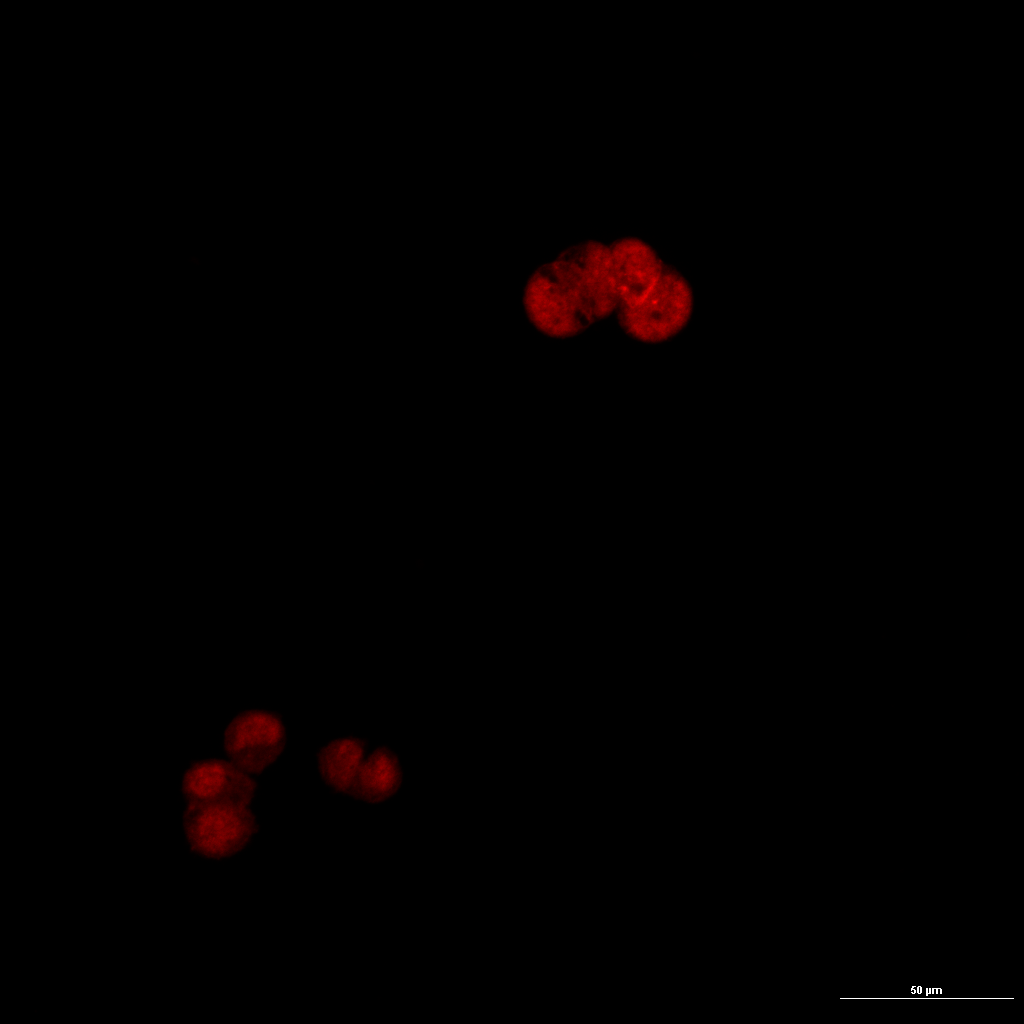

Supplement: Supplementary file 8 [file DataSheet8.zip › IF/IF-Cell expression/SW480/3_RGB_Alexa Fluor 594 cadaverine_H2O.tif]

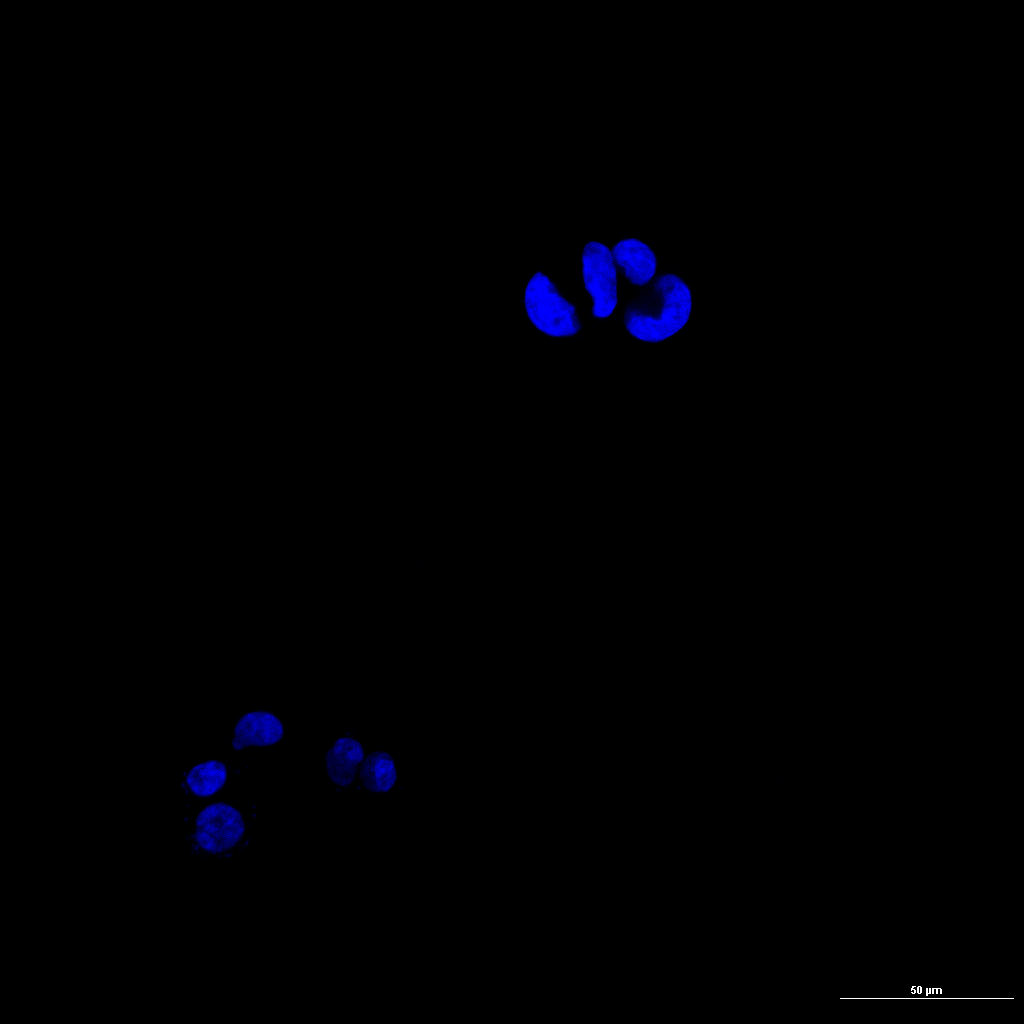

Supplement: Supplementary file 8 [file DataSheet8.zip › IF/IF-Cell expression/SW480/3_RGB_DAPI.tif]

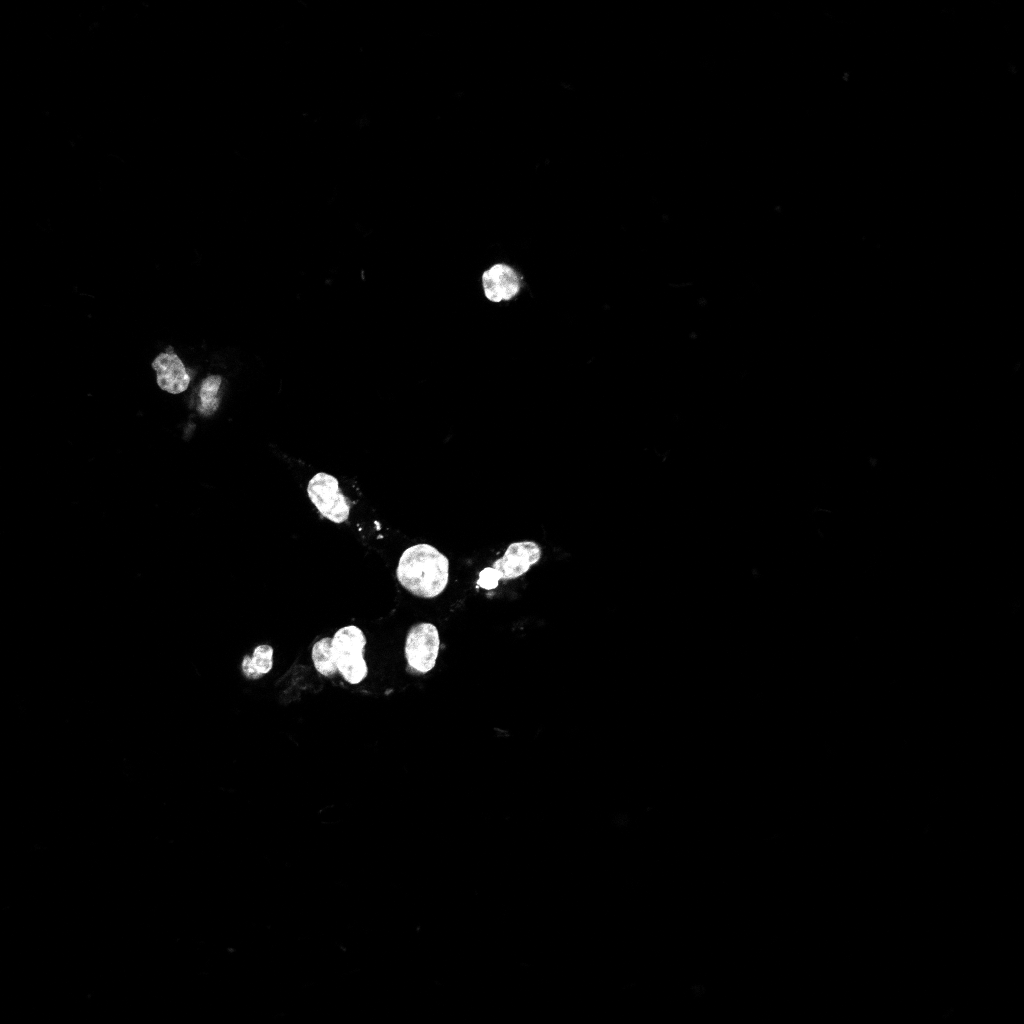

Supplement: Supplementary file 8 [file DataSheet8.zip › IF/IF-Overexpression verification/HCT116/OE/6.tif]

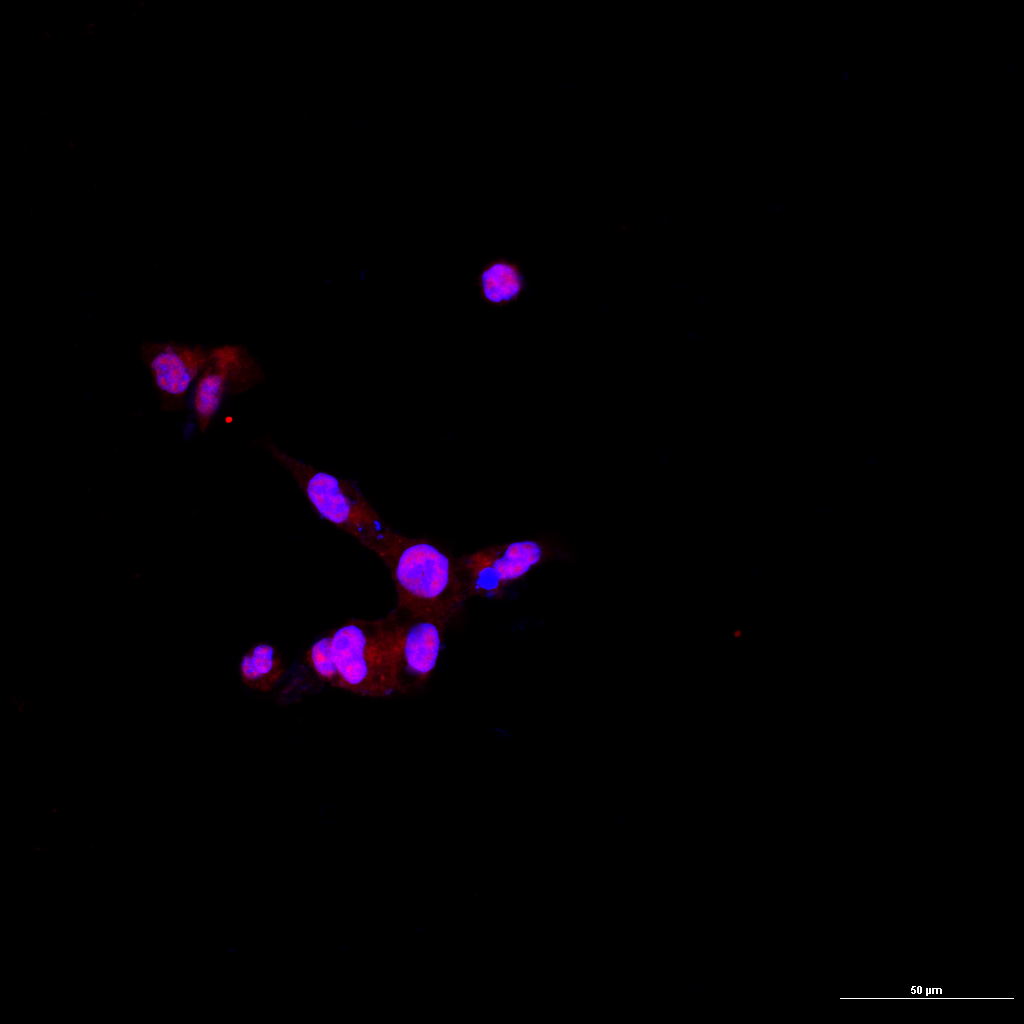

Supplement: Supplementary file 8 [file DataSheet8.zip › IF/IF-Overexpression verification/HCT116/OE/6_RGB.tif]

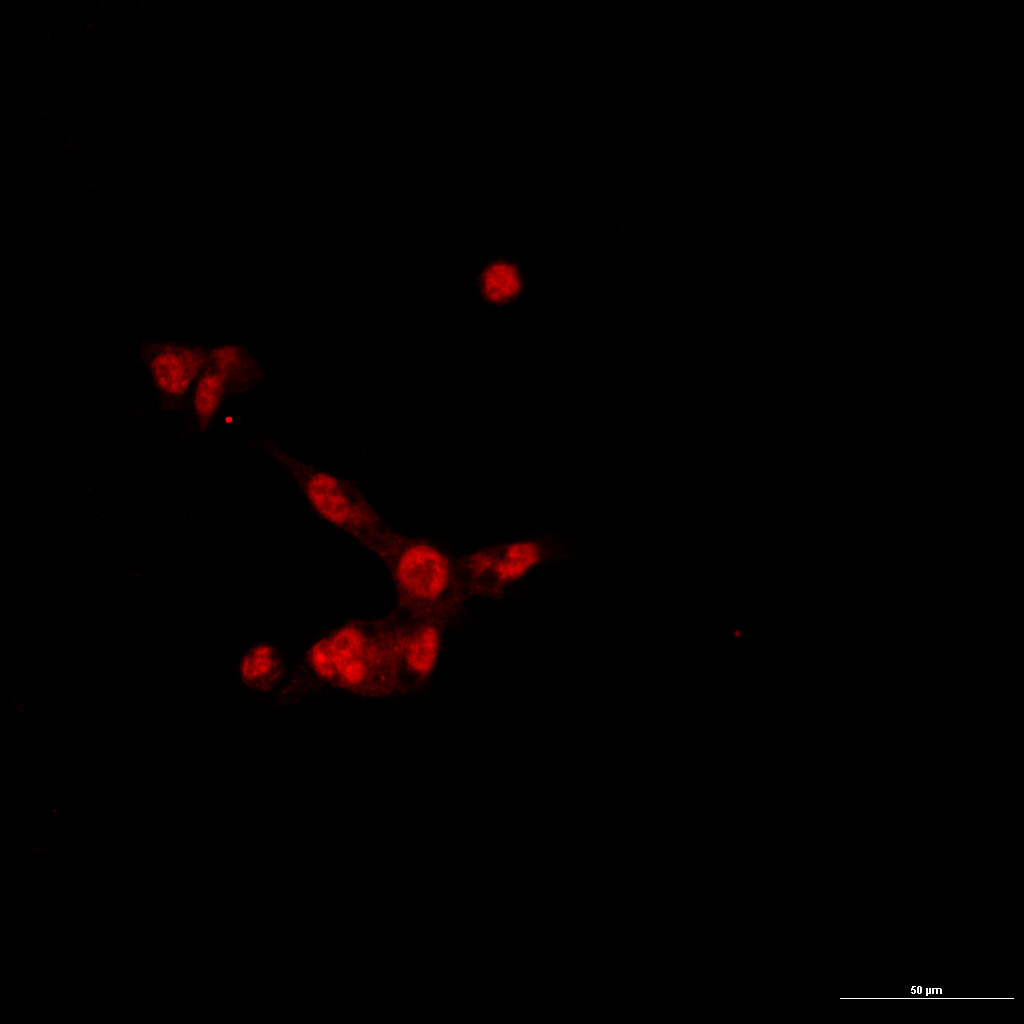

Supplement: Supplementary file 8 [file DataSheet8.zip › IF/IF-Overexpression verification/HCT116/OE/6_RGB_Alexa Fluor 594 cadaverine_H2O.tif]

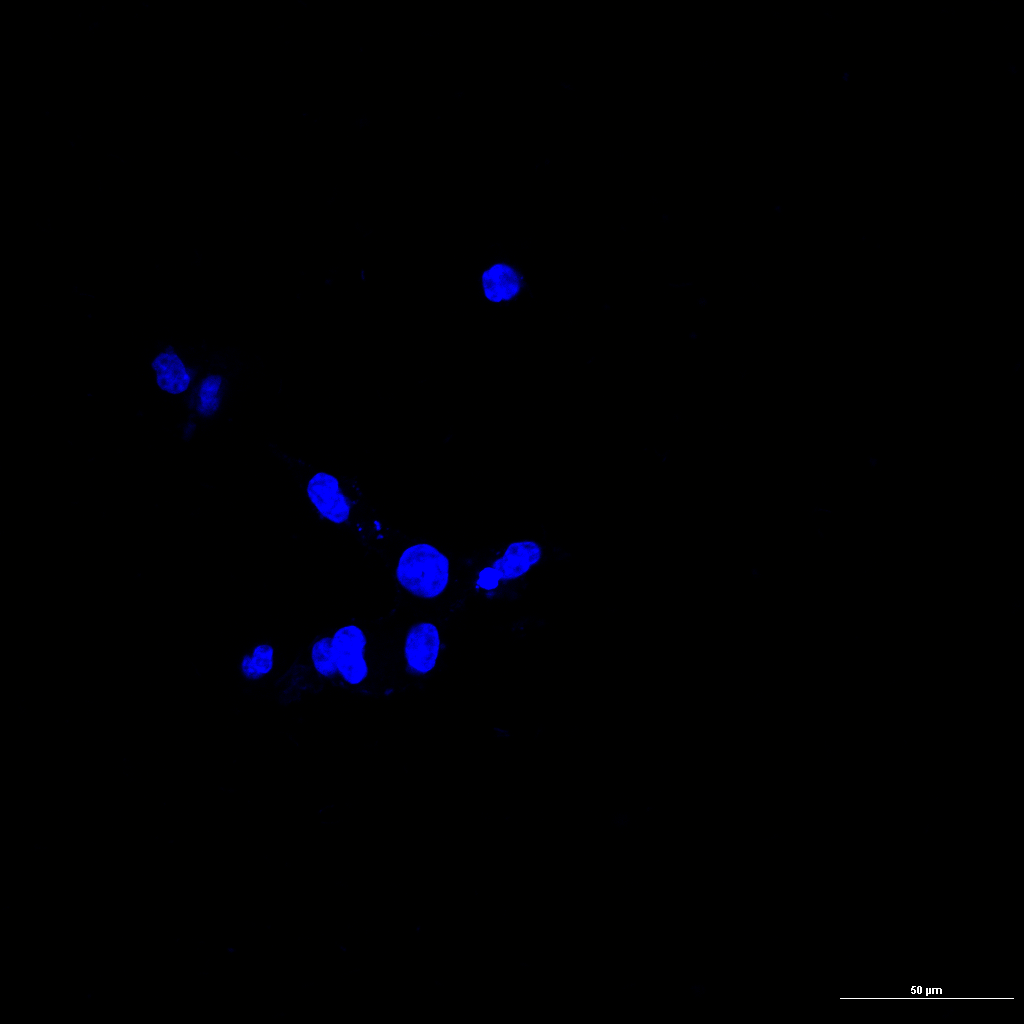

Supplement: Supplementary file 8 [file DataSheet8.zip › IF/IF-Overexpression verification/HCT116/OE/6_RGB_DAPI.tif]

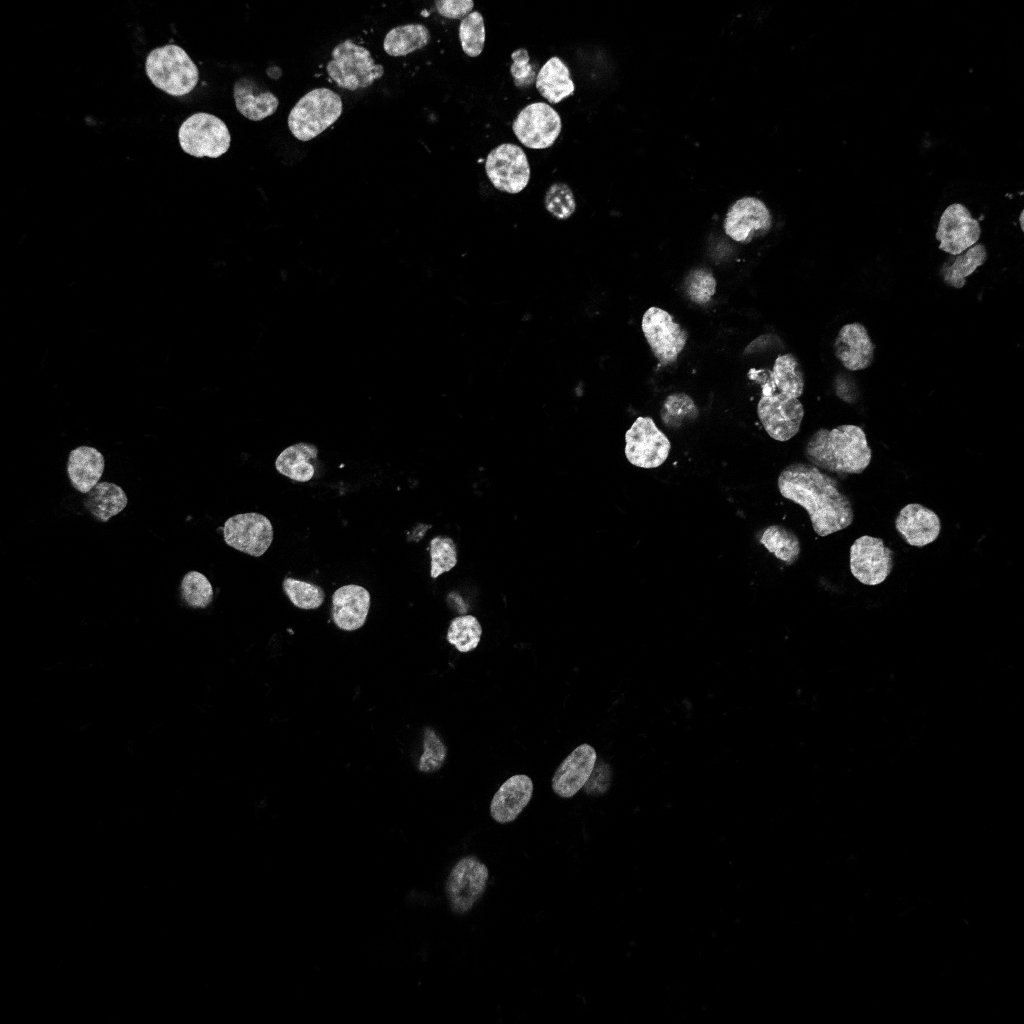

Supplement: Supplementary file 8 [file DataSheet8.zip › IF/IF-Overexpression verification/HCT116/Vector/7.tif]

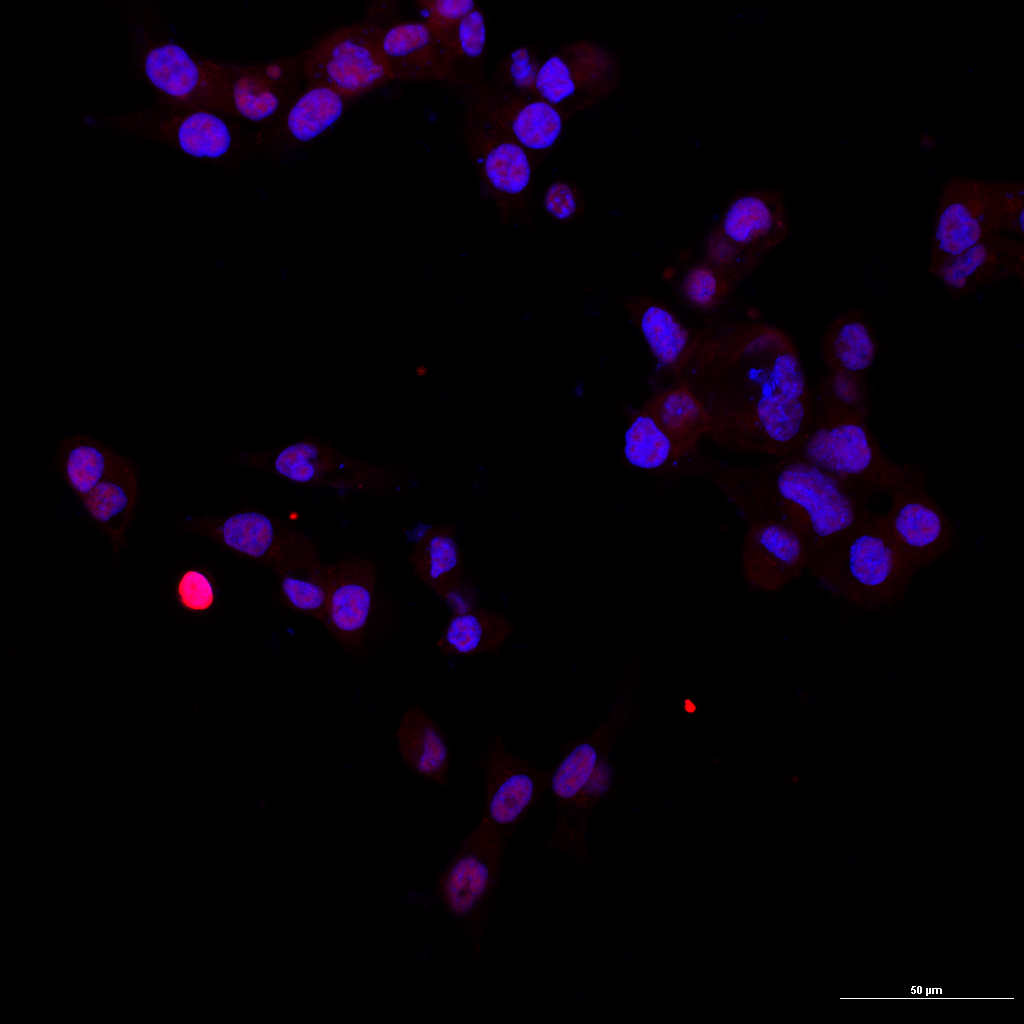

Supplement: Supplementary file 8 [file DataSheet8.zip › IF/IF-Overexpression verification/HCT116/Vector/7_RGB.tif]

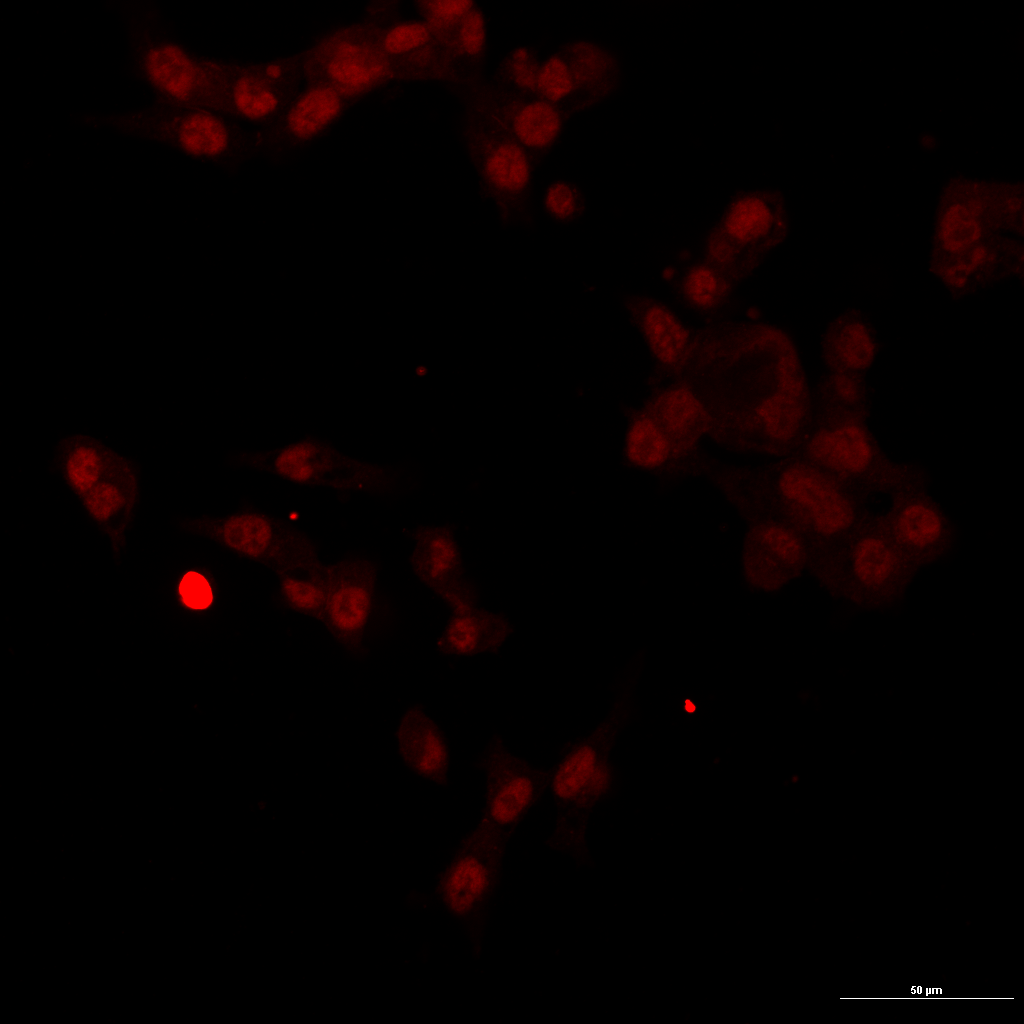

Supplement: Supplementary file 8 [file DataSheet8.zip › IF/IF-Overexpression verification/HCT116/Vector/7_RGB_Alexa Fluor 594 cadaverine_H2O.tif]

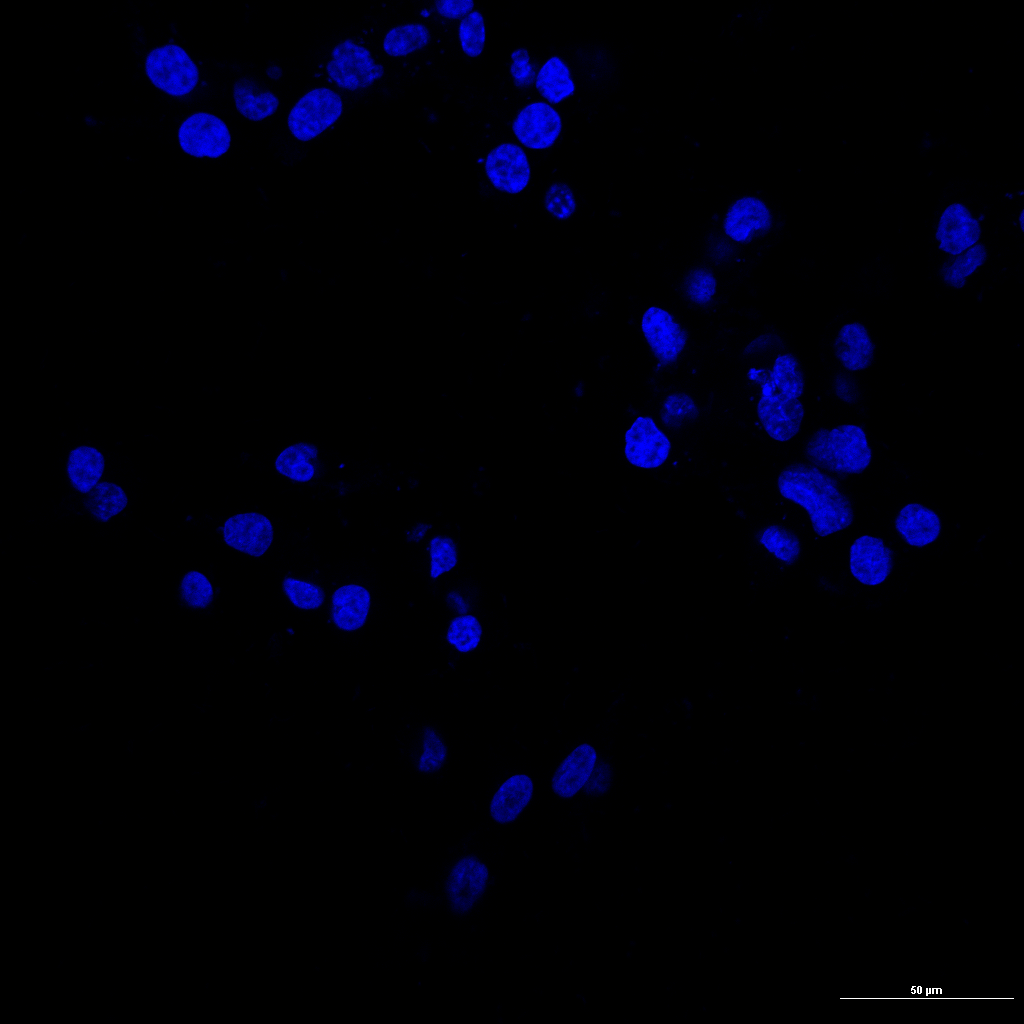

Supplement: Supplementary file 8 [file DataSheet8.zip › IF/IF-Overexpression verification/HCT116/Vector/7_RGB_DAPI.tif]

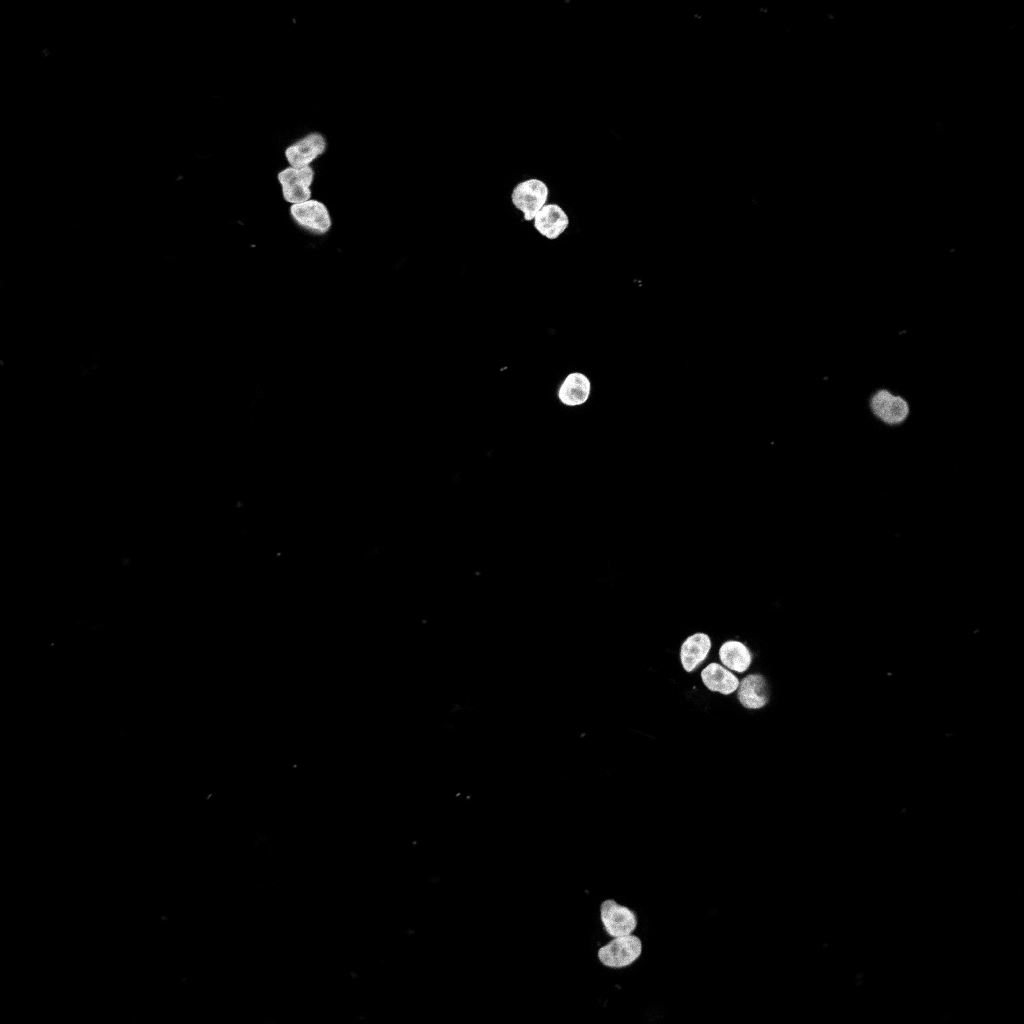

Supplement: Supplementary file 8 [file DataSheet8.zip › IF/IF-Overexpression verification/SW480/OE/4.tif]

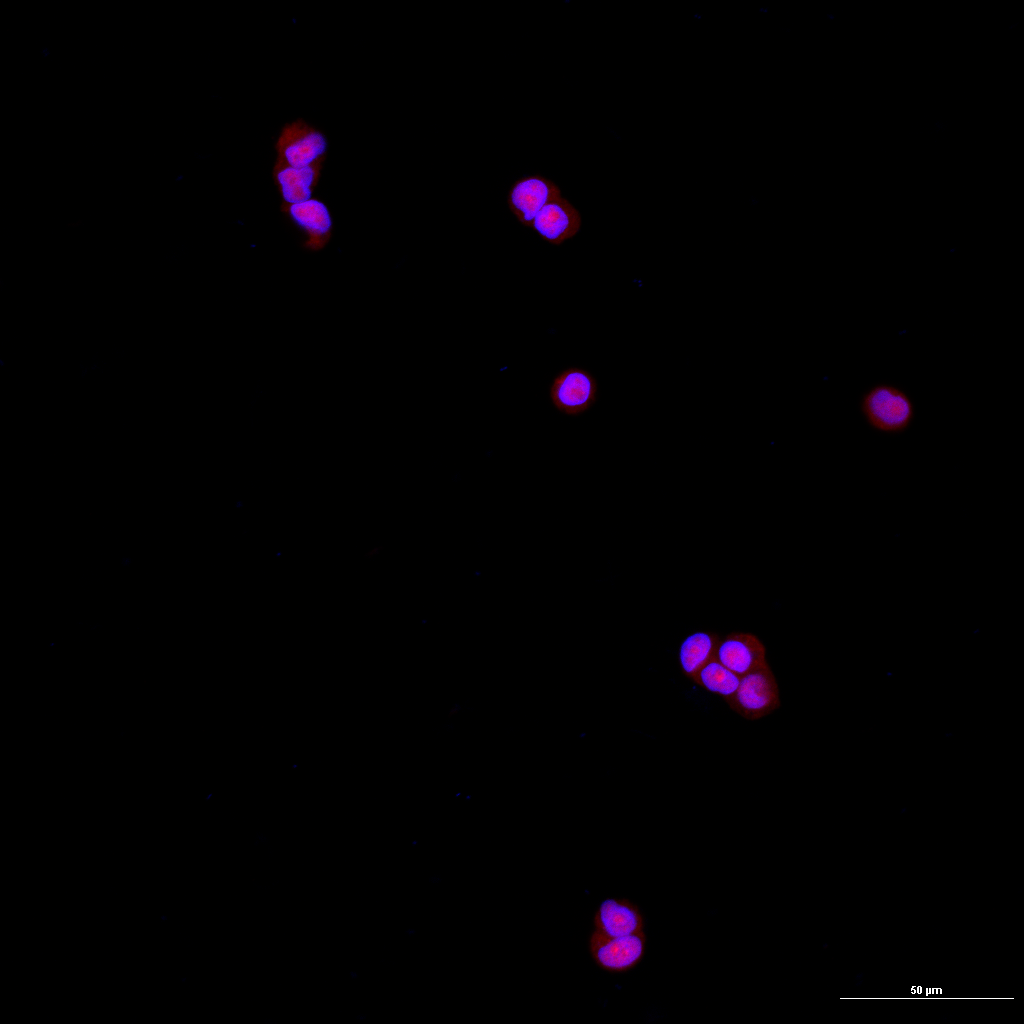

Supplement: Supplementary file 8 [file DataSheet8.zip › IF/IF-Overexpression verification/SW480/OE/4_RGB.tif]

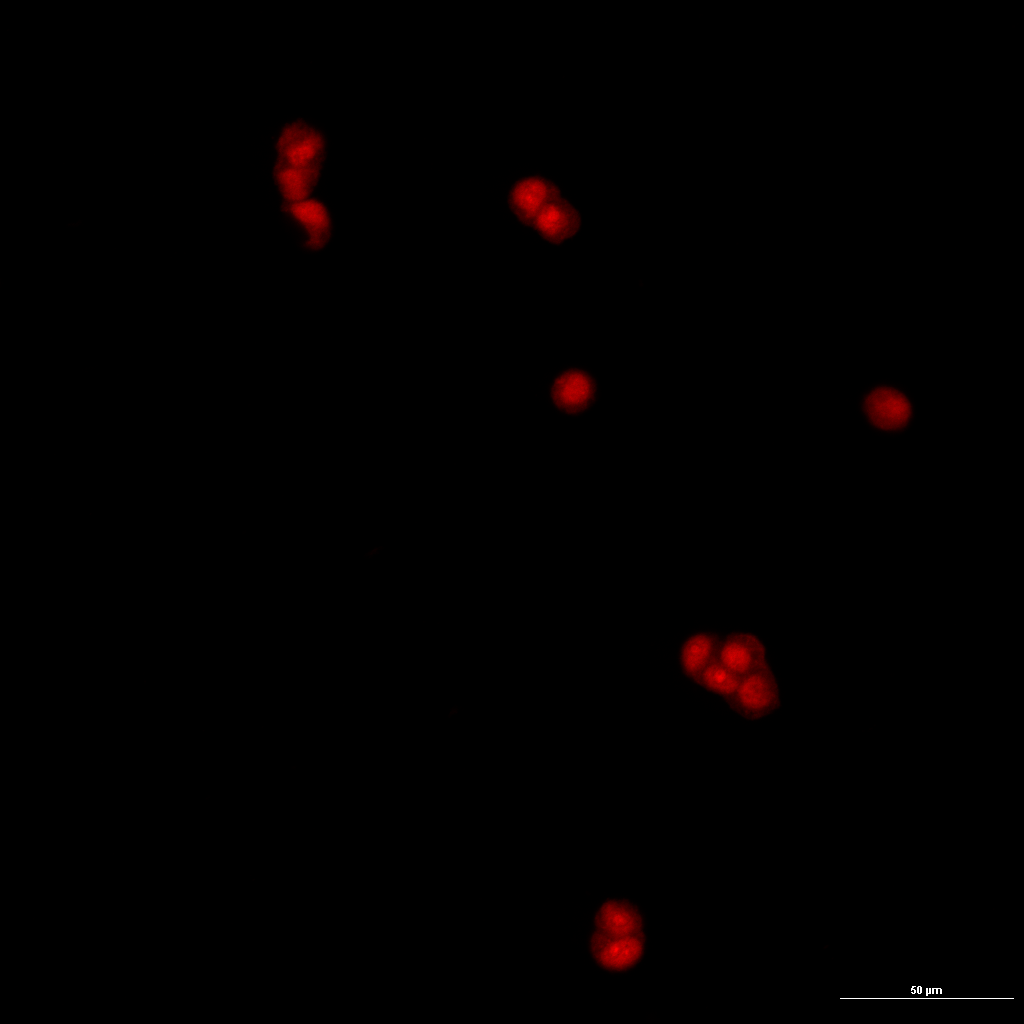

Supplement: Supplementary file 8 [file DataSheet8.zip › IF/IF-Overexpression verification/SW480/OE/4_RGB_Alexa Fluor 594 cadaverine_H2O.tif]

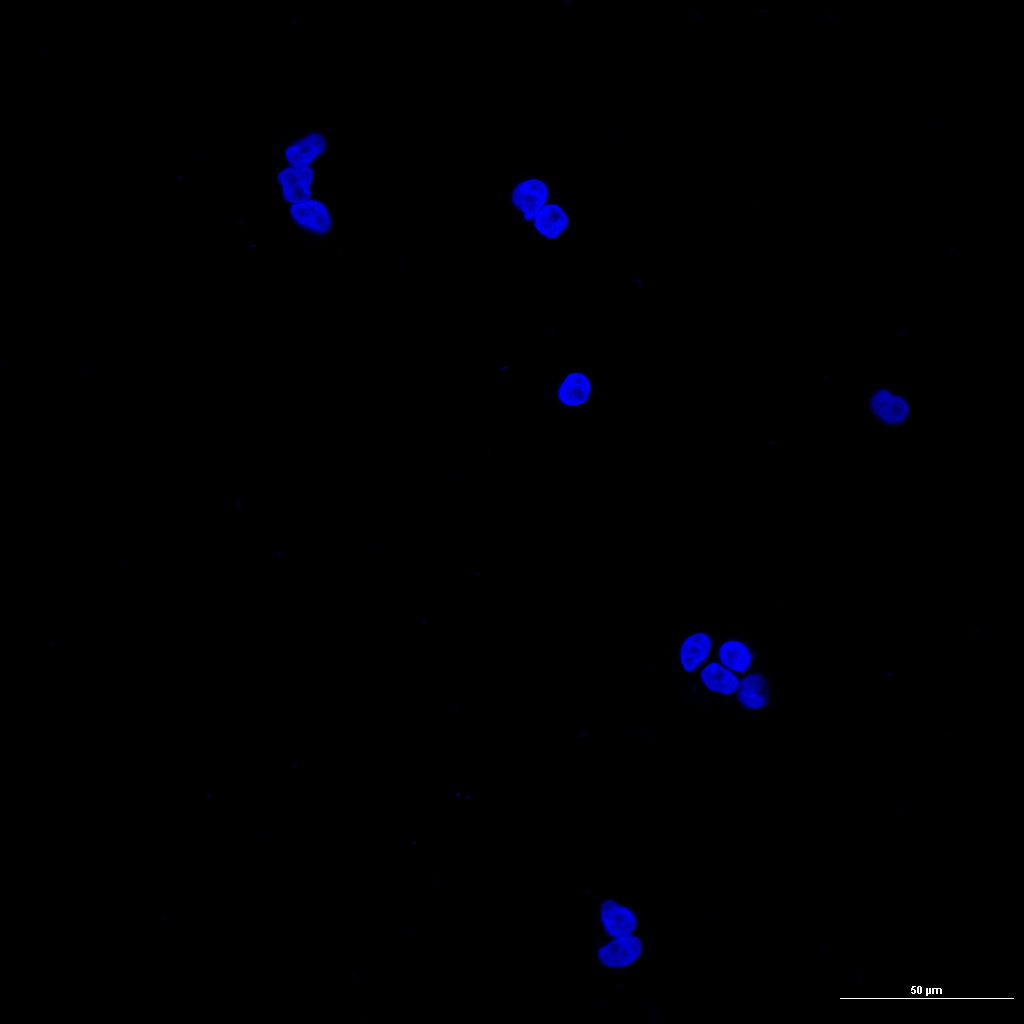

Supplement: Supplementary file 8 [file DataSheet8.zip › IF/IF-Overexpression verification/SW480/OE/4_RGB_DAPI.tif]

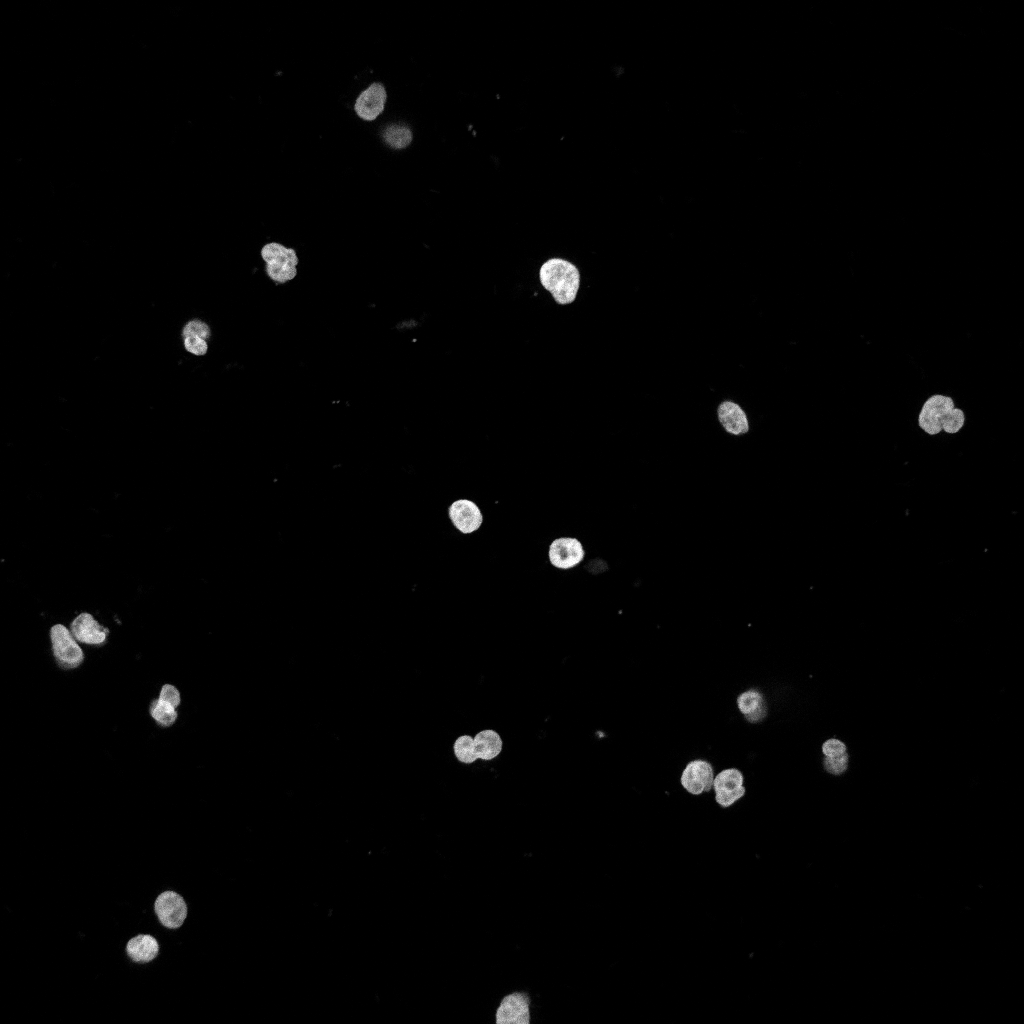

Supplement: Supplementary file 8 [file DataSheet8.zip › IF/IF-Overexpression verification/SW480/Vector/1.tif]

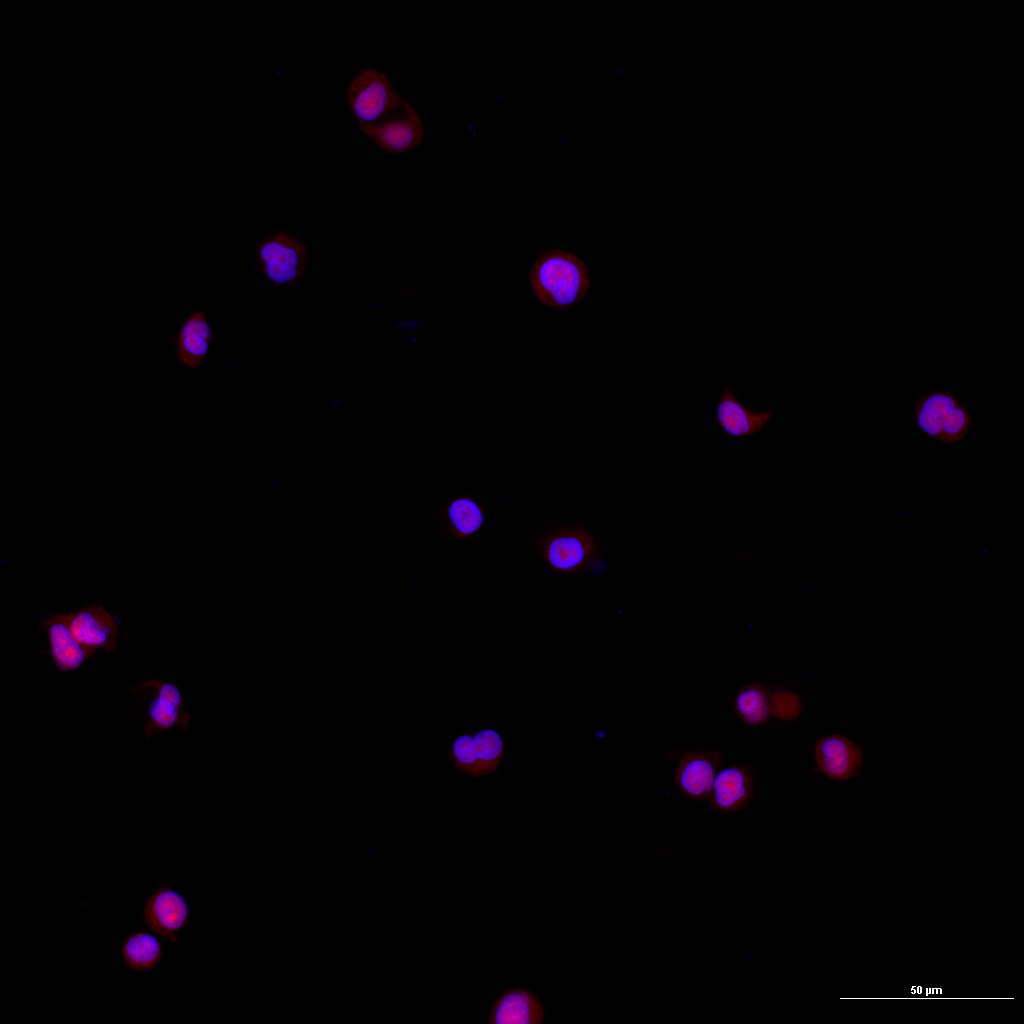

Supplement: Supplementary file 8 [file DataSheet8.zip › IF/IF-Overexpression verification/SW480/Vector/1_RGB.tif]

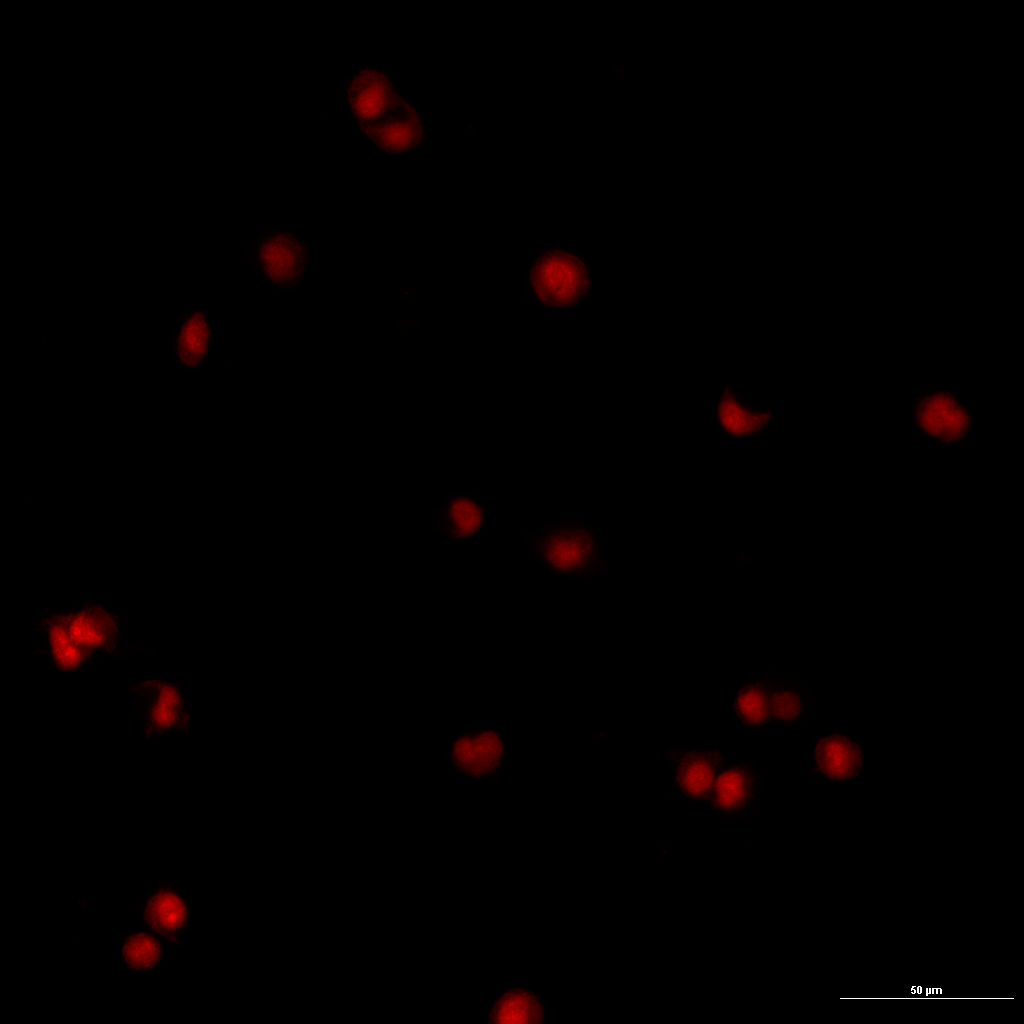

Supplement: Supplementary file 8 [file DataSheet8.zip › IF/IF-Overexpression verification/SW480/Vector/1_RGB_Alexa Fluor 594 cadaverine_H2O.tif]

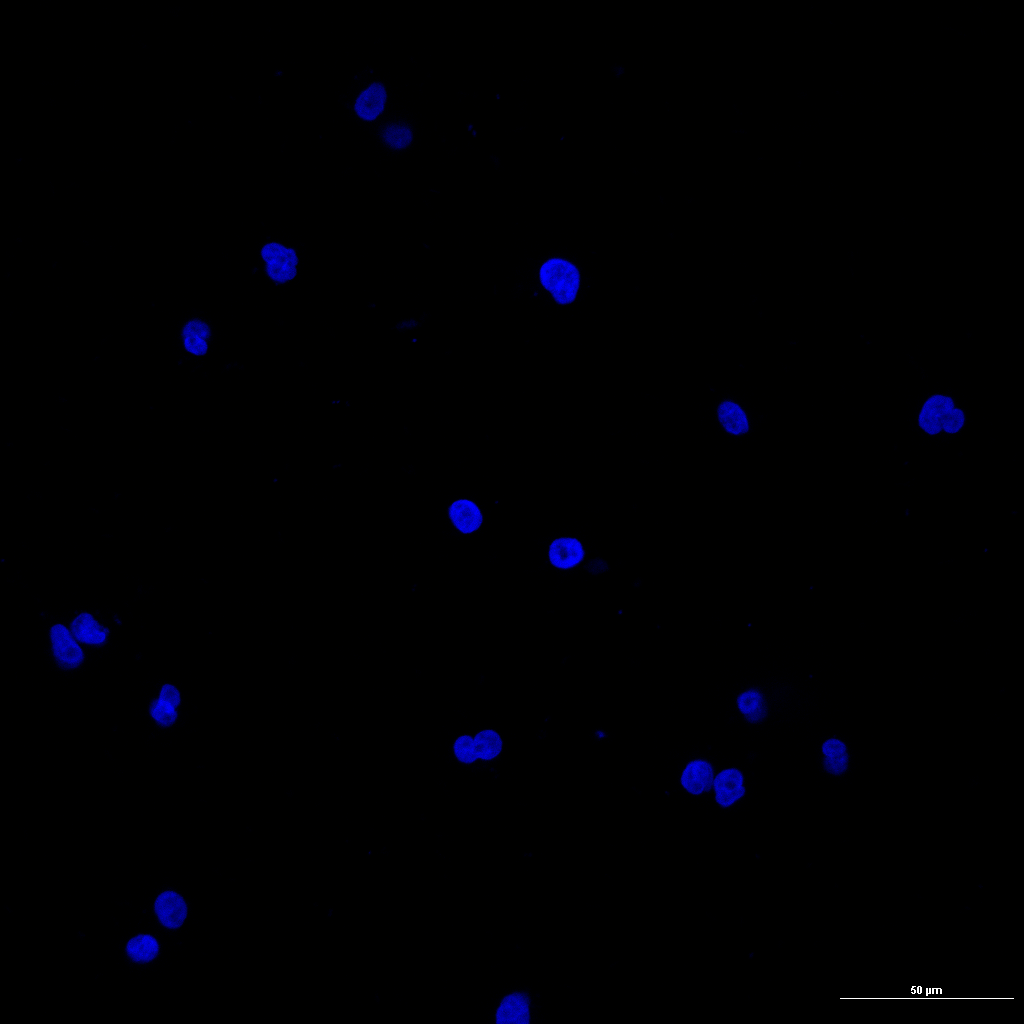

Supplement: Supplementary file 8 [file DataSheet8.zip › IF/IF-Overexpression verification/SW480/Vector/1_RGB_DAPI.tif]

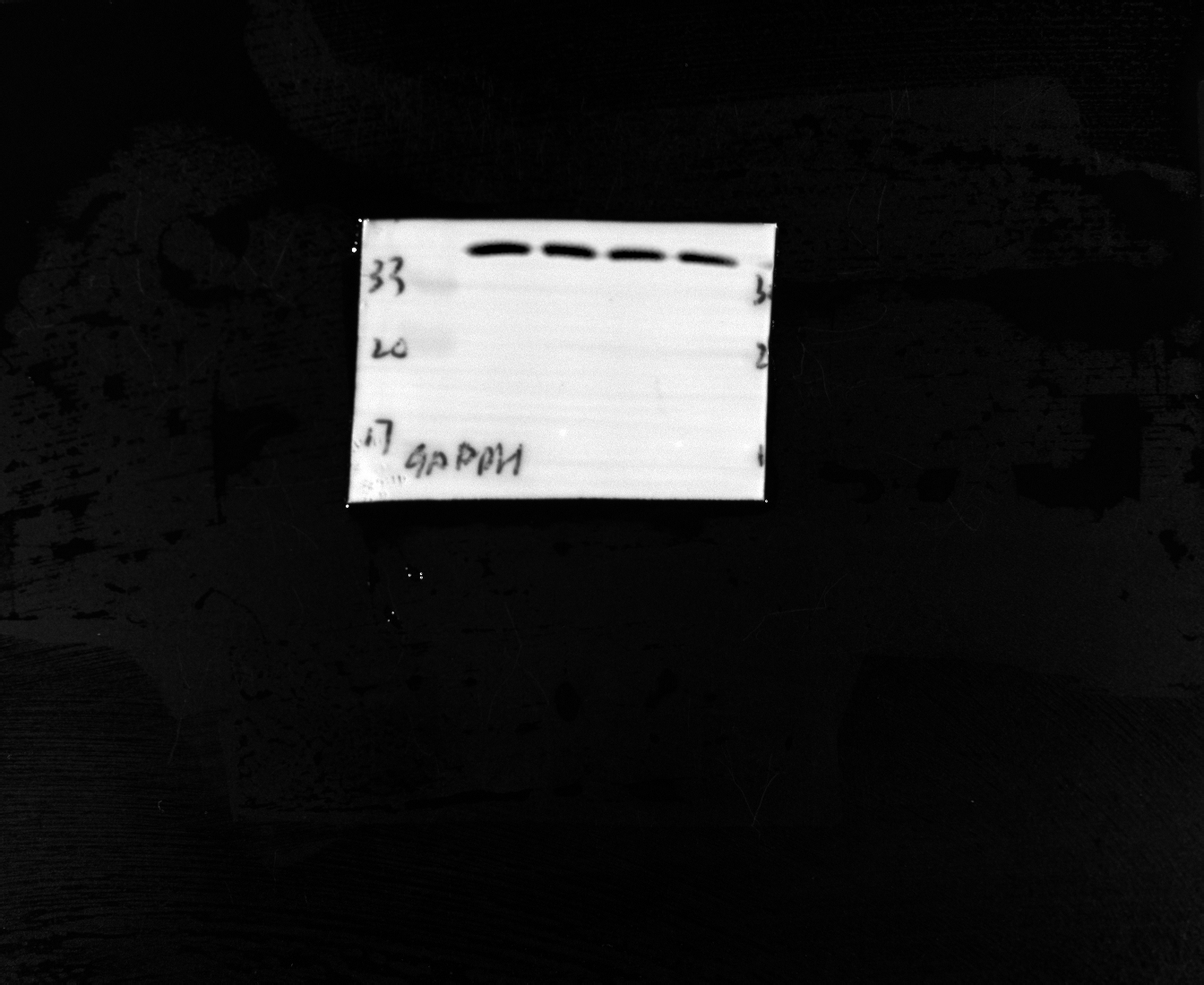

Supplement: Supplementary file 10 [file DataSheet10.zip › Western Blot/Cell transfection-WB/GAPDH.tif]

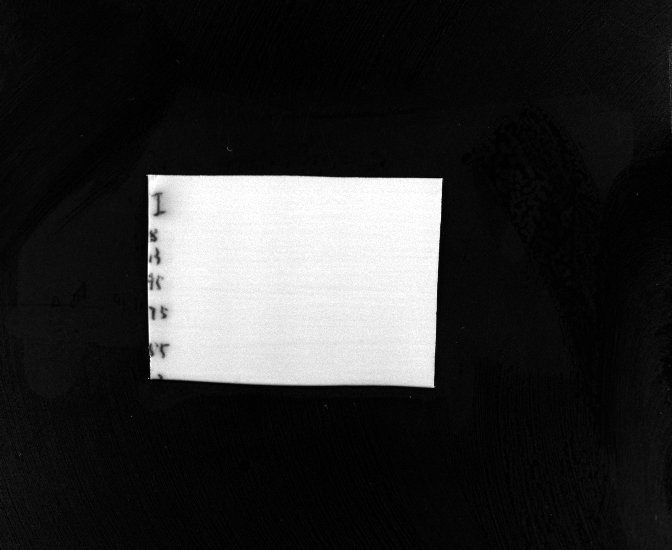

Supplement: Supplementary file 10 [file DataSheet10.zip › Western Blot/Cell transfection-WB/IRX4/016[IRX4]-20241120-173910-brightfield.tif]

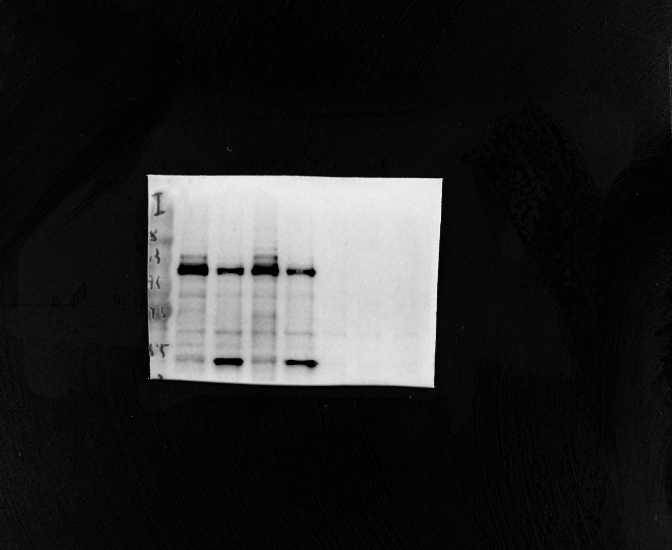

Supplement: Supplementary file 10 [file DataSheet10.zip › Western Blot/Cell transfection-WB/IRX4/016[IRX4]-20241120-173910-luminescence-overlay.tif]

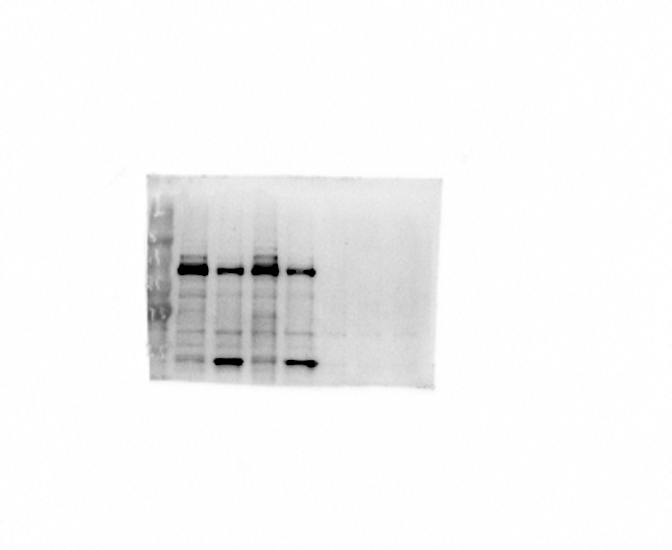

Supplement: Supplementary file 10 [file DataSheet10.zip › Western Blot/Cell transfection-WB/IRX4/016[IRX4]-20241120-173910-luminescence.tif]

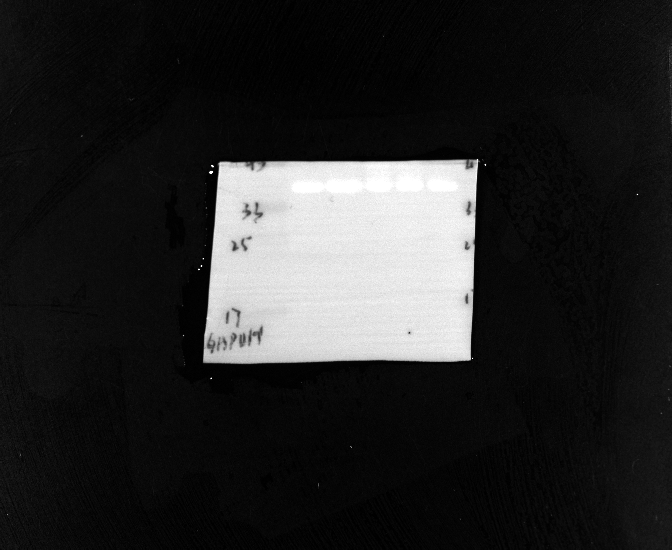

Supplement: Supplementary file 10 [file DataSheet10.zip › Western Blot/Cells-WB/GAPDH/018[GAPDH]-20241120-174316-brightfield.tif]

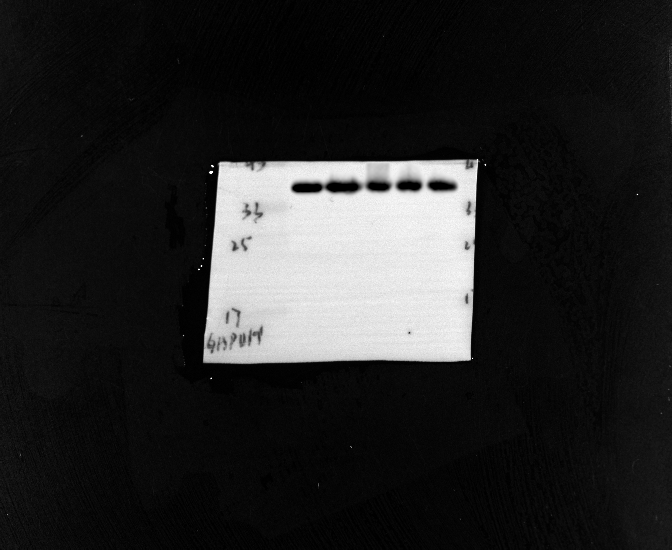

Supplement: Supplementary file 10 [file DataSheet10.zip › Western Blot/Cells-WB/GAPDH/018[GAPDH]-20241120-174316-luminescence-overlay.tif]

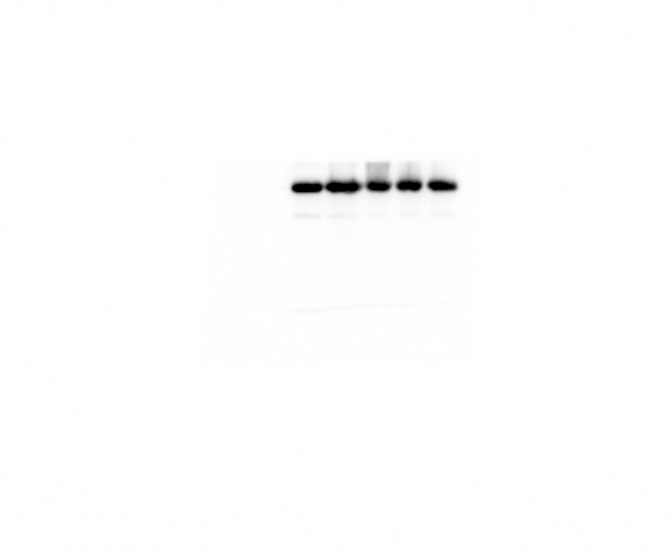

Supplement: Supplementary file 10 [file DataSheet10.zip › Western Blot/Cells-WB/GAPDH/018[GAPDH]-20241120-174316-luminescence.tif]

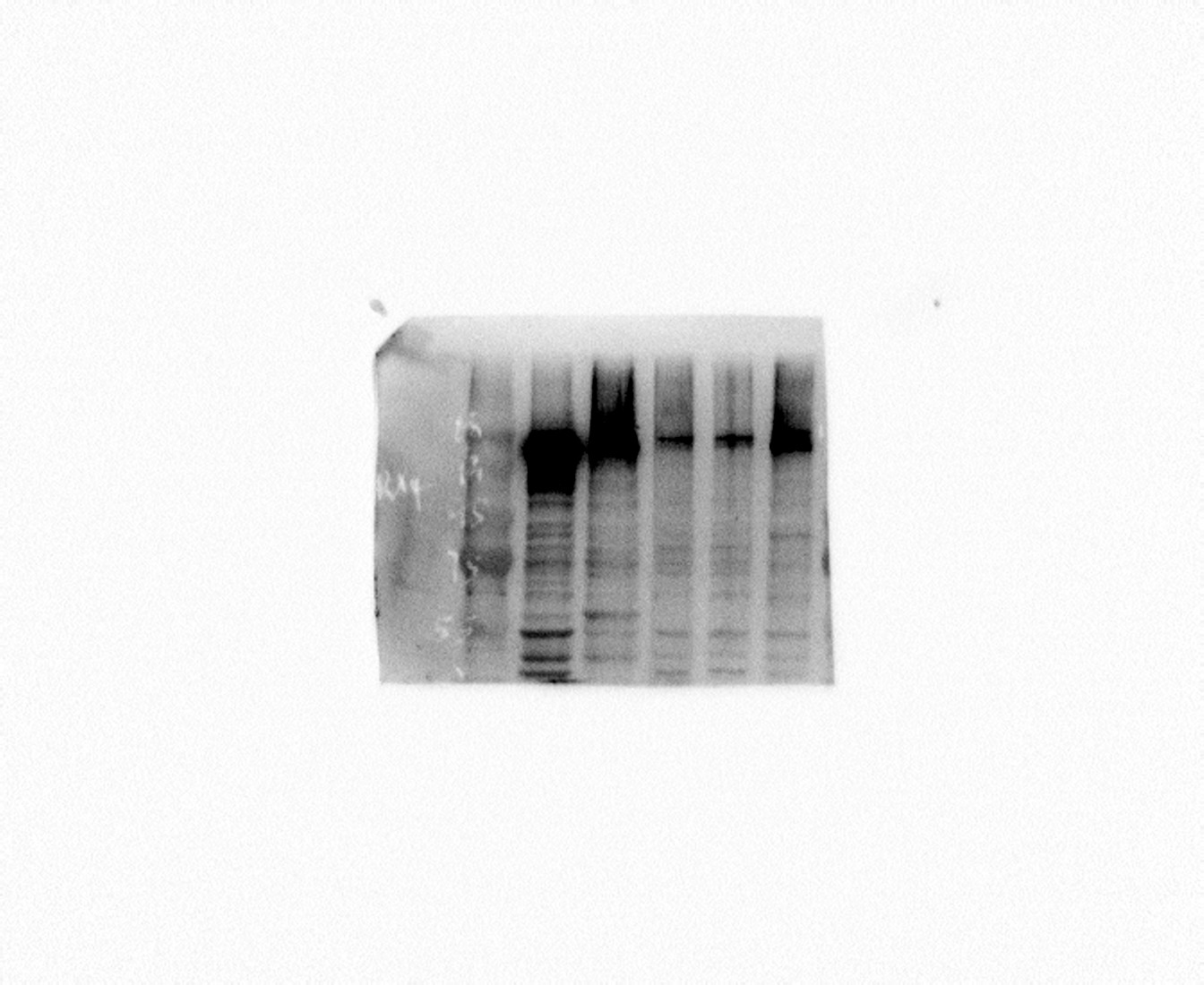

Supplement: Supplementary file 10 [file DataSheet10.zip › Western Blot/Cells-WB/IRX4.tif]

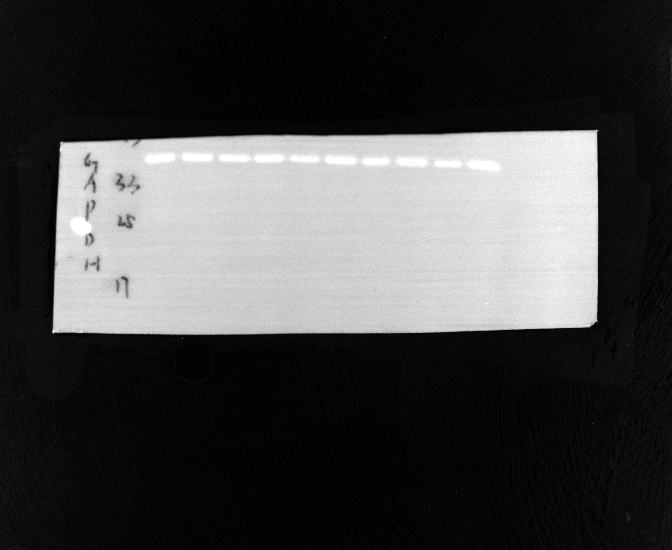

Supplement: Supplementary file 10 [file DataSheet10.zip › Western Blot/Tissues-WB/GADPH/013[GADPH]-20241120-173234-brightfield.tif]

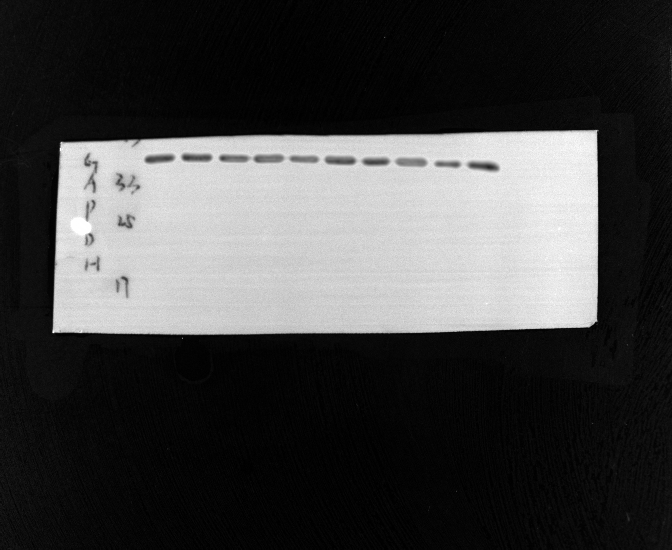

Supplement: Supplementary file 10 [file DataSheet10.zip › Western Blot/Tissues-WB/GADPH/013[GADPH]-20241120-173234-luminescence-overlay.tif]

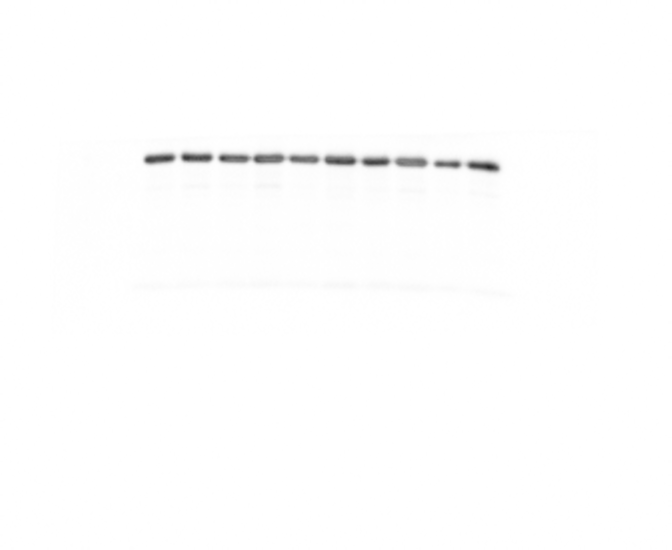

Supplement: Supplementary file 10 [file DataSheet10.zip › Western Blot/Tissues-WB/GADPH/013[GADPH]-20241120-173234-luminescence.tif]

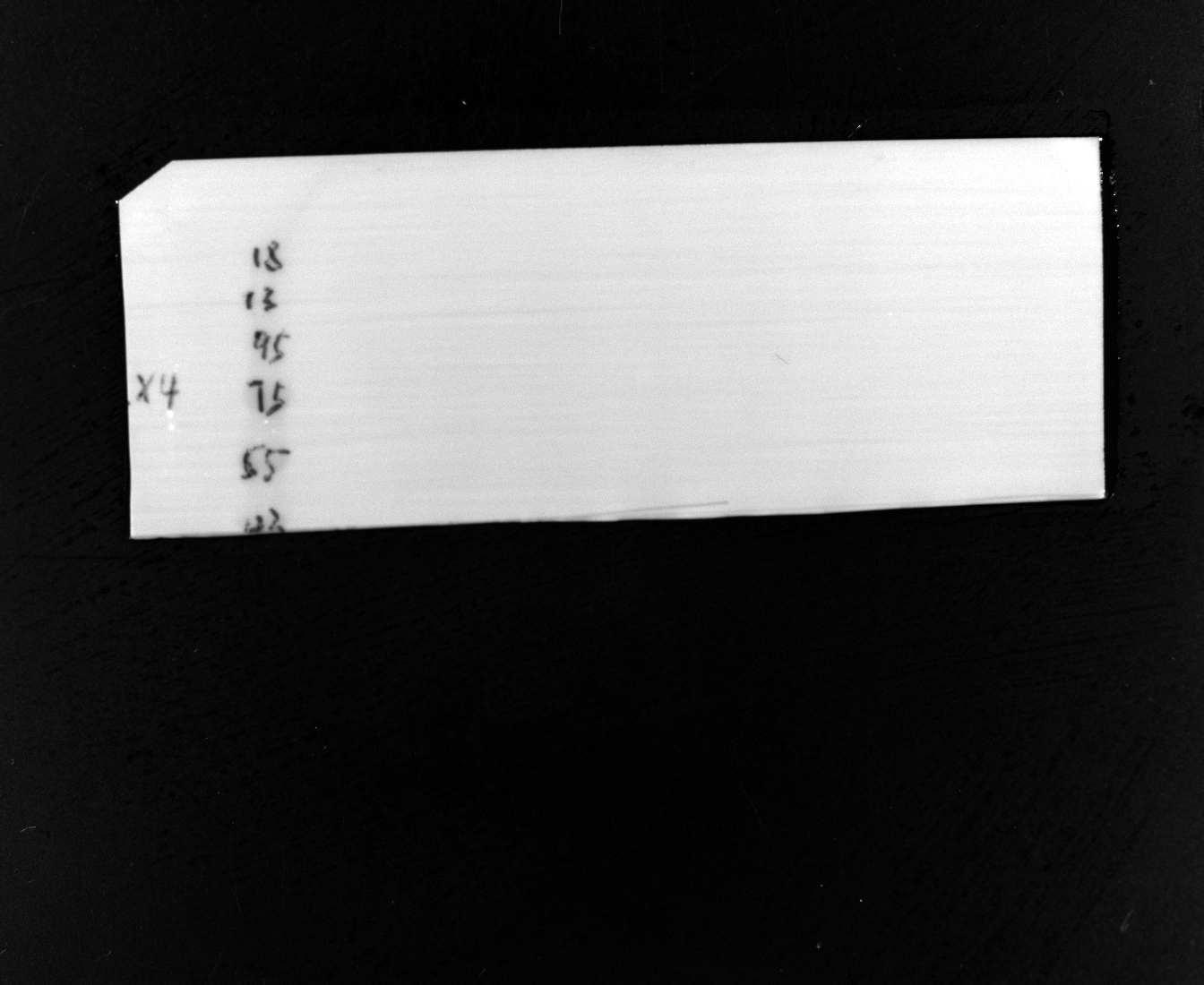

Supplement: Supplementary file 10 [file DataSheet10.zip › Western Blot/Tissues-WB/IRX4/002[IRX4]-20241117-130126-brightfield.tif]

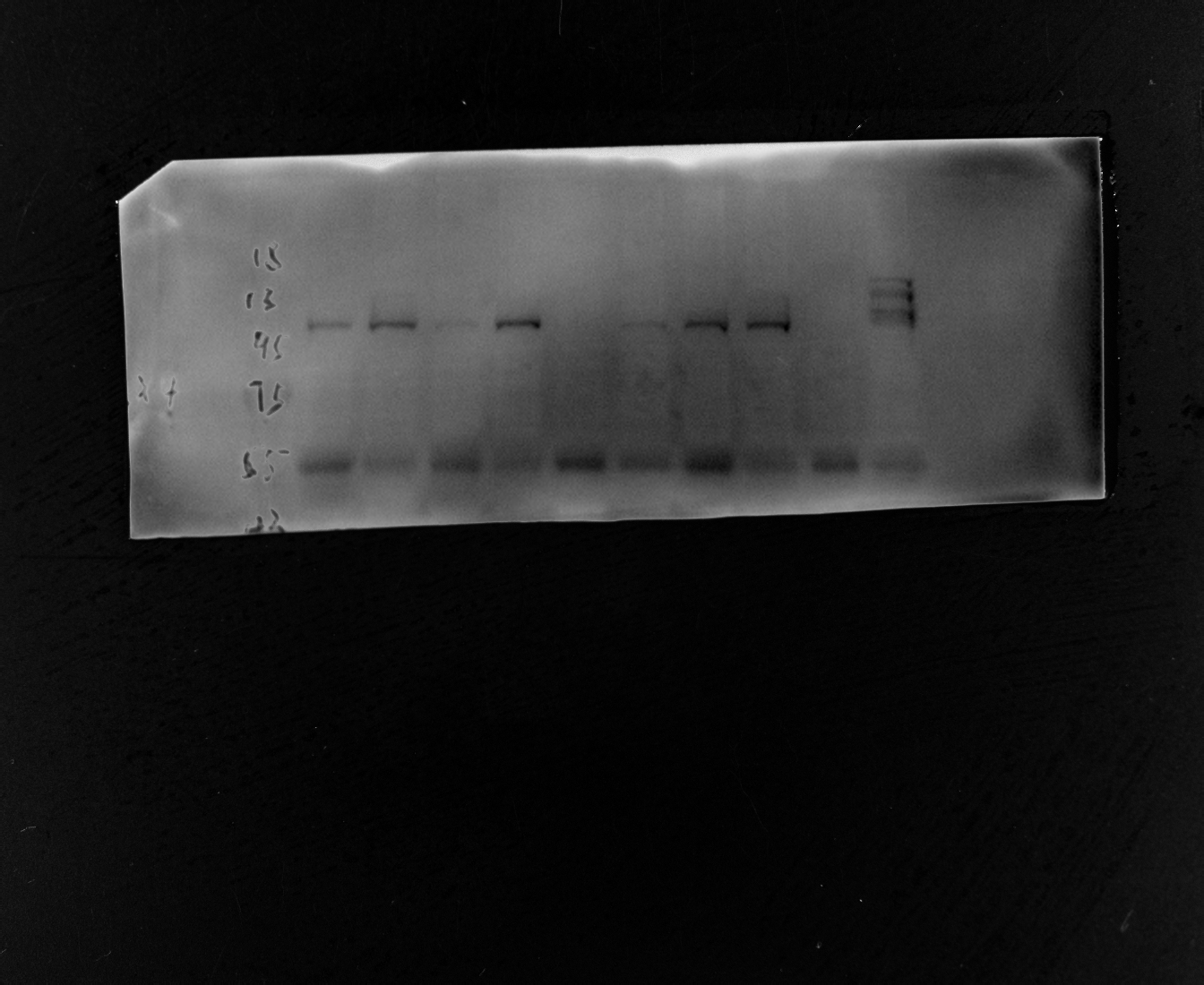

Supplement: Supplementary file 10 [file DataSheet10.zip › Western Blot/Tissues-WB/IRX4/002[IRX4]-20241117-130126-luminescence-overlay.tif]

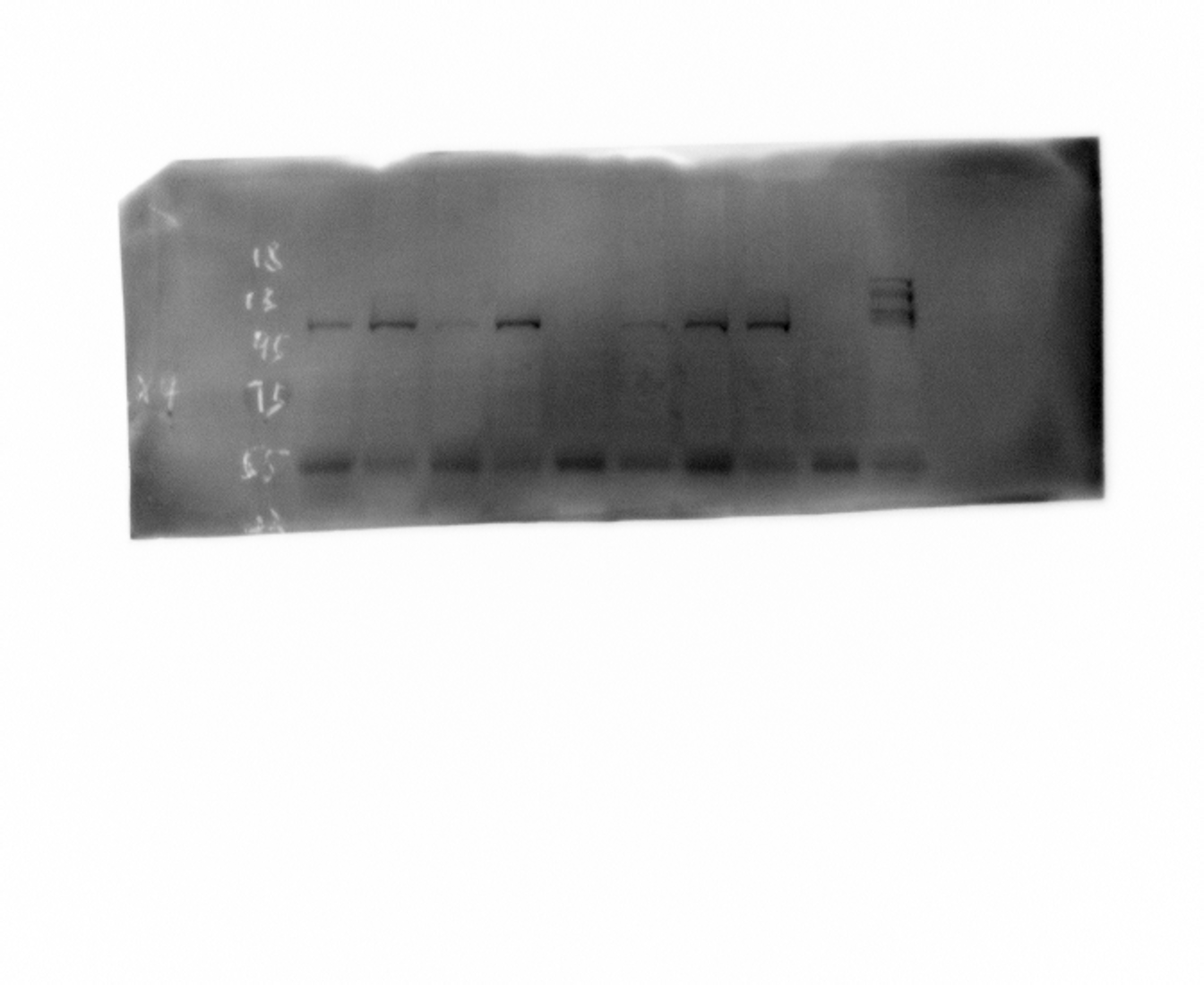

Supplement: Supplementary file 10 [file DataSheet10.zip › Western Blot/Tissues-WB/IRX4/002[IRX4]-20241117-130126-luminescence.tif]
