# Supplementary material for: Anti-colorectal cancer effects of IRX4 and sensitivity studies to oxaliplatin
Source: Front Immunol. 2026 Jan 21;16:1581244. doi: 10.3389/fimmu.2025.1581244 (PMC12867854; doi:10.3389/fimmu.2025.1581244)
Supplement: Supplementary file 11 [file DataSheet11.pdf]

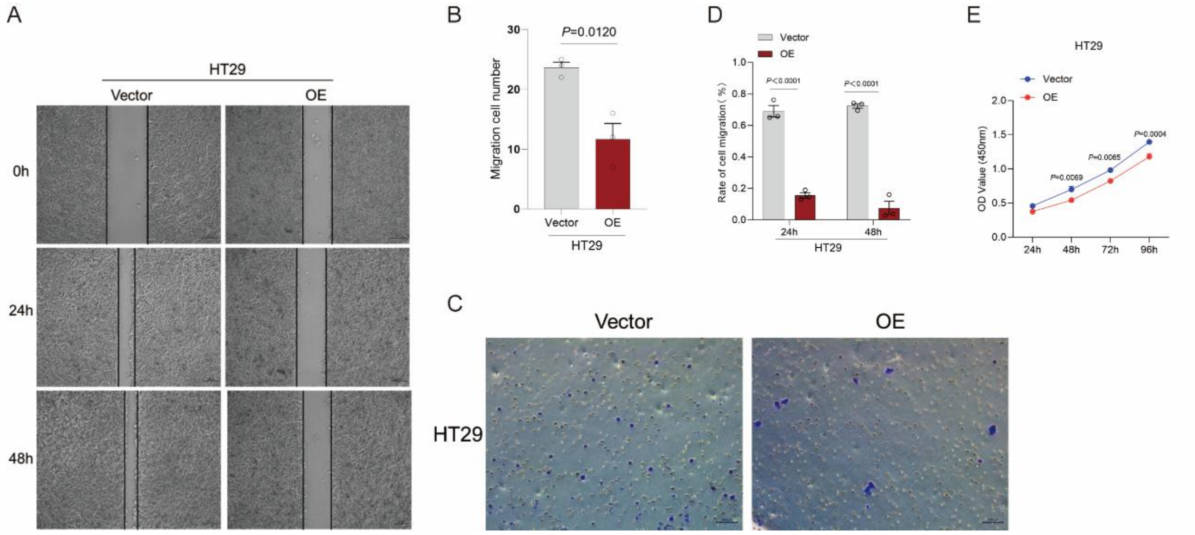

Supplementary Figure1. Functional assays of HT29 cells following IRX4 overexpression. (A, B) Wound closure of HT29 cells at 24 hours and 48 hours after IRX4 overexpression. (C, D) Results of the Transwell assay: Number of invading HT29 cells at 24 h. (E) CCK-8 assay: Cell viability at 0–96 h (mean ± SEM, n=3). Data are presented as the mean ± SEM (n = 3). $P < 0.05$ is considered the difference to be statistically significant.

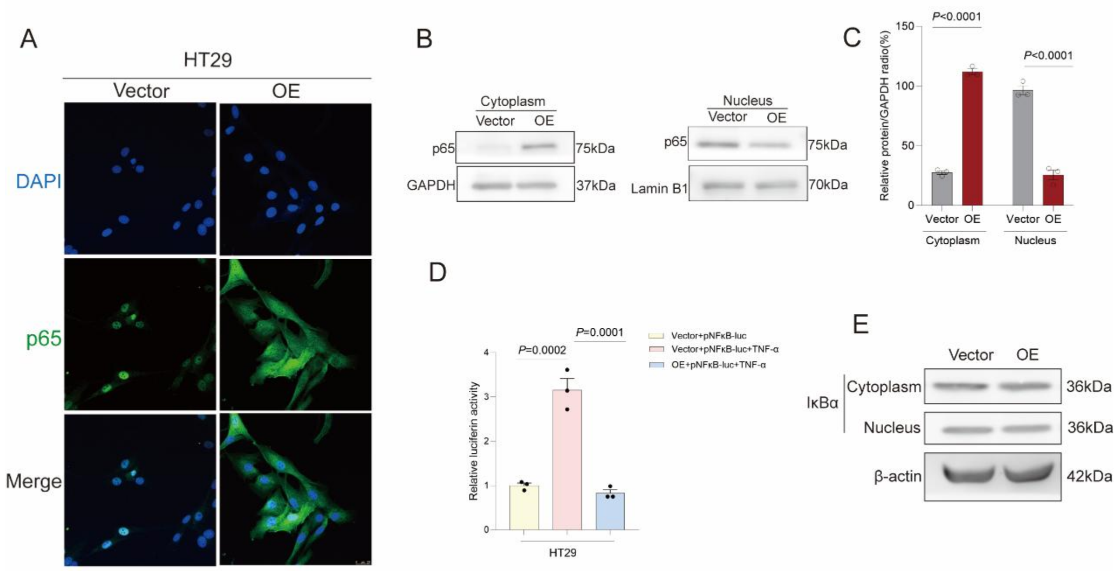

Supplementary Figure 2. Overexpression of IRX4 can inhibit the NF-κB pathway. (A) Detection of nuclear translocation of NF-κB p65 in CRC by IF. Anti-p65 antibody (green) and DAPI (blue) stain p65 and nuclei, respectively; visualized via confocal microscopy (scale bar = 25 μm). (B, C) Western Blot showed that p65 protein expression was down-regulated in nucleus. (D) NF-κB luciferase activity in HT29 cell

line. IRX4 overexpression attenuates TNF-α-induced NF-κB activit. (E) IκBα protein was unchanged after IRX4 overexpression compared with cytoplasm. Data are presented as the mean ± SEM (n=3). $P < 0.05$ is considered the difference to be statistically signficant.

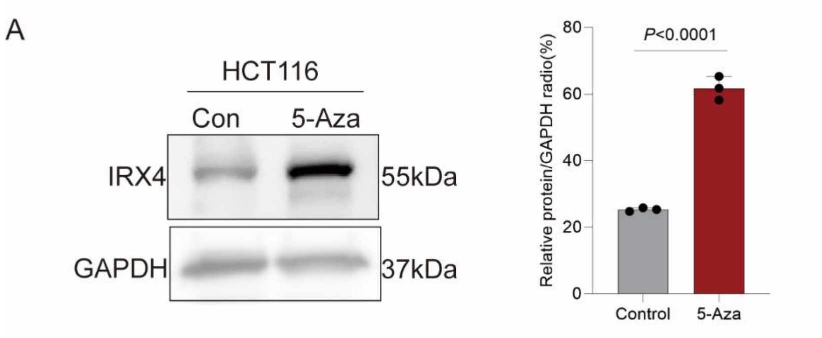

Supplementary Figure 3. (A) 5-Aza treatment induces re-expression of IRX4 gene in HCT116 cells. IRX4 re-expression occurs after 5-Aza treatment in HCT116 cells. Data are presented as the mean ± SEM (n=3). $P < 0.05$ is considered the difference to be statistically signficant.

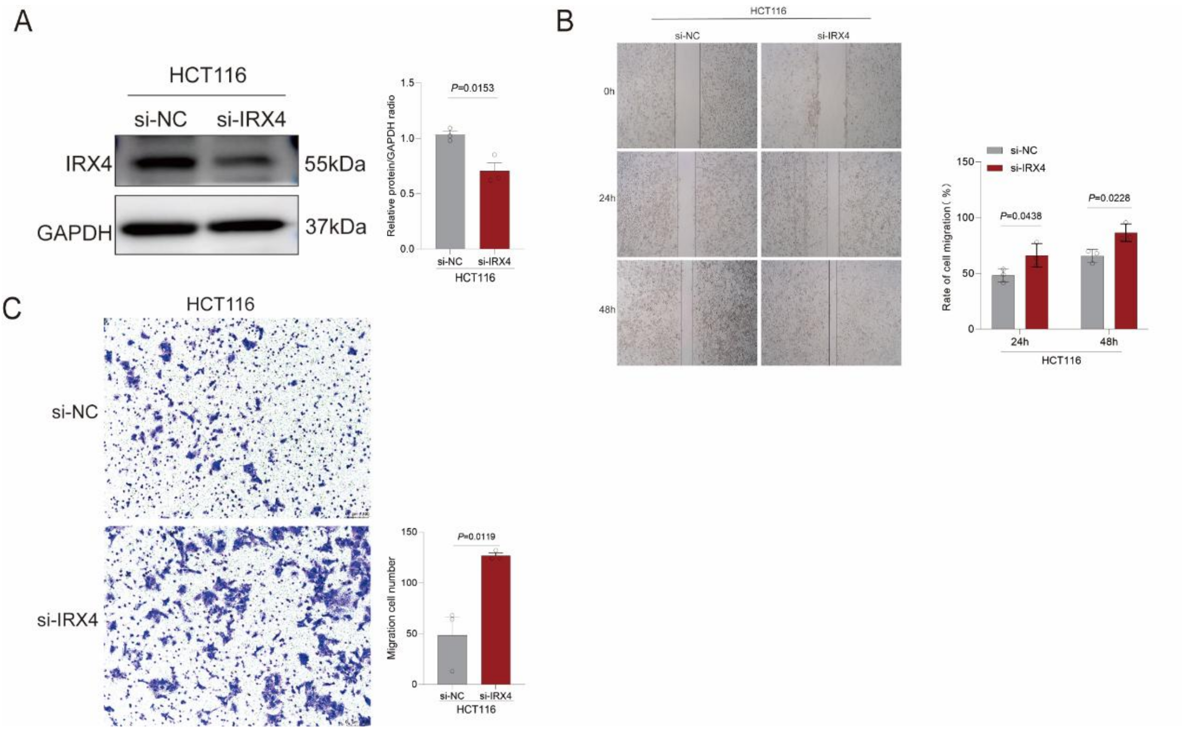

Supplementary Figure 4. Knockdown of the IRX4 gene significantly induced phenotypic alterations in HCT116 cells. (A) Western blot analysis revealed a notably reduced transfection efficiency of siRNA in the si-IRX4 group compared to the negative control group (si-NC) in HCT116 cells. (B) Wound healing assay demonstrated

enhanced migration of HCT116 cells upon IRX4 downregulation. (C) Transwell assay indicated increased invasion of HCT116 cells following IRX4 downregulation. Data are presented as the mean ± SEM (n = 3). *P* < 0.05 is considered the difference to be statistically significant.

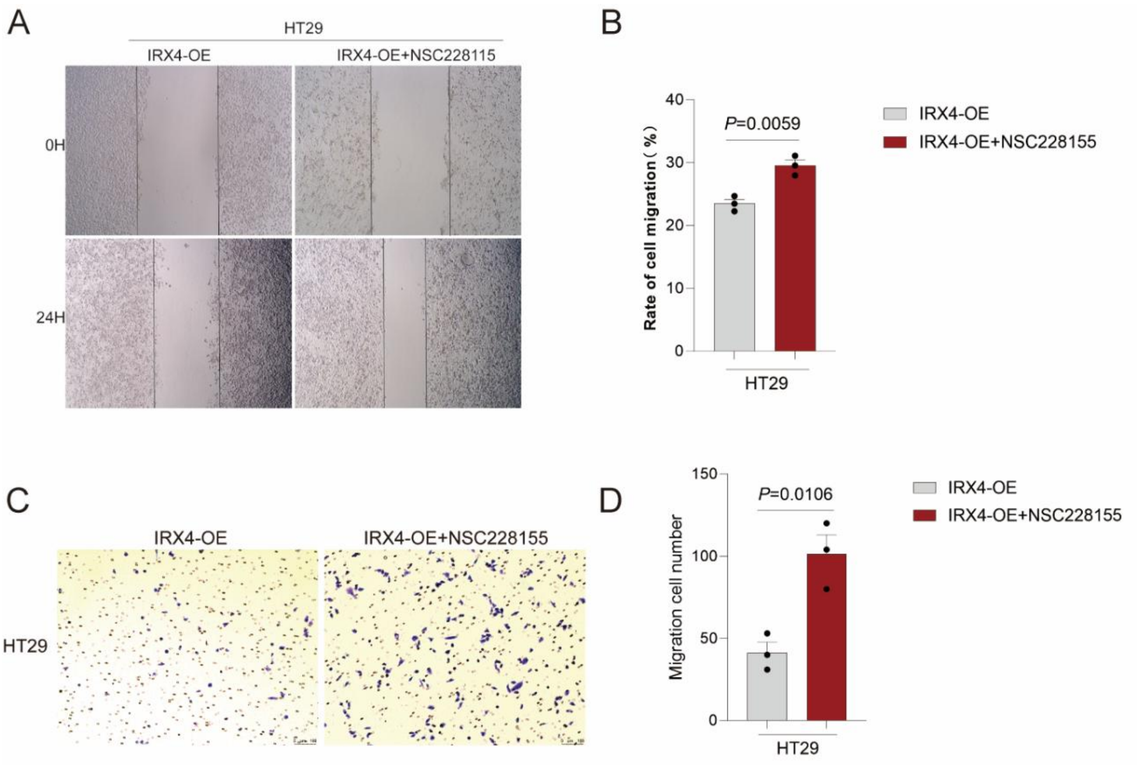

Supplementary Figure 5. Activation of EGFR by NSC228155 significantly reversed the inhibition of IRX4 overexpression on cell migration and invasion. (A, B) The effect of NSC228155 on the migration ability of HT29 cells was examined by scratch test. (C, D) Transwell invasion assay was used to detect the effect of NSC228155 on the invasion ability of HT29 cells. Data are presented as the mean ± SEM (n = 3). *P* < 0.05 is considered the difference to be statistically significant.

Kits and Antibodies

Cell viability was assessed using the Cell Counting Kit-8 (CCK-8, SC119; SEVEN). Proliferation was evaluated with the Elab Fluor® 594 E-Click EdU Cell Proliferation Imaging Assay Kit (E-CK-A377; Elabscience). Apoptosis was measured using the AnnexinV-FITC/7-AAD Apoptosis Detection Kit (Biolegend). Luciferase reporter assay kit (Beyotime, C0533, China) was to test the firefly luciferase activities.

The primary antibodies were as follows: IRX4(1:1500, PA5-97879; Invitrogen), GAPDH (1:20000, 60004-1-IG; Proteintech), Lami B1(1:10000, 12987-1-AP; Proteintech), NF-κB p65(1:5000, 80379-2-RR; Proteintech), IκBα(1:5000, 10268-1-AP; Proteintech), anti-EGFR (1:1000, 18986–1-AP; Proteintech), anti-phospho-EGFR (1:1000, 84906–1-AP; Proteintech), anti-AKT(1:1000, 10176–2-AP; Proteintech), anti-phospho-AKT (1:1000, 66444-1-IG; Proteintech), anti-ERK (1:1000, 11257-1-AP; Proteintech), anti-phospho-ERK (1:1000, 28733-1-AP; Proteintech). The secondary antibodies included horseradish peroxidase (HRP)-conjugated goat anti-rabbit IgG (H + L) (1:20000, 31402; Thermo Fisher) and HRP-conjugated goat anti-mouse IgG (H + L) (1:10000, SA00001-7H; Proteintech). All antibodies were stored at -20°C.

Primer Sequences

Supplementary Table 1 Primer Sequences

Pyrosequencing Primers
Fragment 1 Primers
Forward Primer: 5'-GAGGAGTAGGGGTATTTAAATTG- 3'
Reverse Primer: 5'-ATAAACTAACCCACTCTCCTACTTC-3'
Sequencing Primer: 5'-TATTTAAATTGAGGGTAGGATATG- 3'
Fragment 2 Primers
Forward Primer: 5' -GGAGGGAGAAGTAGGAGAGT - 3'
Reverse Primer : 5'-ATAAAACTAACCTCAAAACCTCCTCC-3'
Sequencing Primer: 5' -GAGGGGGTTTTGGGA - 3'
RT-qPCR Primers
IRX4 Primers
Forward: 5'-CAAGAACCCCTACCCCACC-3'
Reverse: 5'-CTTCTCCTCTTTGCCCACG-3'
GAPDH Primers ：
Forward: 5'-GGAGCGAGATCCCTCCAAAAT-3'
Reverse: 5'-GGCTGTTGTCATACTTCTCATGG-3'
Plasmid Sequencing and Cloning Primers

pcDNA3.1-F: CTAGAGAACCCACTGCTTAC

pcDNA3.1-R(BGH-R): TAGAAGGCACAGTCGAGG
