# Supplementary material for: Anti-colorectal cancer effects of IRX4 and sensitivity studies to oxaliplatin
Source: Front Immunol. 2026 Jan 21;16:1581244. doi: 10.3389/fimmu.2025.1581244 (PMC12867854; doi:10.3389/fimmu.2025.1581244)
Supplement: Supplementary file 12 [file Table1.docx]

Dear Editor,

We could like to submit the enclosed manuscript entitled “Anti-colorectal cancer effects of IRX4 and sensitivity studies to oxali-platin”. No conflict of interest exits in the submission of this manuscript, and manuscript is approved by all authors for publication.I would like to declare on behalf of my co-authors that the work described was original research that has not been published previously, and not under consideration for publication elsewhere, in whole or in part.

I outline briefly below the significant findings reported in this manuscript and reasons why this manuscript deserves serious consideration for publication.

This study investigates the role of IRX4 methylation in colorectal cancer (CRC) progression and chemotherapy resistance. Using data from methylation microarrays, bioinformatics analysis, and pyrophosphate sequencing, the study found that hypermethylation of IRX4 is associated with CRC pathogenesis. Overexpression of IRX4 inhibited CRC cell proliferation, migration, and invasion, while promoting apoptosis. Low IRX4 expression was linked to poor prognosis in CRC patients, and a negative correlation was observed between IRX4 expression and tumor invasion depth. Furthermore, IRX4 overexpression enhanced the cytotoxicity of oxaliplatin (OXA), improving chemotherapy efficacy in CRC cells, suggesting its potential as a therapeutic target for CRC treatment.I hope this paper is suitable for “Frontiers in Immunology”.

We deeply appreciate your consideration of our manuscript, and we look forward to receiving comments from the reviewers. If you have any problems, please don’t hesitate to contact me at the address below.

Thank you and best regards.

Yours sincerely,

Corresponding author: Yabin Liu

E-mail: [liuyabin@hebmu.edu.cn](mailto:liuyabin@hebmu.edu.cn)
